# Dialkyl Ethers and Sacrificial Zinc: Oxide Synthons for Calcium *tert*‐Butoxide Formation

**DOI:** 10.1002/anie.9268230

**Published:** 2026-09-08

**Authors:** Kyle G. Pearce, Agustín Morales, Michael S. Hill, Claire L. McMullin

**Affiliations:** ^1^ Department of Chemistry University of Bath Bath UK

**Keywords:** alkyl, calcium, density functional theory, heterobimetallic, hydride, zinc

## Abstract

The calcium hydride, [(BDI)CaH]_2_ (BDI = HC{C(Me)NDipp}_2_, where Dipp = 2,6‐*i‐*Pr_2_C_6_H_3_), reacts with Zn*t*‐Bu_2_ to form the monomeric and toluene‐stabilised calcium dialkyl(hydrido)zincate, [(BDI)Ca‐μ‐H‐Zn(*t*‐Bu)_2_(η^6^‐C_7_H_8_)]. When treated with dialkyl ethers, this compound initiates a sequence of twofold ether deprotonation and dealkylation by alkene extrusion, from which the resultant oxide dianion is sequestered as a *tert*‐butoxide anion. Through a combination of experiment and theory, this completely selective C─O bond forming process is deduced to result from the formation of incipient calcium *tert*‐butyl and hydride intermediates, and subsequent tertiary carbenium ion generation enabled by reduction of Zn(II) to elemental zinc. The overall transformation, therefore, may be rationalised as a consequence of latent extreme basicity in harness with a cooperative redox process that is intrinsic to the combination of dissimilar constituent metals.

## Graphical abstract

When treated with dialkyl ethers, [(BDI)Ca‐μ‐H‐Zn(*t*‐Bu)_2_(η^6^‐C_7_H_8_)] (BDI = HC{C(Me)NDipp}_2_ initiates a sequence of twofold ether dealkylation and oxide sequestration as *tert*‐butoxide in a process predicated on a combination of extreme basicity and a cooperative redox process administered by the dissimilar constituent metals. (Generative AI (ChatGPT) was used to render the image of an ingot of zinc).

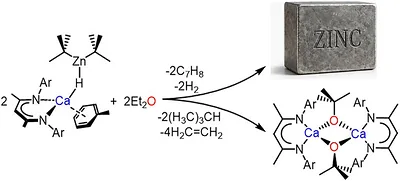

## 1 Introduction

The preparation of Grignard reagents is historically dependent upon the use of polar and solubilising solvent media, especially diethyl ether and THF [1]. Although the solvent is usually considered an inert spectator in organo‐magnesium‐based synthesis, it has long been known that many polar organometallics, for example organolithiums and their heavier group 1 congeners, can induce the degradation of aliphatic and cyclic ethers, even under low temperature conditions [2, 3, 4, 5, 6, 7, 8, 9]. While these processes can be capricious and their outcomes unpredictable, the general mechanistic consensus favours a sequence of either α‐ or β‐C─H deprotonation, or even intramolecular α‐β′ tautomerisation, prior to alkene elimination (e.g., Scheme 1a) [3, 10]. Although this behaviour most commonly results in an intractable mixture of alkenes, lithium alkoxides or enolates, Mulvey and co‐workers have shown that oxide and dimetallated butadiene products resulting from THF cleavage may be ‘captured’ and fully characterised through the use of bimetallic (Na/Mg or Na/Mn) bases (Scheme 1b) [11].

**SCHEME 1:**
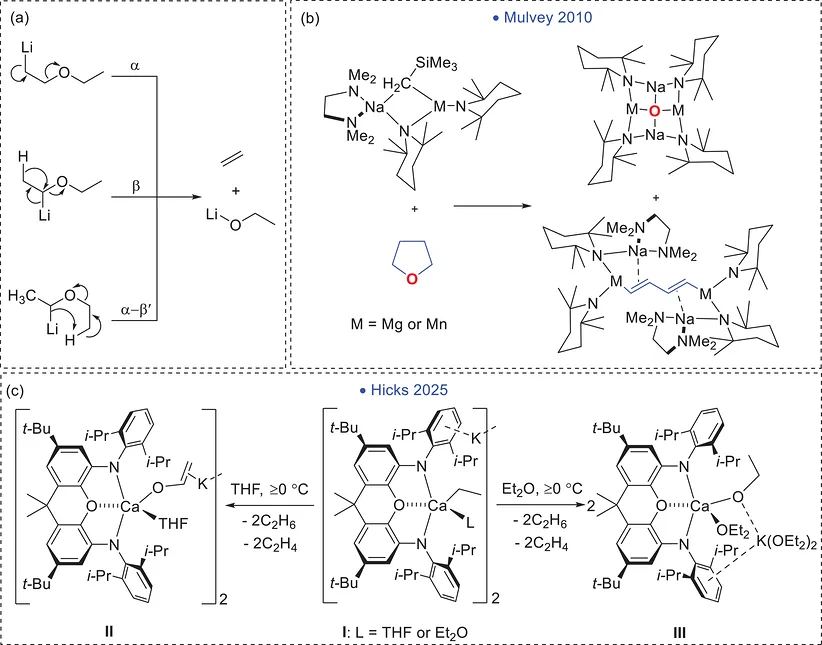
(a) Postulated modes of alkene extrusion from lithiated diethyl ether; (b) Mulvey's THF ‘cleave and capture’ by bimetallic (Mg/Na and Mn/Na) bases [11]; (c) Hicks’ ethylcalciate (I) and its reactivity toward THF and diethyl ether to form compounds, **II** and **III** [12].

Similar issues of ether cleavage are commonly encountered, and even exacerbated, for organoderivatives of the heavier elements of group 2 [13, 14, 15, 16]. Eaborn and Smith's solvent‐free [Ca{C(SiMe_3_)_3_}_2_], for example, was reported to react immediately with diethyl ether to give a mixture of HC(SiMe_3_)_3_, ethene and Ca(OEt)_2_ [17], while Westerhausen and co‐workers’ successful isolation of calcium mono‐ and di‐aryls also led to a variety of THF‐stabilised calcium oxide clusters in which the O^2–^ anion was speculated to arise from the cyclic ether solvent [18, 19, 20]. More recently, Hicks and co‐workers have reported that in situ generation of the potassium ethylcalciate species (**I**) by treatment of its hydride analogue with ethene, and in the presence of either THF or diethyl ether, results in the respective generation of the dimeric ethenolate and monomeric ethoxide derivatives, **II** and **III** (Scheme 1c) [12]. Consistent with fragmentation processes initiated through ether deprotonation by the highly basic ethylcalciate moiety, the formation of **II** and **III** was accompanied by the observable generation of ethane and ethene.

On a similar theme, we have developed a number of complementary routes to highly reactive calcium alkyls and aryls supported by the β‐diketiminate ligand, HC{C(Me)NDipp}_2_ (BDI, where Dipp = 2,6‐*i‐*Pr_2_C_6_H_3_) [21, 22, 23]. Reactions of the base‐free hydride, [(BDI)CaH]_2_ (**IV**), with terminal alkenes, including ethene, provide a reliable route to the corresponding dimeric primary *n*‐alkyls, which are sufficiently nucleophilic to alkylate even benzene [21, 24, 25]. Reactivity derived from Ca─H/C═C insertion, however, is clearly inapplicable to the synthesis of the lightest methyl homologue, [(BDI)CaMe]_2_, while reactions with internal alkenes to yield potentially more basic secondary alkyls were either unsuccessful or complicated by intramolecular BDI ligand activation [23]. To address this limitation, therefore, we have established that compound **IV** also undergoes hydride transmetallation with more readily accessible organo, amido and phosphido group 12 derivatives [26, 27, 28, 29, 30, 31]. Reactions with ZnR_2_ (R = Me, Et), for example, occur in a stepwise manner through the initial formation of isolable calcium dialkyl(hydrido)zincate intermediates, which equilibrate to the alkyls, [(BDI)CaR]_2_, by extrusion of the relevant alkyl(hydrido)zinc (Scheme 2) [28, 29]. Although the calcium methyl and ethyl species prepared by this method were prone to further transmetallation to [(BDI)ZnR], both processes of onward equilibration were suppressed for the reaction derived from Zn(*i‐*Pr)_2_ by the precipitation of the initially formed calcium di‐*iso*‐propyl(hydrido)zincate dimer (Scheme 2) [29].

**SCHEME 2:**
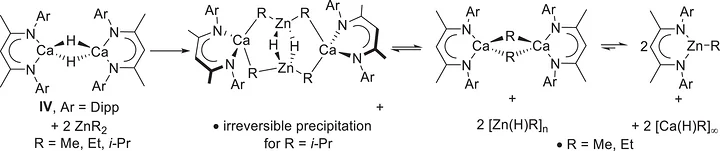
Reactivity of **IV** with ZnR_2_ (R = Me, Et, *i‐*Pr).

In this contribution, we extend this chemistry to the reaction of **IV** with Zn(*t*‐Bu)_2_. We report that the resultant calcium zincate species adopts a contrasting monomeric structure and effects twofold alkene elimination and formal oxide abstraction from dialkyl ethers resulting in the entirely selective generation of a calcium *tert*‐butoxide. Furthermore, this process is deduced by a joint experimental and theoretical study to be predicated on both the extreme basicity of putative terminal *tert‐*butylcalcium and hydridocalcium intermediates. Crucially, these processes operate in conjunction with the ability of Zn(II) to act as a two‐electron sink for the effective oxidation of a *tert*‐butyl anion to its equivalent tertiary carbenium ion.

## 2 Results and Discussion

Following an analogous procedure leading to the dimeric calcium dialkyl(hydrido)zincate species (Scheme 2), a reaction was performed in toluene between **IV** and two molar equivalents of Zn(*t*‐Bu)_2_. Cooling of the resultant pale‐yellow solution to −35°C induced the crystallisation of compound **1** in a good yield (83%) (Scheme 3). Analysis of the resultant colourless crystals by ^1^H and ^13^C NMR spectroscopy implied the stoichiometric incorporation of both reactants, most clearly demonstrated by the ^1^H NMR signals for the BDI γ‐methine (*δ*_H_ = 4.77 ppm), hydride (4.05 ppm) and *tert‐*butyl (1.61 ppm) environments that resonated as a series of singlets in a respective 1:1:18 ratio of intensities by relative integration.

**SCHEME 3:**
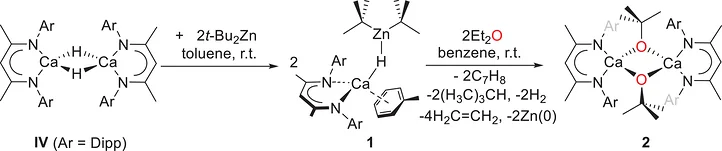
Synthesis of compounds **1** and **2**.

This inference was confirmed by single crystal x‐ray diffraction analysis (Figure 1), which further revealed that, unlike the previously described dimeric examples of calcium dialkyl(hydrido)zincates [28, 29], compound **1** is a monomer in which a single di‐*t‐*butyl(hydrido)zincate interacts via Zn‐μ_2_‐H─Ca bridging to a unique BDI‐coordinated calcium centre. The coordination sphere of the Ca1 atom is further satisfied by its η^6^‐engagement (Ca1···C_cent_ 2.625 Å) with a single molecule of the toluene solvent. Although a number of similarly coordinated calcium‐arene interactions have now been documented, a majority of these reports feature the alkaline earth centre as an explicit cation with charge balance maintained by a weakly or non‐coordinating anion [32, 33, 34, 35]. The only directly comparable charge neutral molecular species, therefore, are provided by a toluene‐coordinated phosphinodiboronate complex (Ca···C_cent_ 2.639 Å) and a more weakly interacting η^6^‐benzene derivative (Ca···C_cent_ 2.94 Å) of a monometallic and charge neutral calcium complex of the xanthene‐derived ligand used in the isolation of compounds **I**–**III** (Scheme 1c) [36, 37]. While the monomeric formulation of **1** may, therefore, be ascribed to the greater steric demands of the zincate *tert*‐butyl substituents in comparison the previously described analogues (Scheme 2), the resultant coordinative unsaturation at Ca1 necessitates its further coordination by a molecule of solvent.

**FIGURE 1:**
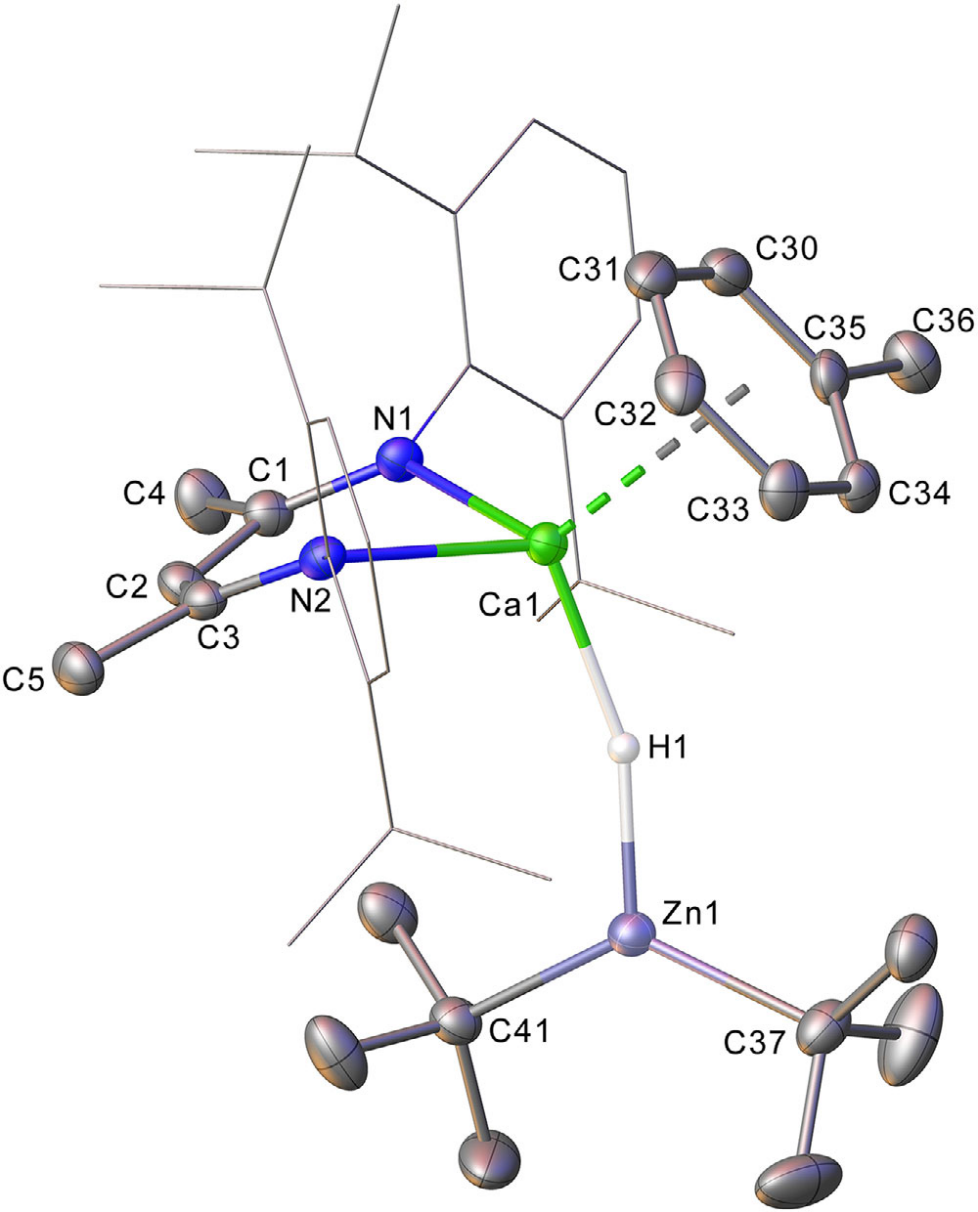
Molecular structure of compound **1** with displacement ellipsoids at 30%. For clarity, hydrogen atoms except H1, disordered atoms and an occluded but disordered molecule of toluene solvent are omitted. Similarly, Dipp substituents are displayed as wireframe. Selected bond lengths (Å) and angles (°): Ca1‐N1 2.3037(11), Ca1‐N2 2.3109(12), Zn1‐C37 2.0213(16), Zn1‐C41 2.0253(15), N1‐Ca1‐N2 80.14(4) and C37‐Zn1‐C41 129.27(7).

Unlike its less sterically encumbered variants (Scheme 2), compound **1** displayed no evidence for its further solution equilibration, either to the known [(BDI)Zn(*t*‐Bu)] or its unreported organocalcium analogue [38]. To investigate the inferred stabilising influence of the coordinated toluene, therefore, a *d*_6_‐benzene solution of compound **1** was treated with 2 molar equivalents of diethyl ether as a representative *O*‐donor base. A reaction was observed to proceed over the course of 16 h through the emergence of a single predominant β‐diketiminato‐containing product (**2**), which was most readily characterised by a singlet signal at *δ*_H_ 0.90 ppm that resonated with nine times the intensity of a newly appeared BDI γ‐methine environment (4.99 ppm). Notably, this process was accompanied by the deposition of a grey powder, assumed to be metallic zinc (Figure S1, *vide infra*). The solution was, thus, filtered and compound **2** was isolated in good yield (87%) by removal of all volatiles and crystallisation from hexane to provide colourless single crystals. The resultant x‐ray diffraction analysis identified compound **2** as the centrosymmetric calcium *tert*‐butoxide dimer illustrated in Figure 2.

**FIGURE 2:**
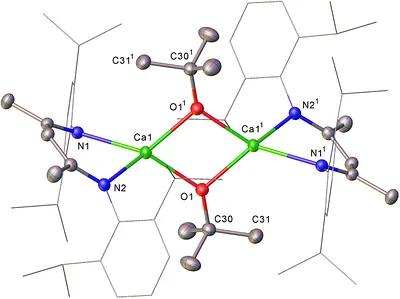
Molecular structure of compound **2** with displacement ellipsoids at 30%. For clarity, all hydrogen atoms are omitted. Similarly, Dipp substituents are displayed as wireframe. Selected bond lengths (Å) and angles (°): Ca1‐O1 2.2394(9), Ca1‐O1^1^ 2.2580(9), Ca1‐N1 2.4104(10), Ca1‐N2 2.3589(10), O1‐C30 1.4180(16), O1‐Ca1‐O1^1^ 80.27(3), O1‐Ca1‐N1 140.33(3), O1^1^‐Ca1‐N1 126.23(3), O1‐Ca1‐N2 114.85(3), O1^1^‐Ca1‐N2 117.90(3), N2‐Ca1‐N1 80.88(3) and Ca1‐O1‐Ca1^1^ 99.73(3). Symmetry operations to generate equivalent atoms: ^1^1‐*x*, 1‐*y*, 1‐*z*.

Although BDI‐supported calcium alkoxides are by no means common [39, 40, 41, 42, 43], the resultant metric data for **2** are unremarkable and consistent with these precedents. The reactivity of alkylzinc species with dioxygen and resultant alkoxide formation is well precedented [44, 45]. Our initial assumption that the oxygen atom in **2** most likely resulted from adventitious dioxygen was contradicted by subsequent duplication of the experiment. Several repetitions of the reaction invariably produced an identical outcome, indicating that the diethyl ether itself was the sole source of oxygen and encouraging more detailed analysis. Although monitoring of the reaction by ^1^H NMR spectroscopy at room temperature revealed the transient formation of a number β‐diketiminate environments, none of these species could be identified with any meaningful level of confidence. Careful scrutiny of the spectra presented by these reactions, however, did provide a tentative implication of 2‐methylpropane and H_2_ as likely reaction by‐products. Although the complexity of the spectra in the relevant regions did not allow for definitive identification of either compound, the presence of ethene was more clearly evidenced by the unimpaired observation of a singlet resonance at *δ*_H_ 5.26 ppm (Figure S6) [46, 47].

The generation of ethene *en route* to compound **2** was also supported by a further reaction of **IV** and Zn*t*‐Bu_2_ performed in the presence of Et_2_O but carried out in hexane rather than arene solvent. Although the major product of this reaction was again identified by NMR spectroscopy as **2**, the modified solvent conditions also induced the deposition of a small number of colourless single crystals of a new species (**3**) after storage of the reaction solution at −35°C for 72 h. The resultant x‐ray diffraction analysis identified **3** as a tri‐calcium complex that may be considered to comprise a molecule of **IV** in combination with the calcium zincate species, [(BDI)Ca(H_2_ZnCH_2_CH_2_C(CH_3_)_3_}] (Figure 3). While we caution against overinterpretation of this result, and we offer no rationale for the fate of the Et_2_O oxygen atom, the neopentyl group of the latter moiety may be rationalised to result from insertion of a molecule of ether‐derived ethene into a *tert*‐butyl *C‐*to‐zinc or *C*‐to‐calcium residue.

**FIGURE 3:**
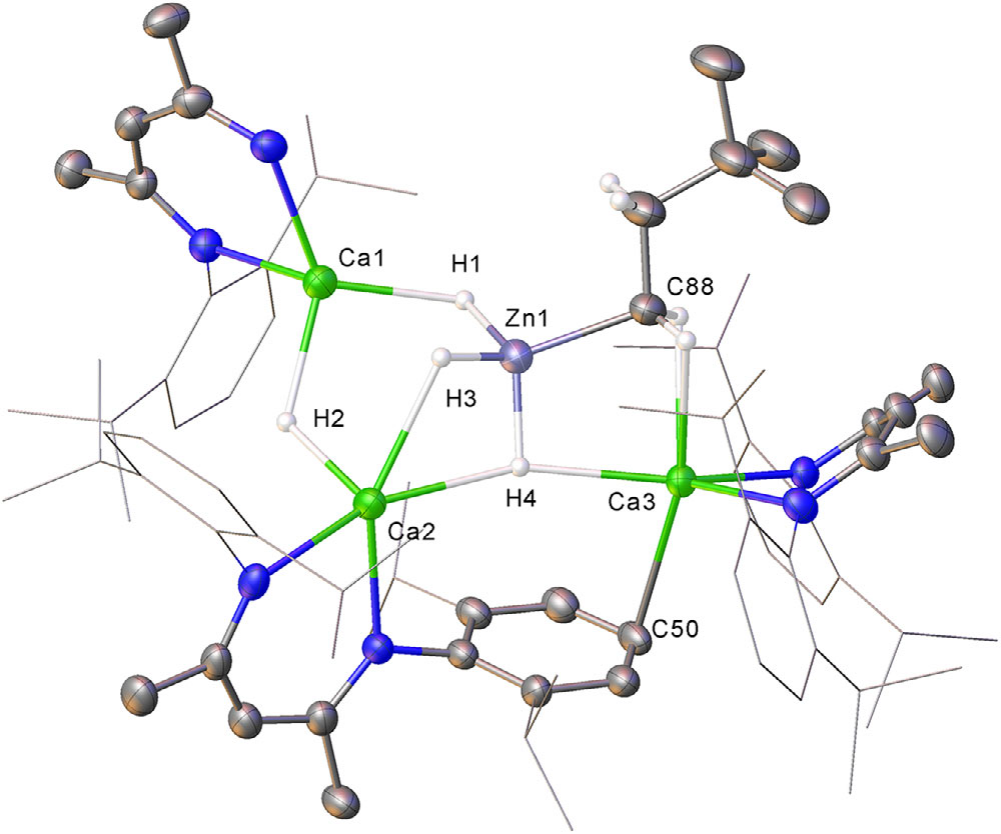
Molecular structure of compound **3** with displacement ellipsoids at 30%. For clarity, hydrogen atoms except H1–H4 and those attached to C88 and C89, and disordered atoms are omitted. Similarly, Dipp substituents are displayed as wireframe. Selected bond lengths (Å) and angles (°): Ca1‐N1 2.343(3), Ca1‐N2 2.367(3), Ca2‐N3 2.384(3), Ca2‐N4 2.345(3), Ca3‐N5 2.363(3), Ca3‐N6 2.358(3), Zn1‐C88 2.080(4), C88‐C89 1.522(6), N1‐Ca1‐N2 80.27(10), N4‐Ca2‐N3 78.17(11) and N6‐Ca3‐N5 80.88(10).

The mode of ethene generation was also investigated through a further repeat of the reaction performed with *d*_10_‐diethyl ether [48]. Although the resultant ^1^H NMR spectrum bore a close resemblance to the data provided by its protio analogue, the ethene singlet resonance was now notably absent. More informative, therefore, was the corresponding ^2^H NMR spectrum, which readily identified D_2_C═CD_2_ through the observation of a signal at *δ* 5.26 ppm alongside a further lower intensity resonance at *δ* 1.62 ppm. This latter signal presented as a binomial pentet (^3^ *J*_HD_ = 1.1 Hz) and was, thus, assigned as the unique deuterium nucleus of 2‐methylpropane‐2*d* (Figures S15–S18) [47]. Although conclusive evidence for the generation of HD could not obtained from this spectrum, the identification of 2‐methylpropane‐2*d* was confirmed by the observation of a molecular ion at *m*/*z* 59.1 obtained by GC–MS analysis of the headspace of the NMR tube (Figures S30 and S31).

While these data provided strong corroborative evidence that ethene had resulted from deprotonative degradation of Et_2_O, a definitive demonstration that the oxide component of **2** arises from the same fragmentation process required the preparation of an ^17^O‐enriched ether variant. Et_2_^17^O was, thus, prepared by a modification of the process reported by Marković et al. for the synthesis of similarly labelled THF, in this case by deprotonation of (20%–24.9%) H_2_^17^O and reaction with iodoethane [49]. Treatment of **1** with this reagent provided a ^1^H NMR spectrum that was identical to its unlabelled analogue. Although attempted analysis by ^17^O NMR spectroscopy was inconclusive, hydrolysis of the resultant sample of **2** and further GC‐MS analysis provided a molecular ion for (CH_3_)_3_C^17^OH at *m*/*z* 75.1, which was clearly observed in the correct isotopic ratio alongside its unlabelled ^16^O counterpart at *m*/*z* 74.1 (Figures S32 and S33).

While continuing to exercise a degree of caution, we suggest that the stoichiometry presented in Scheme 3 provides a realistic representation of the overall mass balance associated with the exclusive transformation of **1** to **2**. Any mechanistic hypothesis leading to **2**, therefore, must account for both the stoichiometry of the by‐products and the, at first sight counterintuitive, deduction that its *tert*‐butoxy group arises from C─O bond formation between a negatively charged oxide and a formal *tert*‐butyl carbanion. To this end, therefore, the energy profile of the reaction of **1** with Et_2_O was investigated computationally. Initial displacement of toluene from **1** and formation of its Et_2_O‐adducted analogue (**A**, Figure 4) was found to be marginally exergonic (Δ*G =* −2.4 kcal mol^−1^). Although transition states could be located for the deprotonation at either the α‐ or β‐C─H position of the calcium‐coordinated ether by an intact (i.e., zinc‐bound) zincate *tert*‐butyl residue, the resultant barriers (>35 kcal mol^−1^, see Figure S40) were inconsistent with the facility of the transformation at room temperature. Mindful of the implied extreme basicity displayed by our previous attempts to synthesise BDI‐supported calcium *sec*‐alkyls [23], an alternative pathway invoking zinc‐to‐calcium transfer of a zincate *tert*‐butyl substituent was explored. The generation of the terminally‐coordinated *tert*‐butylcalcium species (**B,** Figure 4), in which the resultant organozinc hydride retains the Ca‐μ‐H─Zn bridging interaction, was, thus, identified to be kinetically viable via **TS(A‐B)** (Δ*G* ^‡^ = 24.0 kcal mol^−1^). Deprotonation of a β‐C─H bond of the coordinated ether by the putative calcium alkyl unit of **B** was then found to be quite facile via **TS(B‐C)** (Δ*G* ^‡^ = 14.8 kcal mol^−1^) to yield the diorgano(hydrido)zincate anion of **C**. Although this anion maintains contact with calcium through a combination of Ca‐μ‐H─Zn bridging and coordination of its pendent ether residue, **C** is evidently short‐lived [51]. Intermediate **C** undergoes an effectively barrierless reaction sequence, **TS(D‐E)** (Δ*G* ^‡^ = 5.7 kcal mol^−1^), to effect ethene extrusion to yield the ethoxy(hydrido)zincate species (**G**, Figure 5). Although **G** continues to display a similar bidentate μ‐H‐κ^1^‐O mode of engagement with calcium, the consequent potential for further direct ethoxide deprotonation by the bridging hydride from **G** was found to be highly geometrically constrained and, thus, energetically inaccessible in **TS(G‐V)** (Δ*G* ^‡^ = 48.4 kcal mol^−1^); see Figure S43.

**FIGURE 4:**
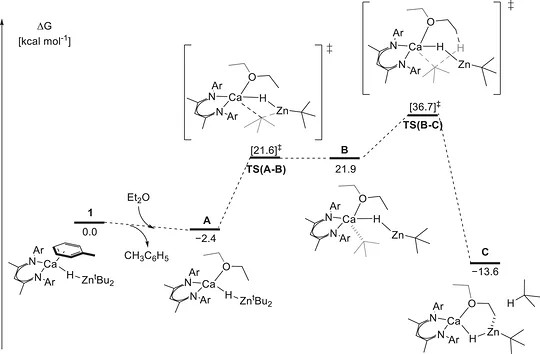
Computed reaction profile (BP86‐D3^BJ^(SMD = C_7_H_8_)/def2‐TZVPP//BP86/def2‐SVP in kcal mol^−1^) for Et_2_O coordination to **1** and the generation of **C** by ether β‐C─H alkyl deprotonation [50].

**FIGURE 5:**
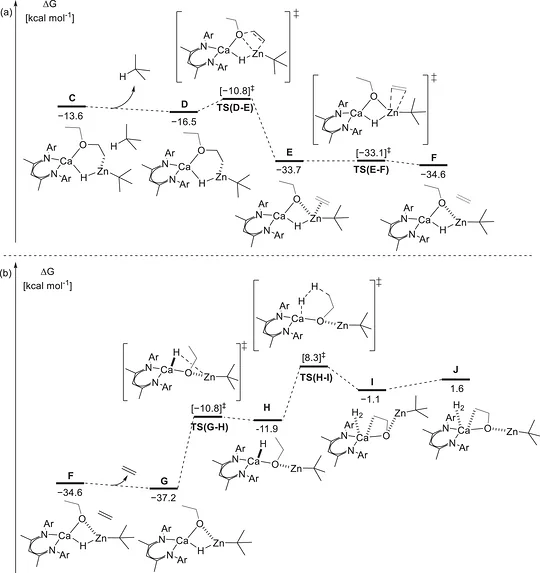
Computed reaction profiles (BP86‐D3^BJ^(SMD = C_7_H_8_)/def2‐TZVPP//BP86/def2‐SVP in kcal mol^−1^) for (a) release of (CH_3_)_3_CH and ethene extrusion with subsequent ethene decoordination from Zn to provide **F**; (b) Zn─H bond cleavage with subsequent ethoxide β‐C─H deprotonation by terminal Ca hydride intermediate **H** resulting in dihydrogen–calcium intermediate **J** [52].

Although several profiles for ethene extrusion invoking hydride detachment from calcium were also evaluated, the most energetically viable pathway leading to the ultimate formation of **2** is summarised in Figures 5b and 6. Ethoxide β‐C─H deprotonation, thus, requires rupture of the Zn─H bond of **G** via **TS(G‐H**) (Δ*G* ^‡^ = 20.2 kcal mol^−1^), whereupon the terminal calcium hydride **H** initiates a mildly exergonic sequence of H_2_ elimination and ethene extrusion events via intermediates **I** to **N** (Δ*G* **_(H_**_→_ **_N_**_)_ = −14.3 kcal mol^−1^).

**FIGURE 6:**
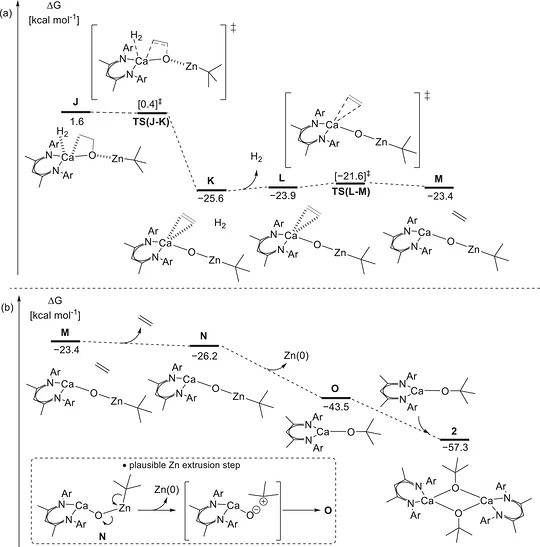
Computed reaction profiles (BP86‐D3^BJ^(SMD = C_7_H_8_)/def2‐TZVPP//BP86/def2‐SVP in kcal mol^−1^) for (a) dihydrogen release and ethene extrusion resulting in the formation of intermediate **M**; (b) Zn extrusion from **N** and dimerisation of intermediate **O** resulting in **2**.

While the molecular Ca‐μ‐O─Zn bridged species (**N**) is thermodynamically feasible in relation to **A** (Δ*G* = −23.8 kcal mol^−1^), it is evidently unstable with respect to elimination of Zn(0). A demonstration of the viability of this latter process required recourse to our recently introduced protocol to estimate the energetic impact of bulk metal formation during solvent phase molecular transformations [53, 54, 55, 56]. Construction of a hybrid Hess’ law cycle using the calculated energies of the putative species in conjunction with the thermochemical free energy of atomisation for zinc (see Supporting Information) [57], thus, supported the viability of the transformation of **N** to **O** (Δ*G* = −43.5 kcal mol^−1^). While not addressed explicitly in these calculations, we also suggest that the formation of Zn(0) may be considered as a process of reductive elimination underpinned by formal two electron oxidation of the *tert*‐butyl carbanion of **N** and a C─O bond forming step between a calcium‐bound oxide and the resultant classical tertiary carbenium ion (see insert, Figure 6b) [58, 59, 60]. Although it is not possible to provide confident insight into the precise chronology of events leading to the final dimeric alkoxide structure, this process of Zn(0) elimination ensures that the overall transformation of **1** to **2** is significantly exergonic (Δ*G* = −57.3 kcal mol^−1^).

This theoretical interpretation of the reaction of **1** and Et_2_O suggests that the various molecular steps of β‐C─H deprotonation, alkene extrusion, zinc reduction and tertiary carbenium ion formation prior to C─O bond formation may be equally applicable to other homologues of diethyl ether. Accordingly, monitoring of analogous reactions between **1** and di‐*iso*‐propyl, di‐*n*‐butyl and di‐*n*‐hexyl ether by ^1^H NMR spectroscopy, in each case indicated that the similar formation of compound **2** had occurred with respective conversions of 96%, 79% and 91% alongside the generation of the respective 1‐butene, 1‐hexene and propene by‐products (Figures S24–S26).

In conclusion, the combination of highly reactive organocalcium and more noble organozinc chemistries enables a process of twofold di‐*n*‐alkyl ether activation and Zn(II)/Zn(0) redox‐based C─O bond formation. We are continuing to study and elaborate the potential generality of this counterintuitive but highly selective mode of bimetallic cooperation.

## Author Contributions

**Kyle G. Pearce**: investigation, methodology, writing – review and editing, conceptualization, formal analysis. **Agustín Morales**: conceptualization, investigation, writing – review and editing, methodology, formal analysis. **Michael S. Hill**: conceptualization, investigation, funding acquisition, writing – original draft, methodology, project administration, supervision. **Claire L. McMullin**: conceptualization, investigation, methodology, visualization, writing – review and editing, project administration, supervision.

## Conflicts of Interest

The authors declare no conflicts of interest.

## Supporting information



Full experimental and instrumental details, NMR spectra, details of the x‐ray analysis of compounds **1**–**3** [61], and the methods employed in the quantum chemical investigations of this chemistry are available in the Supporting Information to this article.
**Supporting File**: anie74587‐sup‐0001‐SuppMat.pdf.

## Data Availability

The data that support the findings of this study are available in the Supporting Information of this article.
