# Supplementary material for: Dialkyl Ethers and Sacrificial Zinc: Oxide Synthons for Calcium tert‐Butoxide Formation
Source: Angew Chem Int Ed Engl. 2026 Sep 8;65(43):e9268230. doi: 10.1002/anie.9268230 (PMC13676388; doi:10.1002/anie.9268230)
Supplement: Supplementary file 1 [file ANIE-65-e9268230-s001.pdf]

Synthetic Details

General Considerations

All manipulations were carried out using standard Schlenk line and glovebox techniques under an inert atmosphere of argon. NMR experiments were conducted in J-Young's NMR tubes and prepared in a glovebox. NMR spectra were recorded on a Bruker BioSpin GmbH spectrometer operating at 400.13 MHz (^{1}H), 61.42 MHz (^{2}H), 100.62 MHz (^{13}C) and 54.26 MHz (^{17}O). Elemental analyses were performed at Elemental Microanalysis Ltd., Okehampton, Devon, UK or by the Elemental Analysis Services Team at London Metropolitan University. GC-MS was performed with a 8890 gas chromatography system coupled with 5977B MSD used for the analysis. Split injections of 1 µL were performed, with a split ratio of 10:1 (split flow of 10 mL/min), using a split inlet liner, with tapered focus and deactivated glass wool (Agilent 210-4022-5). The inlet was heated to 280 °C with a 3 mL/min septum purge flow. An Agilent HP-5MS (30 m, 0.25mm, 0.25µm) column was used with He (BOC, N5.5) as the carrier gas, at a constant flow of 1.0 mL/min. The column oven gradient was started at 70 °C, held for 4 minutes, then ramped at 15 °C/min to 280 °C, with a 5 minute hold. The MSD transfer line was set at 280 °C, MSD source at 230 °C, and the MSD quad temperature was set to 150 °C. After an initial solvent delay of 7 minutes the MSD detection was performed using full scan mode, over the range of 180 – 550 *m*/*z*, with a scan speed of 1562 µs, and a gain factor of 15. For headspace analysis, a GilAir Plus Sampling Pump was used to load an offline collection tube for subsequent analysis. Data analysis was performed in the Agilent Qualitative Analysis v.10.0 and used the NIST 17 library to identify and confirm compounds through spectral matching. Solvents were dried by passage through a commercially available solvent purification system and stored under argon in ampoules over 4 Å molecular sieves. C_6D_6, C_7D_8 and $C_4D_{10}O$ were purchased from Merck, dried over potassium, distilled and stored over molecular sieves. ^{17}O-Water (20-24.9 atom % ^{17}O) and iodoethane were purchased from Merck and used as purchased. ^{i}PrO^{i}Pr, ^{n}BuO^{n}Bu and ^{n}HexO^{n}Hex were purchased from Merck, dried over $[CaH_2]_n$, distilled and stored over 4 Å molecular sieves prior to use. $[(BDI)CaH]_2$ (**IV**, BDI = HC{(Me)CN-2,6-*i*-$Pr_2C_6H_3$}$_2$),[1] and Zn^{t}Bu$_2$ were synthesised according to literature procedures.[2]

Synthesis of [(BDI)Ca(Tol)(µ-H)Zn^{t}Bu$_2$] (1)

Zn^{t}Bu$_2$ (28.6 mg, 0.16 mmol) was added to a pre-weighed vial containing $[(BDI)CaH]_2$ (**IV**, 73.2 mg, 0.08 mmol) dissolved in d_8-toluene (*ca.* 1 cm^3) resulting in a very pale-yellow colouration. The reaction mixture was placed in the freezer (–35 °C), affording colourless crystals after 16 hours. The supernatant was decanted, and the crystals were washed with cold hexane (–35 °C; 2 × 1 cm^3) before being dried *in vacuo*. Yield: 48.3 mg, 83%. ^{1}H NMR (C_7D_8): δ = 7.10-7.03 (Ar, 6H), 4.77 (s, HCCN, 1H), 4.05 (s, Ca–µ$_2$–H–Zn, 1H), 3.08 (hept, HC(CH$_3$)$_2$, $^3J_{HH}$ = 6.72 Hz, 4H), 1.61 (s, Zn^{t}Bu$_2$, 18H), 1.55 (s, NCCH$_3$, 6H), 1.22 (d, HC(CH$_3$)$_2$, $^3J_{HH}$ = 6.72 Hz, 12 H), 1.15 (d, HC(CH$_3$)$_2$, $^3J_{HH}$ = 6.72 Hz, 12 H). ^{13}C{^{1}H} NMR (C_7D_8): δ = 166.6 (NCCH$_3$), 145.5 (*i*-Ar-C), 141.7 (Ar-C), 123.9 (*p*-Ar-C), 94.8 (HCCN), 34.9

(C(C$\underline{H}_3$)$_3$), 28.1 (H$\underline{C}$(CH$_3$)$_2$), 24.7 (HC($\underline{C}$H$_3$)$_2$), 23.9 (NC$\underline{C}$H$_3$), 23.55 (Zn-$\underline{C}$(CH$_3$)$_3$). Anal. Calc. for $C_{44}H_{68}N_2Zn_1Ca_1$: C, 72.34; H, 9.38; N, 3.87. Found: C, 72.55; H, 9.32; N, 3.69.

Synthesis of [(BDI)CaO^{t}Bu]$_2$ (2)

Et_2O (5.6 µl, 0.056 mmol) was added to a benzene solution of [(BDI)Ca(Tol)(µ-H)Zn^{t}Bu$_2$] (**1**, 20 mg, 0.027 mmol), observing ethene formation immediately (Figure S6). The sample was left to sit for 16 hours, observing the deposition of metallic powder, believed to be Zn metal. The solution was filtered away from the powder and all volatiles were removed *in vacuo*, affording [(BDI)CaO^{t}Bu]$_2$. Yield: 12.5 mg, 87%. ^{1}H NMR (C_7D_8): δ = 7.12 (s, Ar-H, 6H), 4.99 (s, $\underline{H}$CCN, 1H), 3.19 (hept, $\underline{H}$C(CH$_3$)$_2$, $^3J_{HH}$ = 6.89 Hz, 4H), 1.72 (s, NCC$\underline{H}_3$, 6H), 1.31 (d, HC(C$\underline{H}_3$)$_2$, $^3J_{HH}$ = 6.87 Hz, 12 H), 1.13 (d, HC(C$\underline{H}_3$)$_2$, $^3J_{HH}$ = 6.87 Hz, 12 H), 0.90 (s, C(CH$_3$)$_3$, 9H). ^{13}C{^{1}H} NMR (C_7D_8): δ = 167.6 (N$\underline{C}$CH$_3$), 141.5 ((*i*-Ar-C), 126.0 (Ar-C), 124.0 (Ar-C), 95.4 (H$\underline{C}$CN), 33.7 (C($\underline{C}$H$_3$)$_3$), 28.9 (H$\underline{C}$(CH$_3$)$_2$), 24.0 (HC($\underline{C}$H$_3$)$_2$), 23.9 (HC($\underline{C}$H$_3$)$_2$), 23.5 (NC$\underline{C}$H$_3$), 22.6 (C(CH$_3$)$_3$, observed by HMBC).

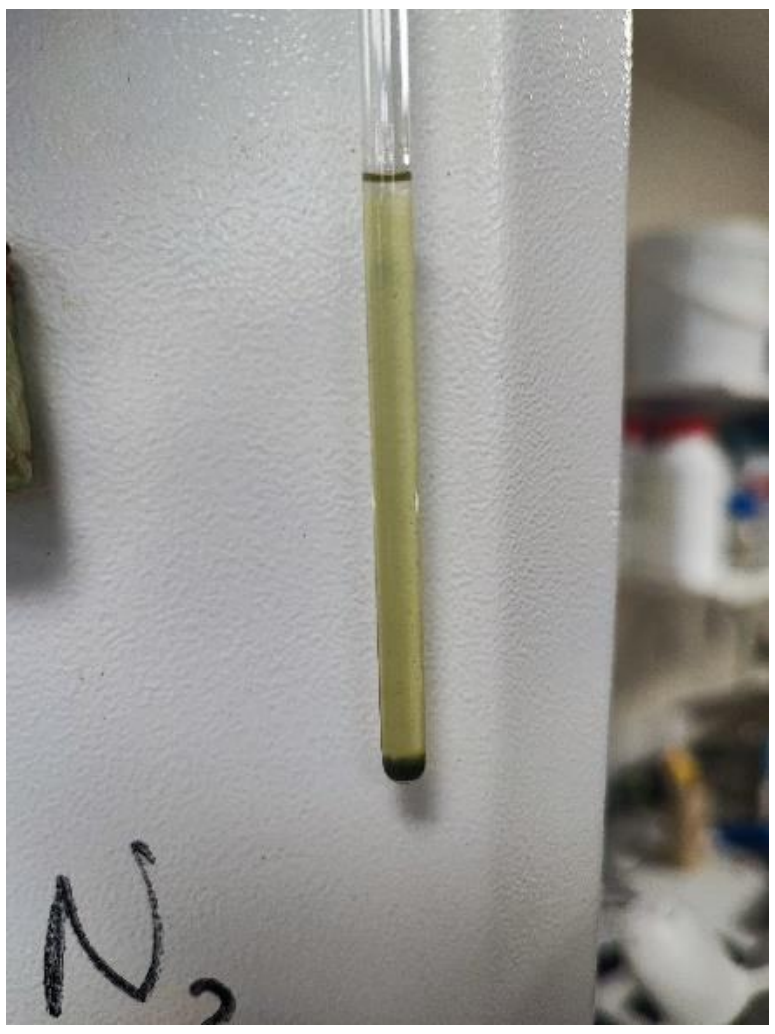

Figure S1. Deposition of metallic zinc coincident with the formation of 1.

Isolation of [{(BDI)Ca(µ-H)$_2$}$_2$Zn(µ-H)µ-CH$_2$CH$_2$C(CH$_3$)$_3$Ca(BDI)] (3)

Zn^{t}Bu$_2$ (20.04 mg, 0.11 mmol) was added to a pre-weighed vial containing [(BDI)CaH]$_2$ (**IV**, 52.2 mg, 0.056 mmol) dissolved in hexane (*ca.* 1 cm^3) in the presence of diethyl ether, resulting in a deep yellow colouration. The reaction mixture was placed in the freezer (–35 °C), affording a few colourless crystals after 72 hours.

Synthesis of ^{17}O-labelled Diethyl Ether

This procedure was based upon the literature procedure to prepare ^{17}O-enriched THF,[3] and was first trialled with unlabelled water.

^{17}O-labelled water (20-24.9% ^{17}O) (1.01 g, 56.08 mmol) was added to an ampoule containing NaOH (0.832 g, 20.80 mmol). $^{n}Bu_4NOH$ (0.676 g, 2.61 mmol) and iodoethane (1.616 g, 10.36 mmol) were added, before sealing the ampoule and heating to 130 °C for 48 hours. After allowing the solution to cool to ambient temperature, the solution was vacuum transferred into a fresh ampoule, diluted with benzene (2 cm^3) and molecular sieves were added before heating the mixture at 40 °C for 4 hours. After cooling to ambient temperature, the solution was filtered into an ampoule containing $[CaH_2]_n$ and heated at 70 °C for 16 hours whilst stirring. After 16 hours the solution was allowed to cool to ambient temperature and vacuum transferred to a pre-weighed ampoule, affording (20-24.9%) ^{17}O-diethyl ether. Yield: 162 mg, 21%. ^1H NMR (C_7D_8): δ = 3.26 (q, $OC\underline{H}_2CH_3$, $^3J_{HH}$ = 7.05 Hz, 4H), 1.11 (t, $OCH_2C\underline{H}_3$, $^3J_{HH}$ = 7.05 Hz, 6H). ^{17}O NMR (C_7D_8): δ = 17.72 (s).

Reaction of [(BDI)Ca(Tol)(μ-H)Zn^{t}Bu$_2$] with *d*$_{10}$-Diethyl Ether

d_{10}-Et_2O (3.78 μl, 0.063 mmol) was added to a d_8-toluene solution of [(BDI)Ca(Tol)(μ-H)Zn^{t}Bu$_2$] (**1**, 23 mg, 0.031 mmol) and the formation of [(BDI)CaO^{t}Bu]$_2$ (**2**) was observed by ^1H NMR spectroscopy (Figure S), as well as the evolution of deuterated-ethene and the deuterated methine resonance for 2-methylpropane-2*d* (C_4H_9D) in the corresponding ^2H NMR spectrum (Figure S14). No evidence for H_2 or HD could be observed spectroscopically. The deposition of metallic powder, believed to be Zn metal was also noted. The closest literature precedent for 2-methylpropane-2*d* (C_4H_9D) for which there is a reported ^2H NMR spectrum is propane-2*d* (C_3H_7D), observed at 1.19 ppm.[4]

Reaction of [(BDI)Ca(Tol)(μ-H)Zn^{t}Bu$_2$] with ^{17}O-Diethyl Ether

^{17}O-diethyl ether (20-24.9% ^{17}O) was added to a J-Young's tube containing [(BDI)Ca(Tol)(μ-H)Zn^{t}Bu$_2$] (**1**, 5 mg, 0.07 mmol) dissolved in C_6D_6 (0.6 cm^3) and monitored by ^1H and ^{17}O NMR spectroscopy. The formation of [(BDI)CaO^{t}Bu]$_2$ (**2**) was readily observed in the ^1H NMR spectrum. In the corresponding ^{17}O NMR spectrum, however, only ^{17}O-diethyl ether was observed (Figure S21), even after 380,000 scans. The absence of a resonance associated with [(BDI)Ca^{17}O^{t}Bu]$_2$ is postulated to be a consequence of the high degree of asymmetry at oxygen.

General Reaction Procedure for the Activation of Ethers

The selected ether (^{i}Pr, ^{n}Bu, ^{n}Hex; 0.027 mmol) was added to a sample of [(BDI)Ca(Tol)(μ-H)Zn^{t}Bu$_2$] (**1**, 10 mg, 0.014 mmol) dissolved in d_8-toluene (0.6 cm^3). The reaction was left to sit for 16 hours before spectroscopic analysis. In each case, [(BDI)CaO^{t}Bu]$_2$ (**2**), was formed alongside the corresponding alkene, which was identified by the characteristic vinylic resonance, consistent with literature values for propylene,[6] butene[6] and hexene.[7]

NMR Spectra

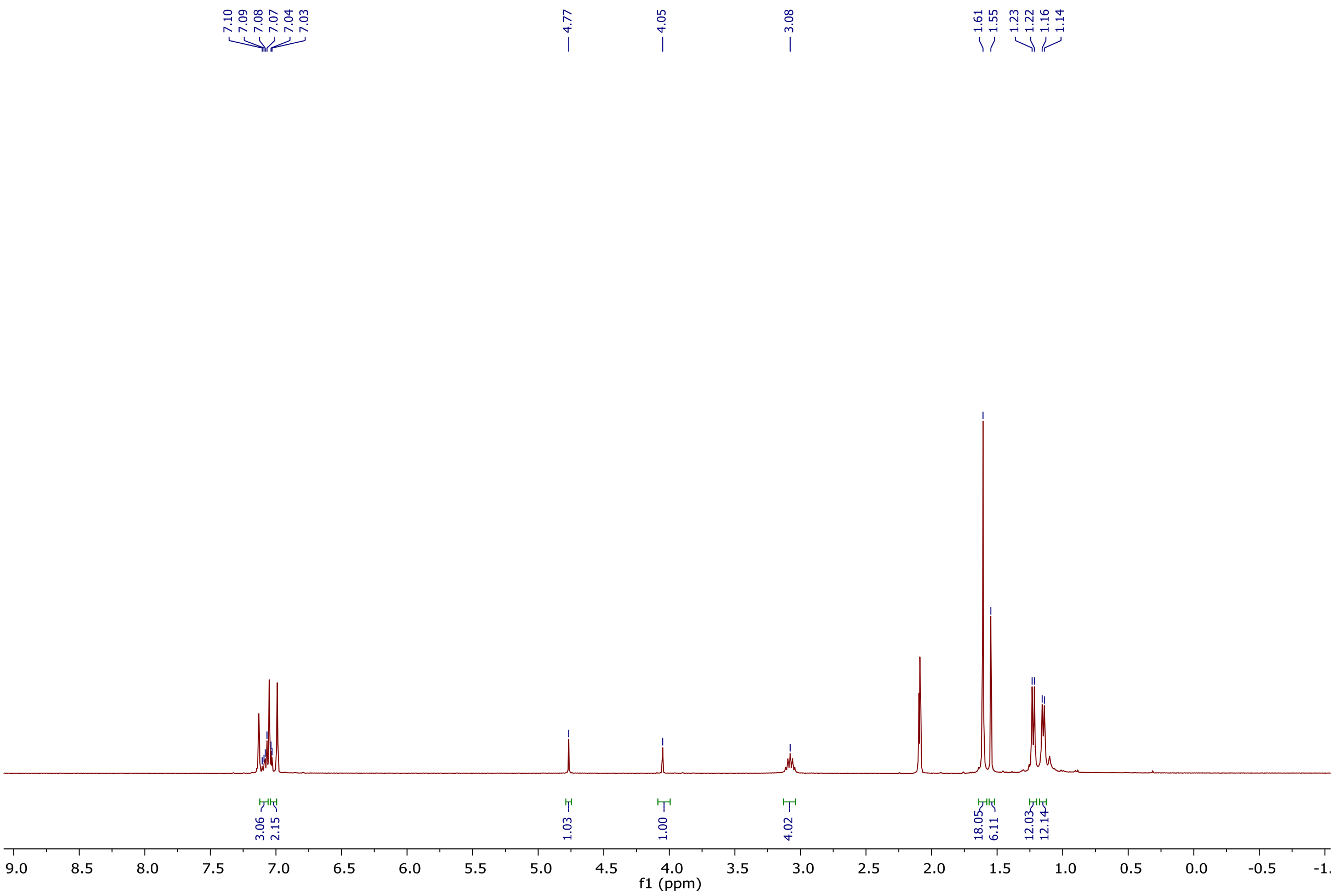

Figure S2. *¹H NMR Spectrum (C_7D_8, 253 K, 400.13 MHz) for* ***[(BDI)Ca(Tol)(μ-H)Zn^{t}Bu$_2$] (1)***.

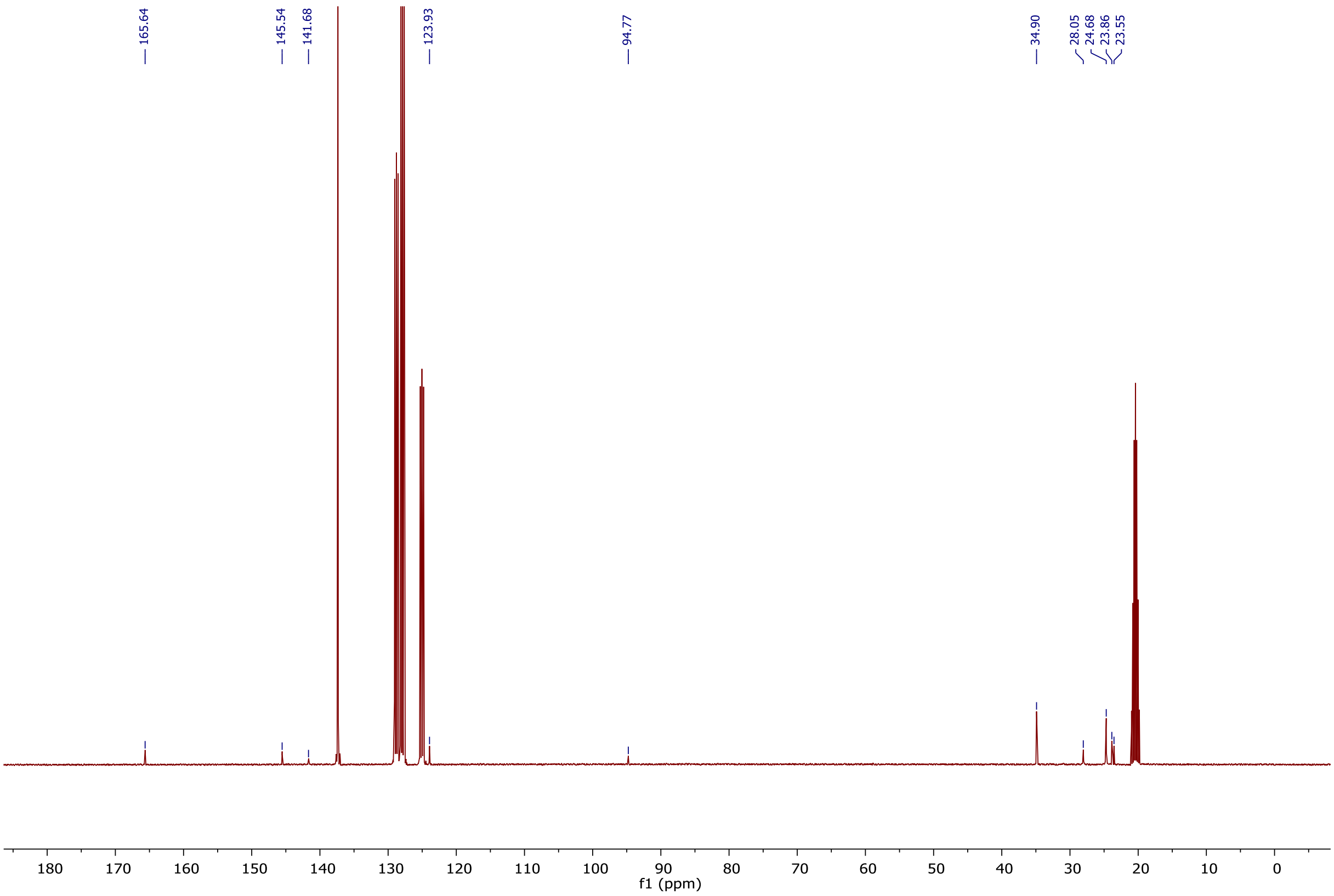

Figure S3. *$^{13}C\{^1H\}$ NMR Spectrum (C_7D_8, 253 K, 100.62 MHz) for* ***[(BDI)Ca(Tol)(μ-H)Zn^{t}Bu$_2$] (1)***.

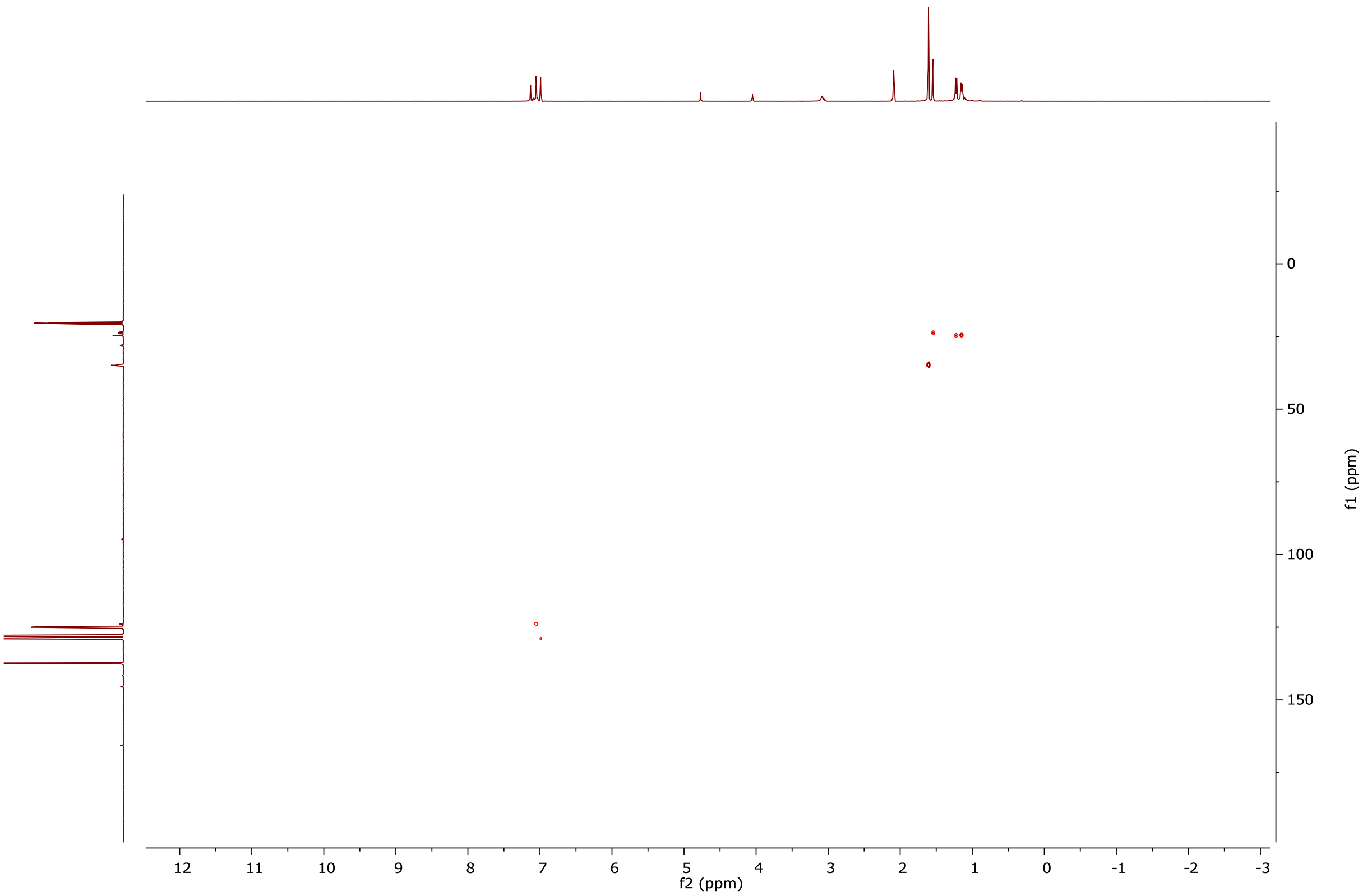

Figure S4. *^{1}H-^{13}C HSQC trace (C_7D_8, 253 K, 400.13, 100.62 MHz) for* ***[(BDI)Ca(Tol)(μ-H)Zn^{t}Bu$_2$] (1)***.

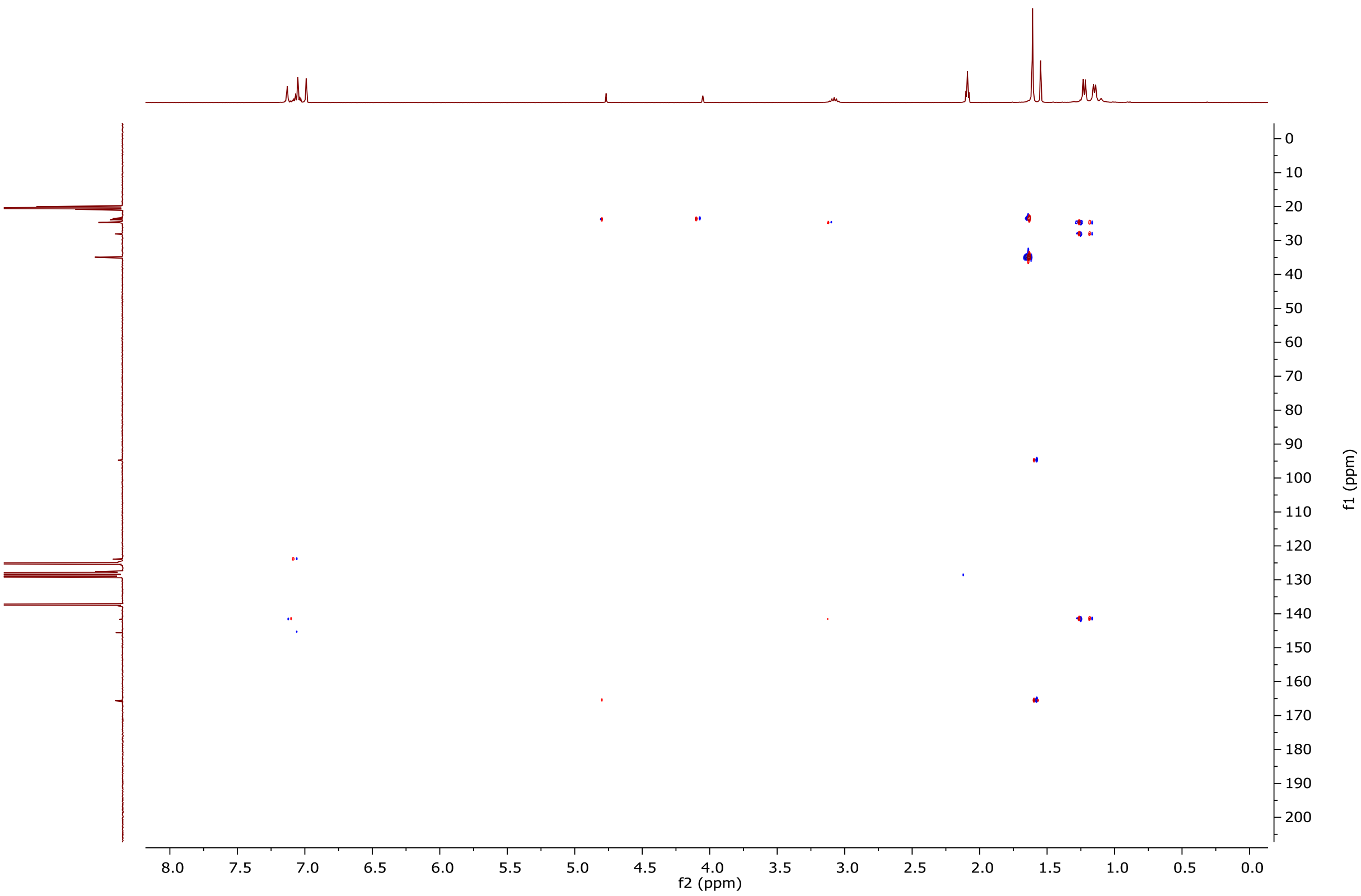

Figure S5. *^{1}H-^{13}C HMBC trace (C_7D_8, 253 K, 400.13, 100.62 MHz) for* ***[(BDI)Ca(Tol)(μ-H)Zn^{t}Bu$_2$] (1)***.

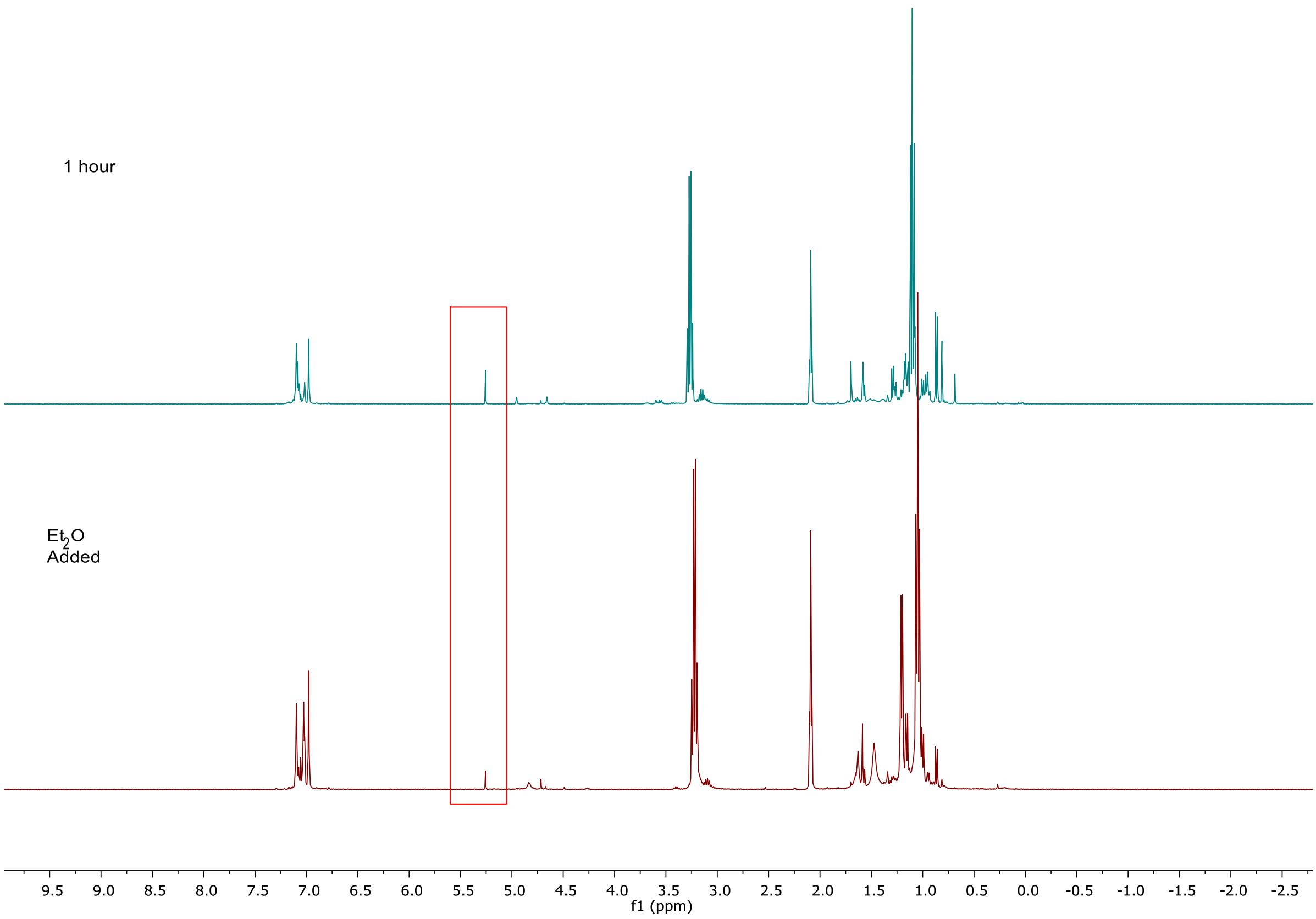

Figure S6. *^{1}H NMR Spectra (C_7D_8, 253 K, 400.13 MHz) for [(BDI)Ca(Tol)(μ-H)Zn^{t}Bu$_2$] (**1**) + Et_2O. The red rectangle highlights ethene formation.*

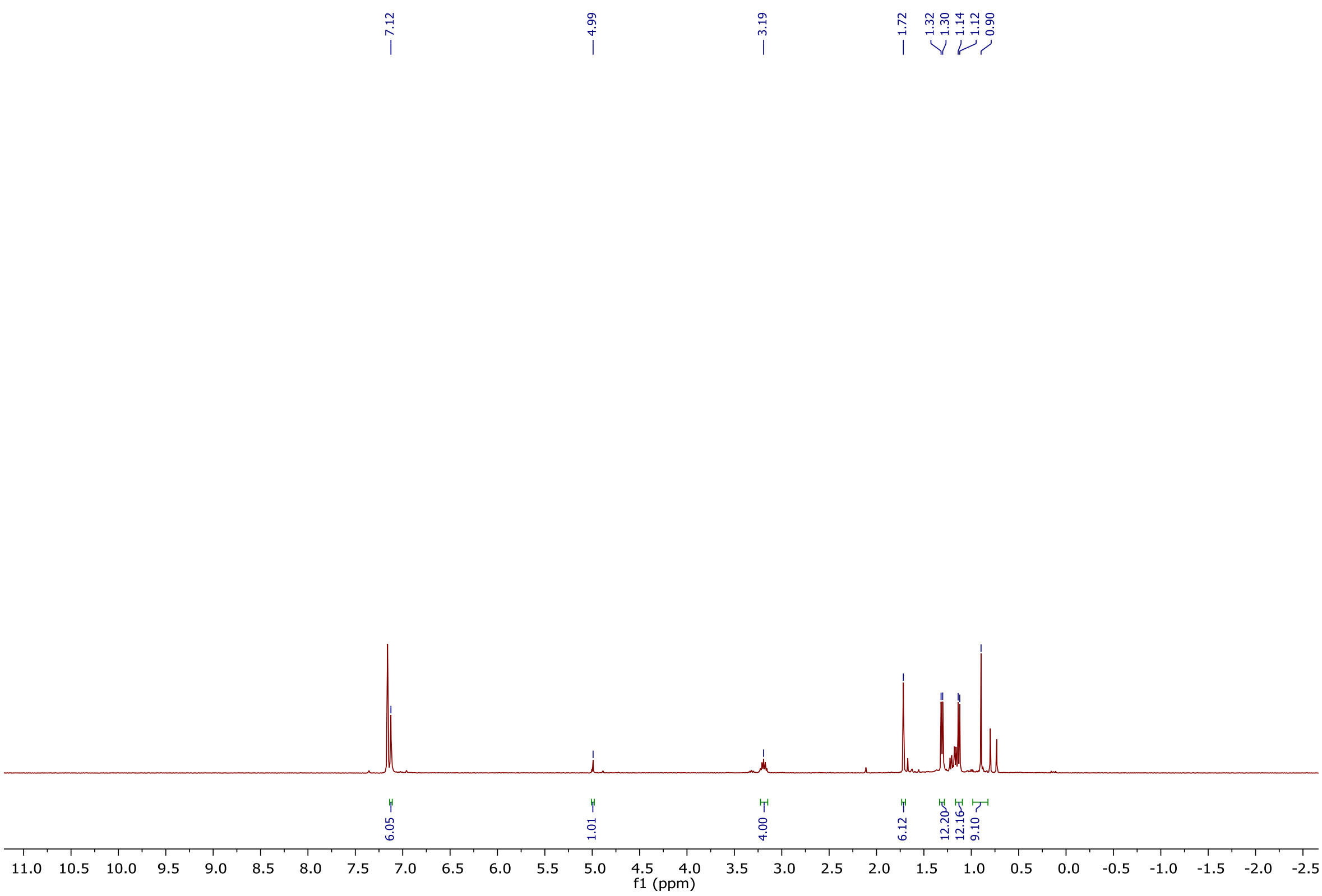

Figure S7. *^{1}H NMR Spectrum (C_6D_6, 298 K, 400.13 MHz) for **[(BDI)CaO^{t}Bu]$_2$ (2)**.*

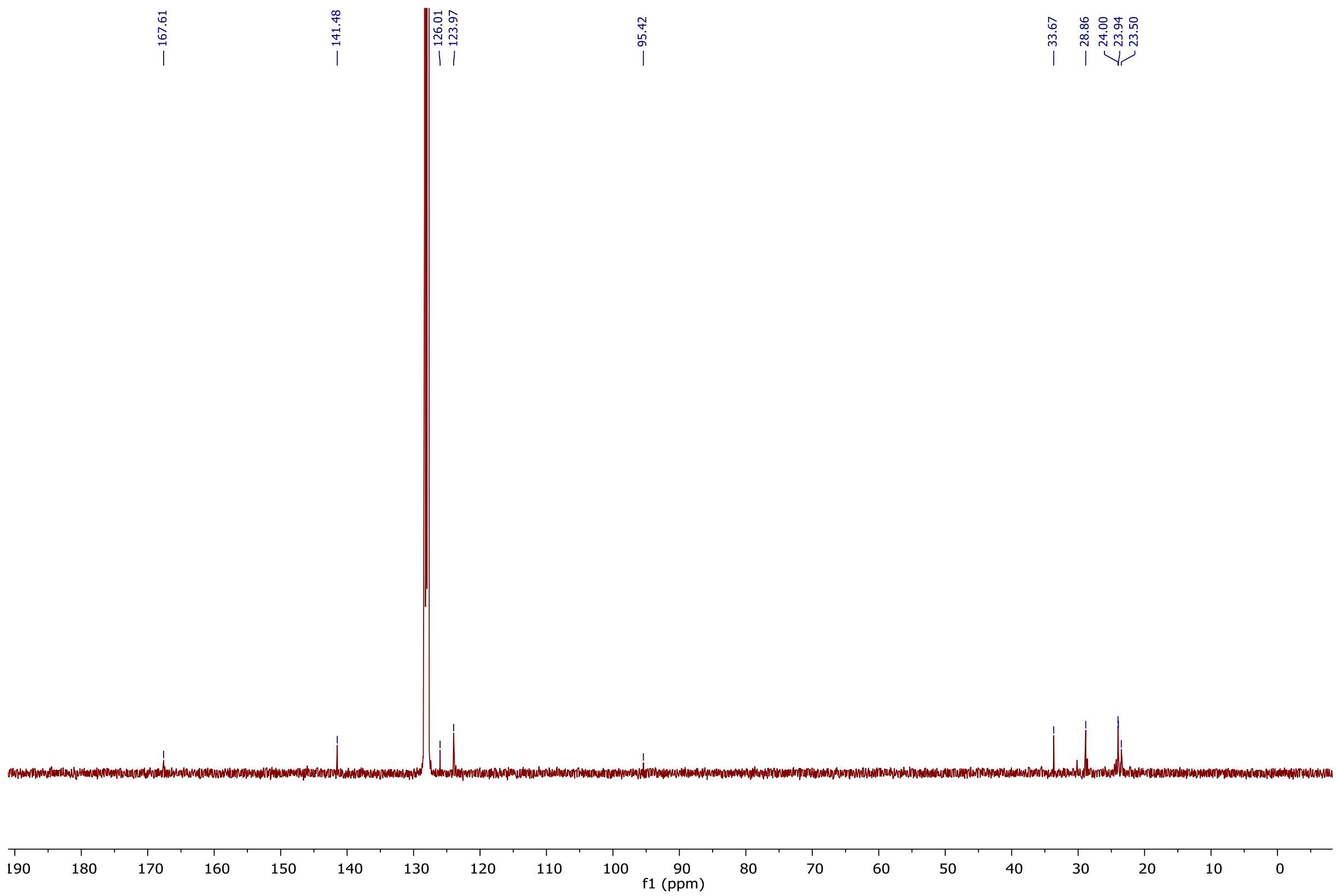

Figure S8. *$^{13}C\{^{1}H\}$ NMR Spectrum (C_6D_6, 298 K, 100.62 MHz) for* ***[(BDI)CaO^{t}Bu]$_2$ (2)****.*

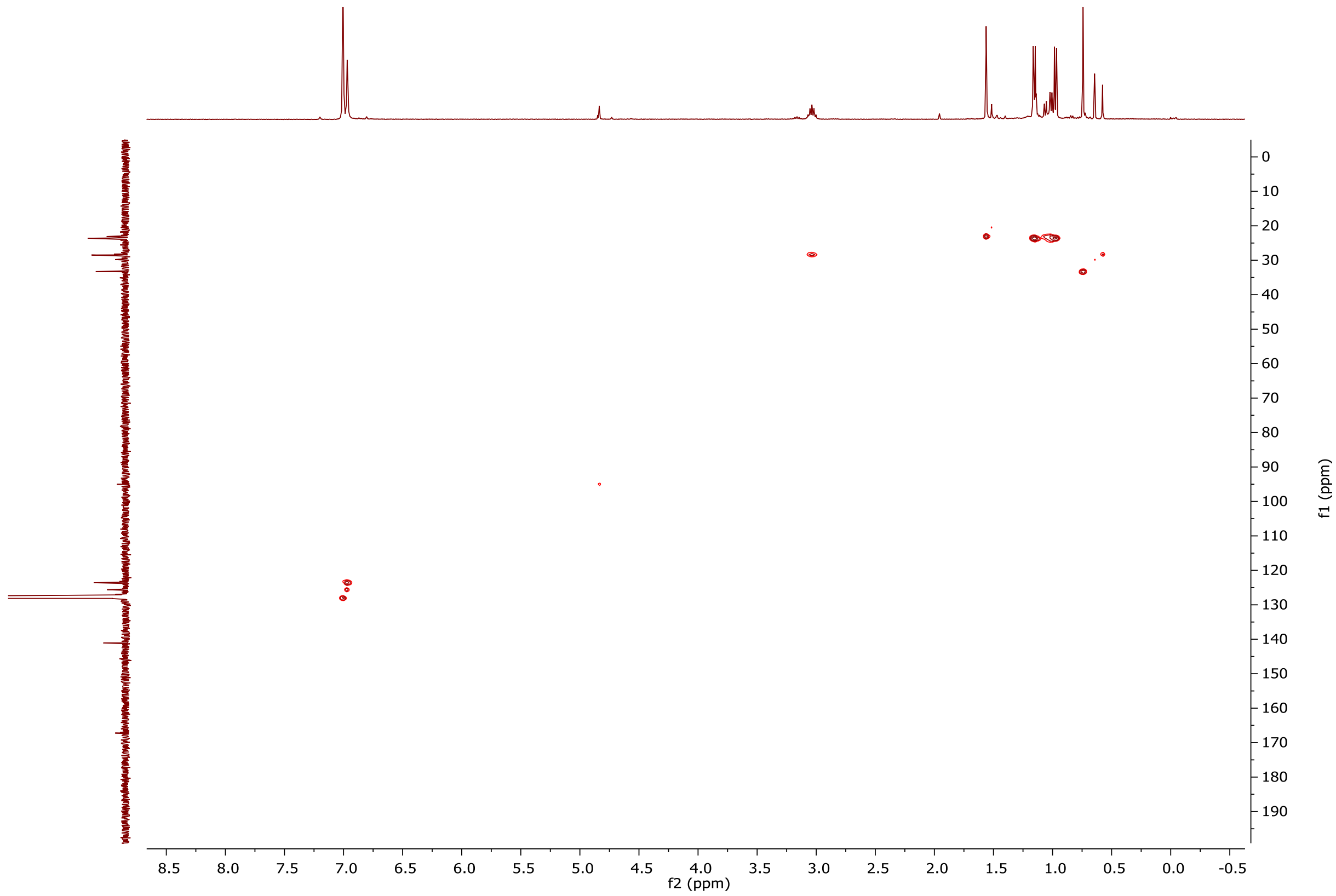

Figure S9. *^{1}H-^{13}C HSQC trace (C_7D_8, 298 K, 400.13, 100.62 MHz) for* ***[(BDI)CaO^{t}Bu]$_2$ (2)****.*

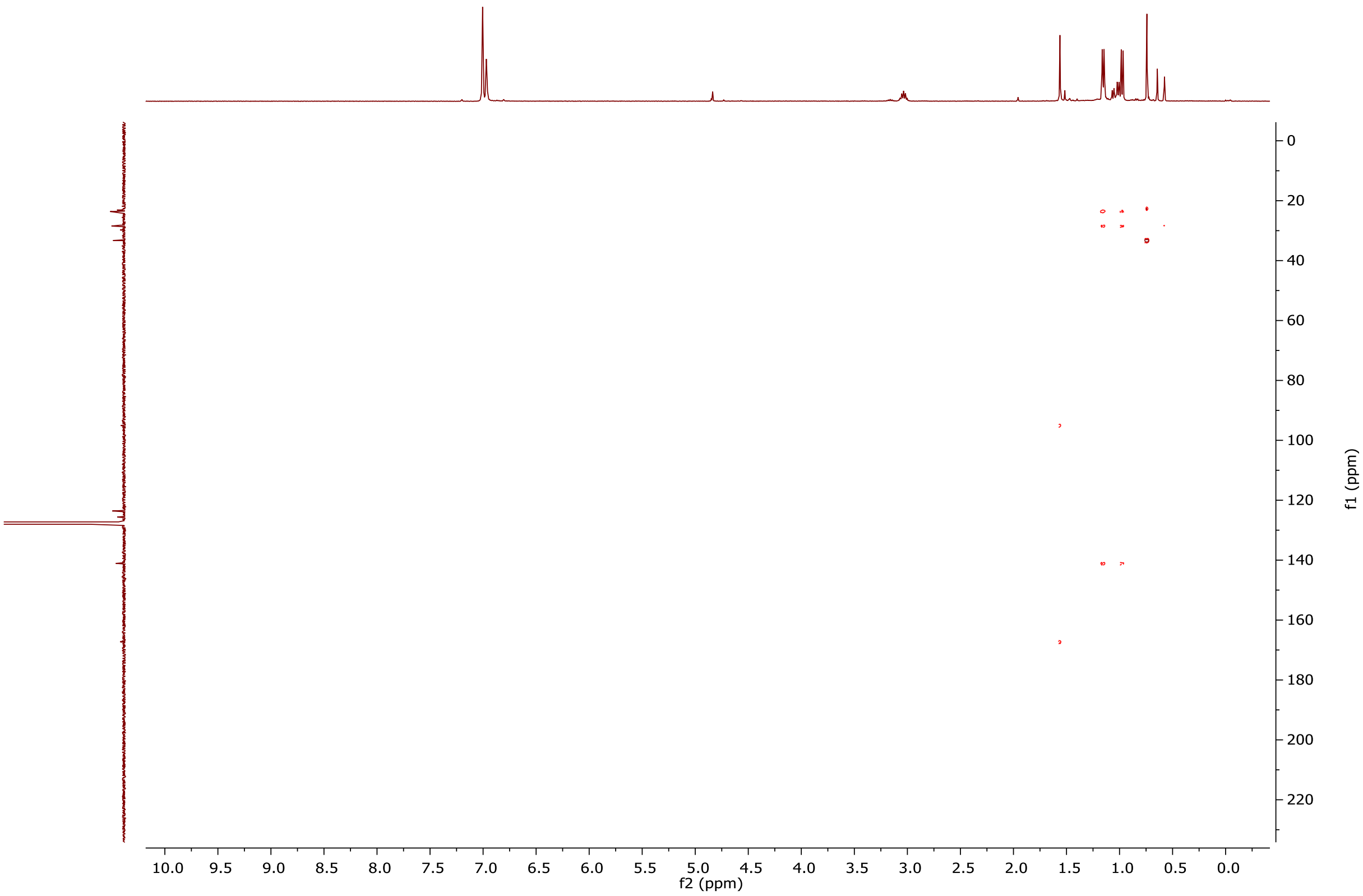

Figure S10. *^{1}H-^{13}C HMBC trace (C_7D_8, 298 K, 400.13, 100.62 MHz) for* ***[(BDI)CaO^{t}Bu]$_2$ (2)***.

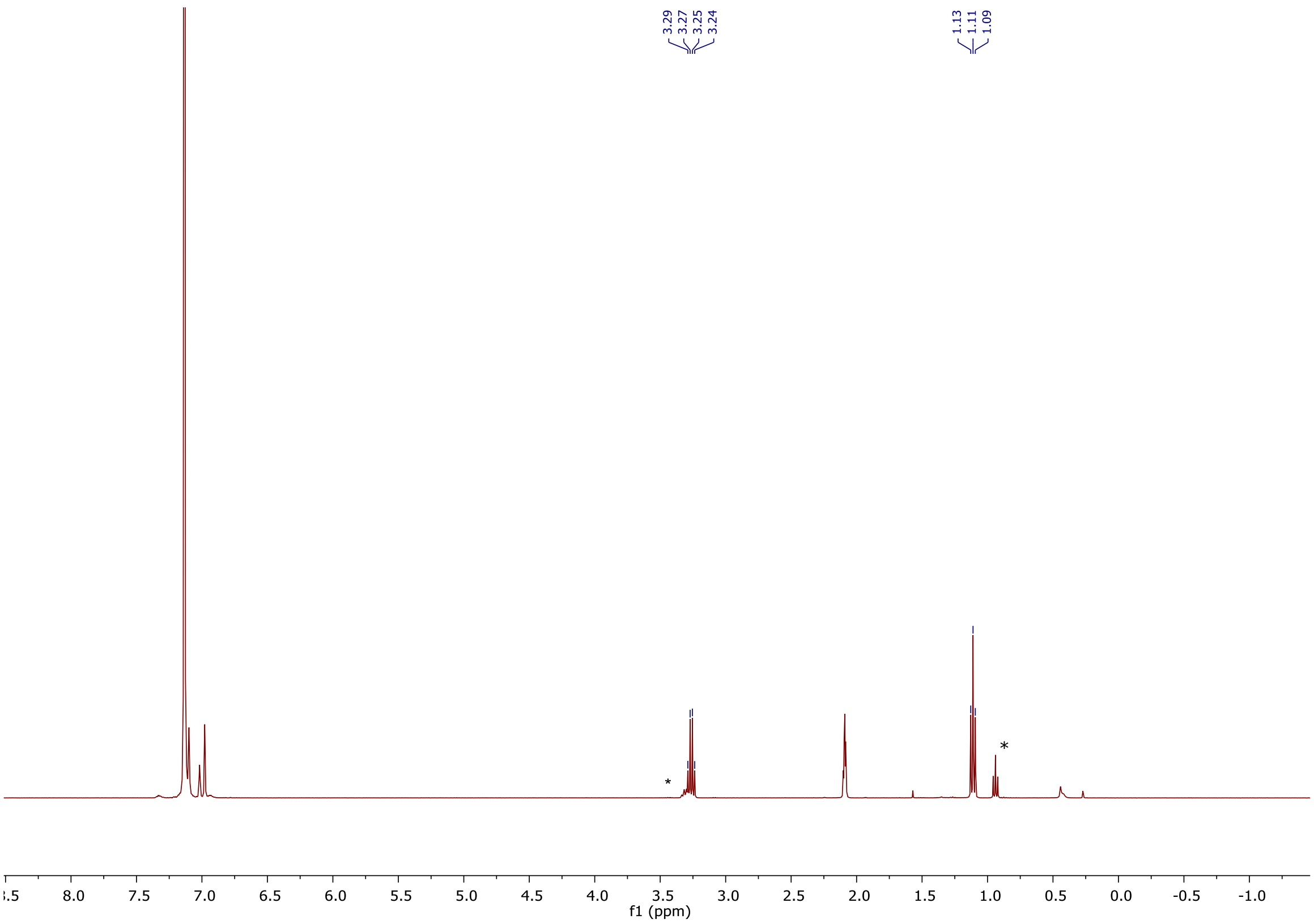

Figure S11. *^{1}H NMR Spectrum (C_7D_8, 298 K, 400.13 MHz) for ^{17}O-diethyl ether. * = residual iodoethane.*

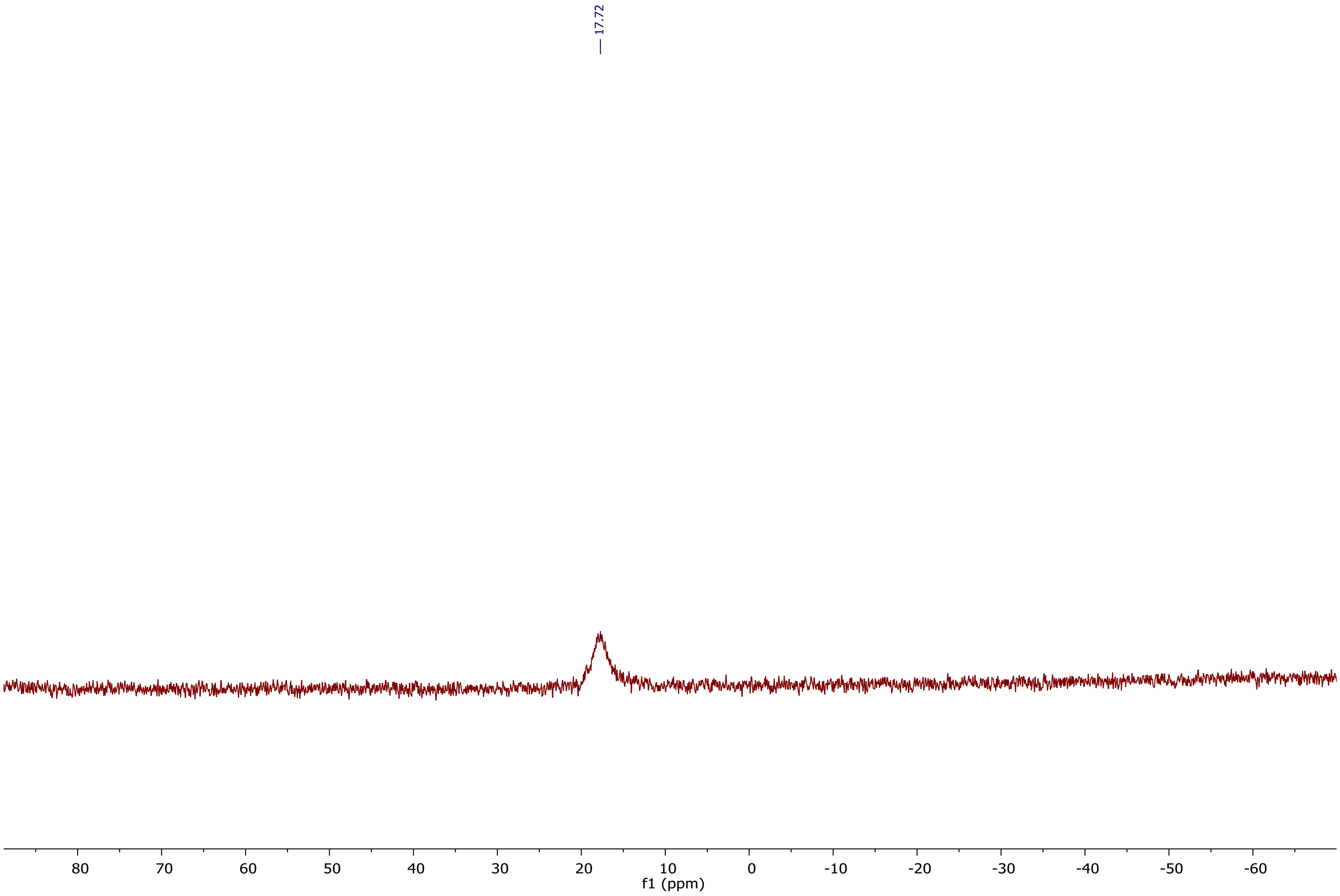

Figure S12. *^{17}O NMR Spectrum (C_7D_8, 298 K, 54.24 MHz) for ^{17}O-diethyl ether.*

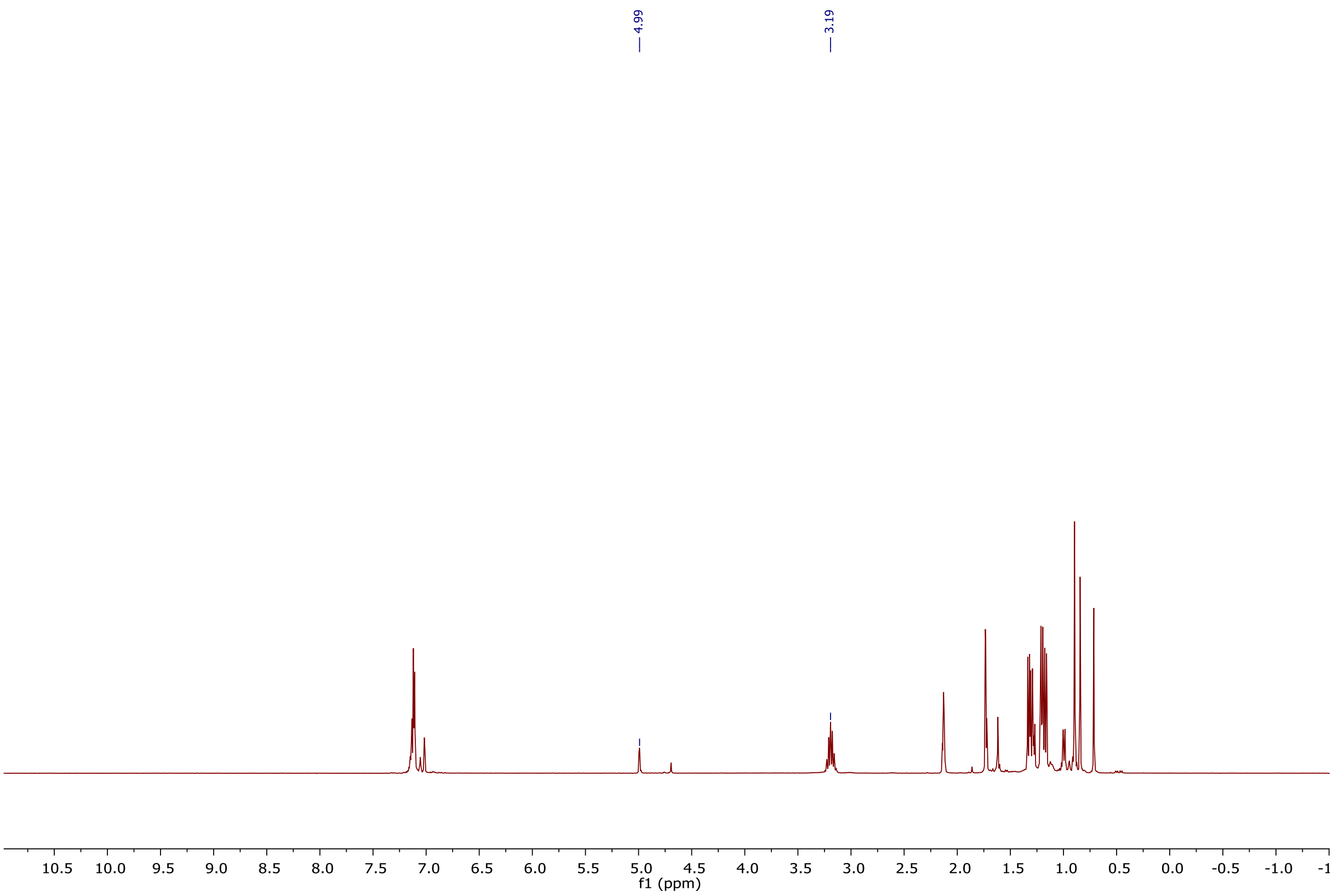

Figure S13. *^{1}H NMR Spectrum (C_7D_8, 298 K, 400.13 MHz) from the reaction of **[(BDI)Ca(Tol)(μ-H)Zn^{t}Bu$_2$] (1)** with d_{10}-diethyl ether.*

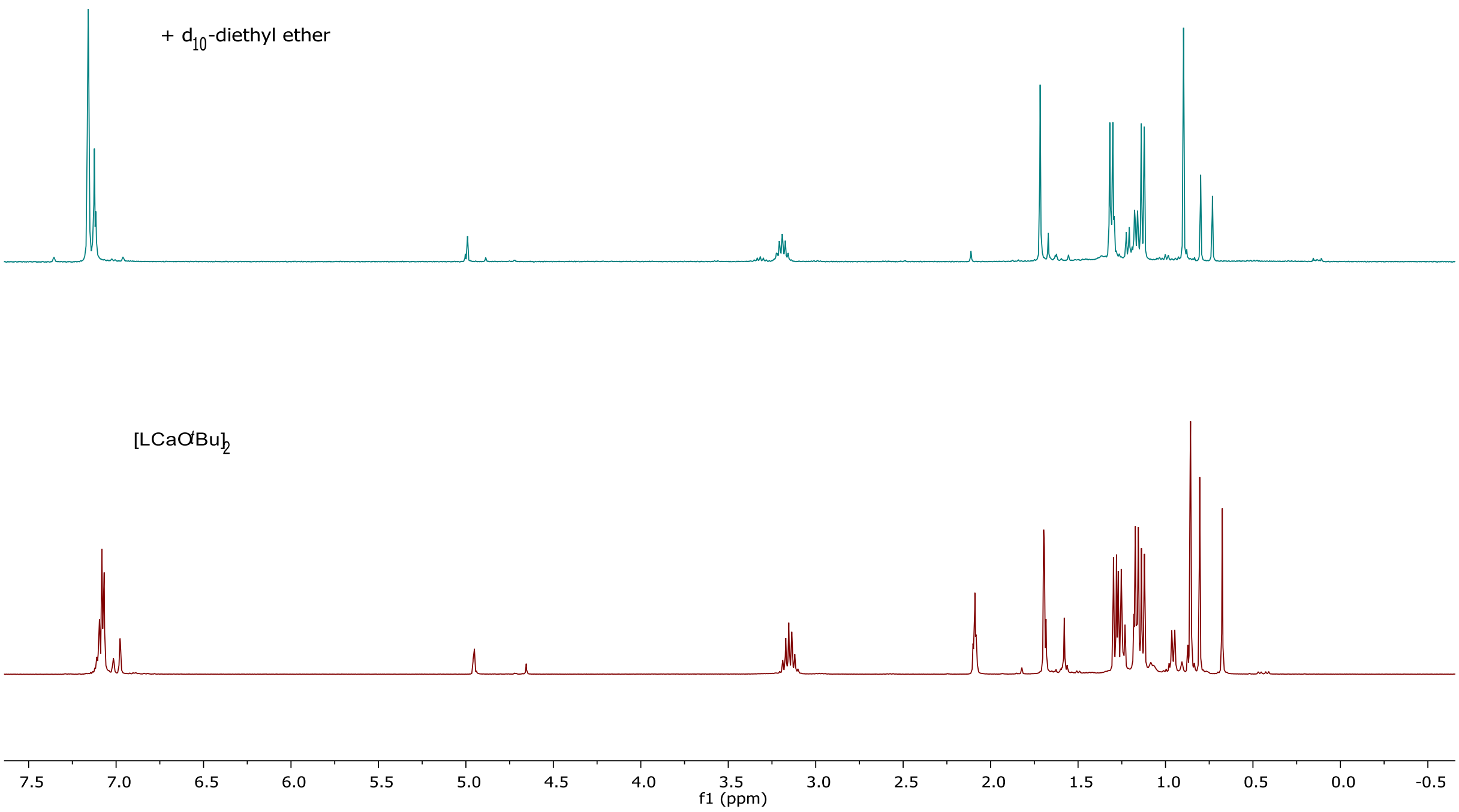

Figure S14. *Overlayed ^{1}H NMR Spectra (C_6D_6, 298 K, 400.13 MHz) from reacting [(BDI)Ca(Tol)(μ-H)Zn^{t}Bu$_2$]* ***(1)*** *with d_{10}-diethyl ether and [(BDI)CaO^{t}Bu]$_2$* ***(2)****.*

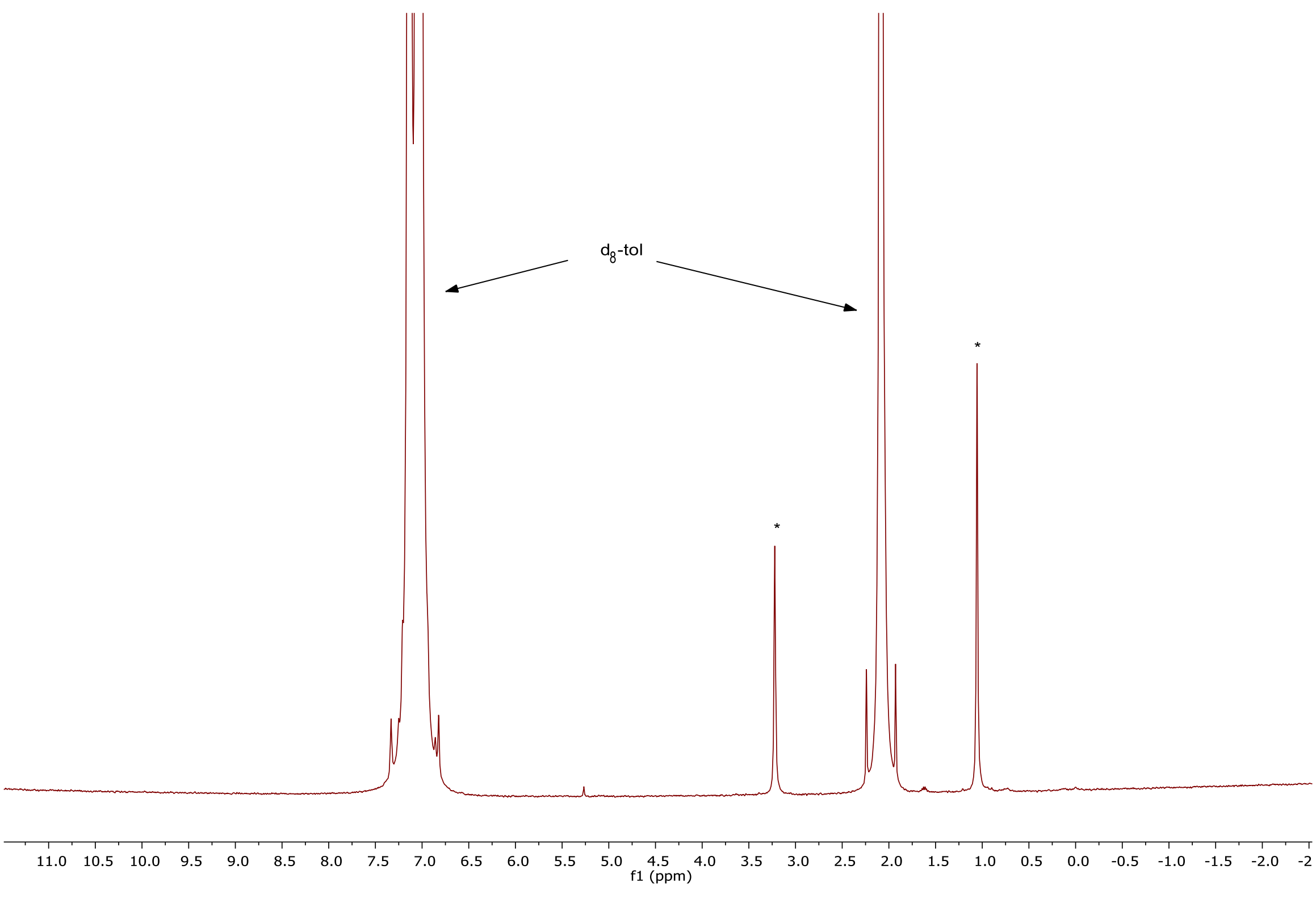

Figure S15. *^{2}H NMR Spectrum (C_7D_8, 298 K, 61.43 MHz) from reacting [(BDI)Ca(Tol)(μ-H)Zn^{t}Bu$_2$]* ***(1)*** *with d_{10}-diethyl ether. * = d_{10}-diethyl ether.*

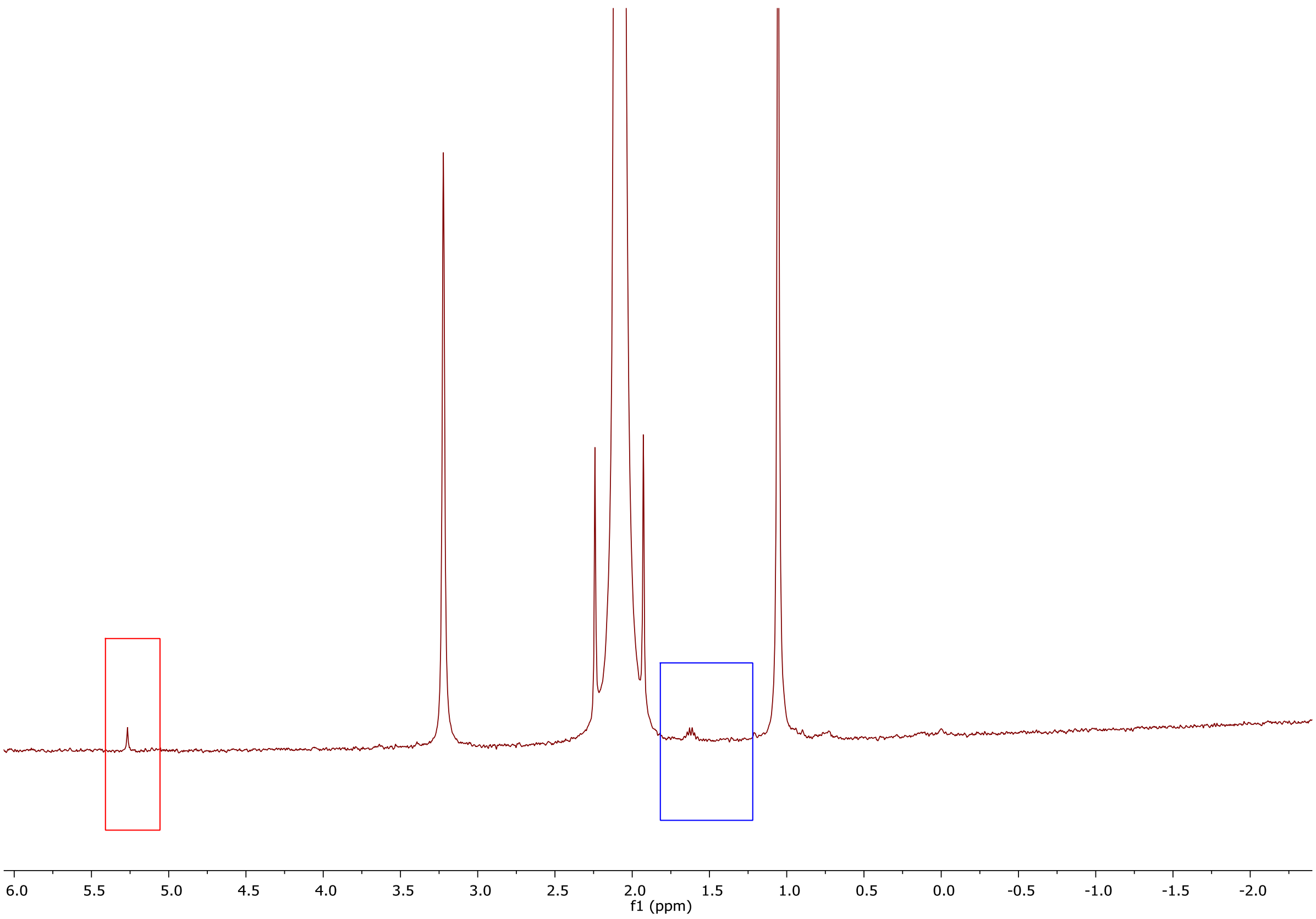

Figure S16. *^{2}H NMR Spectrum (C_7D_8, 298 K, 61.43 MHz) from reacting [(BDI)Ca(Tol)(μ-H)Zn^{t}Bu$_2$]* ***(1)*** *with d_{10}-diethyl ether. Highlighting deuterated ethene (red), and the deuterium signal for 2-methylpropane-2d (C_4H_9D; blue).*

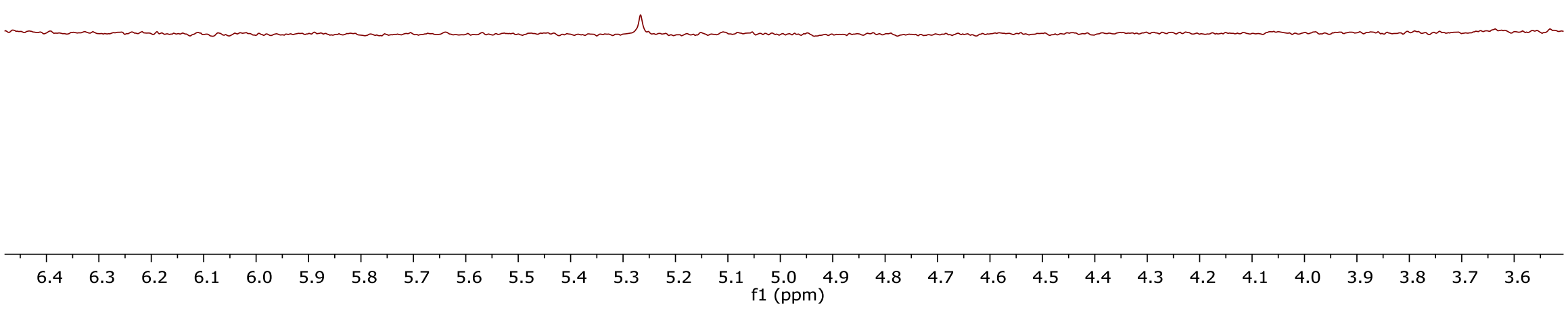

Figure S17. *^{2}H NMR Spectrum (C_7D_8, 298 K, 61.43 MHz) from reacting [(BDI)Ca(Tol)(μ-H)Zn^{t}Bu$_2$]* ***(1)*** *with d_{10}-diethyl ether. Expanded on the deuterated ethene resonance.*

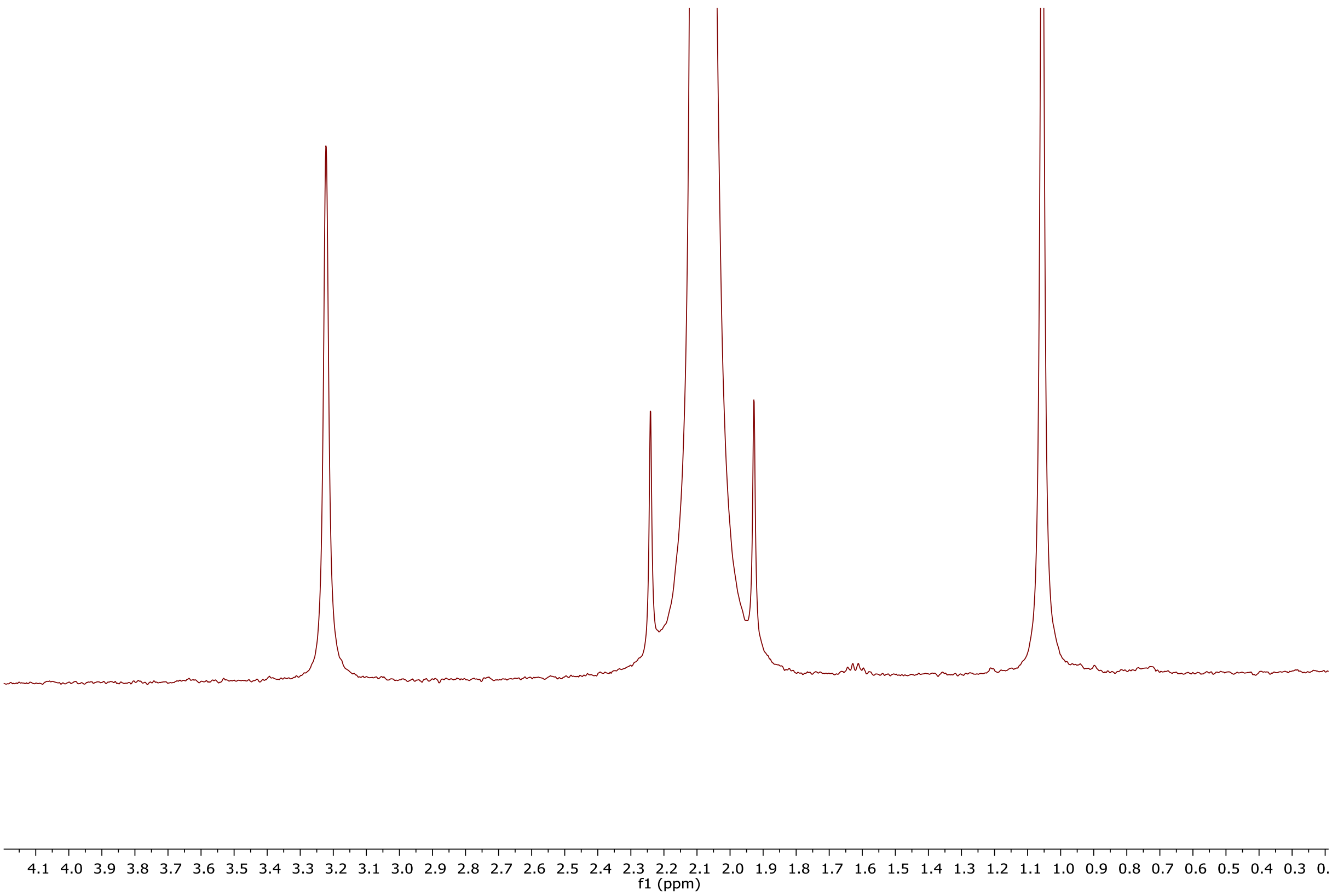

Figure S18. *^{2}H NMR Spectrum (C_7D_8, 298 K, 61.43 MHz) from reacting [(BDI)Ca(Tol)(μ-H)Zn^{t}Bu$_2$]* ***(1)*** *with d_{10}-diethyl ether. Expanded on the deuterated ethene resonance.*

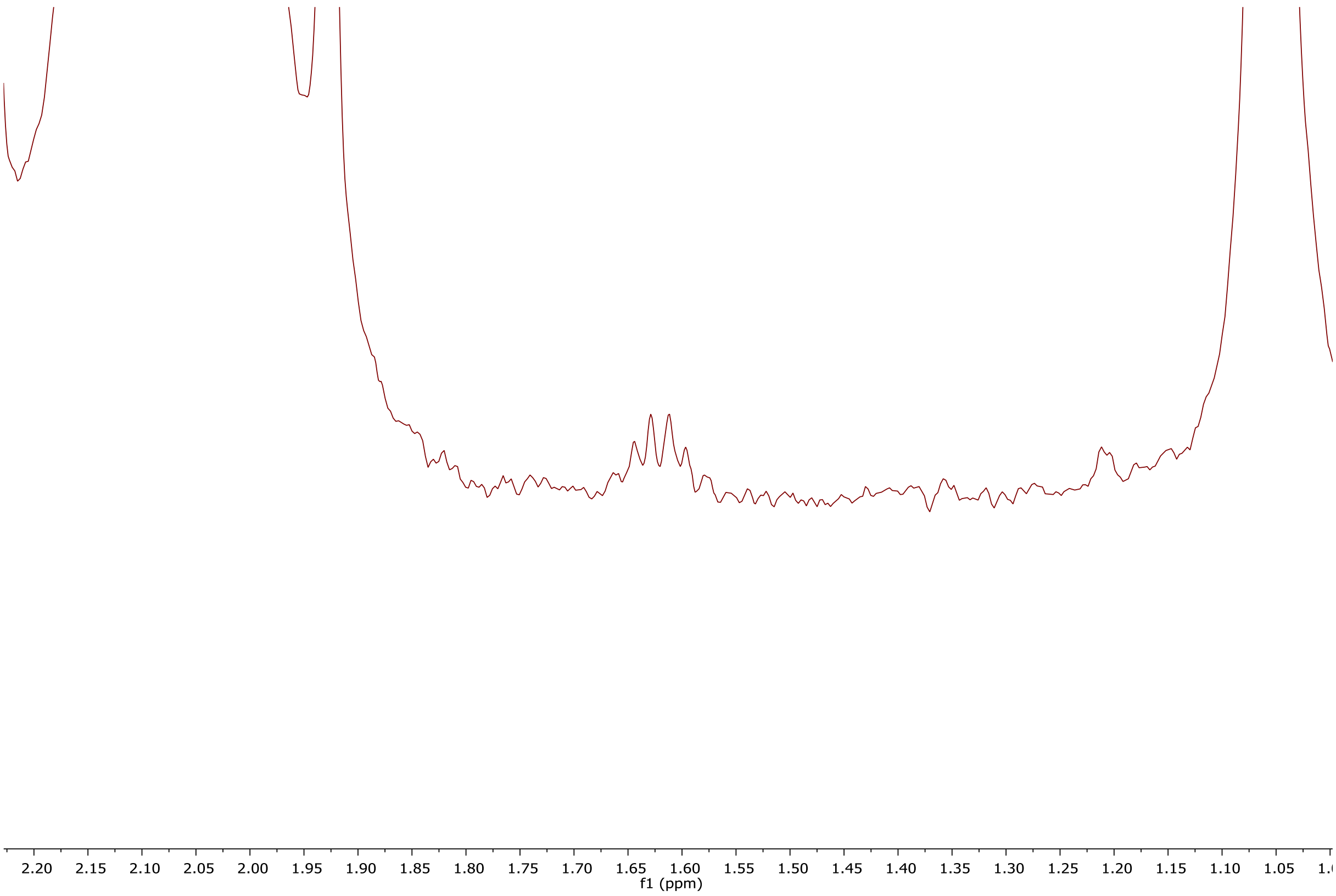

Figure S19. *^{2}H NMR Spectrum (C_7D_8, 298 K, 61.43 MHz) from reacting [(BDI)Ca(Tol)(μ-H)Zn^{t}Bu$_2$]* ***(1)*** *with d_{10}-diethyl ether. Expanded on the deuterated ethene resonance.*

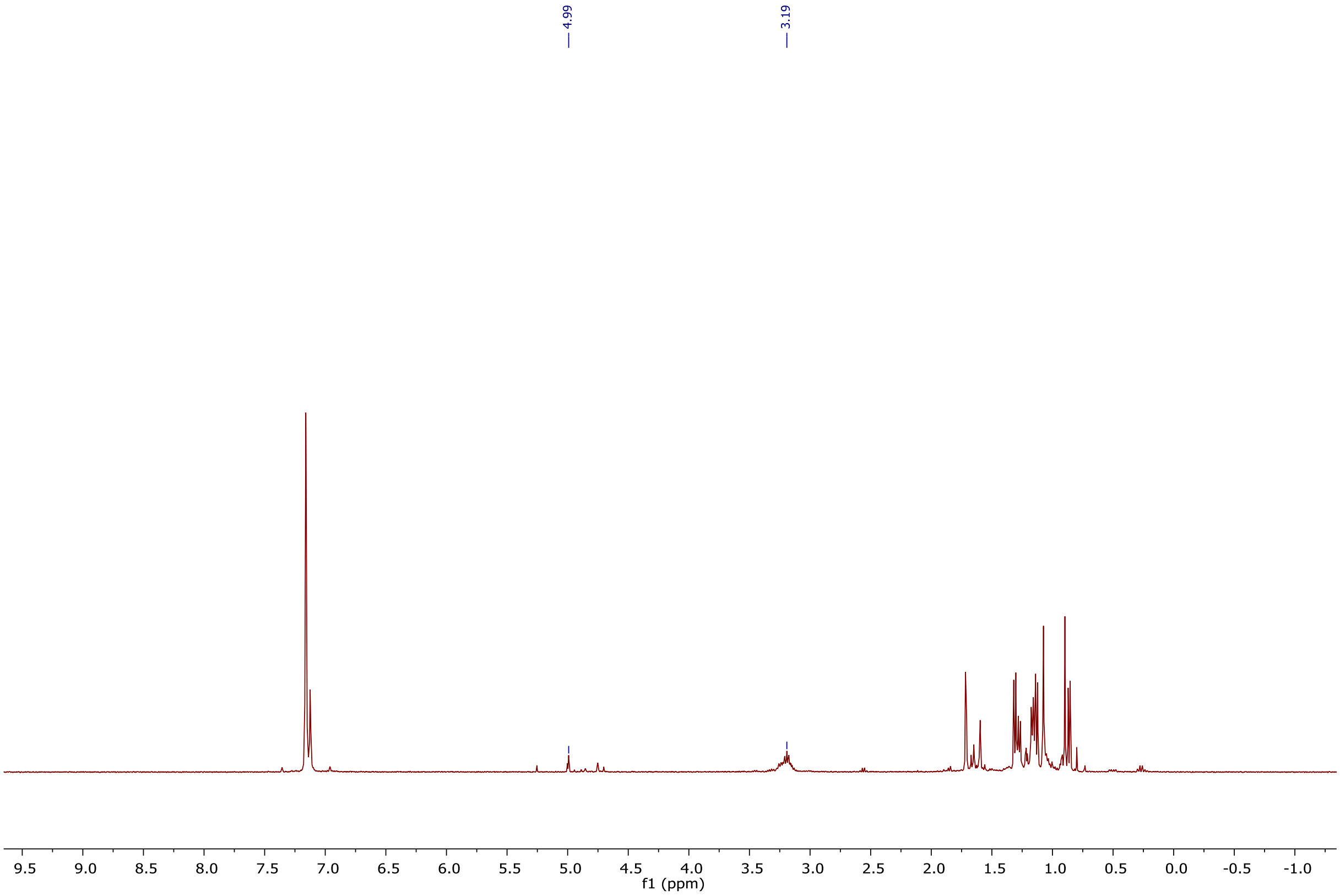

Figure S20. *^{1}H NMR Spectrum (C_6D_6, 298 K, 400.13 MHz) from reacting [(BDI)Ca(Tol)(µ-H)Zn[t]Bu[2]]* ***(1)*** *with [17]O-diethyl ether, demonstrating the formation of [(BDI)CaO[t]Bu][2]* ***(2)***.

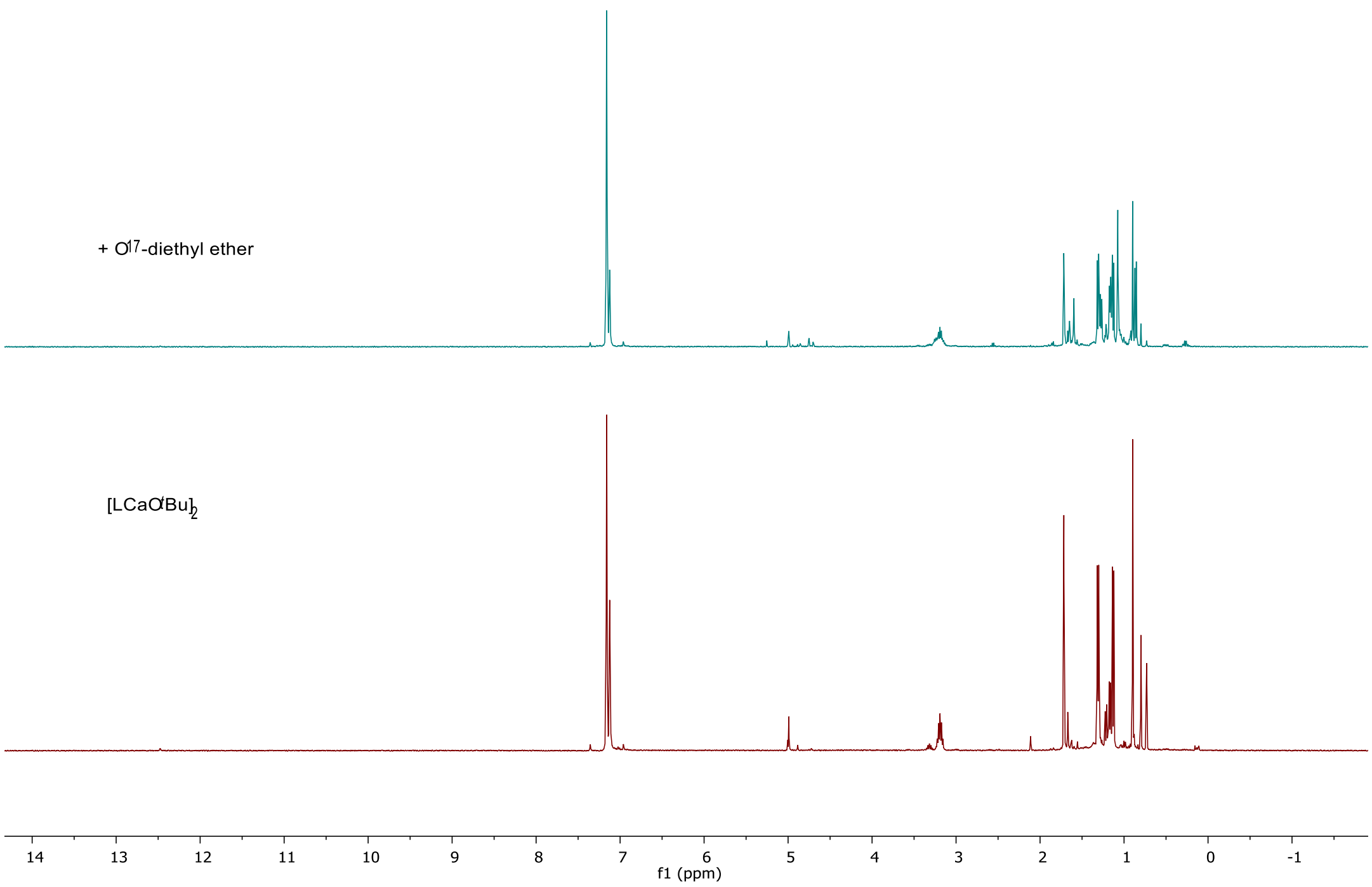

Figure S21. *Overlayed ^{1}H NMR Spectra (C_6D_6, 298 K, 400.13 MHz) from reacting [(BDI)Ca(Tol)(µ-H)Zn[t]Bu[2]]* ***(1)*** *with [17]O-diethyl ether and [(BDI)CaO[t]Bu][2]* ***(2)***.

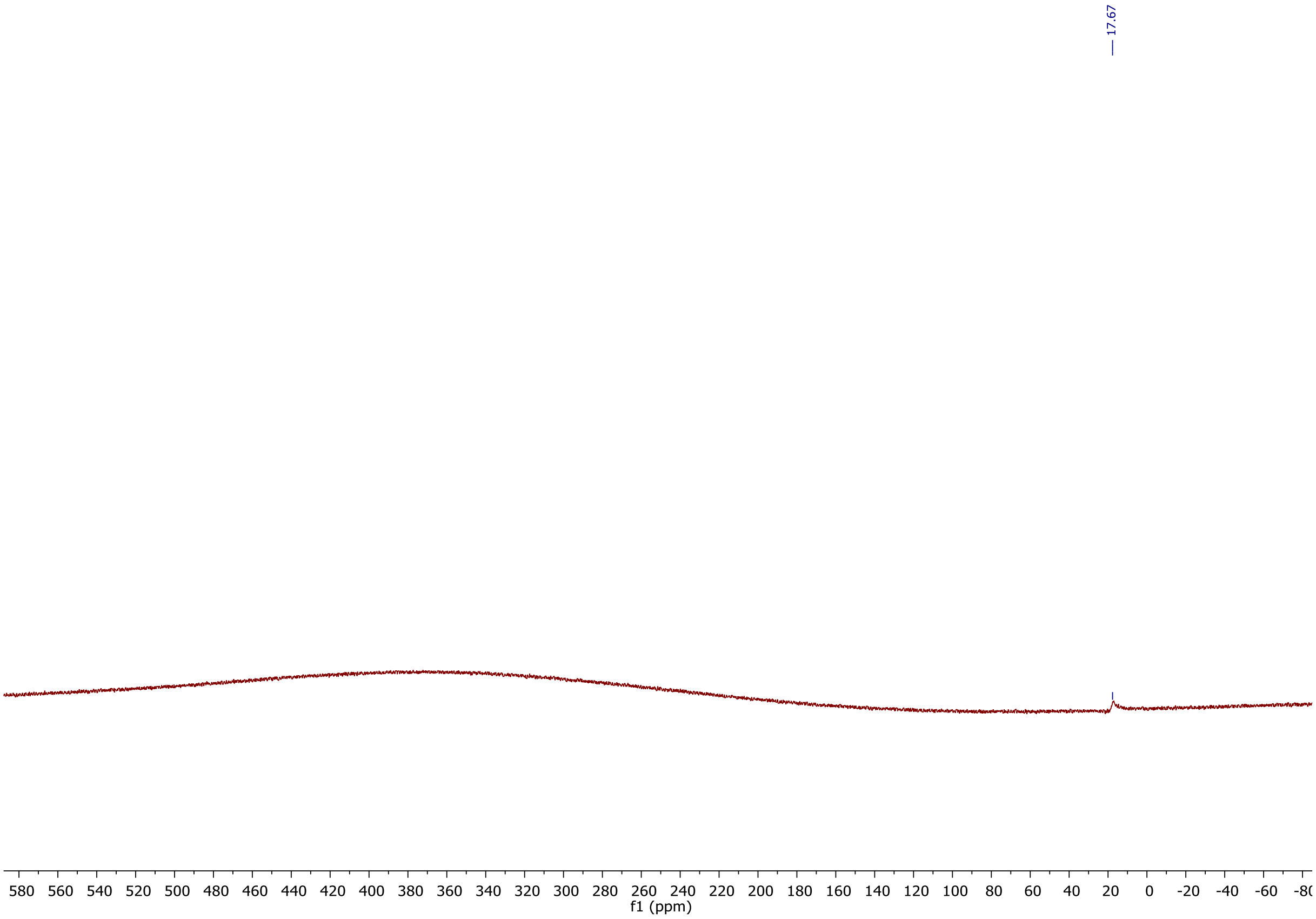

Figure S22. *^{17}O NMR Spectrum (C_7D_8, 298 K, 54.24 MHz) from reacting [(BDI)Ca(Tol)(μ-H)Zn^{t}Bu$_2$]* ***(1)*** *with ^{17}O-diethyl ether.*

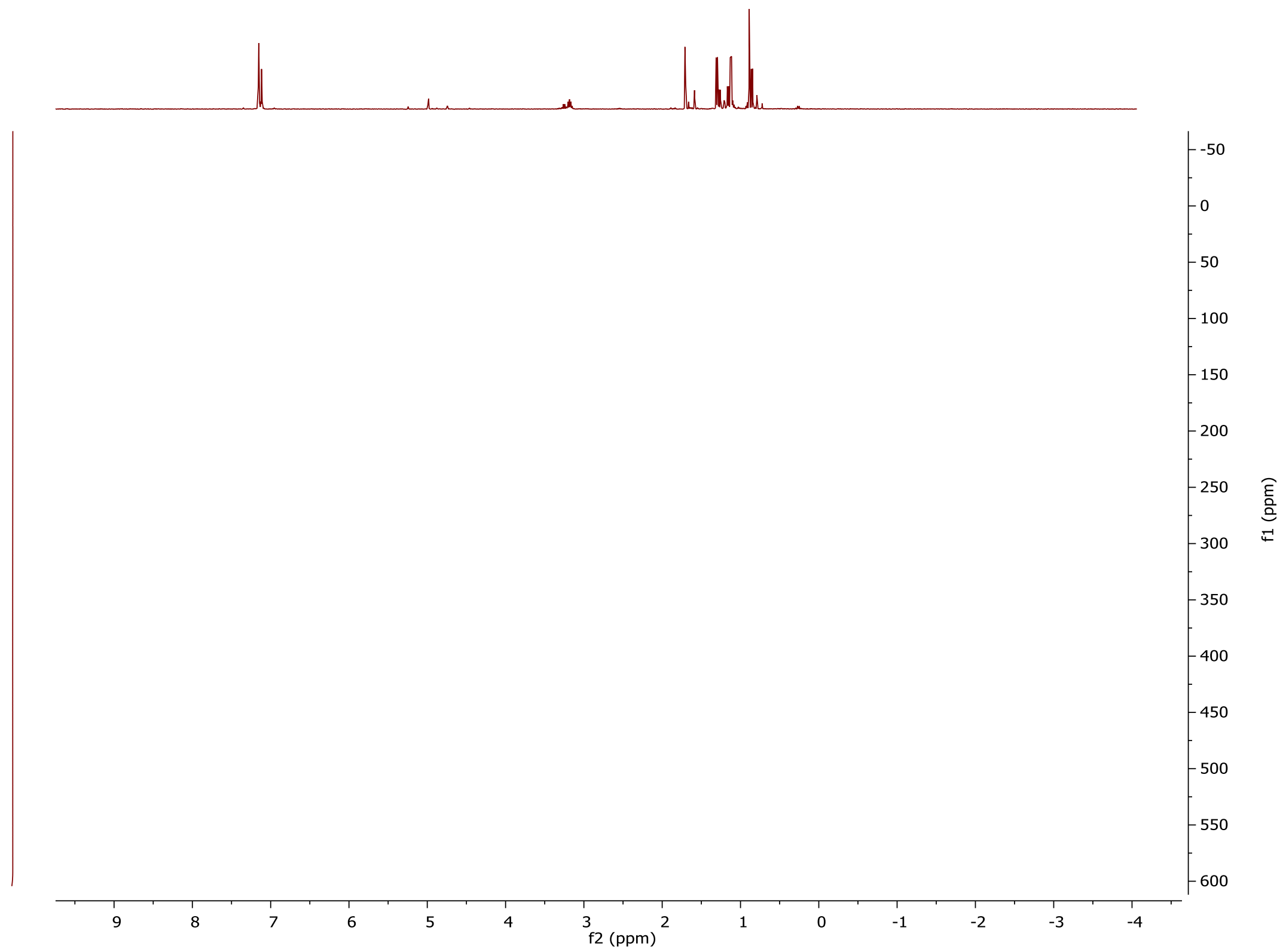

Figure S23. *^{1}H-^{17}O HSQC trace (C_7D_8, 253 K, 400.13, 54.24 MHz) for* ***$[(BDI)Ca^{17}O^{t}Bu]_2$***.

NMR yields of the ethers, ^{i}Pr, ^{n}Bu, ^{n}Hex

[(BDI)Ca(Tol)(μ-H)Zn^{t}Bu$_2$] (7.6 mg (^{i}Pr), 4.5 mg (^{n}Bu), 8.5 mg (^{n}Hex), 0.0107, 0.00616, 0.0116 mmol) and hexamethylbenzene (4 mg, 0.025 mmol) were introduced into a J. Young's NMR tube. d$_8$-toluene (0.6 cm^3) was added along with two equivalents of the selected ether (^{i}Pr, ^{n}Bu, ^{n}Hex). The reaction was left to sit for 16 hours before spectroscopic analysis. In each case, [(BDI)CaO^{t}Bu]$_2$, was formed alongside the corresponding alkene, which was identified by the characteristic vinylic resonance, consistent with literature values (Propylene,[6] Butene,[6] Hexene).[7] NMR yields of [(BDI)CaO^{t}Bu]$_2$ formed were determined relative to the internal standard using a D1 of 5 seconds.

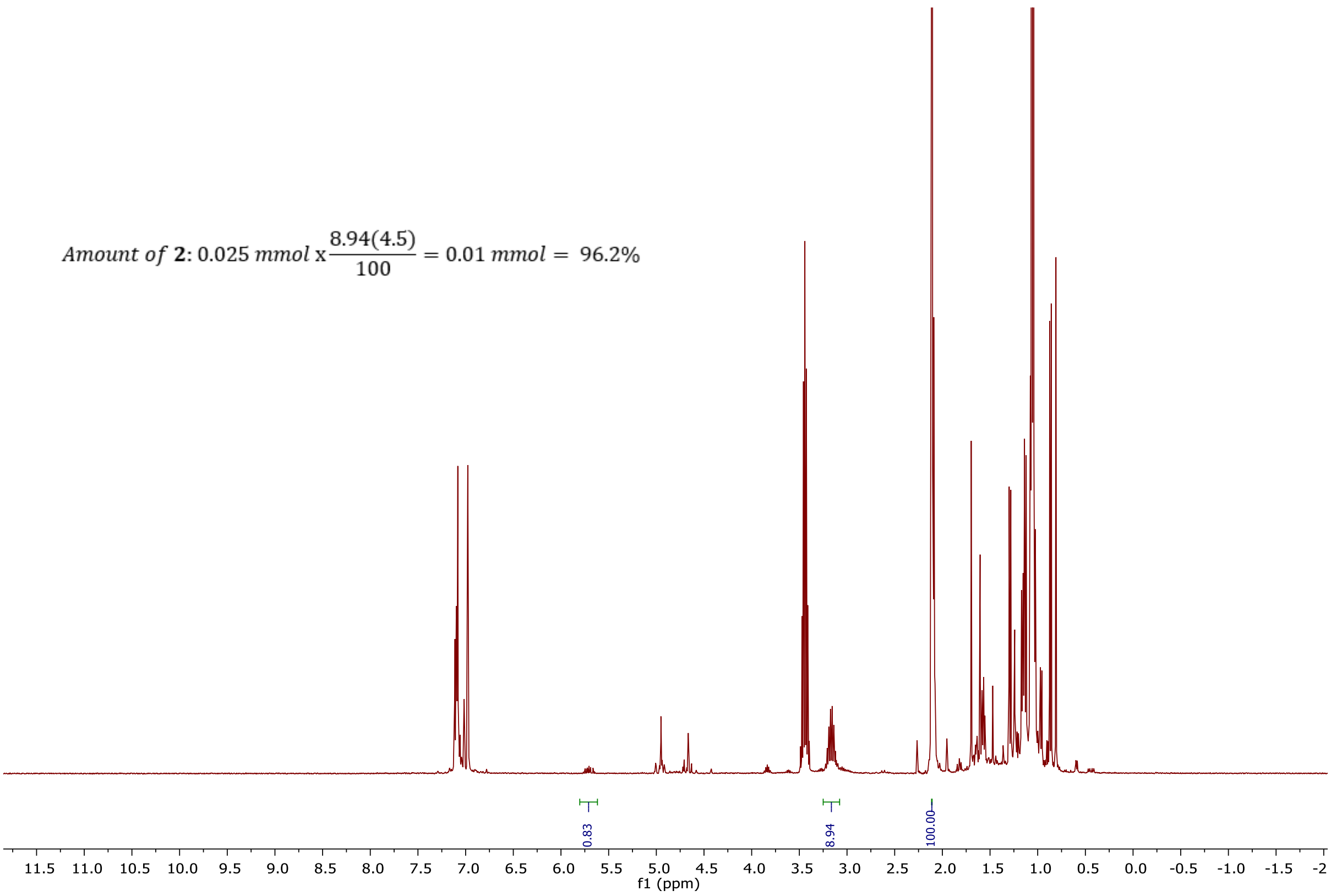

Figure S24. *^{1}H NMR Spectrum (C$_7$D$_8$, 298 K, 400.13 MHz) from reacting **[(BDI)Ca(Tol)(μ-H)Zn^{t}Bu$_2$] (1)** with ^{i}PrO^{i}Pr in the presence of hexamethylbenzene.*

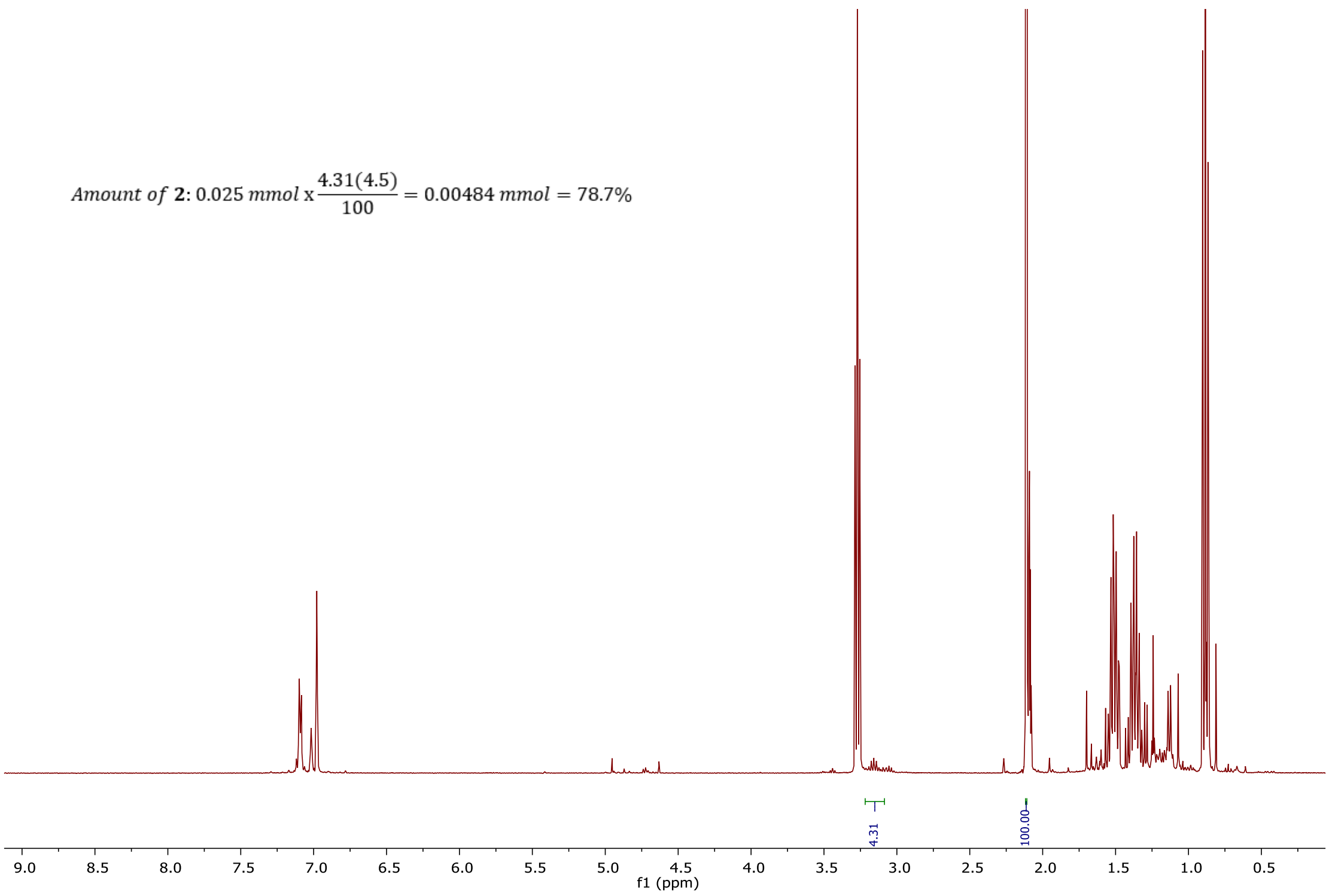

Figure S25. *^{1}H NMR Spectrum (C_7D_8, 298 K, 400.13 MHz) from reacting* ***[(BDI)Ca(Tol)(μ-H)Zn^{t}Bu$_2$] (1)*** *with ^{n}BuO^{n}Bu in the presence of hexamethylbenzene.*

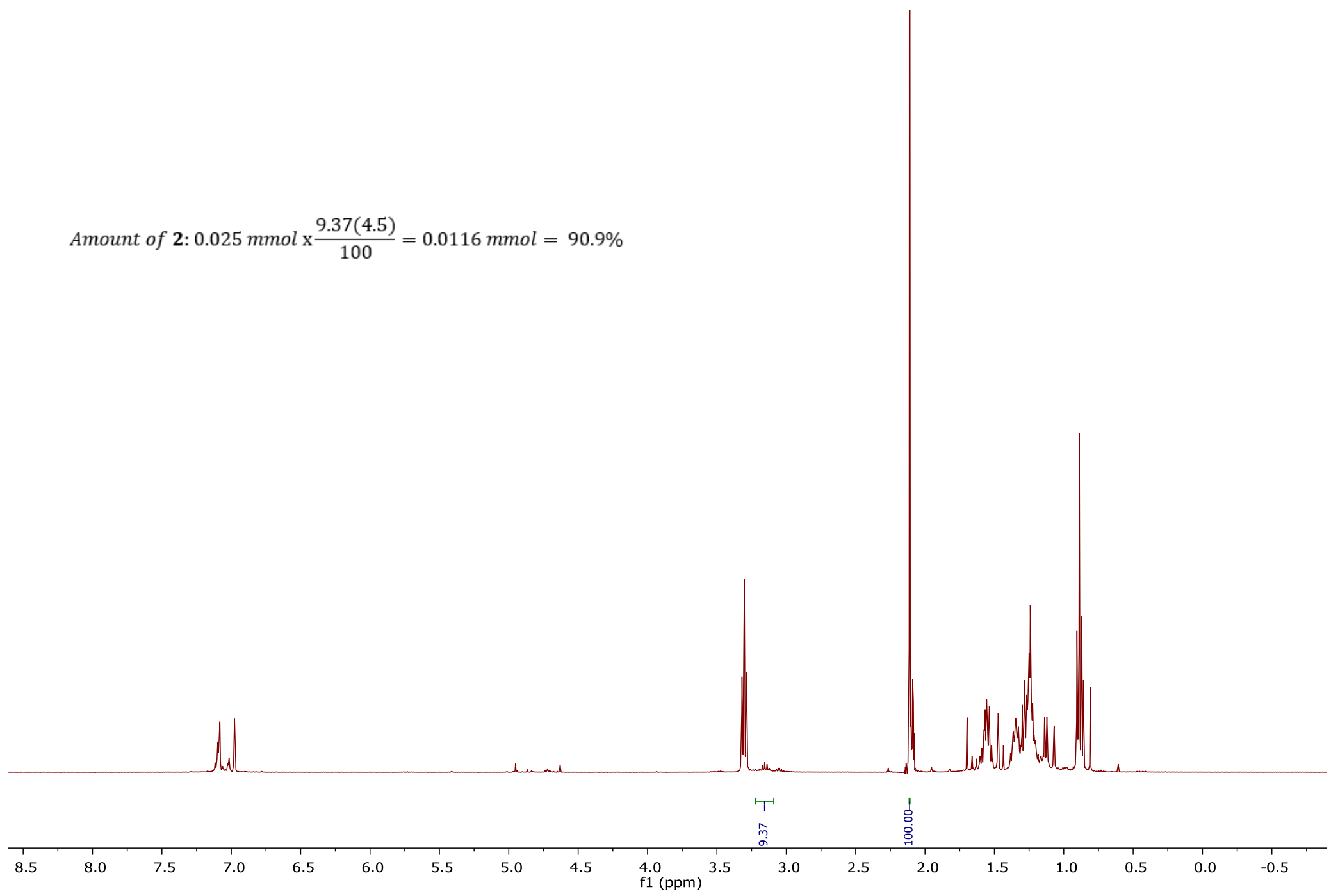

Figure S26. *^{1}H NMR Spectrum (C_7D_8, 298 K, 400.13 MHz) from reacting* ***[(BDI)Ca(Tol)(μ-H)Zn^{t}Bu$_2$] (1)*** *with ^{n}HexO^{n}Hex in the presence of hexamethylbenzene.*

Reaction with THF

THF (1.84 µl, 0.023 mmol) was added to a benzene solution of [(BDI)Ca(Tol)(µ-H)Zn^{t}Bu$_2$] (8.3 mg mg, 0.011 mmol). The sample was left to sit for 16 hours, observing the deposition of metallic powder, believed to be Zn metal. Although the byproducts were not identified the formation of [(BDI)CaO^{t}Bu]$_2$ was observed spectroscopically and confirmed crystallographically.

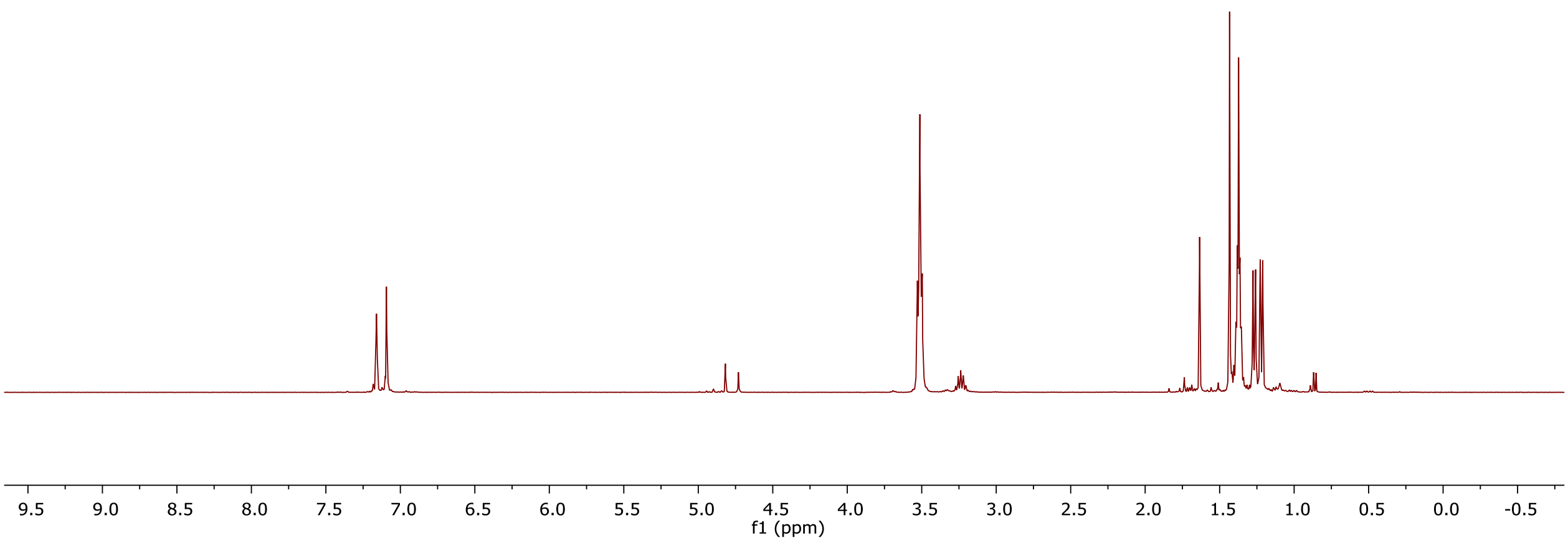

Figure S27. *^{1}H NMR Spectrum (C_6D_6, 298 K, 400.13 MHz) from reacting **[(BDI)Ca(Tol)(µ-H)Zn^{t}Bu$_2$] (1)** with THF.*

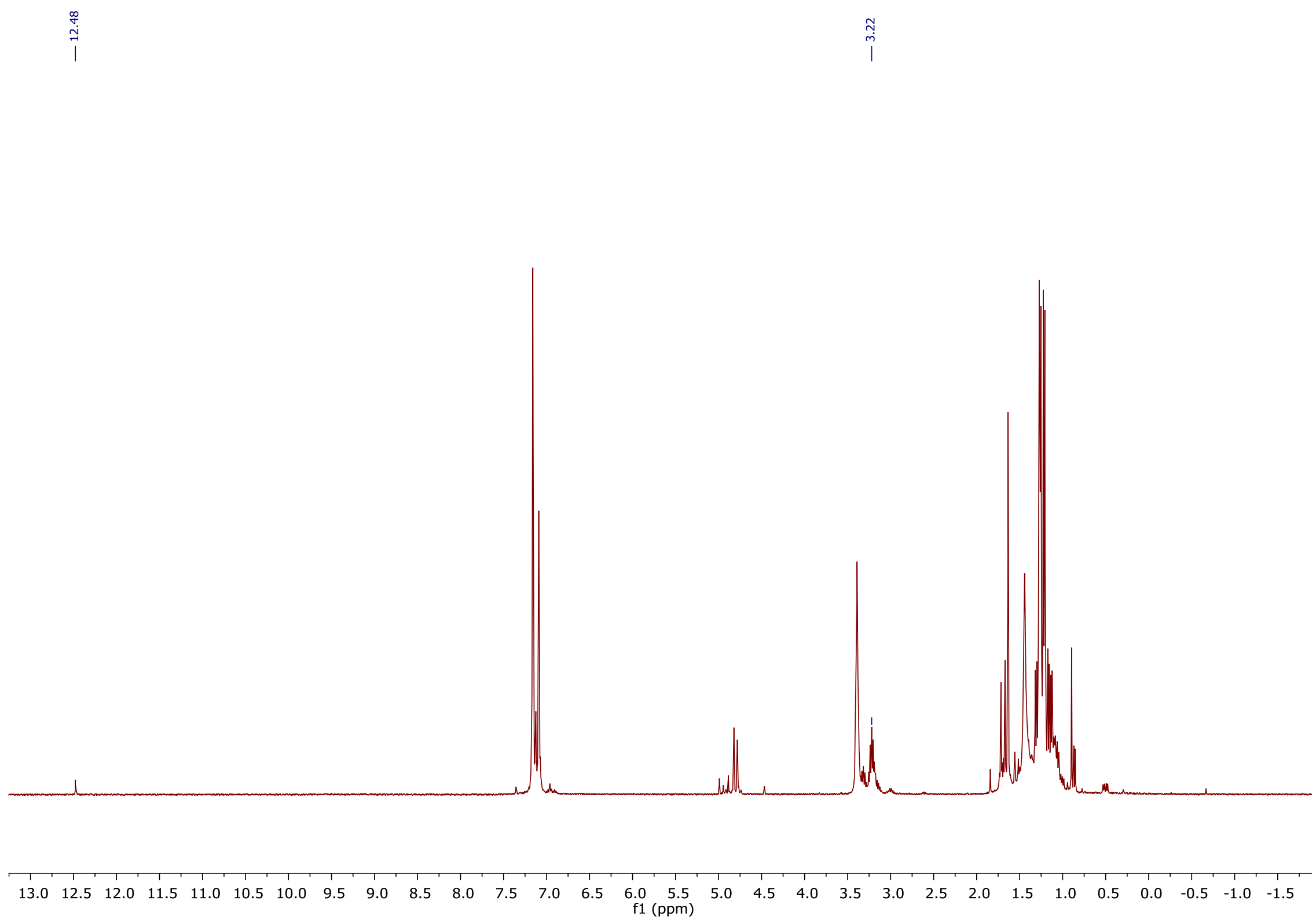

Figure S28. *^{1}H NMR Spectrum (C_6D_6, 298 K, 400.13 MHz) from reacting* ***[(BDI)Ca(Tol)(μ-H)Zn^{t}Bu$_2$] (1)*** *with THF then removing THF and redissolving in benzene. Compound* ***2*** *and protonated NacNac are present.*

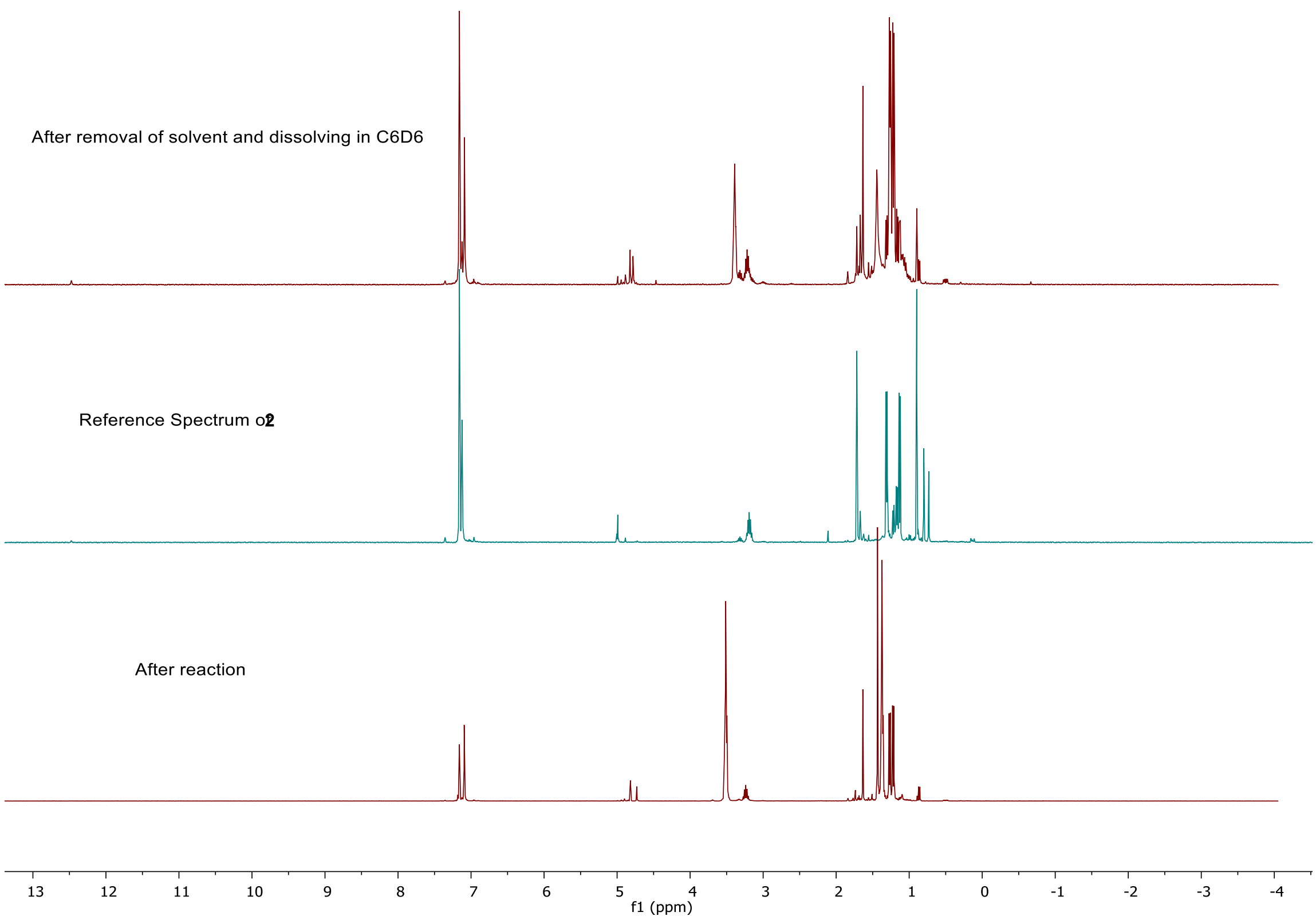

Figure S29. *^{1}H NMR Spectra (C_6D_6, 298 K, 400.13 MHz) stacked spectra showing the reaction outcome from reacting* ***1*** *with THF, the spectrum after removing volatiles and redissolving in C_6D_6 and a reference spectrum of* ***2*** *to identify the formation of* ***2****.*

Mass Spectrometry

Using a GilAir Plus Sampling Pump, the headspace of the reaction between [(BDI)Ca(Tol)(μ-H)ZntBu$_2$] and d$_{10}$-Et$_2$O was loaded onto an offline collection tube and subsequently analysed by GC-MS, readily observing the presence of 2-Methylpropane-2d (isobutane):

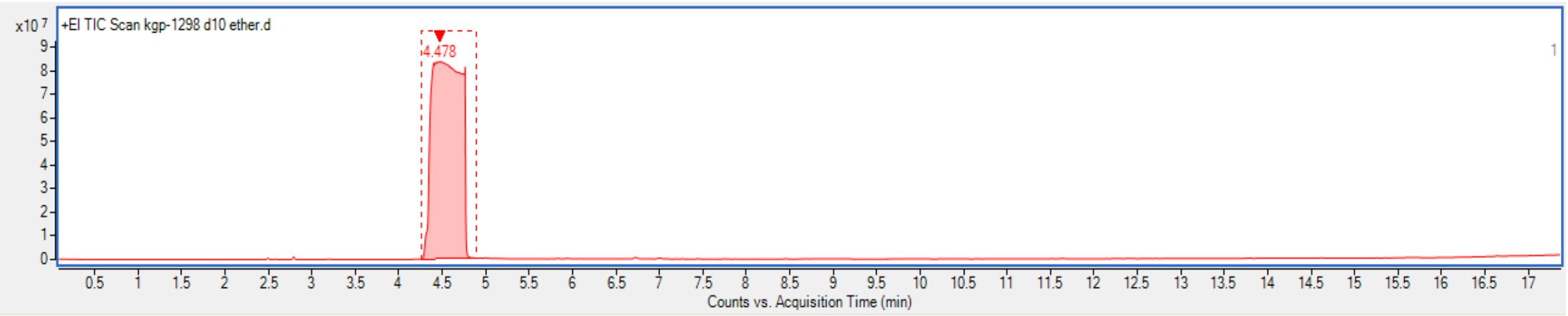

Figure S30. *GC-MS chromatogram from the headspace of the reaction between [(BDI)Ca(Tol)(μ-H)ZntBu$_2$] (**1**) and d$_{10}$-Et$_2$O. Expansion of the peak for 2-Methylpropane-2d.*

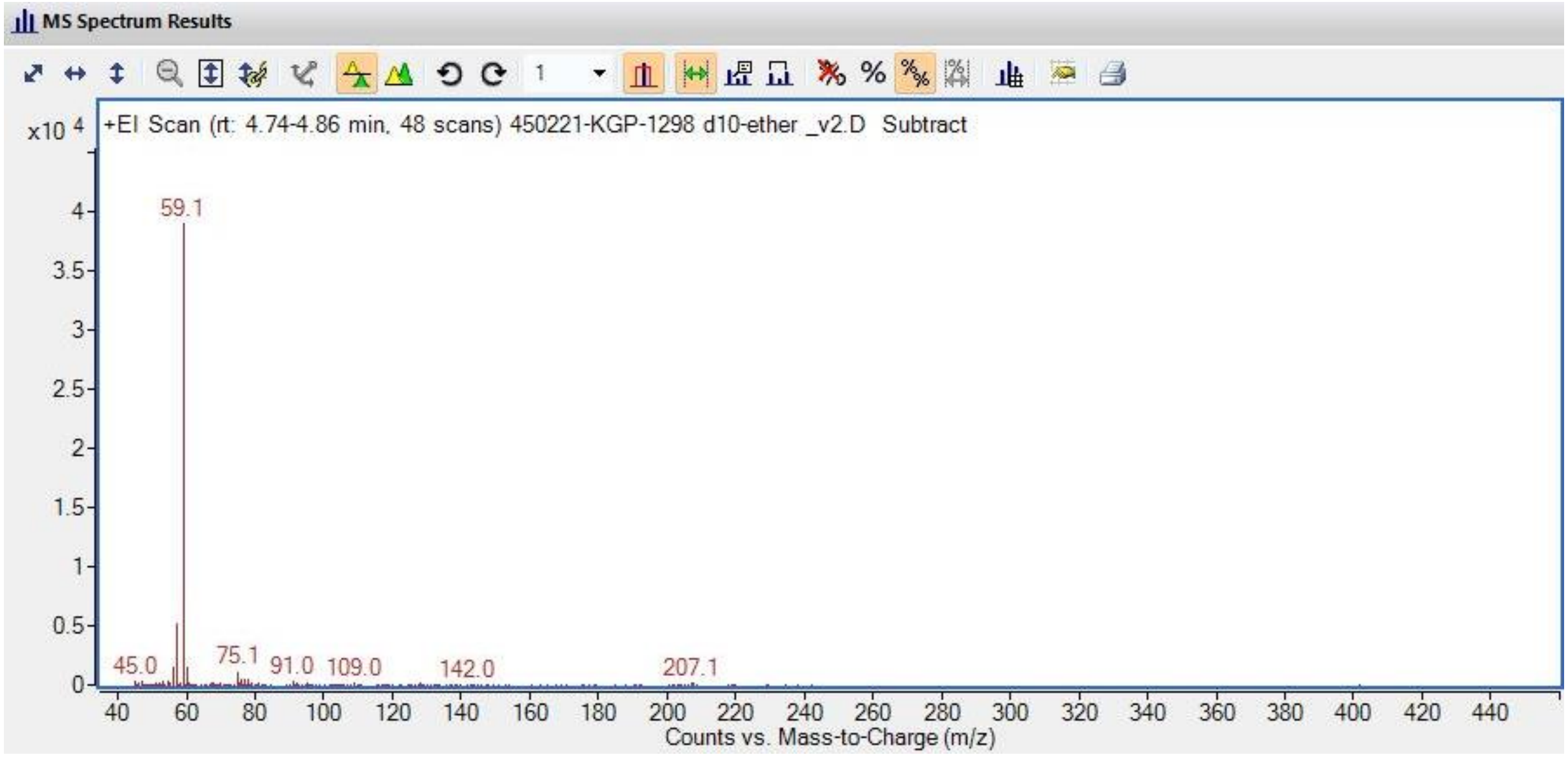

Figure S31. *GC-MS trace from the headspace of the reaction between [(BDI)Ca(Tol)(μ-H)ZntBu$_2$] (**1**) and d$_{10}$-Et$_2$O, showing the molecular ion for 2-Methylpropane-2d.*

The J. Young's NMR tube containing the reaction between [(BDI)Ca(Tol)(μ-H)Zn^{t}Bu$_2$] and ^{17}O-Et$_2$O (20-24.9 % labelled), was exposed to air, and an aliquot was transferred to a GC-MS vial for analysis:

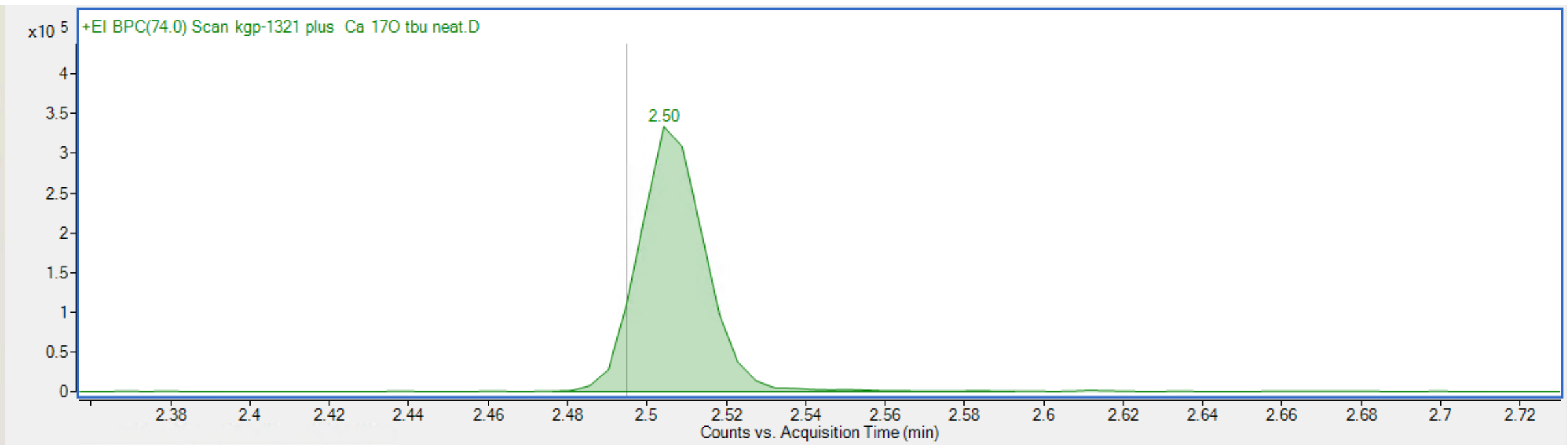

Figure S32. *GC-MS chromatogram of the resulting spectra from the reaction between [(BDI)Ca(Tol)(μ-H)Zn^{t}Bu$_2$] (**1**) and ^{17}O-Et$_2$O (20-24.9 % labelled). Expansion of the peak for tert-butanol.*

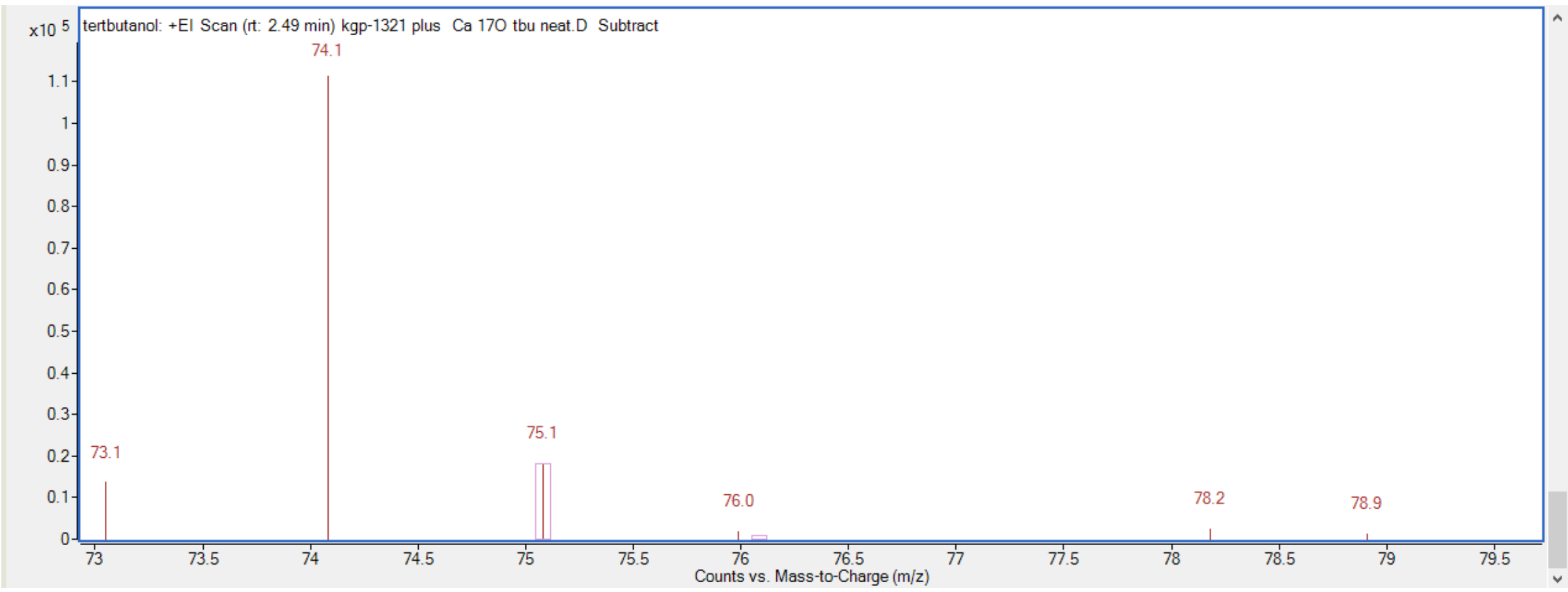

Figure S33. *GC-MS trace from the reaction between [(BDI)Ca(Tol)(μ-H)ZntBu$_2$] (**1**) and ^{17}O-Et$_2$O (20-24.9 % labelled), after exposure to air, showing the molecular ion for ^{17}O-labelled and unlabelled tert-butanol.*

Crystallographic Details

Single Crystal X-ray diffraction data for compound1 **1** (s24msh54) were collected on an Agilent SuperNova EosS2 diffractometer using Cu-Kα (1.54184 Å) radiation, while data for compounds **2** (y24msh13) and **3** (y24msh26) were collected on a XtaLAB Synergy, Dualflex, HyPix-Arc 100 diffractometer using Cu-Kα radiation. In each case, the crystals were maintained at 150 K during data collection. Using Olex2,[8] the structures were solved with the olex2.solve[9] structure solution program or ShelXT and refined with the ShelXL[10] refinement package using Least−Squares minimisation.

The asymmetric unit in **1** contains one molecule of the bimetallic complex and one molecule of toluene. Disorder was addressed (55:45) for the toluene ligand and for C42-C44 in the former, while a 3-way split (67:20:13 ratio) was incorporated into the refinement for the guest solvent. Distance and ADP restraints were employed, on merit, in disordered regions to assist convergence. The hydride ligand was located and refined without restraints.

The asymmetric unit for **2** is comprised of half of a dimeric molecule, which is completed by virtue of space-group inversion symmetry. A solvent mask was required for the molecules of hexane associated with the dimer, which was readily implemented to assist convergence. The solvent mask is consistent with the presence of 1[C_6H_{14}] per formula unit which accounts for 50 electrons per unit cell.

The asymmetric unit for **3** is comprised of a single tetrametallic molecular unit comprised of 1 zinc and 3 calcium atoms. Disorder was identified on the Dipp unit attached to N1: C6-C17, the Dipp isopropyl unit C24-C26 and the tert-butyl functionality (C90-93), which were all readily modelled in a 63:37, 80:20 and 63:37 ratio, respectively. Distance and ADP restraints were employed, on merit, to assist convergence. A solvent mask was required for the two hexane molecules associated with the dimer, the solvent mask if consistent with the presence of 2[C_6H_{14}] molecules per asymmetric unit, which accounts for 200 electrons per unit cell.

*Table S1. Crystal Data and Structure Refinement for Compounds **1** - **3**.*

Identification code	s24msh54 (**1**)	y24msh13 (**2**)	y24msh26 (**3**)
Empirical formula	$C_{51}H_{76}CaN_2Zn$	$C_{72}H_{114}Ca_2N_4O_2$	$C_{105}H_{168}Ca_3N_6Zn$
Formula weight	822.58	1147.83	1700.05
Crystal system	monoclinic	triclinic	triclinic
Space group	$P2_1/n$	P-1	P-1
a/ Å	12.2409(1)	10.8198(2)	14.6211(4)
b/ Å	22.4230(2)	13.0796(3)	17.4498(3)
c/ Å	18.1071(2)	13.1344(2)	20.5402(5)
α/ °	90	94.441(2)	96.101(2)
β/ °	97.893(1)	101.539(2)	95.551(2)
γ/ °	90	106.832(2)	96.204(2)
U/ Å^3	4922.91(8)	1724.92(6)	5149.2(2)
Z	4	1	2
ρ_{calc}/ g cm^{-3}	1.110	1.105	1.096
μ/ mm^{-1}	1.834	1.765	1.972
F(000)	1784.0	630.0	1860.0
Crystal size/ mm^3	0.301 × 0.097 × 0.089	0.14 × 0.11 × 0.07	0.14 × 0.06 × 0.03
2Θ range for data collection/°	8.222 to 146.53	6.942 to 160.642	4.352 to 148.994
Index ranges	-15 ≤ h ≤ 12<br>-27 ≤ k ≤ 27<br>-22 ≤ l ≤ 22	-13 ≤ h ≤ 12<br>-16 ≤ k ≤ 16<br>-16 ≤ l ≤ 16	-18 ≤ h ≤ 17<br>-14 ≤ k ≤ 20<br>-25 ≤ l ≤ 25
Reflections collected	69427	19600	70682
Independent reflections, R_{int}	9862, 0.0368	7292, 0.0198	20235, 0.1197
Data/restraints/parameters	9862/796/680	7292/0/362	20235/517/1147
Goodness–of–fit on F^2	1.021	1.097	1.021
Final R1, wR2 [I>=2σ(I)]	0.0334, 0.0899	0.0355, 0.0984	0.0644, 0.1624
Final R1, wR2 [all data]	0.0369, 0.0933	0.0382, 0.1000	0.1323, 0.1931
Largest diff. peak/hole/ e Å^{-3}	0.40/-0.39	0.32/-0.23	0.36/-0.68

Computational details

DFT calculations were performed with Gaussian 16 (C.01).[11] Initial BP86[12] optimizations were carried out using the 'grid = ultrafine' option with a split-valence polarised basis set, BS1 = def2-SVP, for all the atoms (H, C, N, O, Ca and Zn atoms).[13] Dispersion corrections employed Grimme's D3 parameter set with Becke-Johnson damping (-D3^{BJ}),[14] alongside corrections for the effect of toluene (ε = 2.3741) solvent introduced using the SMD continuum universal solvation model.[5] All energies were recomputed with a larger basis set, the triple-zeta valence with double polarisation, BS2 = def2-TZVPP, for all atoms.[13] Frequency calculations characterised the stationary points, either as minima or transitions states, with all eigenvalues of the Hessian matrix real and positive (minima) , or only one negative eigenvalue (transition states). Connections between the transition states and the corresponding minima were checked via IRC (Intrinsic Reaction Coordinate) calculations displaced in both directions. A modified version of PyMOL ball-and-stick style provided by the Paton group was used to generate computed structures.[6] This computational methodology is the same as our previously published work on a Ca-Zn system.[17]

Breakdown of Energy Contributions

Table S2. Contribution details for the relative energies of the successive corrections to the initial SCF energy. Terms used are:

$\mathbf{\Delta E_{BS1}}$	SCF energy computed with the BP86 functional and BS1
$\mathbf{\Delta G_{BS1}}$	Free energy at 298.15 K and 1 atm with the BP86 functional and BS1
$\mathbf{\Delta G_{BS1/C_7H_8}}$	Free energy corrected for toluene solvent (SMD) with the BP86 functional and BS1
$\mathbf{\Delta G_{BS1/D3^{BJ}/C_7H_8}}$	Free energy corrected for toluene solvent (SMD) and dispersion (-D3^{BJ}) with the BP86 functional and BS1
$\mathbf{\Delta E_{BS2}}$	SCF energy computed with the BP86 functional and BS2
$\mathbf{\Delta G_{tol}}$	Overall Free energy computed at the BP86-D3^{BJ}; SMD=toluene/BS2//BP86/BS1 level

Table S2 (cont.).

	ΔE_{BS1}	ΔG_{BS1}	$\Delta G_{BS1/C_7H_8}$	$\Delta G_{BS1/D3^{BJ}/C_7H}$	ΔE_{BS2}	ΔG_{tol}
**1**(+Et_2O)	0.0	0.0	0.0	0.0	0.0	**0.0**
**A**(+toluene)	−6.8	−7.3	−8.1	−5.2	−4.0	**−2.4**
**TS(A-B)**	18.4	20.5	21.5	19.5	20.5	**21.6**
**B**	17.7	20.1	20.7	19.8	19.9	**21.9**
**TS(B-C)**	29.5	30.9	34.9	31.1	35.1	**36.7**
**C**	−22.5	−30.9	−30.7	−15.5	−20.6	**−13.6**
**D**(+^tBuH)	−22.5	−34.0	−33.7	−18.3	−20.6	**−16.5**
**TS(D-E)**	−15.5	−28.5	−26.2	−12.0	−14.3	**−10.8**
**E**	−38.7	−54.5	−50.9	−34.5	−37.9	**−33.7**
**TS(E-F)**	−38.4	−55.3	−53.3	−33.1	−38.3	**−33.1**
**F**	−38.4	−57.7	−55.9	−34.6	−38.4	**−34.6**
**G**(+C_2H_4)	−38.2	−64.8	−63.7	−35.7	−39.8	**−37.2**
**TS(G-H)**	−4.2	−28.7	−32.9	−9.1	−5.9	**−10.8**
**H**	−6.4	−31.9	−33.0	−12.4	−5.9	**−11.9**
**TS(H-I)**	13.4	−14.5	−11.3	10.4	13.7	**8.3**
**I**	10.6	−19.9	−17.9	4.1	8.0	**−1.1**
**J**	10.2	−20.0	−20.0	5.8	5.5	**1.6**
**TS(J-K)**	10.6	−21.2	−20.2	7.4	5.1	**0.4**
**K**	−5.4	−49.0	−46.6	−17.1	−13.9	**−25.6**
**L**(+H_2)	−5.7	−47.3	−45.4	−15.3	−14.3	**−23.9**
**TS**(L-M)	−2.1	−48.4	−49.1	−12.1	−11.7	**−21.6**
**M**	−2.1	−50.7	−51.4	−13.7	−11.8	**−23.4**
**N**(+C_2H_4)	−2.0	−54.6	−55.5	−16.1	−12.1	**−26.2**
**O**(+Zn)	12.1	−48.1	−48.8	−7.2	−5.1	**−24.5**
**2**	0.1	−45.8	−39.5	−23.5	−14.7	**−38.3**
**TS(A-P)**	26.2	26.8	29.3	27.7	31.7	**33.2**
**P**	−21.0	−29.8	−30.9	−12.1	−19.4	**−10.5**
**TS(A-C)**	31.3	31.1	33.0	33.8	35.9	**38.4**
**TS(D-Q)**	15.4	2.3	2.7	17.2	16.6	**18.4**
**Q**	10.0	−8.2	−8.7	12.5	8.6	**11.1**
**TS(Q-R)**	10.2	−9.9	−11.2	11.4	8.6	**9.8**
**R**(+H_2)	8.7	−17.3	−18.6	6.2	6.9	**4.3**
**TS(R-S)**	9.3	−16.6	−17.5	6.6	7.0	**4.4**
**S**	−8.1	−33.1	−29.2	−16.6	−9.9	**−18.3**
**TS(S-T)**	−6.1	−34.1	−31.3	−15.4	−8.3	**−17.6**
**T**	−6.4	−35.4	−33.8	−13.6	−9.1	**−16.3**
**U**(+C_2H_4)	−6.7	−43.4	−42.0	−16.5	−10.5	**−20.3**
**TS(U-L)**	15.0	−26.2	−26.1	4.6	9.6	**−0.8**
**TS(G-V)**	19.0	−8.9	−8.1	13.3	17.0	**11.2**
**V**	8.8	−31.3	−31.2	−1.0	3.4	**−6.4**
**W**(+H_2)	8.9	−30.8	−30.8	−0.5	3.4	**−6.0**
**TS(W-L)**	9.0	−30.3	−29.5	0.6	3.0	**−5.4**
**TS(G-X)**	−18.8	−46.0	−47.3	−26.7	−20.6	**−28.5**
**X**	−23.6	−47.4	−46.5	−26.5	−25.0	**−27.9**
**TS(X-V)**	23.2	−5.3	−4.6	21.4	21.3	**19.5**

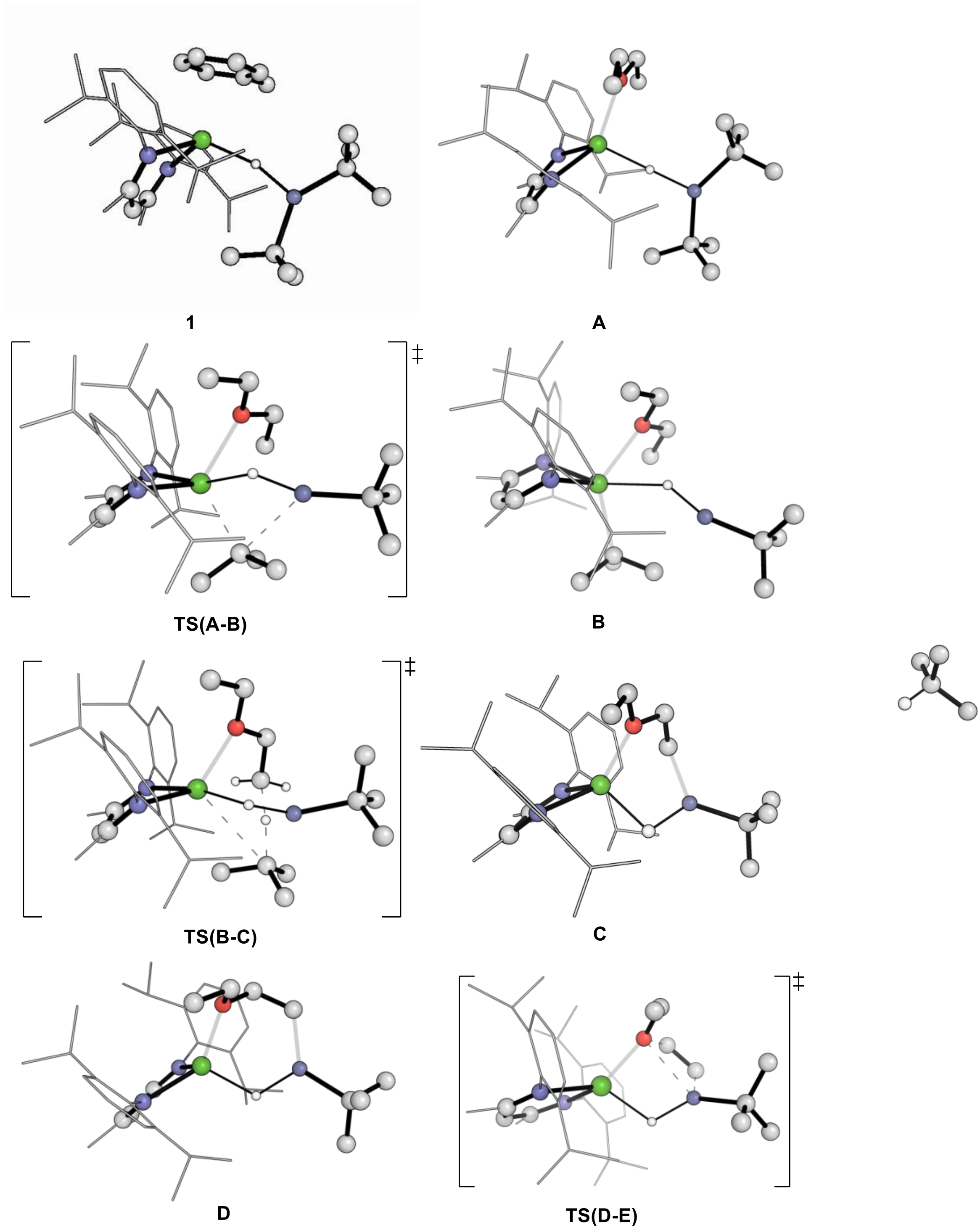

Figure 34. BP86/BS1 Optimised geometries. For clarity, hydrogen atoms from BDI ligand are omitted, and the Dipp substituents are shown in wireframe representation.

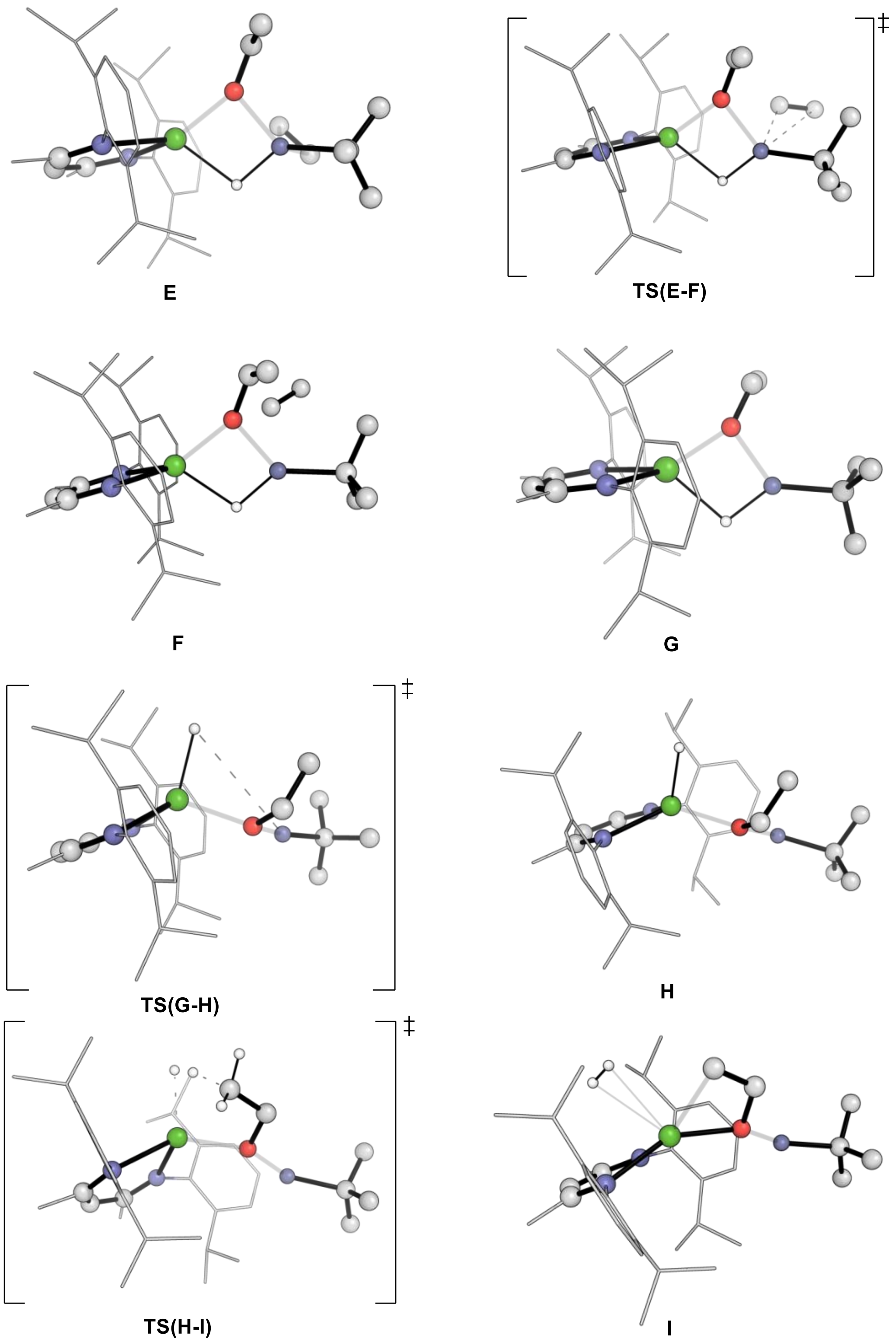

Figure S35. BP86/BS1 Optimised geometries. For clarity, hydrogen atoms from BDI ligand are omitted, and the Dipp substituents are shown in wireframe representation.

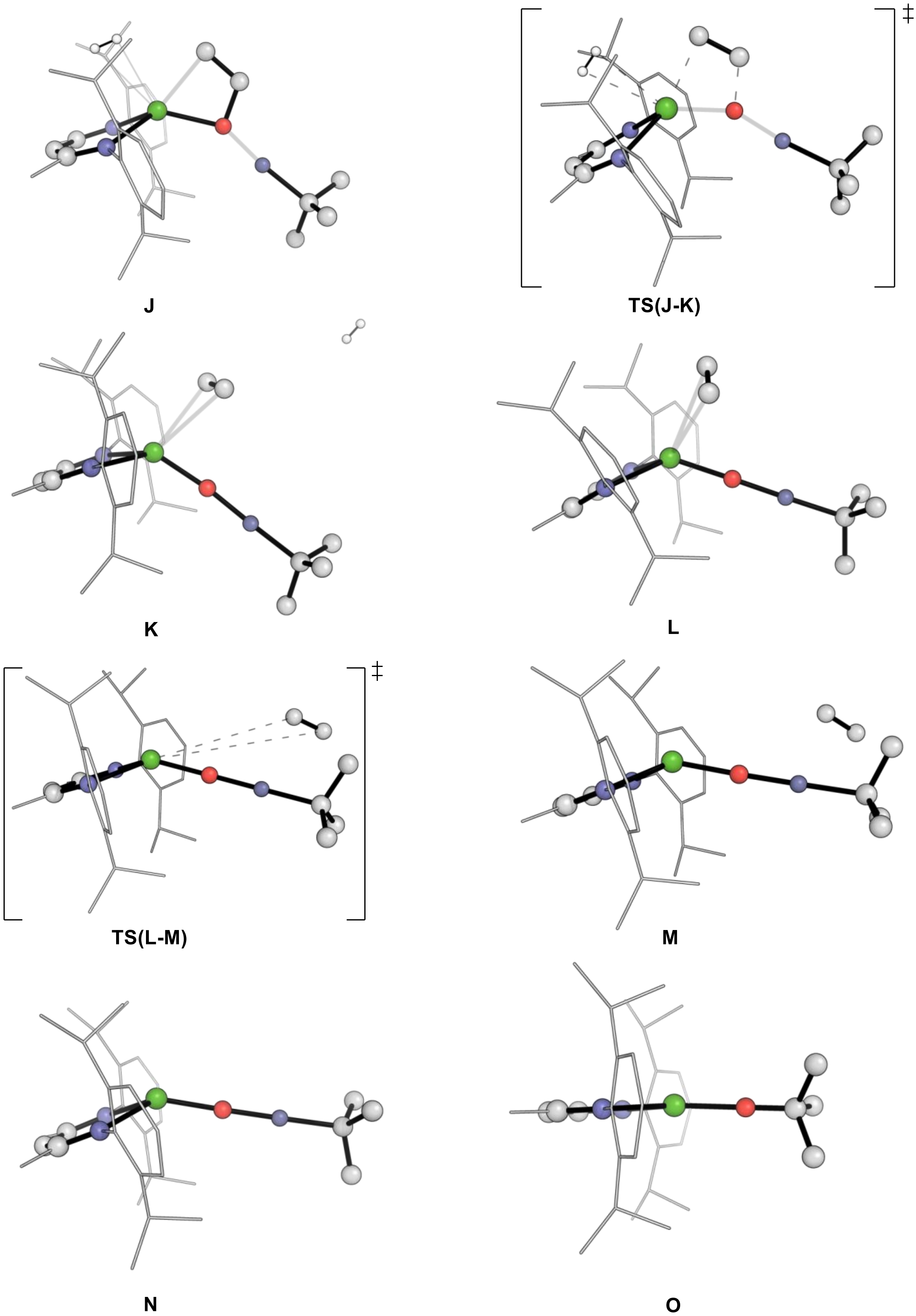

Figure S36. BP86/BS1 Optimised geometries. For clarity, hydrogen atoms from BDI ligand are omitted, and the Dipp substituents are shown in wireframe representation.

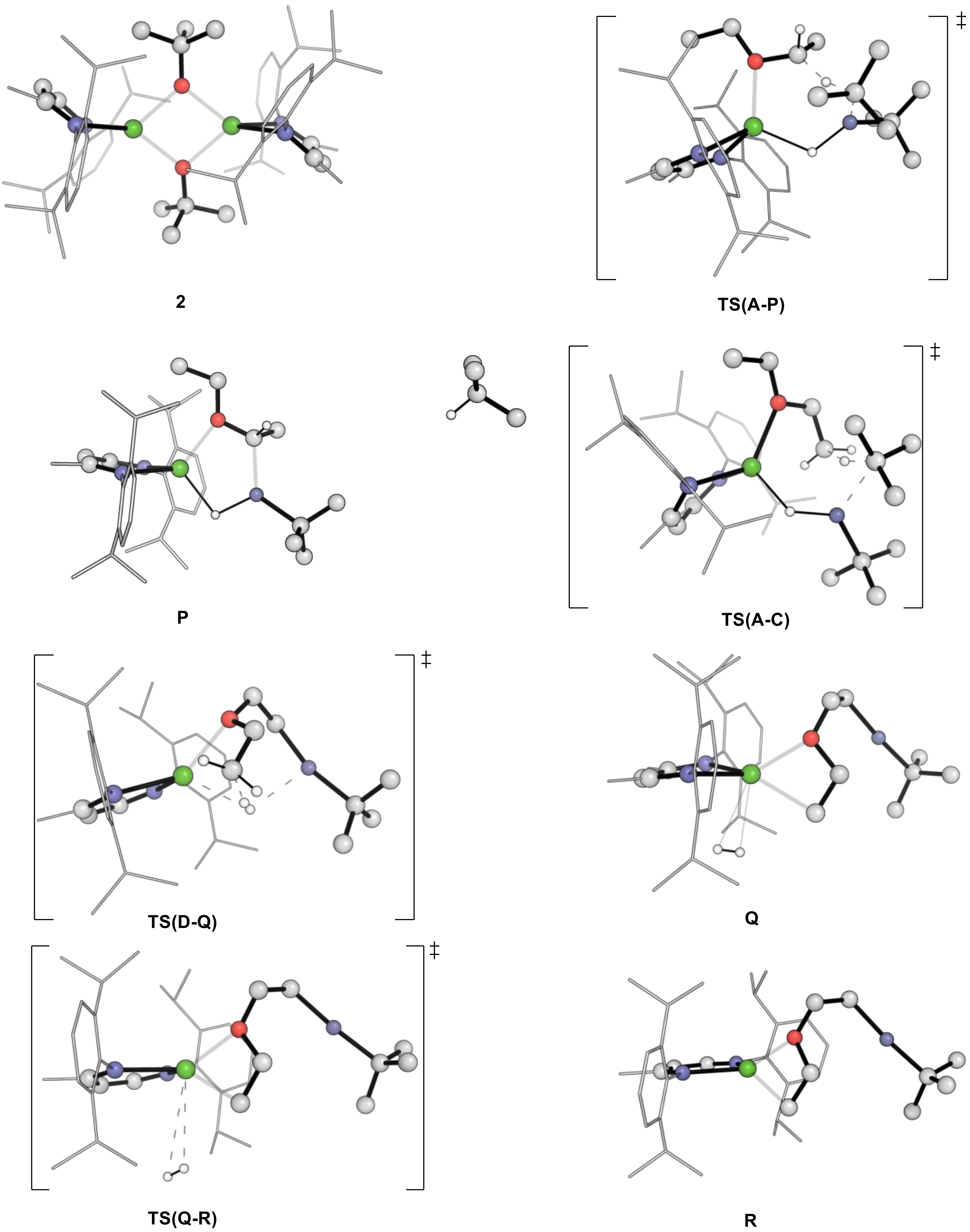

Figure S37. BP86/BS1 Optimised geometries. For clarity, hydrogen atoms from BDI ligand are omitted, and the Dipp substituents are shown in wireframe representation.

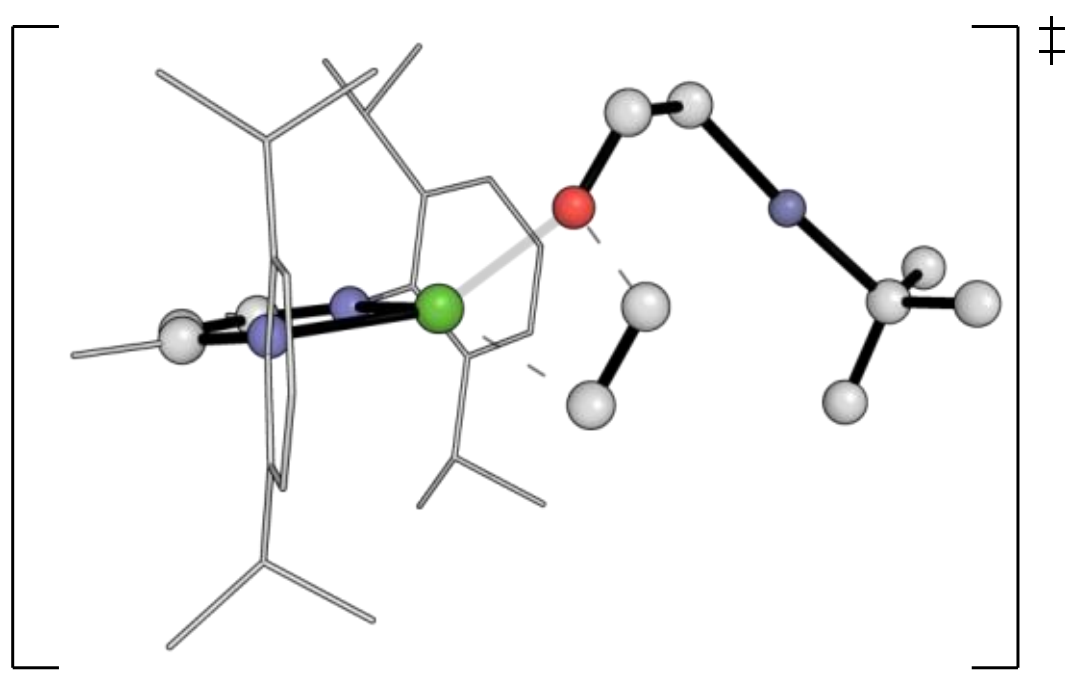

TS(R-S)

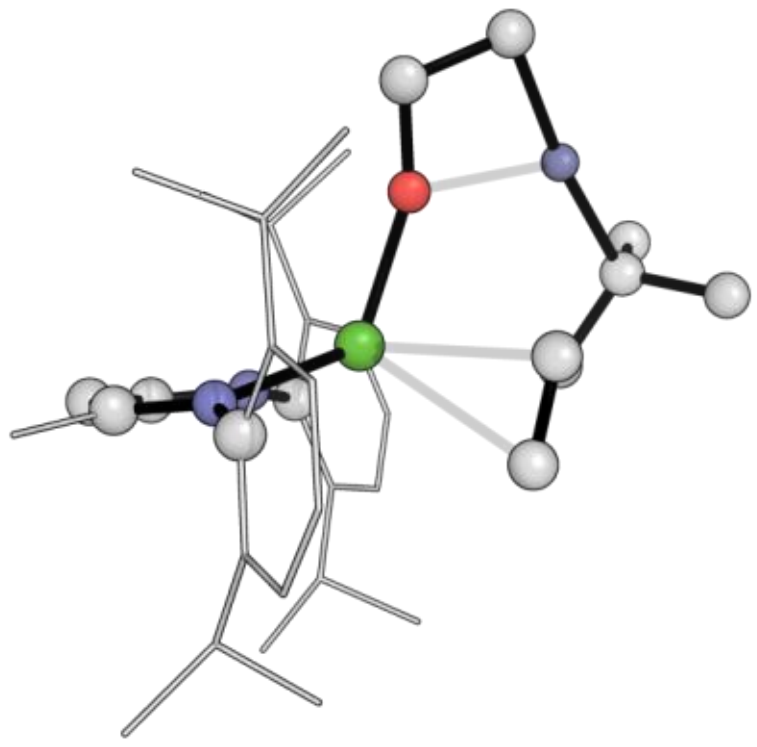

S

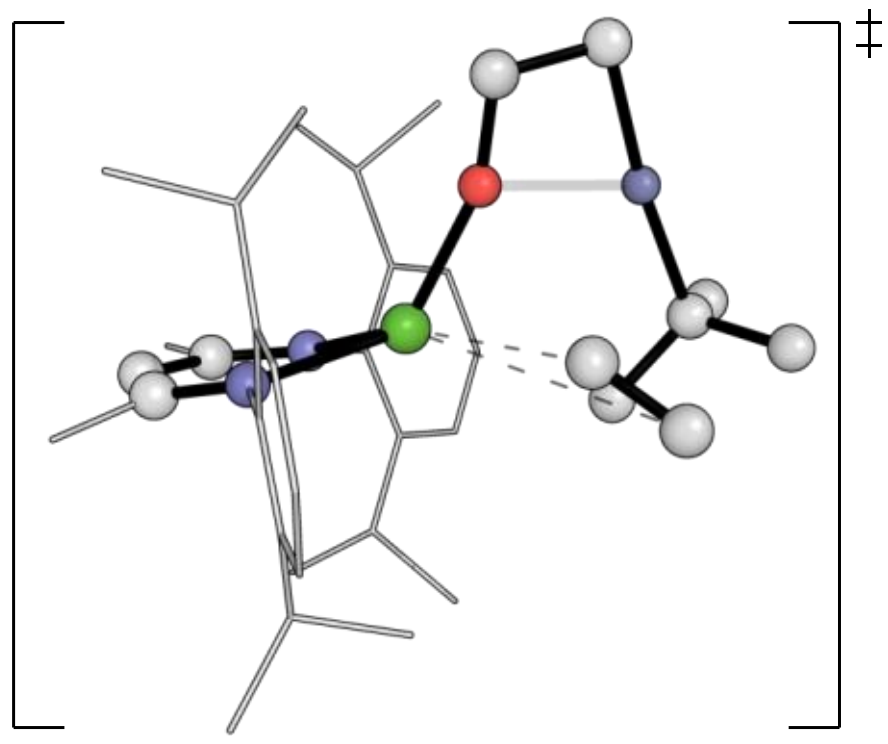

TS(S-T)

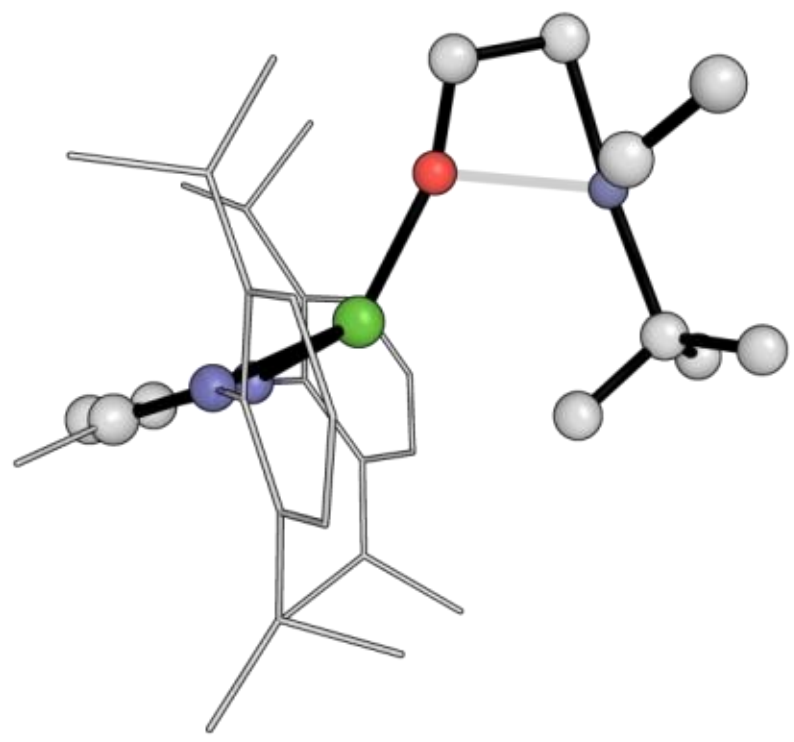

T

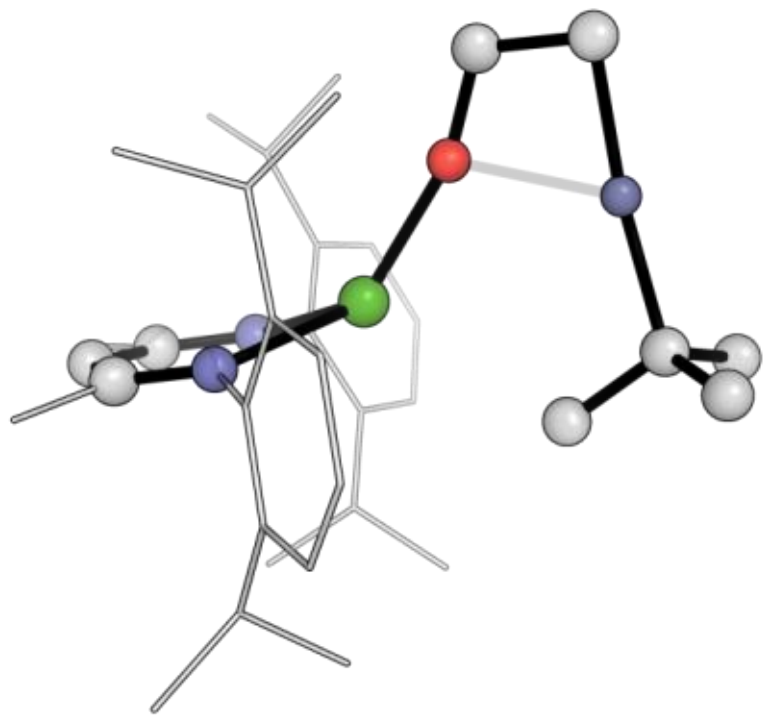

U

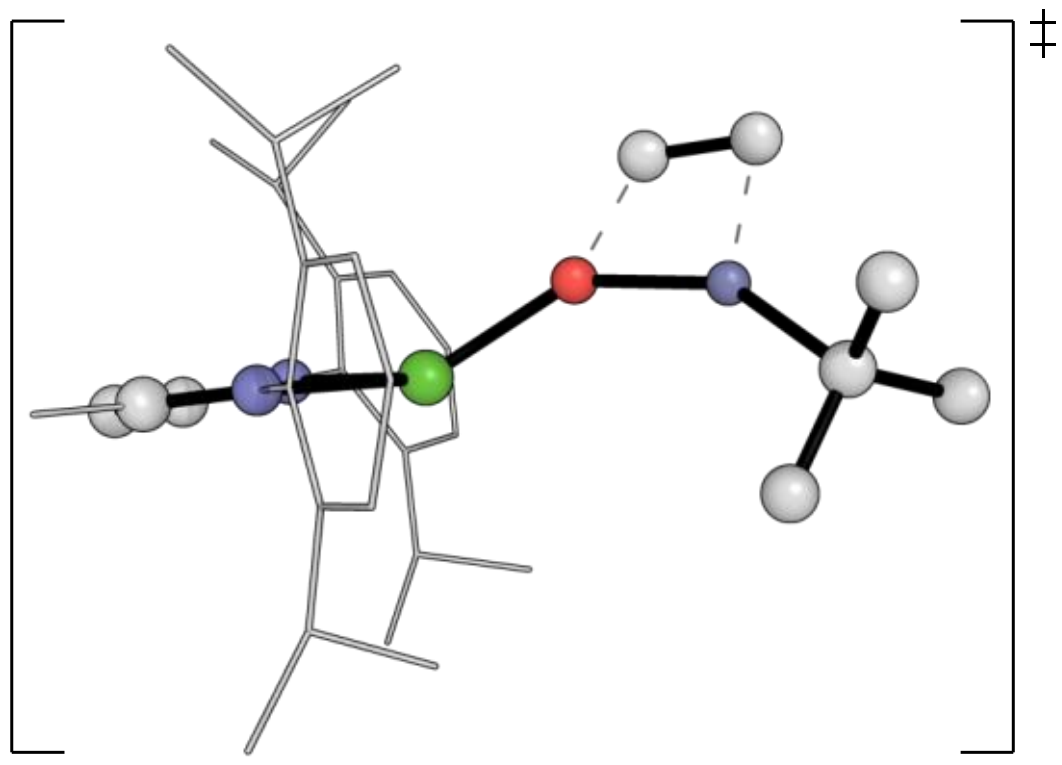

TS(U-L)

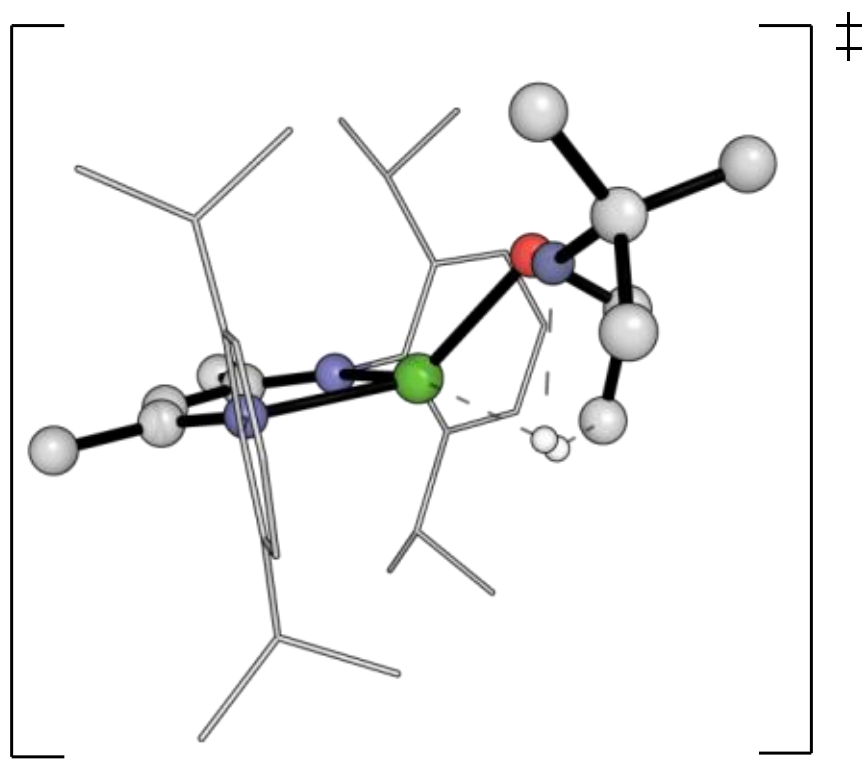

TS(G-V)

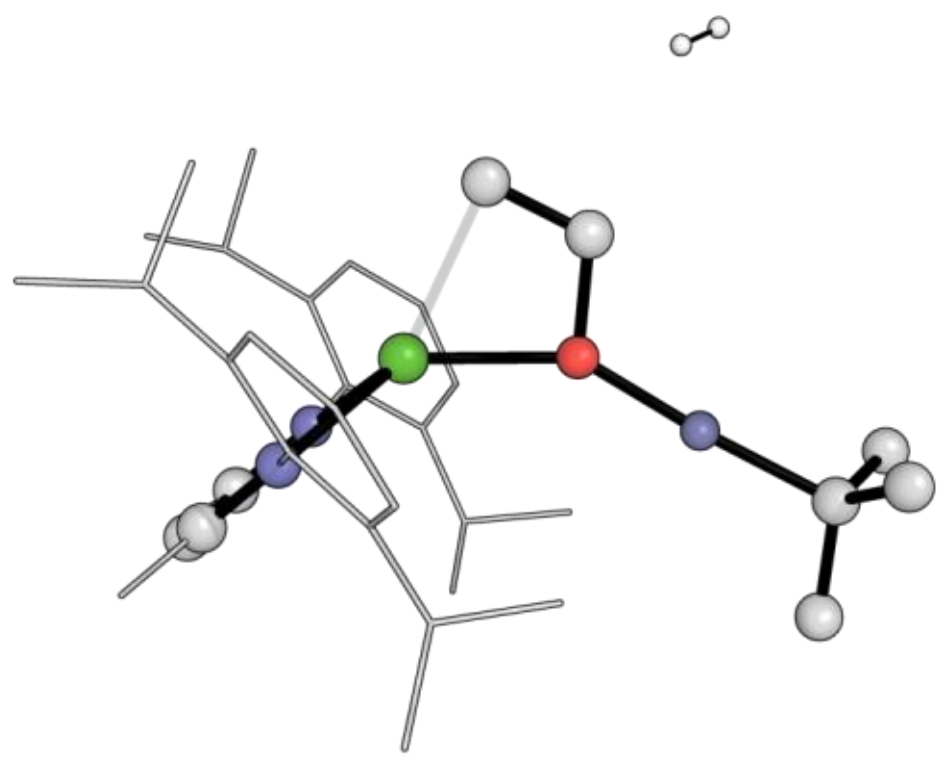

V

Figure S38. BP86/BS1 Optimised geometries. For clarity, hydrogen atoms from BDI ligand are omitted, and the Dipp substituents are shown in wireframe representation.

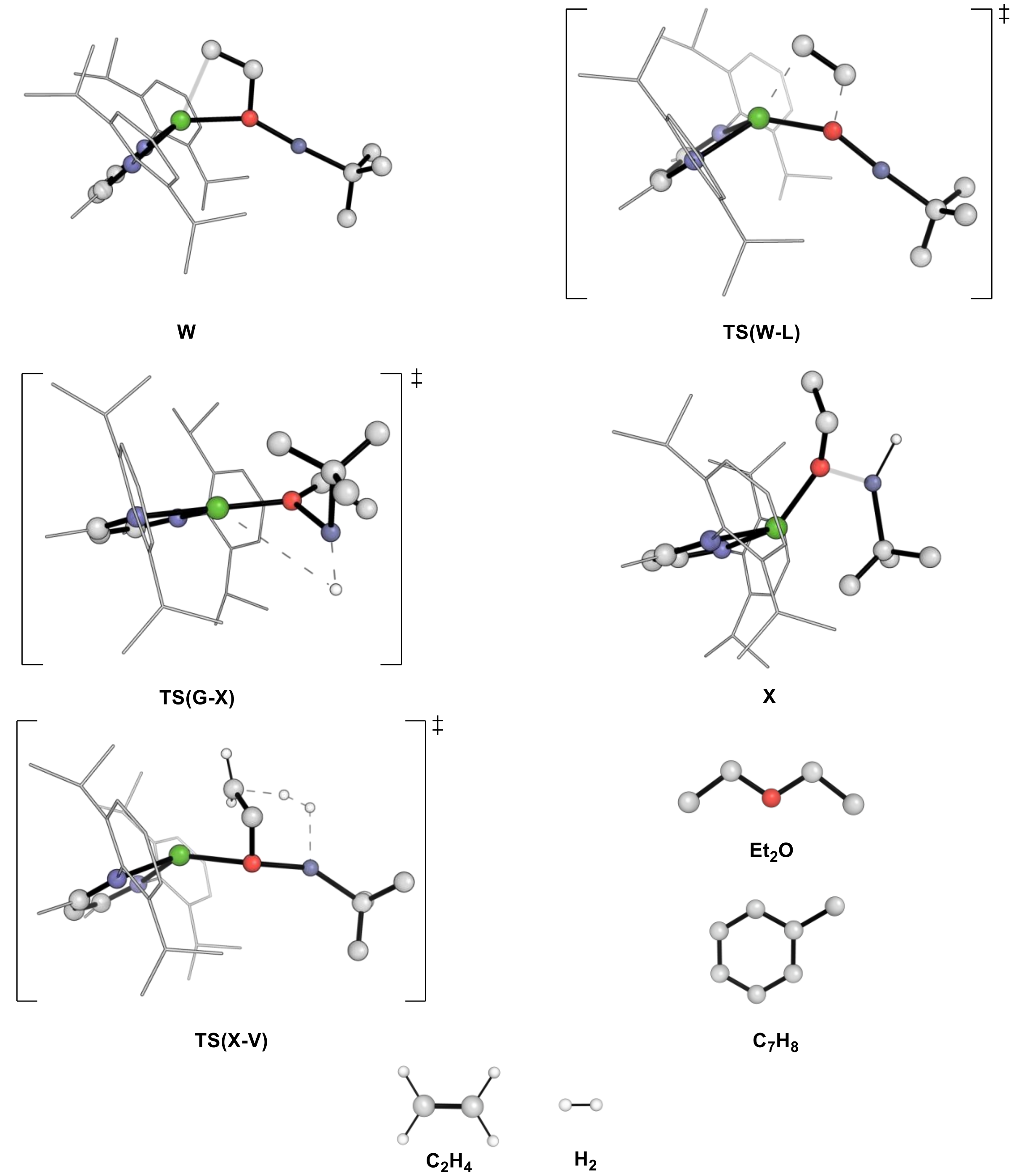

Figure S39. BP86/BS1 Optimised geometries. For clarity, hydrogen atoms from BDI ligand are omitted, and the Dipp substituents are shown in wireframe representation.

Alternative pathways investigated

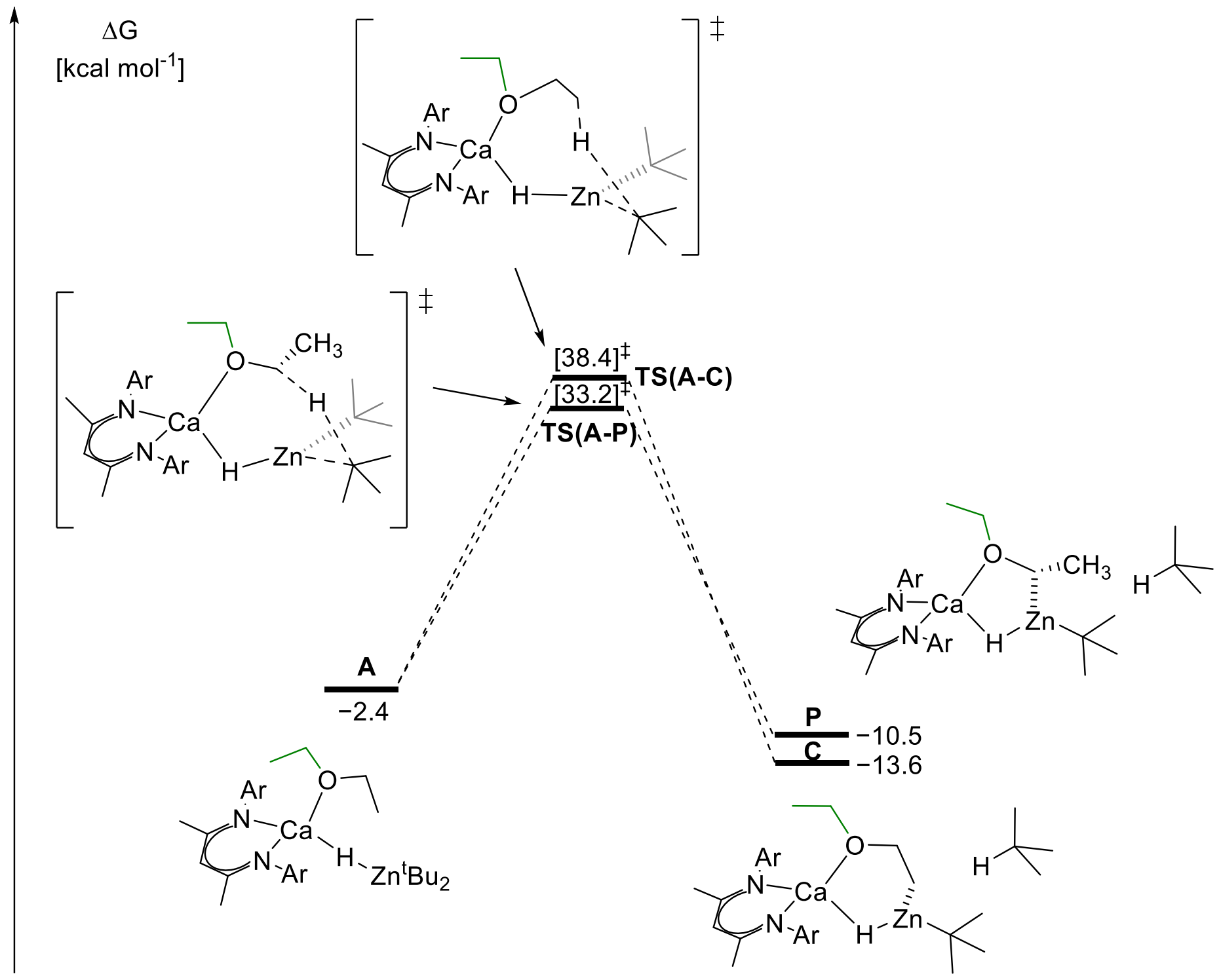

Figure S40. Computed reaction profile (BP86-D3[BJ]($SMD=C_7H_8$)/def2-TZVPP//BP86/def2-SVP in kcal mol^{-1}) for one-fold deprotonation of coordinated ether by zinc-bound alkyl in intermediate **A**.

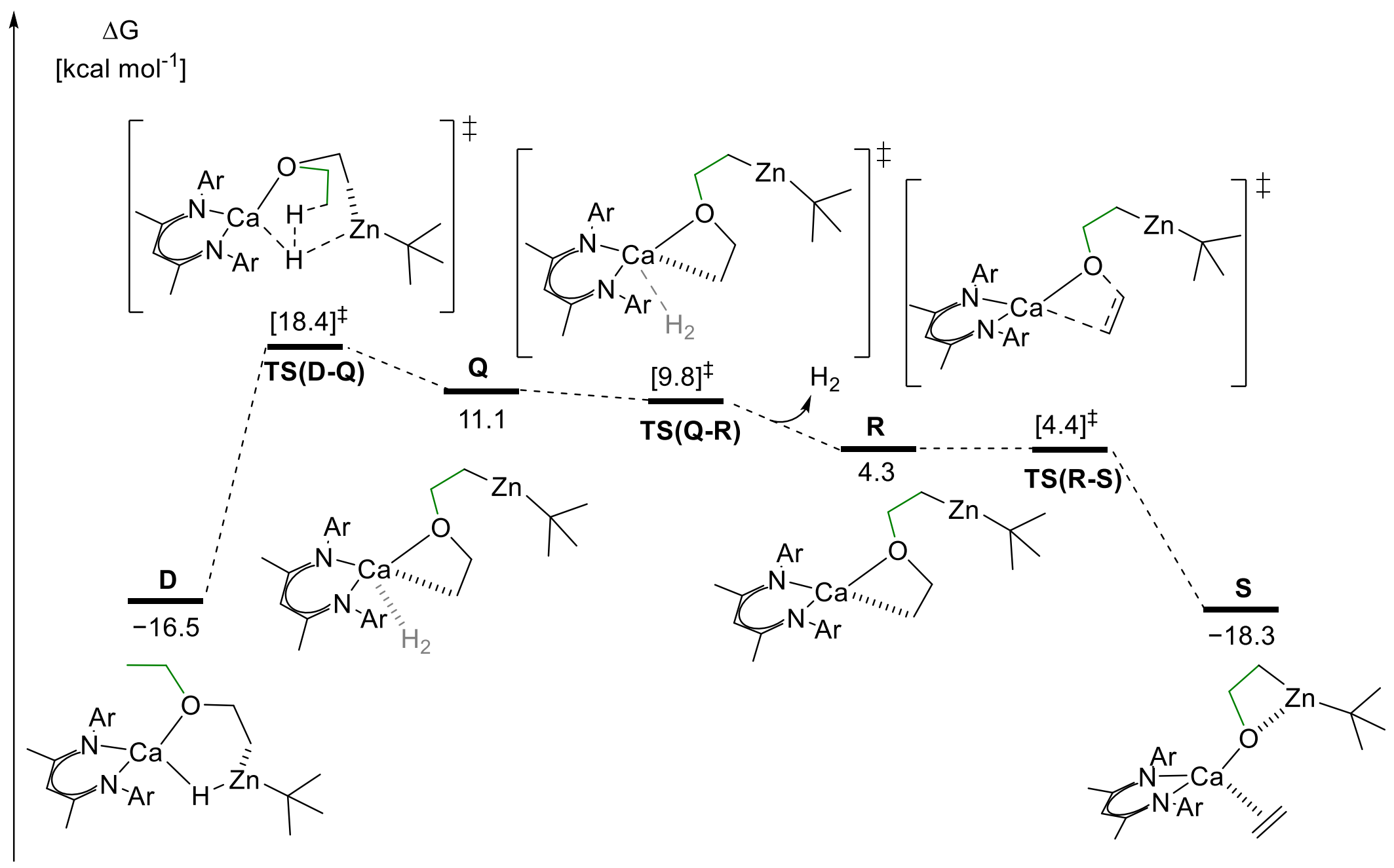

Figure S41. Computed reaction profile (BP86-D3[BJ]($SMD=C_7H_8$)/def2-TZVPP//BP86/def2-SVP in kcal mol^{-1}) for second deprotonation from the bridging hydride in intermediate **D** resulting in dihydrogen release and ethylene extrusion.

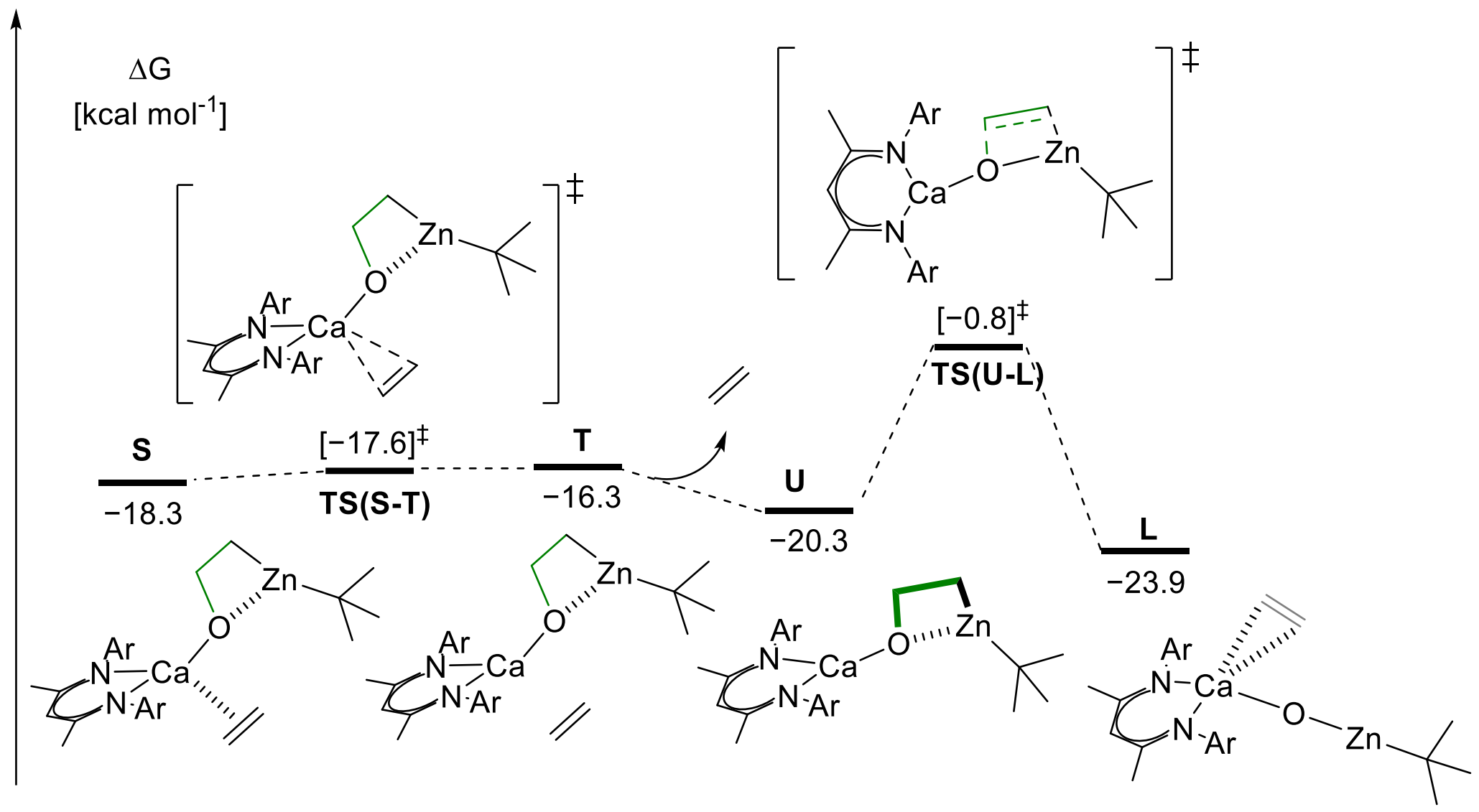

Figure S42. Computed reaction profile (BP86-D3[BJ](SMD=C_7H_8)/def2-TZVPP//BP86/def2-SVP in kcal mol^{-1}) for ethylene release from **S**.

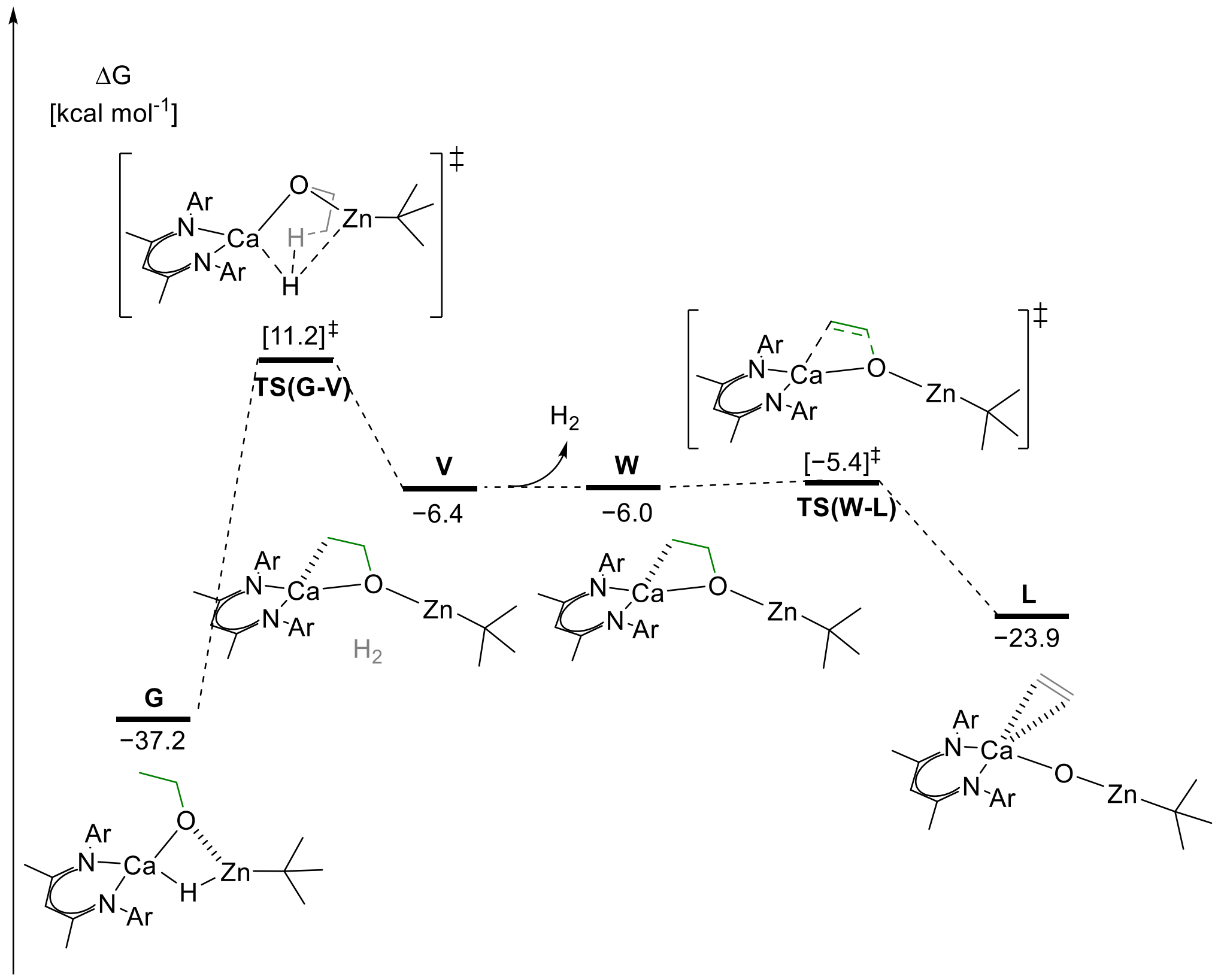

Figure S43. Computed reaction profile (BP86-D3[BJ](SMD=C_7H_8)/def2-TZVPP//BP86/def2-SVP in kcal mol^{-1}) for dihydrogen formation from bridging hydride and ethylene extrusion.

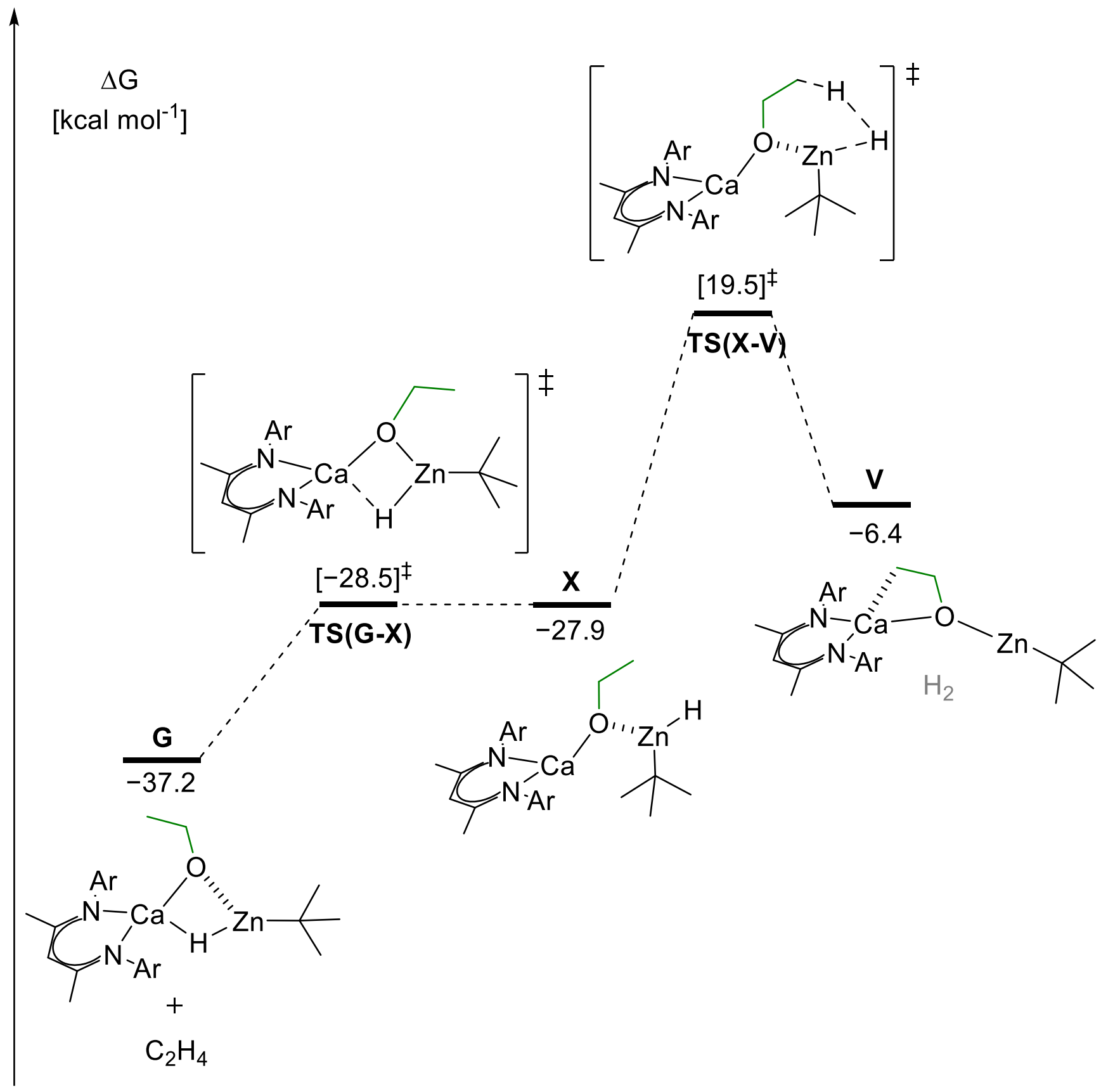

Figure S44. Computed reaction profile (BP86-D3[BJ](SMD=C_7H_8)/def2-TZVPP//BP86/def2-SVP in kcal mol^{-1}) for two-fold deprotonation from the **G** intermediate: cleavage of Ca-H bond and deprotonation at β-position of ethoxide.

Construction of a Combined Experimental and Computational Hess Cycle: Zinc deposition from intermediate N

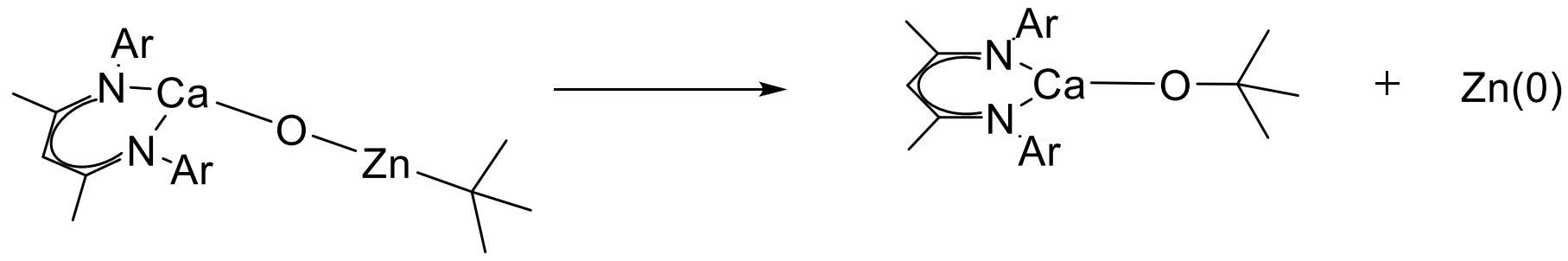

N O

Figure S45. Zn deposition from intermediate **N**.

To construct the Hess cycle for the Zn deposition (see Figure S40 above), three thermodynamic functions were calculated:

1. Computed Gibbs energy of the reaction in solution ($\Delta G_{\text{rxn (sol.)}}$)

2. Experimental Gibbs atomisation energy (ΔG_{at}) of Zn(0) atoms

3. Computed Gibbs solvation energy (ΔG_{solv}) of Zn(0) atoms

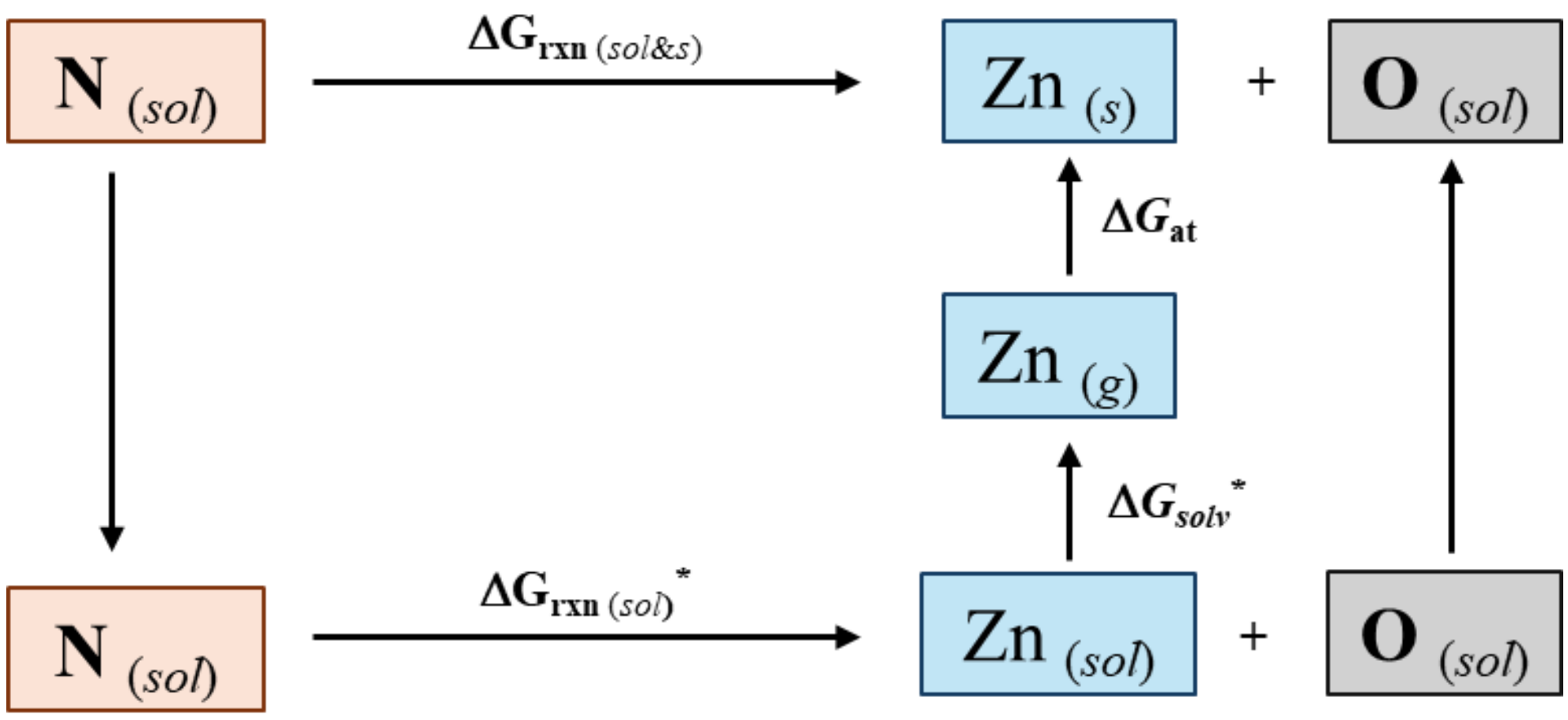

$$\Delta G_{\text{rxn }(sol\&s)} = \Delta G_{\text{rxn (sol)}} - \Delta G_{\text{at}}[\text{Zn}^0] - \Delta G_{\text{solv}}[\text{Zn}^0] \quad (\text{Eq. 1})$$

Figure S46. Combined computational Hess cycle with experimental data to calculate the reaction Gibbs energy, $\Delta G_{\text{rxn }(sol\&s)}$ (* = computed values).

Table S3. Gibbs energy (in kcal mol^{-1}) for the Zn deposition from intermediate **N** in solution and using a combined experimental and computational Hess cycle. The energies are referenced respect to **1**. Bold value is used on the main text.

**ΔG (*sol*)** / kcal mol^{-1}	**ΔG (*sol&s*)** / kcal mol^{-1}
−24.5	**−43.5**

Computational identification of species **N** as a key intermediate *en route* to compound **2** prompted synthetic consideration of its rational synthesis by reaction of *t*-Bu_2Zn with the putative calcium hydroxide complex, $[(BDI)CaOH]_2$. Attempts to synthesise this latter species, however, either by oxygen insertion by N_2O or selective protonolysis with water have been unsuccessful.[19]

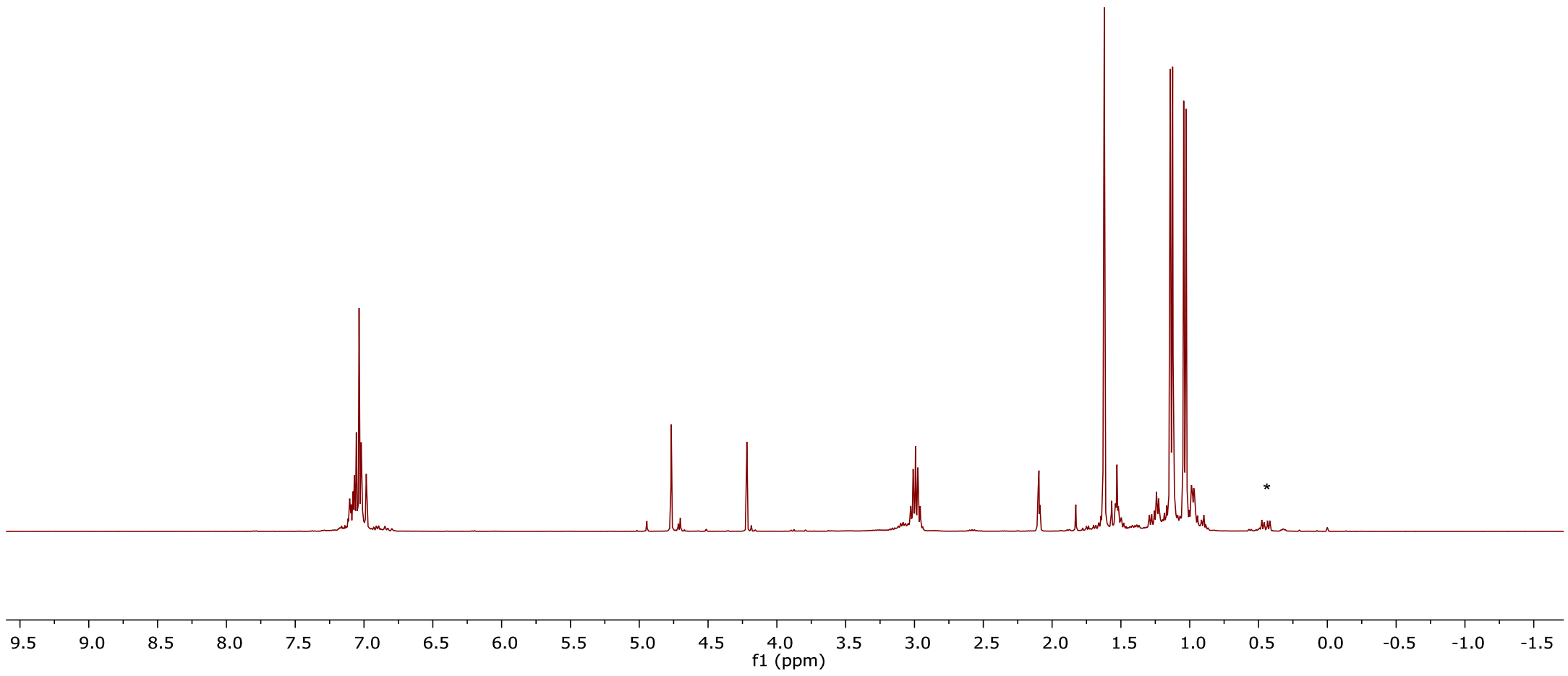

Figure S47. *^{1}H NMR Spectrum (C_7D_8, 298 K, 400.13 MHz) from the reaction of $[(BDI)CaH]_2$ and N_2O. * = homoleptic calcium complex, $[(BDI)_2Ca]$. The major signals are still starting material.*

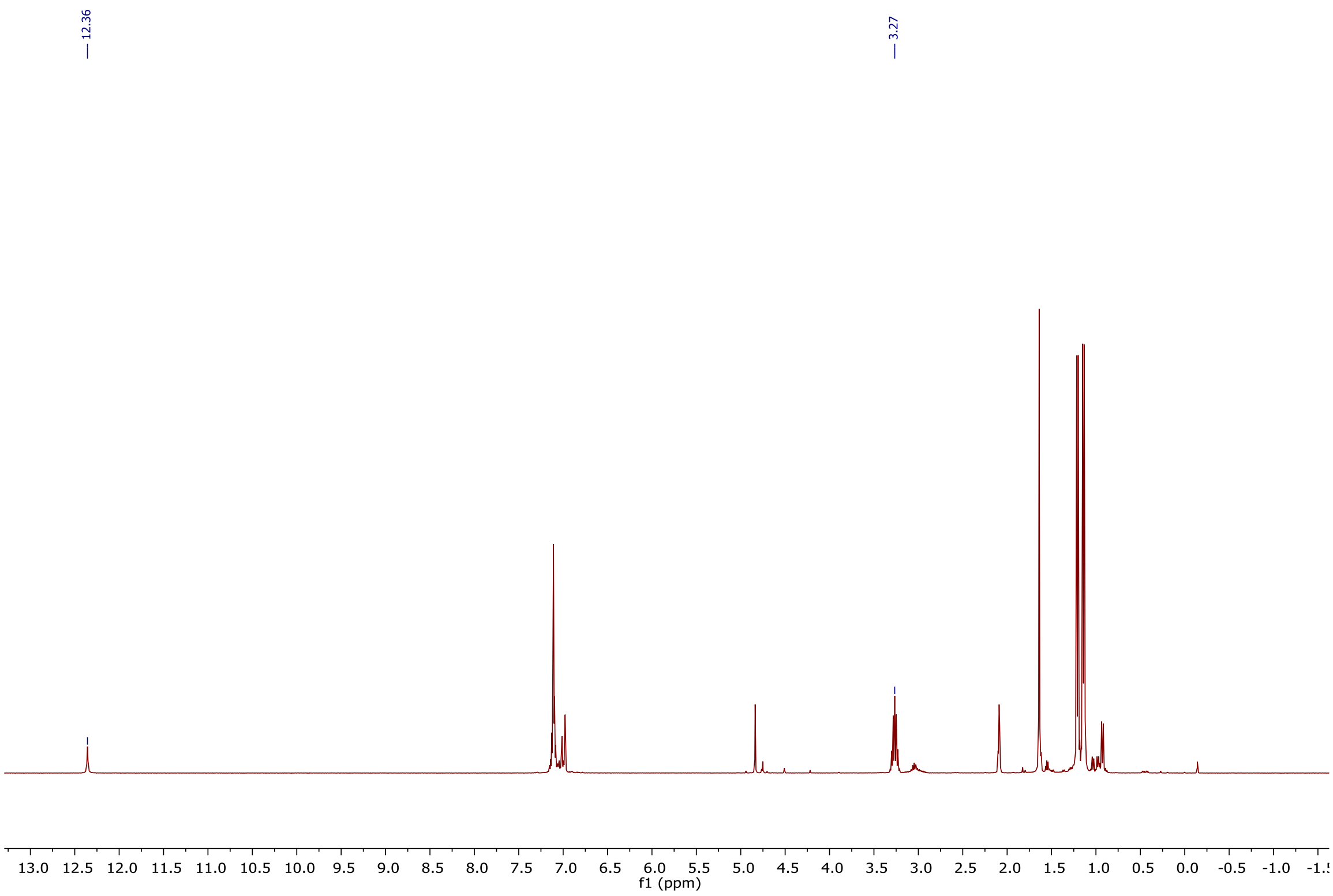

Figure S48. *^{1}H NMR Spectrum (C_7D_8, 298 K, 400.13 MHz) from the reaction of $[(BDI)CaH]_2$ and $CuSO_4.5H_2O$, demonstrating the formation of protonated NacNac.*

References

1. A. S. S. Wilson, M. S. Hill, M. F. Mahon, C. Dinoi, L. Maron, *Science*, **2017**, *358*, 1168-1171.
2. E. Hevia, G. W. Honeyman, A. R. Kennedy and R. E. Mulvey, *J. Am. Chem. Soc.*, **2005**, *127*, 13106-13107.
3. D. Marković, C. M. R. Volla, P. Vogel, A. Varela-Álvarez and J. A. Sordo, *Chem. Eur. J.*, **2010**, *16*, 5969-5975.
4. H. A. Olszowy and W. Kitching, *Organometallics*, **1984**, *3*, 1676-1683.
5. D. R. Lane, C. M. Beavers, M. M. Olmstead and N. E. Schore, *Organometallics*, **2009**, *28*, 6789-6797.
6. J. J. Eisch and S. Dutta, *Organometallics*, **2005**, *24*, 3355-3358.
7. H. Lehmkuhl and O. Olbrysch, *Liebigs Ann. Chem.*, **1975**, 1162-1175.
8. O. V. Dolomanov, L. J. Bourhis, R. J. Gildea, J. A. K. Howard and H. Puschmann, *J. Appl. Cryst.,* **2009**, *42*, 339-341.
9. G. M. Sheldrick, *Acta Cryst.,* **2015**, *A71*, 3-8.
10. G. M. Sheldrick, *Acta Cryst.,* **2015**, *C71*, 3-8.
11. Gaussian 16, Revision C.01, M. J. Frisch, G. W. Trucks, H. B. Schlegel, G. E. Scuseria, M. A. Robb, J. R. Cheeseman, G. Scalmani, V. Barone, G. A. Petersson, H. Nakatsuji, X. Li, M. Caricato, A. V. Marenich, J. Bloino, B. G. Janesko, R. Gomperts, B. Mennucci, H. P. Hratchian, J. V. Ortiz, A. F. Izmaylov, J. L. Sonnenberg, D. Williams-Young, F. Ding, F. Lipparini, F. Egidi, J. Goings, B. Peng, A. Petrone, T. Henderson, D. Ranasinghe, V. G. Zakrzewski, J. Gao, N. Rega, G. Zheng, W. Liang, M. Hada, M. Ehara, K. Toyota, R. Fukuda, J. Hasegawa, M. Ishida, T. Nakajima, Y. Honda, O. Kitao, H. Nakai, T. Vreven, K. Throssell, J. A. Montgomery, Jr., J. E. Peralta, F. Ogliaro, M. J. Bearpark, J. J. Heyd, E. N. Brothers, K. N. Kudin, V. N. Staroverov, T. A. Keith, R. Kobayashi, J. Normand, K. Raghavachari, A. P. Rendell, J. C. Burant, S. S. Iyengar, J. Tomasi, M. Cossi, J. M. Millam, M. Klene, C. Adamo, R. Cammi, J. W. Ochterski, R. L. Martin, K. Morokuma, O. Farkas, J. B. Foresman, and D. J. Fox, Gaussian, Inc., Wallingford CT, 2016.
12. (a) A. D. Becke, *Phys. Rev. A* **1988**, *38*, 3098-3100; (b) J. P. Perdew, *Phys. Rev. B* **1986**, *33*, 8822- 8824.
13. F. Weigend, R. Ahlrichs, *Phys. Chem. Chem. Phys.* **2005**, *7*, 3297-3305.
14. S. Grimme, S. Ehrlich, L. Goerigk, *J. Comp. Chem.* **2011**, *32*, 1456-1465.
15. J. Tomasi, B. Mennucci, R. Cammi, *Chem. Rev.* **2005**, *105*, 2999-3094.
16. R. Paton. Ball & Stick style for Pymol. Zenodo. **2022**. https://doi.org/10.5281/zenodo.7072564
17. K. G. Pearce, A. Morales, M. S. Hill, M. F. Mahon, C. L. McMullin, *J. Am. Chem. Soc.* **2025**, *147*, 29554-29567.
18. Gas-phase electronic energies are consistent; however, inclusion of the energy corrections places intermediate **B** higher in Gibbs energy than **TS(A–B)**, whereas at the BP86/BS1 level it remains lower (exergonic) relative to the transition state (see Table S2).
19. The sole example of a molecular calcium hydroxide is provided by $[(BDI)Ca(OH)(THF)]_2$, which is presumably much less labile than the current system due to the presence of the coordinated cyclic ether. See, C. Ruspic, S. Nembenna, A. Hofmeister, J. Magull, S. Harder, H. W. Roesky, *J. Am. Chem. Soc.* **2006**, 128, 15000–15004.

XYZ coordinates and raw energies

1

SCF (BP86) Energy = -4282.823187
Enthalpy 0K = -4281.846968
Enthalpy 298K = -4281.783828
Free Energy 298K = -4281.948401
Lowest Frequency = 15.5786 cm^{-1}
Second Frequency = 23.5907 cm^{-1}
SCF (BP86-D3^{BJ}) Energy = -4283.117053
SCF ($C_6H_5CH_3$) Energy = -4282.871097
SCF (BS2) Energy = -4285.135356

Zn	-3.58905	0.12872	-0.07028
H	-1.88038	0.02441	0.43148
Ca	0.23107	0.02015	0.43116
N	1.28633	-1.48187	-1.02705
N	1.33693	1.63481	-0.85794
C	1.42711	-1.15399	-2.32405
C	1.42004	0.17639	-2.82775
H	1.48317	0.23409	-3.92442
C	1.45727	1.44445	-2.18500
C	1.57190	-2.24477	-3.37806
H	1.97594	-3.18419	-2.95502
H	2.21662	-1.91642	-4.21627
H	0.57274	-2.47769	-3.80651
C	1.60671	2.64145	-3.11563
H	0.59979	2.96976	-3.45455
H	2.18896	2.38446	-4.02142
H	2.07956	3.50610	-2.61197
C	1.48861	-2.81427	-0.55337
C	2.77089	-3.16878	-0.01371
C	2.93723	-4.44161	0.56746
H	3.91923	-4.72396	0.97826
C	1.87885	-5.36142	0.63047
H	2.03012	-6.35121	1.08987
C	0.63319	-5.01340	0.09347
H	-0.19312	-5.74217	0.12951
C	0.41139	-3.75481	-0.50740
C	3.95219	-2.19885	-0.10070
H	3.51729	-1.17505	-0.13174
C	4.74299	-2.39275	-1.41499
H	5.17417	-3.41553	-1.46678
H	5.58034	-1.66604	-1.48224
H	4.10309	-2.25108	-2.30713
C	4.91140	-2.27738	1.10272
H	4.38030	-2.19755	2.07424
H	5.65740	-1.45684	1.05458
H	5.48518	-3.22830	1.11813
C	-0.96280	-3.45602	-1.11250
H	-0.91738	-2.44164	-1.56316
C	-1.31634	-4.44850	-2.24240
H	-0.52352	-4.49174	-3.01649
H	-2.26416	-4.15079	-2.73769
H	-1.45551	-5.47856	-1.85027
C	-2.07823	-3.43192	-0.04754
H	-2.17670	-4.41218	0.46583
H	-3.05805	-3.19832	-0.51429
H	-1.89263	-2.65874	0.72642
C	1.60025	2.90258	-0.25322
C	2.91477	3.16597	0.25965
C	3.14583	4.36967	0.95523
H	4.15301	4.58242	1.34671
C	2.12039	5.30567	1.15998
H	2.32229	6.23964	1.70810
C	0.84105	5.04383	0.65497
H	0.03826	5.78360	0.80794
C	0.55489	3.85855	-0.05645
C	4.05964	2.17432	0.03680
H	3.58871	1.17521	-0.09731
C	5.03993	2.08825	1.22324
H	5.65191	3.00937	1.32660
H	5.75131	1.24884	1.07602
H	4.51942	1.93016	2.19094
C	4.83590	2.48645	-1.26313
H	4.17836	2.46305	-2.15339
H	5.64805	1.74612	-1.42492
H	5.29968	3.49493	-1.21144
C	-0.85693	3.65114	-0.60963
H	-0.86155	2.68869	-1.16517
C	-1.24870	4.76088	-1.60995
H	-1.33748	5.74821	-1.10855
H	-2.23047	4.53481	-2.07610
H	-0.49992	4.86587	-2.42123
C	-1.90949	3.53769	0.51229
H	-1.69812	2.68714	1.19375
H	-2.92042	3.36967	0.08533
H	-1.95443	4.46178	1.12722
C	-4.79165	0.05918	1.58972
C	-4.45929	1.22023	2.53958
H	-4.63833	2.21407	2.07234
H	-3.39517	1.20381	2.86798
H	-5.08543	1.18892	3.47134
C	-4.58016	-1.27046	2.33015
H	-3.52172	-1.40663	2.65024
H	-4.84564	-2.15147	1.70435
H	-5.20886	-1.33654	3.25834
C	-6.26708	0.16611	1.16442
H	-6.95809	0.12570	2.04828
H	-6.56944	-0.66074	0.48483
H	-6.48280	1.11966	0.63414
C	-3.80529	0.26516	-2.10629
H	-3.99844	2.47343	-2.12392
C	-4.64276	-0.93167	-2.59251
H	-5.64069	-0.97541	-2.10370
H	-4.82749	-0.88413	-3.69896
H	-1.87013	-0.65890	-2.64162
C	-4.56208	1.56481	-2.43535
H	-4.74502	1.66875	-3.53823
H	-5.55701	1.61082	-1.94073
H	-4.13989	-1.90661	-2.40046
C	-2.45674	0.26698	-2.83669
H	-1.81300	1.12269	-2.53197

H	-2.57805	0.33973	-3.95042
C	0.10246	-1.37909	3.26057
C	-0.60958	-0.16925	3.45760
H	-1.67287	-0.20740	3.74062
C	0.02065	1.08147	3.30034
H	-0.55516	2.00478	3.46767
C	1.37622	1.15443	2.92173
H	1.86686	2.13105	2.78938
C	2.09070	-0.04138	2.69197
H	3.14769	-0.00148	2.38780
C	1.45843	-1.29144	2.85802
H	2.02441	-2.21789	2.66937
C	-0.54882	-2.71829	3.50729
H	-1.64576	-2.67185	3.36010
H	-0.36982	-3.03974	4.55693
H	-0.13351	-3.50384	2.84510

A

SCF (BP86) Energy = -4244.951746
Enthalpy 0K = -4243.967219
Enthalpy 298K = -4243.903493
Free Energy 298K = -4244.070326
Lowest Frequency = 12.8201 cm^{-1}
Second Frequency = 14.2455 cm^{-1}
SCF (BP86-D3^{BJ}) Energy = -4245.232327
SCF ($C_6H_5CH_3$) Energy = -4244.997418
SCF (BS2) Energy = -4247.235095

Zn	3.56429	-0.01533	0.10387
H	1.78963	0.18674	0.17410
Ca	-0.30436	0.03580	0.35912
N	-1.50575	1.55597	-0.99331
N	-1.36307	-1.55655	-1.03077
C	-1.93793	1.29018	-2.23912
C	-1.93622	0.00285	-2.84137
H	-2.24427	0.00271	-3.89608
C	-1.74414	-1.29417	-2.29709
C	-2.51450	2.41690	-3.08321
H	-3.40347	2.85672	-2.58415
H	-2.81626	2.06269	-4.08606
H	-1.78908	3.24648	-3.20295
C	-2.04032	-2.45447	-3.23626
H	-1.19989	-3.17775	-3.24782
H	-2.22531	-2.10544	-4.26897
H	-2.93052	-3.02341	-2.89671
C	-1.65569	2.87215	-0.45279
C	-2.77664	3.15657	0.39265
C	-2.85688	4.41536	1.02339
H	-3.71824	4.63812	1.67465
C	-1.86829	5.39058	0.83391
H	-1.94704	6.36823	1.33543
C	-0.78291	5.11284	-0.00835
H	-0.01125	5.88436	-0.16377
C	-0.65181	3.87191	-0.66627
C	-3.89181	2.13314	0.62435
H	-3.64028	1.23414	0.02321
C	-5.26023	2.65199	0.13271
H	-5.59585	3.53784	0.71302
H	-6.04011	1.86864	0.24052
H	-5.22363	2.94884	-0.93554
C	-3.96958	1.69667	2.10310
H	-3.01475	1.24215	2.44284
H	-4.77254	0.94500	2.25318
H	-4.18705	2.55609	2.77255
C	0.55767	3.64416	-1.57876
H	0.43018	2.65467	-2.06592
C	0.64698	4.70753	-2.69610
H	-0.29539	4.78253	-3.27771
H	1.46422	4.45637	-3.40389
H	0.86377	5.71677	-2.28600
C	1.87882	3.59388	-0.78206
H	2.04029	4.52609	-0.20001
H	2.74222	3.47036	-1.46825
H	1.90125	2.73653	-0.07822
C	-1.45056	-2.89800	-0.53994
C	-2.69835	-3.38400	-0.02579
C	-2.74383	-4.66843	0.55297
H	-3.70017	-5.04528	0.95179
C	-1.60181	-5.47834	0.63038
H	-1.65939	-6.47985	1.08558
C	-0.38931	-5.00443	0.11307
H	0.50535	-5.64658	0.16065
C	-0.28590	-3.72747	-0.47840
C	-3.98045	-2.54589	-0.07347
H	-3.74875	-1.61410	-0.62911
C	-4.44065	-2.13438	1.34178
H	-4.67087	-3.02215	1.96882
H	-5.35931	-1.51191	1.29382
H	-3.66307	-1.54483	1.87048
C	-5.12287	-3.26497	-0.82284
H	-4.81737	-3.56943	-1.84495
H	-6.00736	-2.60030	-0.91844
H	-5.45367	-4.18107	-0.28873
C	1.06051	-3.28725	-1.05859
H	0.93556	-2.25369	-1.44856
C	1.48734	-4.17599	-2.24810
H	1.67572	-5.22215	-1.92537
H	2.42273	-3.79101	-2.70494
H	0.71103	-4.20662	-3.04007
C	2.17178	-3.24911	0.00985
H	1.92384	-2.55709	0.84164
H	3.12796	-2.90148	-0.43445
H	2.35533	-4.25184	0.45120
C	4.32595	-0.03473	2.01163
C	3.44128	-0.82549	2.98540
H	3.38441	-1.90517	2.72064
H	2.39699	-0.43801	3.00932
H	3.83079	-0.77863	4.03816
C	4.41999	1.42373	2.49365
H	3.42377	1.92000	2.53621
H	5.06646	2.04699	1.83681
H	4.85349	1.49231	3.52769
C	5.73642	-0.65005	2.00087
H	6.20527	-0.63355	3.02114
H	6.42750	-0.10252	1.32327
H	5.72989	-1.71232	1.67033
C	4.27285	0.01088	-1.82118
H	4.49810	-2.18331	-2.05308
C	5.19211	1.23992	-1.95674

H	6.04429	1.21029	-1.24240
H	5.63664	1.30955	-2.98530
H	2.51273	1.01569	-2.70320
C	5.10680	-1.25209	-2.10046
H	5.56076	-1.22631	-3.12680
H	5.94641	-1.37284	-1.38134
H	4.65201	2.19726	-1.78160
C	3.14071	0.10962	-2.85336
H	2.45512	-0.76613	-2.81122
H	3.53446	0.15897	-3.90346
H	1.13851	2.13135	2.04396
O	-0.46310	0.09386	2.82396
C	-0.78143	-1.01071	3.70648
C	-0.63633	-2.33595	2.98578
H	-1.81477	-0.86483	4.10049
H	-0.08354	-0.96631	4.57289
H	0.40551	-2.49092	2.63928
H	-0.88315	-3.16432	3.67988
H	-1.32434	-2.43442	2.12025
C	-0.16127	1.29691	3.57661
C	0.24731	2.41670	2.64246
H	0.66172	1.05514	4.28731
H	-1.05940	1.57552	4.17612
H	-0.57665	2.72568	1.96612
H	0.52686	3.31034	3.23535

TS(A–B)

SCF (BP86) Energy = -4244.911688
Enthalpy 0K = -4243.926646
Enthalpy 298K = -4243.864290
Free Energy 298K = -4244.026081
Lowest Frequency = -52.0612 cm^{-1}
Second Frequency = 9.2022 cm^{-1}
SCF (BP86-D3^{BJ}) Energy = -4245.200078
SCF ($C_6H_5CH_3$) Energy = -4244.954525
SCF (BS2) Energy = -4247.196110

Zn	3.26314	-1.56331	-0.51801
H	2.19207	-0.40949	-0.60460
Ca	-0.00393	-0.42019	0.27928
N	-2.41299	-0.13816	0.62794
N	0.17261	1.91648	0.89180
C	-2.72800	0.68831	1.63380
C	-1.82076	1.56140	2.31316
H	-2.26060	1.98035	3.23211
C	-0.60333	2.20329	1.95954
C	-4.16157	0.79948	2.15177
H	-4.20879	0.47370	3.21221
H	-4.87582	0.19314	1.56597
H	-4.49535	1.85705	2.13117
C	-0.18753	3.30284	2.93981
H	0.26048	2.82865	3.84013
H	-1.05723	3.89252	3.29040
H	0.56260	3.99237	2.51116
C	-3.43176	-0.85342	-0.08108
C	-3.88049	-0.33804	-1.34167
C	-4.83041	-1.06246	-2.08888
H	-5.17751	-0.65899	-3.05333
C	-5.35444	-2.27603	-1.62496
H	-6.10119	-2.82652	-2.21904
C	-4.91942	-2.77598	-0.39243
H	-5.33554	-3.72704	-0.02278
C	-3.96370	-2.09797	0.39577
C	-3.40434	1.02003	-1.85645
H	-2.45543	1.25050	-1.32520
C	-4.41904	2.12527	-1.48595
H	-5.40217	1.93514	-1.96786
H	-4.06365	3.12258	-1.82147
H	-4.58742	2.17522	-0.39185
C	-3.11848	1.03151	-3.37024
H	-2.42530	0.21460	-3.65767
H	-2.66201	1.99727	-3.67186
H	-4.04439	0.91433	-3.97254
C	-3.56513	-2.73080	1.73403
H	-2.70522	-2.15229	2.13510
C	-4.70990	-2.66573	2.77216
H	-5.06629	-1.63279	2.94550
H	-4.37480	-3.07896	3.74710
H	-5.58202	-3.26727	2.43693
C	-3.11828	-4.20056	1.57310
H	-3.96071	-4.84724	1.24819
H	-2.75159	-4.60326	2.54002
H	-2.30277	-4.31107	0.83230
C	1.08889	2.90486	0.41023
C	0.58731	3.98503	-0.39501
C	1.48451	4.95612	-0.88371
H	1.09486	5.78958	-1.48990
C	2.85791	4.88787	-0.61886
H	3.54187	5.65941	-1.00683
C	3.34947	3.82066	0.14192
H	4.42964	3.76488	0.35051
C	2.49787	2.82300	0.66252
C	-0.90163	4.12561	-0.72906
H	-1.37250	3.14080	-0.52397
C	-1.14886	4.47254	-2.21233
H	-0.79849	5.49579	-2.46476
H	-2.23508	4.44280	-2.44063
H	-0.63469	3.76706	-2.89601
C	-1.59818	5.17210	0.17003
H	-1.50407	4.92545	1.24470
H	-2.68117	5.23808	-0.06842
H	-1.15629	6.18047	0.01691
C	3.11236	1.72346	1.53117
H	2.37592	0.89207	1.57202
C	3.34704	2.19821	2.98292
H	4.07501	3.03737	3.00973
H	3.75317	1.37434	3.60740
H	2.41165	2.55228	3.45610
C	4.42823	1.16753	0.95127
H	4.31856	0.87954	-0.11616
H	4.76562	0.27983	1.52824
H	5.25397	1.90809	1.00696
C	4.72533	-2.81610	-1.06320
C	5.32726	-2.29274	-2.38296
H	5.76025	-1.27398	-2.27822
H	4.57949	-2.25383	-3.20554
H	6.15461	-2.95960	-2.73319
C	4.15224	-4.22908	-1.28036
H	3.37210	-4.25512	-2.07308

H	3.70406	-4.65292	-0.35535
H	4.95769	-4.93666	-1.60038
C	5.81531	-2.86079	0.02286
H	6.64121	-3.55358	-0.27657
H	5.42729	-3.22568	0.99818
H	6.27973	-1.86607	0.19807
C	1.03130	-2.00317	2.00426
H	-0.39453	-0.90387	3.31208
C	2.54327	-2.23230	1.98740
H	3.13569	-1.29951	1.79687
H	2.95893	-2.62709	2.96135
H	0.59626	-4.09687	1.41286
C	0.69673	-1.11196	3.21288
H	1.01002	-1.57962	4.19060
H	1.20464	-0.12030	3.17806
H	2.83828	-3.00629	1.23405
C	0.36935	-3.36751	2.22475
H	-0.73563	-3.29419	2.29524
H	0.70672	-3.85842	3.18338
H	-0.37581	-3.22855	-0.46332
O	-0.00968	-1.12491	-2.16122
C	0.71478	-0.38499	-3.16361
C	0.70647	1.09664	-2.84413
H	0.23919	-0.58095	-4.15371
H	1.76055	-0.77547	-3.21301
H	1.24431	1.32381	-1.90067
H	1.22062	1.65515	-3.65235
H	-0.32886	1.48176	-2.76824
C	-0.11373	-2.51816	-2.51319
C	-0.88774	-3.27258	-1.44966
H	0.91546	-2.93691	-2.63574
H	-0.61918	-2.59564	-3.50472
H	-1.91719	-2.87533	-1.34222
H	-0.96205	-4.34271	-1.73014

B

SCF (BP86) Energy = -4244.912726
Enthalpy 0K = -4243.927489
Enthalpy 298K = -4243.864517
Free Energy 298K = -4244.026790
Lowest Frequency = 14.9254 cm^{-1}
Second Frequency = 18.6575 cm^{-1}
SCF (BP86-D3^{BJ}) Energy = -4245.199347
SCF ($C_6H_5CH_3$) Energy = -4244.956139
SCF (BS2) Energy = -4247.197081

Zn	3.53615	-1.48873	-0.62685
H	2.14849	-0.77651	-0.88864
Ca	-0.01004	-0.43736	0.24535
N	-2.42461	-0.36298	0.60230
N	-0.02310	1.87997	0.87279
C	-2.74914	0.34926	1.68681
C	-1.87206	1.24186	2.38520
H	-2.27532	1.53684	3.36666
C	-0.73438	2.00813	2.01586
C	-4.14862	0.27850	2.29316
H	-4.11050	-0.23577	3.27693
H	-4.86060	-0.26561	1.64574
H	-4.54250	1.29767	2.48052
C	-0.30519	3.03233	3.06666
H	0.34868	2.52458	3.80931
H	-1.16921	3.44420	3.62330
H	0.27292	3.86767	2.62952
C	-3.40001	-1.11857	-0.12364
C	-3.90086	-0.57720	-1.35384
C	-4.77663	-1.34628	-2.14473
H	-5.16142	-0.92497	-3.08679
C	-5.18120	-2.62919	-1.75160
H	-5.86810	-3.21657	-2.38150
C	-4.71043	-3.14743	-0.54030
H	-5.04040	-4.14922	-0.22045
C	-3.82679	-2.42397	0.29106
C	-3.56933	0.85408	-1.77321
H	-2.61988	1.12623	-1.26302
C	-4.65969	1.82337	-1.26204
H	-5.64604	1.57729	-1.71082
H	-4.41744	2.87395	-1.52797
H	-4.77046	1.76908	-0.16064
C	-3.36242	1.02126	-3.28995
H	-2.62047	0.29535	-3.68137
H	-3.00164	2.04507	-3.52112
H	-4.30497	0.87918	-3.86040
C	-3.39567	-3.07767	1.60843
H	-2.64870	-2.40971	2.08723
C	-4.58436	-3.24503	2.58387
H	-5.11200	-2.29073	2.77492
H	-4.23383	-3.64323	3.55962
H	-5.33120	-3.96261	2.18170
C	-2.72594	-4.45134	1.38741
H	-3.43952	-5.18326	0.95273
H	-2.36791	-4.87004	2.35074
H	-1.85369	-4.38456	0.70943
C	0.77152	2.97561	0.41472
C	0.12654	4.07882	-0.24375
C	0.90654	5.15532	-0.71132
H	0.40694	6.00534	-1.20319
C	2.30019	5.17102	-0.57039
H	2.89083	6.02441	-0.93960
C	2.93079	4.08185	0.04159
H	4.02708	4.09052	0.15318
C	2.19914	2.98061	0.53545
C	-1.39073	4.12401	-0.44975
H	-1.76746	3.09142	-0.29181
C	-1.77845	4.55823	-1.87889
H	-1.51782	5.61937	-2.07775
H	-2.87461	4.46553	-2.02879
H	-1.27290	3.94145	-2.64991
C	-2.08790	5.03767	0.58327
H	-1.89005	4.71377	1.62323
H	-3.18846	5.03541	0.43216
H	-1.73533	6.08726	0.48522
C	2.96675	1.86111	1.24007
H	2.27003	1.00172	1.35572
C	3.40890	2.28059	2.65974
H	4.11523	3.13726	2.61593
H	3.91947	1.44352	3.18119
H	2.54559	2.59072	3.27975
C	4.18774	1.38180	0.43021
H	3.90874	1.10899	-0.60986

H	4.66350	0.50369	0.92021
H	4.97686	2.16028	0.36241
C	5.30438	-2.37566	-0.91955
C	6.03270	-1.65877	-2.07326
H	6.25694	-0.59586	-1.83678
H	5.44934	-1.67402	-3.02014
H	7.01236	-2.15353	-2.29156
C	5.03713	-3.84323	-1.31040
H	4.42521	-3.93430	-2.23504
H	4.51710	-4.40823	-0.50623
H	5.99871	-4.37985	-1.50944
C	6.17096	-2.33133	0.35109
H	7.14781	-2.84925	0.17981
H	5.68445	-2.83508	1.21374
H	6.41228	-1.29196	0.66349
C	1.12419	-1.82292	2.08055
H	0.02945	-0.67211	3.62316
C	2.58788	-2.16817	1.80844
H	3.21874	-1.26000	1.61444
H	3.10771	-2.68926	2.66504
H	0.42193	-3.86548	1.55226
C	1.07160	-0.94912	3.34530
H	1.51156	-1.46800	4.24553
H	1.63598	0.00470	3.23731
H	2.70180	-2.87830	0.94508
C	0.40780	-3.14160	2.40008
H	-0.65708	-2.98428	2.67669
H	0.87500	-3.68354	3.27327
H	-0.03189	-3.19424	-0.63204
O	-0.07459	-0.98195	-2.24327
C	0.51058	-0.12404	-3.24064
C	0.47204	1.32242	-2.78922
H	-0.05258	-0.26022	-4.19454
H	1.56171	-0.45395	-3.42347
H	1.09762	1.49847	-1.89020
H	0.86911	1.97558	-3.59233
H	-0.56332	1.64704	-2.56691
C	-0.03321	-2.35913	-2.65525
C	-0.62115	-3.24607	-1.57442
H	1.02742	-2.63779	-2.87038
H	-0.60312	-2.46420	-3.60865
H	-1.67436	-2.97441	-1.35905
H	-0.60415	-4.30425	-1.90478

TS(B–C)

SCF (BP86) Energy = -4244.893908
Enthalpy 0K = -4243.911994
Enthalpy 298K = -4243.850102
Free Energy 298K = -4244.009586
Lowest Frequency = -1361.3789 cm^{-1}
Second Frequency = 11.3542 cm^{-1}
SCF (BP86-D3^{BJ}) Energy = -4245.185159
SCF ($C_6H_5CH_3$) Energy = -4244.931918
SCF (BS2) Energy = -4247.172750

Zn	-2.99678	-1.40848	0.32412
H	-2.25902	0.03250	0.15377
Ca	-0.03651	-0.14736	-0.02808
N	2.27100	-0.45639	-0.68764
N	0.20622	2.04256	-0.95538
C	2.70521	0.20251	-1.77101
C	1.98606	1.22780	-2.45816
H	2.46240	1.51000	-3.41013
C	0.95371	2.12742	-2.07545
C	4.08249	-0.07796	-2.36787
H	3.97960	-0.55741	-3.36425
H	4.69352	-0.73989	-1.72733
H	4.63322	0.87109	-2.52651
C	0.72786	3.26779	-3.06814
H	0.08348	2.90604	-3.89844
H	1.67837	3.60718	-3.52392
H	0.22176	4.13486	-2.60485
C	3.09865	-1.40039	0.00112
C	3.64750	-1.02409	1.27166
C	4.37681	-1.97081	2.01716
H	4.80107	-1.68016	2.99069
C	4.58572	-3.27219	1.54048
H	5.15823	-3.99979	2.13729
C	4.06543	-3.63209	0.29261
H	4.23960	-4.65171	-0.08814
C	3.32389	-2.72634	-0.49660
C	3.52598	0.41252	1.78016
H	2.59638	0.83481	1.33741
C	4.70016	1.26988	1.25537
H	5.66885	0.87898	1.63386
H	4.60433	2.32495	1.58901
H	4.74565	1.26707	0.14827
C	3.41651	0.52561	3.31128
H	2.61326	-0.12487	3.71411
H	3.19611	1.57256	3.60639
H	4.36317	0.24460	3.81973
C	2.81156	-3.21714	-1.85552
H	2.23762	-2.38717	-2.31909
C	3.96635	-3.58905	-2.81421
H	4.68757	-2.75972	-2.94886
H	3.56924	-3.86347	-3.81458
H	4.53426	-4.46458	-2.43372
C	1.86078	-4.42483	-1.71039
H	2.39821	-5.31593	-1.32228
H	1.42449	-4.70289	-2.69273
H	1.02742	-4.21225	-1.01282
C	-0.50446	3.19581	-0.48183
C	0.19188	4.12276	0.36764
C	-0.49787	5.24053	0.87789
H	0.03710	5.95489	1.52302
C	-1.84823	5.46646	0.58018
H	-2.36962	6.34764	0.98680
C	-2.52575	4.55790	-0.24000
H	-3.58654	4.73727	-0.47753
C	-1.88712	3.42105	-0.78176
C	1.67776	3.94648	0.69778
H	1.90655	2.86509	0.57717
C	2.03665	4.35473	2.14081
H	1.96876	5.45294	2.29216
H	3.08375	4.06766	2.37289
H	1.37432	3.87419	2.88907
C	2.57785	4.71765	-0.29479
H	2.42035	4.39065	-1.33999
H	3.65134	4.56555	-0.05276

H	2.36947	5.80826	-0.24659
C	-2.70083	2.51531	-1.71108
H	-2.10547	1.58997	-1.87090
C	-2.93593	3.17399	-3.09033
H	-3.55166	4.09334	-2.98873
H	-3.47829	2.48152	-3.76893
H	-1.98850	3.46345	-3.58411
C	-4.05558	2.10635	-1.09776
H	-3.92679	1.63620	-0.10326
H	-4.57464	1.37736	-1.75570
H	-4.73463	2.97794	-0.98618
C	-4.48723	-2.26318	1.39178
C	-4.55290	-1.57273	2.76889
H	-4.70967	-0.47499	2.68597
H	-3.62909	-1.73106	3.36906
H	-5.39945	-1.97694	3.38094
C	-4.26914	-3.77330	1.59116
H	-3.32244	-3.99967	2.12930
H	-4.23965	-4.32583	0.62731
H	-5.09747	-4.22278	2.19638
C	-5.83126	-2.03726	0.67149
H	-6.67988	-2.44056	1.28094
H	-5.87848	-2.55019	-0.31361
H	-6.04597	-0.96031	0.49630
C	-2.11829	-2.36838	-1.68080
H	0.03473	-1.92133	-2.01320
C	-3.45201	-1.87000	-2.28380
H	-3.59838	-0.77280	-2.16567
H	-3.48756	-2.07073	-3.38568
H	-2.86798	-4.40908	-1.36489
C	-0.97258	-1.65626	-2.41530
H	-0.92964	-1.92343	-3.49898
H	-1.09138	-0.54531	-2.40145
H	-4.33528	-2.38315	-1.84778
C	-2.03231	-3.88762	-1.87813
H	-1.08642	-4.31414	-1.48419
H	-2.08592	-4.16524	-2.95971
H	-1.49194	-2.33135	-0.29738
O	-0.27954	-0.66880	2.25813
C	-0.55476	-0.00523	3.50032
C	-0.38884	1.49382	3.32862
H	0.13281	-0.40135	4.28514
H	-1.59544	-0.25642	3.81293
H	-1.08652	1.89603	2.56510
H	-0.60123	2.01226	4.28560
H	0.64794	1.74856	3.03043
C	-0.53280	-2.11011	2.28637
C	-0.53793	-2.65877	0.86835
H	-1.52034	-2.27080	2.77825
H	0.24852	-2.55966	2.94851
H	0.51323	-2.76152	0.50163
H	-0.92001	-3.69975	0.91431

C

SCF (BP86) Energy = -4244.976811
Enthalpy 0K = -4243.991134
Enthalpy 298K = -4243.927167
Free Energy 298K = -4244.107966
Lowest Frequency = 1.2118 cm^{-1}
Second Frequency = 2.1963 cm^{-1}
SCF (BP86-D3^{BJ}) Energy = -4245.237742
SCF (C6H5CH3) Energy = -4245.020948
SCF (BS2) Energy = -4247.261618

Zn	1.88104	-0.23047	-1.22798
H	0.32584	-0.62135	-1.81895
Ca	-0.91894	-0.06756	-0.11348
N	-2.01903	2.01764	-0.38466
N	-3.11328	-0.89020	-0.51692
C	-3.21305	2.21703	-0.96846
C	-4.09102	1.18378	-1.39556
H	-4.98250	1.54461	-1.92771
C	-4.08886	-0.21574	-1.15459
C	-3.71376	3.63978	-1.17239
H	-2.99067	4.24733	-1.75263
H	-3.82524	4.15139	-0.19308
H	-4.69086	3.65975	-1.68963
C	-5.32673	-0.96500	-1.63177
H	-5.05353	-1.92213	-2.11961
H	-5.91785	-0.35521	-2.34061
H	-5.98627	-1.22904	-0.77840
C	-1.24501	3.13549	0.05966
C	-1.30074	3.52465	1.43594
C	-0.44266	4.54509	1.89434
H	-0.48039	4.84579	2.95448
C	0.45302	5.18838	1.02970
H	1.11726	5.98240	1.40614
C	0.49155	4.81506	-0.32082
H	1.19228	5.32704	-1.00064
C	-0.34268	3.80017	-0.83251
C	-2.26878	2.85932	2.41798
H	-2.88723	2.14619	1.83320
C	-3.22893	3.87791	3.06840
H	-2.68545	4.60685	3.70659
H	-3.97149	3.36197	3.71342
H	-3.78716	4.45675	2.30405
C	-1.51854	2.04796	3.49623
H	-0.88094	1.25765	3.04557
H	-2.23096	1.55253	4.18917
H	-0.85361	2.69709	4.10489
C	-0.24743	3.43814	-2.31829
H	-1.02448	2.67298	-2.52555
C	-0.52635	4.65025	-3.23452
H	-1.50419	5.12447	-3.01017
H	-0.53705	4.33912	-4.30034
H	0.25397	5.43320	-3.12620
C	1.11857	2.81277	-2.66889
H	1.95508	3.51641	-2.47388
H	1.15996	2.51914	-3.73830
H	1.30904	1.89848	-2.06532
C	-3.34297	-2.24306	-0.12224
C	-4.12366	-2.52164	1.04933
C	-4.27576	-3.85960	1.46446
H	-4.87322	-4.07948	2.36422
C	-3.68369	-4.91806	0.75961
H	-3.81947	-5.95714	1.09935
C	-2.91986	-4.64049	-0.38126
H	-2.45935	-5.47282	-0.93787
C	-2.73253	-3.31997	-0.84216

C	-4.75903	-1.40225	1.88164
H	-4.70705	-0.46887	1.28496
C	-3.95736	-1.15124	3.17782
H	-3.93969	-2.05575	3.82287
H	-4.40532	-0.32354	3.76795
H	-2.90489	-0.87549	2.95882
C	-6.24440	-1.66361	2.20614
H	-6.83656	-1.86619	1.28970
H	-6.69258	-0.78276	2.71223
H	-6.37633	-2.53254	2.88538
C	-1.92028	-3.08257	-2.11786
H	-1.75700	-1.98616	-2.21056
C	-2.69797	-3.52915	-3.37601
H	-2.89500	-4.62264	-3.35872
H	-2.11811	-3.30424	-4.29594
H	-3.67687	-3.01442	-3.45937
C	-0.53189	-3.75090	-2.07300
H	0.04999	-3.43067	-1.18396
H	0.05991	-3.47409	-2.96950
H	-0.60568	-4.85910	-2.04833
C	10.53186	-0.91274	1.37395
C	10.45803	-2.05962	2.39696
H	10.22973	-3.02978	1.90694
H	9.67319	-1.87553	3.16102
H	11.42536	-2.17652	2.93399
C	10.79573	0.43607	2.06559
H	10.01718	0.66573	2.82370
H	10.81165	1.27293	1.33564
H	11.77819	0.42988	2.58764
C	11.58469	-1.19922	0.28922
H	12.60186	-1.27798	0.73304
H	11.61525	-0.39226	-0.47315
H	11.37690	-2.15352	-0.23946
C	3.61550	-0.76445	-2.12777
H	3.18993	-2.93449	-1.96537
C	4.74836	0.19103	-1.71347
H	4.91531	0.19684	-0.61328
H	5.72373	-0.10004	-2.18424
H	3.22032	0.27882	-4.03979
C	3.97206	-2.19579	-1.68164
H	4.92731	-2.54345	-2.15580
H	4.11999	-2.27396	-0.58095
H	4.54811	1.24151	-2.02034
C	3.45800	-0.73858	-3.65782
H	2.64867	-1.41621	-4.00731
H	4.40065	-1.06249	-4.17192
H	9.53888	-0.84522	0.87044
O	0.33537	-1.01516	1.72185
C	0.17713	-1.93584	2.82136
C	-0.57456	-3.17830	2.37756
H	-0.34445	-1.41849	3.66146
H	1.19272	-2.21020	3.18744
H	-0.01692	-3.71951	1.58696
H	-0.70282	-3.86510	3.23921
H	-1.58601	-2.94134	1.98947
C	1.52505	-0.16209	1.85110
C	1.62886	0.74047	0.63963
H	2.40935	-0.83693	1.92769
H	1.43185	0.37648	2.82787
H	0.91694	1.59949	0.73371
H	2.62236	1.23775	0.67935

D

SCF (BP86) Energy = -4086.643444
Enthalpy 0K = -4085.785375
Enthalpy 298K = -4085.729836
Free Energy 298K = -4085.878503
Lowest Frequency = 11.5838 cm^{-1}
Second Frequency = 19.2795 cm^{-1}
SCF (BP86-D3^{BJ}) Energy = -4086.888965
SCF ($C_6H_5CH_3$) Energy = -4086.685804
SCF (BS2) Energy = -4088.750915

Zn	-2.87632	-1.63606	-0.52761
H	-1.50960	-1.41047	0.51176
Ca	0.06954	-0.08297	-0.07334
N	0.17499	2.02050	0.94620
N	2.12522	-0.33603	1.04646
C	0.95541	2.39799	1.97482
C	1.99905	1.60825	2.52451
H	2.49830	2.05544	3.39664
C	2.58861	0.39571	2.07134
C	0.74963	3.77196	2.59336
H	-0.30172	3.91205	2.91709
H	0.94831	4.56775	1.84503
H	1.41470	3.93594	3.46150
C	3.84684	-0.03058	2.81893
H	4.11879	-1.08400	2.61716
H	3.71108	0.10437	3.91085
H	4.71016	0.60315	2.52329
C	-0.77774	2.91751	0.37175
C	-0.39760	3.72718	-0.74879
C	-1.38184	4.50146	-1.39597
H	-1.09521	5.12331	-2.26009
C	-2.71556	4.49949	-0.96400
H	-3.47015	5.10926	-1.48568
C	-3.07872	3.71897	0.14172
H	-4.12586	3.72652	0.48603
C	-2.13604	2.92564	0.82856
C	1.04602	3.77297	-1.25878
H	1.65126	3.12879	-0.58708
C	1.63618	5.19814	-1.19007
H	1.10496	5.89534	-1.87256
H	2.70641	5.19467	-1.48691
H	1.56967	5.62106	-0.16640
C	1.16935	3.19868	-2.68710
H	0.81210	2.14889	-2.74228
H	2.22595	3.21617	-3.02849
H	0.57443	3.79022	-3.41550
C	-2.59153	2.11571	2.04633
H	-1.70181	1.57781	2.43746
C	-3.11894	3.02736	3.17719
H	-2.37427	3.79482	3.47333
H	-3.36586	2.42809	4.07875
H	-4.04252	3.56275	2.87038
C	-3.65081	1.05695	1.67705
H	-4.57116	1.52180	1.26505
H	-3.94147	0.46109	2.56711
H	-3.26370	0.34538	0.91659

C	2.85306	-1.43144	0.50065
C	3.98187	-1.20622	-0.35402
C	4.58965	-2.31355	-0.97996
H	5.45737	-2.14795	-1.63956
C	4.11658	-3.61853	-0.78555
H	4.60796	-4.46807	-1.28580
C	3.01572	-3.83468	0.05536
H	2.65351	-4.86198	0.21531
C	2.36793	-2.76598	0.70911
C	4.53412	0.19709	-0.62452
H	3.99085	0.90585	0.03311
C	4.27065	0.64108	-2.07974
H	4.76537	-0.03719	-2.80743
H	4.66168	1.66547	-2.25683
H	3.18612	0.65193	-2.31367
C	6.03690	0.30652	-0.28776
H	6.24957	-0.01952	0.75082
H	6.38656	1.35496	-0.39637
H	6.65696	-0.31829	-0.96530
C	1.21995	-3.04667	1.68336
H	0.58878	-2.12947	1.73436
C	1.75661	-3.28442	3.11335
H	2.41137	-4.18163	3.14203
H	0.91969	-3.44884	3.82437
H	2.34926	-2.42265	3.47939
C	0.30178	-4.20196	1.24851
H	-0.07415	-4.06414	0.21404
H	-0.58203	-4.25912	1.91585
H	0.81490	-5.18569	1.29935
C	-4.17681	-3.10021	0.02409
H	-4.50953	-2.39713	2.09434
C	-3.73902	-4.40587	-0.66588
H	-2.72188	-4.73055	-0.35126
H	-4.43302	-5.25275	-0.41957
H	-5.65167	-2.58252	-1.54466
C	-4.17755	-3.30692	1.54659
H	-4.87007	-4.13552	1.85123
H	-3.16840	-3.56879	1.93384
H	-3.73148	-4.31646	-1.77562
C	-5.59739	-2.73750	-0.44433
H	-5.97569	-1.80797	0.03640
H	-6.33250	-3.54813	-0.19595
O	-0.40851	-0.24161	-2.37430
C	-0.19094	-1.31803	-3.30504
C	1.18834	-1.92206	-3.10174
H	-0.29726	-0.90898	-4.33760
H	-0.98669	-2.08560	-3.16833
H	1.31245	-2.36242	-2.08887
H	1.34931	-2.74135	-3.83157
H	1.98858	-1.17002	-3.25328
C	-1.72181	0.42875	-2.56877
C	-2.91638	-0.41909	-2.19246
H	-1.70284	0.78425	-3.62905
H	-1.65129	1.34148	-1.93458
H	-3.77044	0.27136	-2.00527
H	-3.24246	-1.05218	-3.04863

TS(D-E)

SCF (BP86) Energy = -4086.632312
Enthalpy 0K = -4085.776129
Enthalpy 298K = -4085.720537
Free Energy 298K = -4085.869743
Lowest Frequency = -297.1740 cm^{-1}
Second Frequency = 13.5072 cm^{-1}
SCF (BP86-D3^{BJ}) Energy = -4086.879651
SCF ($C_6H_5CH_3$) Energy = -4086.671524
SCF (BS2) Energy = -4088.740827

Zn	-0.28189	3.18644	-0.15291
H	-0.23861	1.91586	0.95531
Ca	0.01296	-0.06259	0.06096
N	1.85402	-1.34874	0.76608
N	-1.13060	-1.97992	0.79431
C	1.86404	-2.50006	1.45881
C	0.70060	-3.22660	1.83059
H	0.89742	-4.12889	2.42644
C	-0.66535	-3.02684	1.49691
C	3.19646	-3.11186	1.86412
H	3.84537	-2.36501	2.36448
H	3.75616	-3.45396	0.96815
H	3.06135	-3.97836	2.53791
C	-1.63918	-4.09930	1.96053
H	-2.48876	-3.65069	2.51499
H	-1.14665	-4.84912	2.60718
H	-2.08469	-4.62763	1.09157
C	3.07120	-0.73207	0.34376
C	3.66064	-1.10462	-0.90924
C	4.78529	-0.39114	-1.37102
H	5.24043	-0.67000	-2.33560
C	5.33840	0.66338	-0.63084
H	6.21658	1.20869	-1.01121
C	4.76627	1.01491	0.59903
H	5.20926	1.83739	1.18402
C	3.63895	0.33812	1.11013
C	3.09428	-2.24564	-1.76089
H	2.29233	-2.73299	-1.16831
C	4.15597	-3.32049	-2.07787
H	4.96603	-2.92163	-2.72481
H	3.69665	-4.17696	-2.61521
H	4.63189	-3.71385	-1.15577
C	2.44799	-1.71936	-3.06138
H	1.63398	-0.99340	-2.85411
H	2.01460	-2.55377	-3.65250
H	3.19464	-1.20576	-3.70424
C	3.08427	0.73828	2.48147
H	2.15613	0.14860	2.64350
C	4.06736	0.37153	3.61654
H	4.32905	-0.70634	3.60787
H	3.62596	0.60458	4.60861
H	5.01490	0.94471	3.52778
C	2.71116	2.23329	2.55893
H	3.59991	2.88619	2.42616
H	2.27011	2.47314	3.54940
H	1.96410	2.50660	1.78625
C	-2.49890	-1.90721	0.39366
C	-2.91522	-2.51843	-0.83482
C	-4.23451	-2.30964	-1.28466
H	-4.56034	-2.77212	-2.23079
C	-5.14244	-1.52845	-0.55478

H	-6.16806	-1.37597	-0.92654
C	-4.73630	-0.95097	0.65546
H	-5.45584	-0.34981	1.23509
C	-3.42860	-1.12697	1.15441
C	-1.96066	-3.37865	-1.66928
H	-1.04247	-3.53369	-1.06579
C	-1.53488	-2.65988	-2.96798
H	-2.41024	-2.44757	-3.61832
H	-0.82464	-3.28310	-3.55160
H	-1.03514	-1.69104	-2.75771
C	-2.54822	-4.77038	-1.98510
H	-2.86367	-5.30195	-1.06344
H	-1.79615	-5.40300	-2.50214
H	-3.43515	-4.70431	-2.65045
C	-3.05308	-0.51649	2.50820
H	-1.98232	-0.75421	2.68822
C	-3.86397	-1.15393	3.65897
H	-4.94773	-0.92992	3.56228
H	-3.52761	-0.75912	4.64092
H	-3.75373	-2.25777	3.68121
C	-3.20335	1.01866	2.52570
H	-2.57294	1.50831	1.75495
H	-2.89744	1.42949	3.51104
H	-4.25474	1.32963	2.34861
C	-1.52159	4.80113	-0.18296
H	-2.63748	4.25827	1.64740
C	-1.84887	5.21183	-1.62725
H	-2.44475	4.43983	-2.16277
H	-2.45366	6.15530	-1.65410
H	0.21832	6.16714	-0.02252
C	-2.82502	4.54655	0.59025
H	-3.47015	5.46334	0.61429
H	-3.43812	3.73897	0.13271
H	-0.93406	5.40291	-2.23065
C	-0.73647	5.93743	0.50072
H	-0.48793	5.70322	1.55995
H	-1.33028	6.88835	0.51537
O	-0.09979	1.06420	-1.88290
C	-1.04823	1.39006	-2.89181
C	-2.47913	1.20315	-2.39280
H	-0.87372	0.76647	-3.80373
H	-0.90786	2.45507	-3.20078
H	-2.67785	1.85774	-1.51690
H	-3.20764	1.47506	-3.18528
H	-2.68645	0.15272	-2.09684
C	1.47841	1.99730	-2.09754
C	1.48518	3.22970	-1.37922
H	1.40238	2.00315	-3.19988
H	2.13012	1.18112	-1.73346
H	2.24925	3.30594	-0.58252
H	1.37680	4.15198	-1.97763

E

SCF (BP86) Energy = -4086.669345
Enthalpy 0K = -4085.813955
Enthalpy 298K = -4085.756492
Free Energy 298K = -4085.911179
Lowest Frequency = 10.7427 cm^{-1}
Second Frequency = 14.9607 cm^{-1}
SCF (BP86-D3^{BJ}) Energy = -4086.913166
SCF ($C_6H_5CH_3$) Energy = -4086.706447
SCF (BS2) Energy = -4088.778514

Zn	-0.23569	2.91048	-0.00297
H	0.14925	1.75481	1.15229
Ca	-0.00044	-0.14039	-0.00338
N	1.70991	-1.72282	0.40276
N	-1.32882	-1.99949	0.56310
C	1.60797	-2.99667	0.82100
C	0.38351	-3.65786	1.10658
H	0.49660	-4.69147	1.46259
C	-0.96304	-3.22072	0.98534
C	2.87514	-3.81953	0.99984
H	3.56829	-3.33425	1.71724
H	3.43102	-3.89369	0.04157
H	2.65443	-4.84217	1.35798
C	-2.03820	-4.23148	1.35654
H	-2.74318	-3.80163	2.09764
H	-1.60294	-5.15821	1.77460
H	-2.65166	-4.50231	0.47182
C	2.98806	-1.14041	0.14703
C	3.52407	-1.16064	-1.18173
C	4.73088	-0.48016	-1.44222
H	5.14690	-0.49180	-2.46297
C	5.41438	0.20842	-0.42985
H	6.35524	0.73550	-0.65377
C	4.89018	0.21885	0.87033
H	5.43241	0.75623	1.66565
C	3.68714	-0.44549	1.18795
C	2.80567	-1.89225	-2.31882
H	1.97471	-2.46889	-1.86101
C	3.71969	-2.89952	-3.04673
H	4.55151	-2.39306	-3.58092
H	3.14387	-3.47288	-3.80362
H	4.17100	-3.62676	-2.34052
C	2.18007	-0.89508	-3.31872
H	1.48740	-0.18456	-2.81816
H	1.60872	-1.42702	-4.10876
H	2.96126	-0.28506	-3.82077
C	3.17595	-0.42025	2.63206
H	2.24423	-1.02380	2.65998
C	4.18071	-1.07150	3.60827
H	4.44607	-2.10474	3.30283
H	3.75470	-1.11997	4.63267
H	5.12549	-0.49030	3.66892
C	2.81859	1.00800	3.09762
H	3.70944	1.67216	3.08784
H	2.42769	0.99349	4.13729
H	2.04108	1.46898	2.45274
C	-2.70310	-1.66209	0.37592
C	-3.35669	-1.98922	-0.85747
C	-4.66904	-1.52512	-1.07412
H	-5.17731	-1.76814	-2.02139
C	-5.34404	-0.76048	-0.11078
H	-6.36940	-0.40643	-0.30217
C	-4.70506	-0.45471	1.09764
H	-5.24137	0.13738	1.85720
C	-3.39113	-0.89175	1.36740
C	-2.64597	-2.80016	-1.94582

H	-1.74251	-3.25131	-1.48636
C	-2.15871	-1.88837	-3.09274
H	-3.01278	-1.40203	-3.61037
H	-1.58815	-2.46789	-3.84932
H	-1.50014	-1.07603	-2.71943
C	-3.50904	-3.95425	-2.49503
H	-3.87535	-4.61558	-1.68250
H	-2.92126	-4.57696	-3.20199
H	-4.39765	-3.58323	-3.04848
C	-2.75348	-0.56811	2.72206
H	-1.70758	-0.94394	2.68777
C	-3.46715	-1.31290	3.87264
H	-4.51957	-0.97357	3.97992
H	-2.95435	-1.12722	4.84016
H	-3.48690	-2.40920	3.70387
C	-2.70148	0.94748	3.00687
H	-2.13583	1.49695	2.22591
H	-2.20126	1.14498	3.97849
H	-3.71874	1.38982	3.06166
C	-1.03419	4.76915	0.08131
H	-2.72300	4.06153	1.33440
C	-0.86437	5.56265	-1.22506
H	-1.34575	5.06182	-2.09284
H	-1.33032	6.57797	-1.14129
H	0.69802	5.73195	1.08073
C	-2.53833	4.60647	0.38277
H	-3.03436	5.60587	0.48393
H	-3.08108	4.06140	-0.42015
H	0.20327	5.72558	-1.49001
C	-0.38575	5.54605	1.24254
H	-0.48749	5.01338	2.21332
H	-0.86620	6.54985	1.37036
O	-0.40329	1.45503	-1.46295
C	-0.76508	1.84154	-2.77662
C	-2.26196	2.11934	-2.92593
H	-0.47034	1.03809	-3.49738
H	-0.18965	2.74966	-3.08669
H	-2.57853	2.94437	-2.25416
H	-2.51039	2.41313	-3.96850
H	-2.86092	1.22215	-2.66490
C	2.59390	2.81513	-1.15525
C	2.46515	3.85610	-0.31061
H	2.39948	2.92085	-2.23551
H	2.91464	1.81964	-0.80409
H	2.67607	3.75322	0.76706
H	2.18013	4.85977	-0.66655

TS(E-F)

SCF (BP86) Energy = -4086.668755
Enthalpy 0K = -4085.814003
Enthalpy 298K = -4085.756876
Free Energy 298K = -4085.912529
Lowest Frequency = -17.9935 cm^{-1}
Second Frequency = 12.7187 cm^{-1}
SCF (BP86-D3^{BJ}) Energy = -4086.906665
SCF ($C_6H_5CH_3$) Energy = -4086.708288
SCF (BS2) Energy = -4088.779185

Zn	0.26017	2.87299	0.13645
H	0.36261	1.63581	1.25493
Ca	-0.07768	-0.16560	0.01824
N	1.18273	-2.11976	0.40114
N	-1.83444	-1.64180	0.51140
C	0.76138	-3.33932	0.77946
C	-0.59462	-3.68517	1.02637
H	-0.74692	-4.72355	1.35269
C	-1.78972	-2.92752	0.89801
C	1.78290	-4.45327	0.95273
H	2.56176	-4.16906	1.68978
H	2.32003	-4.64189	-0.00056
H	1.31038	-5.39704	1.28256
C	-3.08801	-3.65675	1.21177
H	-3.71749	-3.06466	1.90701
H	-2.90037	-4.65140	1.65748
H	-3.69504	-3.79855	0.29286
C	2.57097	-1.86689	0.17782
C	3.11143	-1.99631	-1.14274
C	4.45716	-1.64347	-1.36713
H	4.87843	-1.73965	-2.38103
C	5.27218	-1.17435	-0.32668
H	6.32241	-0.90593	-0.52225
C	4.73845	-1.04893	0.96348
H	5.38119	-0.68027	1.77971
C	3.39795	-1.38628	1.24444
C	2.24947	-2.48615	-2.30943
H	1.30255	-2.87126	-1.87608
C	2.90255	-3.64457	-3.09079
H	3.83098	-3.32423	-3.60955
H	2.20940	-4.03010	-3.86800
H	3.16823	-4.48975	-2.42266
C	1.88467	-1.32137	-3.25645
H	1.37793	-0.49017	-2.71957
H	1.20898	-1.66286	-4.06914
H	2.79197	-0.88859	-3.72918
C	2.87253	-1.23862	2.67576
H	1.81504	-1.57815	2.67281
C	3.64393	-2.13552	3.66897
H	3.63415	-3.20107	3.35946
H	3.19436	-2.07314	4.68250
H	4.70684	-1.82497	3.75780
C	2.88968	0.23173	3.14737
H	3.92346	0.63766	3.17352
H	2.47220	0.31802	4.17310
H	2.28854	0.88708	2.48308
C	-3.07674	-0.96828	0.31272
C	-3.77712	-1.11065	-0.93013
C	-4.93519	-0.33905	-1.14937
H	-5.47823	-0.44087	-2.10298
C	-5.41269	0.55668	-0.18100
H	-6.32078	1.14972	-0.37341
C	-4.72586	0.69016	1.03253
H	-5.10680	1.39023	1.79377
C	-3.55939	-0.05507	1.30518
C	-3.26905	-2.04890	-2.02944
H	-2.50872	-2.71538	-1.57280
C	-2.55726	-1.25746	-3.14830
H	-3.25927	-0.56578	-3.66080
H	-2.13306	-1.94096	-3.91421
H	-1.72641	-0.63912	-2.74778

C	-4.37756	-2.94627	-2.61600
H	-4.90820	-3.51428	-1.82385
H	-3.94709	-3.67915	-3.33051
H	-5.13925	-2.35867	-3.17114
C	-2.87061	0.09396	2.66562
H	-1.90691	-0.45866	2.60517
C	-3.70080	-0.56815	3.78877
H	-4.68200	-0.06151	3.91111
H	-3.16716	-0.50981	4.76106
H	-3.90224	-1.63799	3.57658
C	-2.55349	1.56092	3.02138
H	-1.90914	2.04065	2.25616
H	-2.01345	1.61697	3.98995
H	-3.47626	2.17031	3.12283
C	0.33365	4.88190	0.17802
H	-1.38655	5.03046	1.56995
C	0.74123	5.48748	-1.17609
H	0.01433	5.25382	-1.98381
H	0.80059	6.60415	-1.11136
H	2.38963	4.91949	1.00081
C	-1.05143	5.42232	0.58461
H	-1.03194	6.53881	0.67206
H	-1.84173	5.17442	-0.15779
H	1.73950	5.13395	-1.51379
C	1.36755	5.27863	1.24993
H	1.10929	4.87232	2.25223
H	1.42830	6.39213	1.35451
O	-0.08931	1.54655	-1.37955
C	-0.36225	2.04564	-2.68025
C	-1.73707	2.70778	-2.78023
H	-0.30273	1.20869	-3.41896
H	0.42508	2.77759	-2.98719
H	-1.81049	3.56936	-2.08265
H	-1.92391	3.08608	-3.80816
H	-2.54398	1.99111	-2.52055
C	3.59234	2.32451	-1.10192
C	4.44581	3.35820	-1.01264
H	2.59741	2.43039	-1.56892
H	3.85166	1.32357	-0.71649
H	5.44018	3.24797	-0.54746
H	4.19166	4.35910	-1.40052

F

SCF (BP86) Energy = -4086.668824
Enthalpy 0K = -4085.814037
Enthalpy 298K = -4085.755959
Free Energy 298K = -4085.916220
Lowest Frequency = 6.7926 cm^{-1}
Second Frequency = 9.9530 cm^{-1}
SCF (BP86-D3^{BJ}) Energy = -4086.904859
SCF ($C_6H_5CH_3$) Energy = -4086.708908
SCF (BS2) Energy = -4088.779276

Zn	0.58200	2.81239	0.30990
H	0.49073	1.52914	1.37599
Ca	-0.12110	-0.15755	0.04569
N	0.91562	-2.24222	0.40171
N	-2.03579	-1.46139	0.41407
C	0.35894	-3.42601	0.71108
C	-1.03452	-3.64005	0.89121
H	-1.30518	-4.66892	1.16705
C	-2.13928	-2.75842	0.74906
C	1.25539	-4.64388	0.87610
H	2.01542	-4.47446	1.66634
H	1.82442	-4.83948	-0.05698
H	0.67544	-5.55004	1.13162
C	-3.51776	-3.35824	0.98450
H	-4.10372	-2.73733	1.69269
H	-3.45315	-4.38856	1.38121
H	-4.10472	-3.38565	0.04244
C	2.32985	-2.11998	0.23952
C	2.90427	-2.23629	-1.06776
C	4.28464	-2.00322	-1.22989
H	4.73214	-2.08991	-2.23336
C	5.10070	-1.66494	-0.14069
H	6.17833	-1.48974	-0.28736
C	4.53398	-1.55432	1.13672
H	5.17833	-1.29046	1.99138
C	3.15866	-1.77631	1.35649
C	2.04516	-2.58676	-2.28575
H	1.04850	-2.89365	-1.90456
C	2.61210	-3.77122	-3.09503
H	3.58805	-3.52387	-3.56408
H	1.91673	-4.05224	-3.91397
H	2.76552	-4.66604	-2.45701
C	1.83480	-1.35294	-3.19120
H	1.38986	-0.49841	-2.63637
H	1.16344	-1.59175	-4.04321
H	2.79816	-0.99083	-3.60894
C	2.59804	-1.65357	2.77689
H	1.51584	-1.89761	2.72446
C	3.25434	-2.66231	3.74526
H	3.16617	-3.70624	3.37943
H	2.77516	-2.61230	4.74583
H	4.33573	-2.44960	3.88453
C	2.72602	-0.21553	3.32508
H	3.78964	0.09628	3.40101
H	2.28484	-0.14407	4.34204
H	2.20492	0.52185	2.67935
C	-3.19241	-0.65443	0.19704
C	-3.84913	-0.67287	-1.07739
C	-4.90947	0.22553	-1.30819
H	-5.41803	0.21955	-2.28597
C	-5.33284	1.12811	-0.32114
H	-6.16464	1.82139	-0.52315
C	-4.69027	1.13931	0.92354
H	-5.02968	1.84470	1.69945
C	-3.62107	0.26433	1.20908
C	-3.39606	-1.61707	-2.19551
H	-2.72253	-2.37220	-1.74059
C	-2.57007	-0.86143	-3.25932
H	-3.18085	-0.08344	-3.76485
H	-2.19059	-1.55587	-4.03869
H	-1.69519	-0.34620	-2.80960
C	-4.56620	-2.37734	-2.85217
H	-5.17738	-2.91891	-2.10057
H	-4.18511	-3.12233	-3.58201
H	-5.24665	-1.69645	-3.40616
C	-2.98193	0.28528	2.60132

H	-2.08676	-0.37386	2.55922
C	-3.93323	-0.31224	3.66298
H	-4.85264	0.30282	3.76682
H	-3.43999	-0.35234	4.65727
H	-4.24946	-1.34226	3.39978
C	-2.51378	1.69227	3.02628
H	-1.78333	2.11890	2.30831
H	-2.01684	1.65258	4.01845
H	-3.36345	2.40228	3.11129
C	0.97022	4.78097	0.43083
H	-0.56832	5.08732	1.99810
C	1.33255	5.40230	-0.92880
H	0.50644	5.32042	-1.66788
H	1.55814	6.49393	-0.81927
H	3.08077	4.47600	1.03198
C	-0.27342	5.48877	1.00380
H	-0.07894	6.58306	1.14291
H	-1.15811	5.40464	0.33476
H	2.23399	4.93442	-1.38035
C	2.15095	4.95725	1.40586
H	1.93503	4.52977	2.40942
H	2.38495	6.04204	1.55713
O	0.10612	1.60954	-1.26632
C	-0.09202	2.19372	-2.54578
C	-1.39839	2.98379	-2.63371
H	-0.09379	1.39092	-3.32353
H	0.76529	2.86280	-2.80181
H	-1.40647	3.81466	-1.89598
H	-1.52954	3.42637	-3.64444
H	-2.27287	2.33351	-2.42205
C	3.94266	2.00115	-1.41810
C	4.79486	2.87312	-1.98157
H	2.84999	2.16069	-1.43864
H	4.29421	1.08437	-0.91453
H	5.88620	2.71113	-1.96296
H	4.44196	3.78859	-2.48611

G

SCF (BP86) Energy = -4008.144118
Enthalpy 0K = -4007.339296
Enthalpy 298K = -4007.286433
Free Energy 298K = -4007.431586
Lowest Frequency = 8.3248 cm^{-1}
Second Frequency = 11.9808 cm^{-1}
SCF (BP86-D3^{BJ}) Energy = -4008.366067
SCF ($C_6H_5CH_3$) Energy = -4008.184121
SCF (BS2) Energy = -4010.166007

Zn	-1.04906	-2.83492	-0.13992
H	-0.80951	-1.62327	-1.26156
Ca	0.06438	0.00955	0.00151
N	1.95300	1.31462	-0.47571
N	-0.97794	2.10557	-0.23692
C	2.04387	2.62163	-0.77562
C	0.94074	3.51534	-0.80225
H	1.19907	4.55260	-1.05777
C	-0.43954	3.29867	-0.53801
C	3.40602	3.21328	-1.10636
H	3.32951	4.27341	-1.41178
H	3.89432	2.64135	-1.92241
H	4.09126	3.14909	-0.23531
C	-1.33981	4.52303	-0.59981
H	-2.14265	4.38920	-1.35356
H	-0.77236	5.43939	-0.84703
H	-1.85581	4.67675	0.37103
C	3.11270	0.49005	-0.37545
C	3.85456	0.44710	0.85112
C	4.91237	-0.47508	0.97306
H	5.48572	-0.51654	1.91328
C	5.25245	-1.34276	-0.07594
H	6.08307	-2.05659	0.04185
C	4.52962	-1.29129	-1.27462
H	4.80466	-1.96772	-2.10048
C	3.46026	-0.38814	-1.45178
C	3.49083	1.35322	2.03156
H	2.82854	2.15409	1.64324
C	4.71699	2.03774	2.66830
H	5.39286	1.30808	3.16270
H	4.39652	2.76287	3.44569
H	5.31622	2.58939	1.91447
C	2.68225	0.57971	3.09585
H	1.77019	0.11659	2.66298
H	2.36169	1.25051	3.92098
H	3.28399	-0.24224	3.53874
C	2.72856	-0.34184	-2.79672
H	1.89070	0.38090	-2.68628
C	3.64587	0.19397	-3.91871
H	4.06306	1.19064	-3.66724
H	3.08623	0.29067	-4.87312
H	4.50274	-0.48972	-4.09938
C	2.12659	-1.70665	-3.19241
H	2.91341	-2.47934	-3.32454
H	1.57590	-1.62681	-4.15338
H	1.41054	-2.08136	-2.43123
C	-2.38164	1.96572	-0.00703
C	-2.88624	1.99985	1.33309
C	-4.25265	1.73422	1.55529
H	-4.64545	1.75745	2.58512
C	-5.12204	1.44419	0.49492
H	-6.18667	1.23871	0.68927
C	-4.62483	1.41881	-0.81532
H	-5.31117	1.19287	-1.64793
C	-3.26686	1.67439	-1.09586
C	-1.97437	2.30767	2.52395
H	-0.97655	2.56855	2.11157
C	-1.79510	1.06929	3.42924
H	-2.75886	0.76269	3.88898
H	-1.08168	1.27897	4.25443
H	-1.41548	0.19354	2.86043
C	-2.46501	3.52113	3.34129
H	-2.58186	4.42293	2.70538
H	-1.74437	3.76636	4.15002
H	-3.44659	3.32311	3.82206
C	-2.78399	1.63616	-2.54935
H	-1.70094	1.88151	-2.54237
C	-3.49444	2.69435	-3.42207
H	-4.58181	2.48510	-3.51083
H	-3.07298	2.70126	-4.44956
H	-3.38699	3.71716	-3.00532

C	-2.93955	0.23118	-3.17111
H	-2.39872	-0.54093	-2.58523
H	-2.53951	0.21501	-4.20734
H	-4.00697	-0.07284	-3.22243
C	-1.93391	-4.63651	-0.04598
H	-3.90035	-3.77653	0.51656
C	-0.98352	-5.72078	0.49221
H	-0.65735	-5.51957	1.53544
H	-1.48700	-6.72127	0.50072
H	-1.52279	-5.10470	-2.17235
C	-3.17077	-4.53867	0.86847
H	-3.71833	-5.51520	0.90370
H	-2.90361	-4.28313	1.91725
H	-0.06750	-5.83012	-0.12893
C	-2.38127	-5.01282	-1.47170
H	-3.08107	-4.26593	-1.90590
H	-2.91267	-5.99879	-1.47784
H	1.25439	-4.01770	1.78636
O	-0.19068	-1.72624	1.33925
C	0.07161	-2.36068	2.58145
C	1.34172	-3.21238	2.54616
H	-0.79603	-2.99885	2.88029
H	0.16943	-1.58676	3.38215
H	2.22777	-2.59528	2.28826
H	1.52742	-3.69294	3.53077

TS(G-H)

SCF (BP86) Energy = -4008.089963
Enthalpy 0K = -4007.286740
Enthalpy 298K = -4007.234758
Free Energy 298K = -4007.373956
Lowest Frequency = -13.2410 cm_{-1}
Second Frequency = 14.1151 cm_{-1}
SCF (BP86-D3$_{BJ}$) Energy = -4008.318761
SCF ($C_6H_5CH_3$) Energy = -4008.138496
SCF (BS2) Energy = -4010.112046

Zn	-2.67692	1.79990	0.43158
H	0.56930	-0.18669	2.88482
Ca	0.61079	-0.12496	0.81331
N	2.62139	-0.51574	-0.39687
N	-0.05377	-1.94422	-0.54686
C	2.96324	-1.55414	-1.17036
C	2.07868	-2.60914	-1.53564
H	2.53487	-3.39665	-2.15204
C	0.69121	-2.77852	-1.29050
C	4.37692	-1.63168	-1.72776
H	4.53953	-2.55447	-2.31539
H	5.12611	-1.59308	-0.91041
H	4.58849	-0.75818	-2.37951
C	0.02617	-3.98620	-1.93406
H	-0.45480	-4.62524	-1.16451
H	0.74842	-4.60137	-2.50251
H	-0.78487	-3.67115	-2.62330
C	3.52318	0.55407	-0.12338
C	3.52522	1.71190	-0.97000
C	4.30022	2.82893	-0.59801
H	4.30342	3.72179	-1.24524
C	5.07076	2.82682	0.57337
H	5.66802	3.71093	0.84799
C	5.08035	1.68585	1.38709
H	5.69516	1.68073	2.30209
C	4.32460	0.53914	1.06536
C	2.71536	1.75901	-2.26973
H	2.24946	0.76019	-2.40169
C	3.61132	2.01986	-3.49983
H	4.07511	3.02873	-3.46435
H	3.01868	1.96061	-4.43750
H	4.43466	1.27938	-3.57115
C	1.57197	2.79443	-2.19883
H	0.86076	2.56589	-1.37755
H	0.99449	2.81031	-3.14797
H	1.96398	3.82026	-2.02961
C	4.40764	-0.68947	1.97680
H	3.74794	-1.46835	1.53849
C	5.84155	-1.26478	2.01621
H	6.22916	-1.48996	1.00057
H	5.86798	-2.20527	2.60630
H	6.55179	-0.55483	2.49168
C	3.89360	-0.39867	3.40300
H	4.48317	0.40706	3.89153
H	3.98209	-1.30692	4.03682
H	2.82147	-0.10203	3.39564
C	-1.46214	-2.06814	-0.42754
C	-2.31505	-1.38435	-1.36109
C	-3.70805	-1.37837	-1.13752
H	-4.36754	-0.87987	-1.86749
C	-4.27595	-2.02952	-0.03089
H	-5.36763	-2.02594	0.11686
C	-3.44104	-2.70562	0.86926
H	-3.88803	-3.22786	1.73096
C	-2.04147	-2.74591	0.69679
C	-1.75338	-0.71110	-2.61797
H	-0.65442	-0.86299	-2.60624
C	-2.00568	0.81093	-2.63151
H	-3.09183	1.04577	-2.61924
H	-1.57921	1.28012	-3.54351
H	-1.52016	1.30829	-1.76239
C	-2.30349	-1.35668	-3.90907
H	-2.12596	-2.45145	-3.92682
H	-1.81526	-0.91962	-4.80568
H	-3.39783	-1.19623	-4.01376
C	-1.18920	-3.54149	1.68965
H	-0.13135	-3.43837	1.36634
C	-1.54293	-5.04556	1.65094
H	-2.57822	-5.23002	2.00986
H	-0.85708	-5.62277	2.30623
H	-1.46772	-5.46556	0.62612
C	-1.29000	-2.98899	3.12731
H	-0.91328	-1.94384	3.18879
H	-0.67244	-3.60167	3.81806
H	-2.33588	-3.01658	3.50285
C	-4.58648	2.33872	0.52413
H	-5.31562	1.13799	-1.18052
C	-4.58476	3.82114	0.95351
H	-4.08743	4.48192	0.21100
H	-5.63559	4.18630	1.05763
H	-4.81415	1.57361	2.59184
C	-5.29049	2.19113	-0.83343

H	-6.35057	2.53363	-0.75042
H	-4.81503	2.80326	-1.62923
H	-4.09398	3.98380	1.93741
C	-5.28830	1.47931	1.59149
H	-5.29630	0.40272	1.32222
H	-6.35168	1.80338	1.70356
H	-1.96841	3.10121	2.70953
O	-0.80641	1.72224	0.60476
C	-0.23836	2.78233	1.40560
C	-0.87699	2.88779	2.78627
H	-0.30405	3.74930	0.85251
H	0.85349	2.57585	1.52717
H	-0.72928	1.93474	3.33768
H	-0.42168	3.71473	3.37159

H

SCF (BP86) Energy = -4008.093460
Enthalpy 0K = -4007.290593
Enthalpy 298K = -4007.237761
Free Energy 298K = -4007.379072
Lowest Frequency = 12.4285 cm^{-1}
Second Frequency = 19.7281 cm^{-1}
SCF (BP86-D3^{BJ}) Energy = -4008.327353
SCF ($C_6H_5CH_3$) Energy = -4008.136972
SCF (BS2) Energy = -4010.112022

Zn	-3.04131	-1.03723	-0.45082
H	0.98415	0.61677	-2.86569
Ca	0.73616	0.18531	-0.86118
N	2.70418	0.27006	0.51128
N	0.13670	1.79261	0.78898
C	3.05881	1.11283	1.48652
C	2.21347	2.13853	2.00766
H	2.67176	2.76622	2.78492
C	0.85962	2.43303	1.72197
C	4.43628	0.98766	2.12071
H	4.58660	1.72541	2.93091
H	5.23509	1.12657	1.36363
H	4.58227	-0.03097	2.53697
C	0.20267	3.54683	2.52275
H	-0.14622	4.35505	1.84562
H	0.89380	3.98641	3.26610
H	-0.69742	3.17808	3.05716
C	3.58513	-0.75159	0.04612
C	3.44207	-2.08547	0.55133
C	4.21733	-3.11861	-0.01355
H	4.11212	-4.14543	0.37460
C	5.12255	-2.86618	-1.05310
H	5.71856	-3.68659	-1.48358
C	5.26345	-1.55894	-1.53864
H	5.97637	-1.36173	-2.35587
C	4.51462	-0.48608	-1.01302
C	2.48211	-2.41082	1.70005
H	1.99750	-1.45754	1.99997
C	3.22826	-2.95812	2.93615
H	3.71523	-3.93317	2.72107
H	2.52723	-3.11575	3.78342
H	4.02041	-2.25978	3.27618
C	1.36659	-3.38384	1.26137
H	0.76377	-2.97207	0.42410
H	0.67110	-3.59532	2.10117
H	1.78311	-4.35543	0.92028
C	4.72301	0.91864	-1.58775
H	4.07438	1.61121	-1.01084
C	6.18287	1.39569	-1.41795
H	6.52205	1.34346	-0.36193
H	6.28973	2.44774	-1.75727
H	6.88542	0.78183	-2.02120
C	4.28675	1.00611	-3.06646
H	4.88063	0.31588	-3.70401
H	4.44297	2.03579	-3.45410
H	3.20854	0.75871	-3.18587
C	-1.25540	1.89773	0.62092
C	-2.12923	1.06159	1.40234
C	-3.48630	0.94425	1.02052
H	-4.17586	0.35880	1.65123
C	-4.00427	1.65880	-0.08516
H	-5.06867	1.56807	-0.35271
C	-3.16058	2.51796	-0.79957
H	-3.57092	3.09807	-1.64174
C	-1.79767	2.66743	-0.46368
C	-1.63206	0.30902	2.63935
H	-0.54631	0.51574	2.73067
C	-1.79469	-1.21973	2.50461
H	-2.86645	-1.51466	2.44930
H	-1.36620	-1.74204	3.38571
H	-1.27663	-1.60655	1.60168
C	-2.32313	0.81615	3.92471
H	-2.21060	1.91271	4.04885
H	-1.88714	0.32719	4.82110
H	-3.41136	0.59204	3.91916
C	-0.93908	3.67103	-1.23510
H	0.09814	3.56888	-0.85020
C	-1.40628	5.11610	-0.94345
H	-2.43017	5.29858	-1.33488
H	-0.72821	5.84778	-1.43081
H	-1.42214	5.33663	0.14438
C	-0.90097	3.39442	-2.75225
H	-0.45579	2.39819	-2.97318
H	-0.27379	4.15628	-3.26162
H	-1.91449	3.44387	-3.20604
C	-4.77051	-2.07024	-0.53561
H	-5.96895	-0.80940	0.83583
C	-4.39957	-3.56213	-0.66319
H	-3.83578	-3.93692	0.21840
H	-5.32223	-4.18886	-0.74648
H	-4.98450	-1.78404	-2.72544
C	-5.63836	-1.86353	0.71503
H	-6.56835	-2.48037	0.64898
H	-5.12154	-2.16740	1.65137
H	-3.78886	-3.77264	-1.56738
C	-5.55198	-1.62018	-1.78399
H	-5.83062	-0.54504	-1.74562
H	-6.50427	-2.19880	-1.87875
H	-2.72917	-1.55498	-3.23387
O	-1.19838	-1.10012	-0.98213
C	-0.95860	-2.04022	-2.04534
C	-1.62625	-1.64037	-3.35717
H	-1.27262	-3.06587	-1.73330

H	0.14634	-2.10240	-2.22277
H	-1.22464	-0.66416	-3.70015
H	-1.43422	-2.39784	-4.14676

TS(H-I)

SCF (BP86) Energy = -4008.061769
Enthalpy 0K = -4007.262587
Enthalpy 298K = -4007.210237
Free Energy 298K = -4007.351349
Lowest Frequency = -447.1591 cm^{-1}
Second Frequency = 10.9368 cm^{-1}
SCF (BP86-D3^{BJ}) Energy = -4008.297514
SCF ($C_6H_5CH_3$) Energy = -4008.098527
SCF (BS2) Energy = -4010.080696

Zn	-3.06304	-1.08716	-0.61040
H	0.82860	1.35288	-2.83490
Ca	0.71286	0.24064	-0.88303
N	2.64154	0.28584	0.52752
N	-0.01219	1.73940	0.85684
C	2.93309	1.07940	1.56949
C	2.03430	2.01856	2.14882
H	2.44869	2.59001	2.99272
C	0.66449	2.27406	1.88101
C	4.30314	0.97223	2.22500
H	4.45269	-0.04100	2.65360
H	4.43305	1.71651	3.03274
H	5.11286	1.10939	1.47948
C	-0.03243	3.21521	2.85944
H	-0.10430	2.75296	3.86674
H	-1.05495	3.48042	2.52877
H	0.55432	4.14903	2.98044
C	3.58431	-0.68144	0.06402
C	3.54954	-2.01460	0.58791
C	4.42214	-2.98287	0.05230
H	4.39867	-4.00969	0.45323
C	5.31879	-2.66908	-0.97860
H	5.99471	-3.43981	-1.38184
C	5.34085	-1.36731	-1.49635
H	6.03996	-1.12346	-2.31318
C	4.48678	-0.36047	-1.00108
C	2.57069	-2.41503	1.69538
H	2.06094	-1.48812	2.03238
C	3.28211	-3.02465	2.92157
H	3.77992	-3.98632	2.67383
H	2.55514	-3.23021	3.73587
H	4.05912	-2.34264	3.32459
C	1.48388	-3.37344	1.15983
H	0.91856	-2.92732	0.31394
H	0.75368	-3.63269	1.95572
H	1.92816	-4.32180	0.78935
C	4.54323	1.03844	-1.62160
H	3.77347	1.65503	-1.10972
C	5.90898	1.72218	-1.39280
H	6.16633	1.78392	-0.31527
H	5.90376	2.75590	-1.79905
H	6.72874	1.16912	-1.89930
C	4.19665	1.00853	-3.12611
H	4.95685	0.44230	-3.70565
H	4.15936	2.03794	-3.54096
H	3.21247	0.53228	-3.31226
C	-1.40849	1.79364	0.69211
C	-2.26243	0.92270	1.45126
C	-3.62370	0.81202	1.08413
H	-4.29522	0.17700	1.68485
C	-4.15927	1.55391	0.01068
H	-5.22673	1.46568	-0.24531
C	-3.32627	2.42726	-0.70323
H	-3.74988	3.01490	-1.53190
C	-1.96066	2.56774	-0.38565
C	-1.74061	0.09742	2.63033
H	-0.65977	0.32036	2.73737
C	-1.86407	-1.42004	2.37466
H	-2.92731	-1.73619	2.28908
H	-1.42264	-2.00072	3.21194
H	-1.33375	-1.72037	1.44557
C	-2.43648	0.48833	3.95236
H	-2.34711	1.57458	4.15596
H	-1.98674	-0.05829	4.80789
H	-3.51926	0.23989	3.93287
C	-1.07977	3.54802	-1.16037
H	-0.04386	3.13974	-1.12901
C	-1.03825	4.92326	-0.45486
H	-2.04766	5.38752	-0.43541
H	-0.35437	5.61630	-0.98889
H	-0.68507	4.83894	0.59184
C	-1.46910	3.70534	-2.64032
H	-1.52220	2.72505	-3.15626
H	-0.71074	4.31954	-3.16813
H	-2.44612	4.21977	-2.76520
C	-4.74175	-2.17474	-0.75859
H	-6.08003	-0.93540	0.49503
C	-4.30769	-3.65291	-0.83471
H	-3.81601	-3.99824	0.10044
H	-5.19511	-4.31422	-0.99728
H	-4.77063	-1.91369	-2.96153
C	-5.71428	-1.98143	0.41285
H	-6.61871	-2.62505	0.27893
H	-5.26634	-2.26231	1.39092
H	-3.60385	-3.84613	-1.67291
C	-5.42787	-1.76611	-2.07740
H	-5.75153	-0.70264	-2.07403
H	-6.34477	-2.38322	-2.25039
H	0.56848	-1.32956	-3.99316
O	-1.28038	-0.84851	-1.18636
C	-1.01109	-1.48104	-2.49600
C	0.43261	-1.24503	-2.89543
H	-1.68791	-1.02608	-3.25937
H	-1.30363	-2.56147	-2.41345
H	0.73202	0.45322	-2.97995
H	1.11880	-1.99871	-2.42921

I

SCF (BP86) Energy = -4008.066241
Enthalpy 0K = -4007.268723
Enthalpy 298K = -4007.214256
Free Energy 298K = -4007.360031
Lowest Frequency = 13.1796 cm^{-1}

Second Frequency = 17.3503 cm^{-1}
SCF (BP86-$D3^{BJ}$) Energy = -4008.301926
SCF ($C_6H_5CH_3$) Energy = -4008.104839
SCF (BS2) Energy = -4010.089744

Zn	-3.03434	-1.21584	-0.70026
H	1.73158	2.67935	-1.49356
Ca	0.66526	0.17008	-0.85777
N	2.68595	0.31374	0.43398
N	-0.01673	1.67986	0.87507
C	3.02561	1.24102	1.34086
C	2.14734	2.24545	1.84442
H	2.61748	2.94788	2.54797
C	0.74653	2.42348	1.68735
C	4.43191	1.22902	1.92508
H	4.62642	0.26552	2.44177
H	4.58591	2.05174	2.64799
H	5.19932	1.30757	1.12799
C	0.11617	3.55174	2.49547
H	0.67280	3.74726	3.43239
H	-0.94307	3.33833	2.74071
H	0.12620	4.49270	1.90260
C	3.60559	-0.70346	0.03730
C	3.52156	-2.00053	0.63931
C	4.35757	-3.03255	0.16680
H	4.29599	-4.03068	0.63127
C	5.26464	-2.81673	-0.87911
H	5.90974	-3.63563	-1.23502
C	5.33862	-1.54850	-1.47121
H	6.04672	-1.38118	-2.29930
C	4.52703	-0.48063	-1.03760
C	2.53658	-2.29410	1.77356
H	2.01960	-1.33973	2.00968
C	3.24865	-2.76336	3.05979
H	3.76865	-3.73370	2.91057
H	2.51977	-2.90160	3.88655
H	4.00785	-2.02745	3.39596
C	1.46247	-3.31332	1.33466
H	0.89554	-2.96212	0.44537
H	0.73060	-3.49778	2.14917
H	1.91590	-4.29076	1.06479
C	4.63172	0.87199	-1.74734
H	4.00903	1.59166	-1.17633
C	6.07352	1.42260	-1.76602
H	6.50530	1.48705	-0.74545
H	6.09624	2.44055	-2.20947
H	6.75068	0.78512	-2.37335
C	4.05566	0.79499	-3.17873
H	4.63934	0.08847	-3.80706
H	4.08957	1.78969	-3.67271
H	3.00021	0.44582	-3.18246
C	-1.42955	1.69935	0.84938
C	-2.13983	0.68150	1.57583
C	-3.52902	0.53852	1.37181
H	-4.08587	-0.21785	1.94676
C	-4.23088	1.38165	0.48649
H	-5.31730	1.26191	0.35152
C	-3.53693	2.38866	-0.19550
H	-4.08853	3.05464	-0.87904
C	-2.14696	2.56828	-0.03287
C	-1.41119	-0.21332	2.57974
H	-0.34158	-0.22190	2.27544
C	-1.91359	-1.66837	2.58981
H	-2.95296	-1.75442	2.97146
H	-1.27695	-2.29425	3.24882
H	-1.88357	-2.12244	1.57460
C	-1.47412	0.38582	4.00375
H	-1.05063	1.40882	4.03787
H	-0.90212	-0.24071	4.72048
H	-2.52421	0.44288	4.36289
C	-1.43777	3.66595	-0.82624
H	-0.36966	3.64394	-0.52538
C	-1.98878	5.06789	-0.48777
H	-3.04944	5.17716	-0.79963
H	-1.40758	5.85403	-1.01450
H	-1.93840	5.27595	0.60077
C	-1.49622	3.39981	-2.34590
H	-1.07355	2.40700	-2.60819
H	-0.92392	4.17169	-2.90275
H	-2.54150	3.42386	-2.72167
C	-4.73515	-2.26250	-0.81287
H	-5.87545	-1.31443	0.82810
C	-4.32835	-3.69030	-1.23373
H	-3.71394	-4.20037	-0.46050
H	-5.23590	-4.32364	-1.39616
H	-5.07040	-1.58113	-2.89706
C	-5.52724	-2.31710	0.50042
H	-6.44236	-2.94767	0.37684
H	-4.94094	-2.76201	1.33293
H	-3.75093	-3.70585	-2.18339
C	-5.59621	-1.62117	-1.91844
H	-5.90805	-0.58547	-1.66298
H	-6.53218	-2.21242	-2.07891
H	0.05663	0.48096	-3.96092
O	-1.30838	-0.80614	-1.28565
C	-1.10282	-0.96779	-2.80111
C	0.19008	-0.35343	-3.24138
H	-1.99727	-0.49163	-3.26899
H	-1.16199	-2.06669	-2.98386
H	1.55306	2.37682	-2.17971
H	0.89790	-1.09091	-3.67604

J

SCF (BP86) Energy = -4008.066930
Enthalpy 0K = -4007.268992
Enthalpy 298K = -4007.214650
Free Energy 298K = -4007.360125
Lowest Frequency = 6.2993 cm-1
Second Frequency = 15.7983 cm-1
SCF (BP86-D3BJ) Energy = -4008.291911
SCF (C6H5CH3) Energy = -4008.108631
SCF (Et2O) Energy = N/A
SCF (BS2) Energy = -4010.093736

Zn	2.04498	-2.55930	-0.91863
H	-1.89125	2.77864	-1.83925
Ca	-0.44994	0.48460	-0.91820
N	0.30901	2.18211	0.58466
N	-2.31620	0.48053	0.57373

C	-0.44759	2.85862	1.46527
C	-1.79187	2.53926	1.80387
H	-2.24204	3.22810	2.53262
C	-2.62053	1.42881	1.47358
C	0.16068	4.04461	2.20228
H	1.01375	3.71806	2.83324
H	-0.57877	4.55034	2.85072
H	0.57588	4.78560	1.48853
C	-3.92730	1.32255	2.24847
H	-3.94324	0.39120	2.85294
H	-4.79714	1.25583	1.56382
H	-4.07457	2.18240	2.92805
C	1.69972	2.46941	0.44300
C	2.65193	1.76586	1.25130
C	4.02791	1.96128	1.01777
H	4.76210	1.42183	1.63929
C	4.48413	2.82928	0.01585
H	5.56401	2.96701	-0.15277
C	3.55079	3.52525	-0.76377
H	3.90891	4.21540	-1.54542
C	2.16262	3.36997	-0.56947
C	2.20979	0.81018	2.36292
H	1.10051	0.82766	2.38986
C	2.71799	1.25490	3.75153
H	3.82685	1.22392	3.81169
H	2.32221	0.58993	4.54833
H	2.40178	2.29152	3.98811
C	2.64215	-0.64244	2.07379
H	2.23466	-0.99680	1.10008
H	2.27754	-1.33355	2.86281
H	3.74761	-0.73929	2.02491
C	1.19254	4.17226	-1.44032
H	0.16890	3.95817	-1.06763
C	1.42066	5.69427	-1.31581
H	1.37223	6.03363	-0.26027
H	0.65052	6.25325	-1.88841
H	2.41252	5.99451	-1.71584
C	1.25327	3.72988	-2.91787
H	2.26098	3.90730	-3.35094
H	0.52053	4.29640	-3.53108
H	1.02546	2.64949	-3.03469
C	-3.16965	-0.64864	0.38249
C	-2.88703	-1.87016	1.07551
C	-3.66314	-3.01315	0.79635
H	-3.44810	-3.95376	1.33028
C	-4.70383	-2.97917	-0.14156
H	-5.29885	-3.88326	-0.34677
C	-4.98068	-1.78123	-0.81393
H	-5.80092	-1.75358	-1.55009
C	-4.23710	-0.60785	-0.57236
C	-1.76612	-1.96282	2.11426
H	-1.31186	-0.95278	2.19498
C	-0.65580	-2.93808	1.66739
H	-1.04650	-3.96957	1.53545
H	0.16029	-2.98274	2.41952
H	-0.21416	-2.62395	0.69610
C	-2.29932	-2.34476	3.51180
H	-3.08491	-1.64017	3.85430
H	-1.48146	-2.33265	4.26338
H	-2.74143	-3.36393	3.51744

C	-4.58949	0.66740	-1.34340
H	-3.93951	1.47738	-0.95152
C	-6.05531	1.10003	-1.12273
H	-6.76884	0.35767	-1.53950
H	-6.25641	2.06950	-1.62587
H	-6.29477	1.22102	-0.04580
C	-4.28884	0.51803	-2.85028
H	-3.22609	0.25487	-3.03155
H	-4.50294	1.46412	-3.39169
H	-4.91352	-0.27857	-3.30828
C	3.36083	-3.98565	-0.53495
H	2.13755	-4.97575	1.02434
C	4.77828	-3.39778	-0.67097
H	4.97172	-2.58577	0.06193
H	5.54388	-4.19140	-0.48815
H	3.28806	-4.74388	-2.61949
C	3.14959	-4.53939	0.88495
H	3.88638	-5.35352	1.09436
H	3.29387	-3.76416	1.66714
H	4.97351	-2.98892	-1.68569
C	3.14953	-5.10248	-1.57673
H	2.13977	-5.56143	-1.50884
H	3.89025	-5.92409	-1.41619
H	-1.22134	-0.36611	-3.83587
O	0.84284	-1.27658	-1.45644
C	0.60679	-1.20774	-3.00434
C	-0.26467	-0.06166	-3.36455
H	0.17267	-2.20342	-3.25016
H	1.63451	-1.14857	-3.43153
H	-1.66628	2.33781	-2.43225
H	0.23687	0.69701	-3.99896

TS(J-K)

SCF (BP86) Energy = -4008.066315
Enthalpy 0K = -4007.269723
Enthalpy 298K = -4007.215303
Free Energy 298K = -4007.362002
Lowest Frequency = -264.3459 cm^{-1}
Second Frequency = 5.8805 cm^{-1}
SCF (BP86-D3^{BJ}) Energy = -4008.291409
SCF ($C_6H_5CH_3$) Energy = -4008.106592
SCF (Et2O) Energy = N/A
SCF (BS2) Energy = -4010.094442

Zn	2.24461	-2.38766	-0.88240
H	-2.04396	2.77631	-1.51467
Ca	-0.48197	0.38362	-0.90866
N	0.21482	2.14071	0.55500
N	-2.38236	0.39242	0.54051
C	-0.58017	2.84015	1.38251
C	-1.92671	2.50724	1.69919
H	-2.40692	3.21343	2.39126
C	-2.72144	1.36137	1.40375
C	-0.01728	4.07045	2.08229
H	0.82690	3.79177	2.74705
H	-0.78410	4.58598	2.68993
H	0.39869	4.78994	1.34723
C	-4.02724	1.24334	2.17869
H	-4.01899	0.32619	2.80479

H	-4.89629	1.13971	1.49819
H	-4.19493	2.11498	2.83822
C	1.60750	2.43851	0.45706
C	2.53366	1.77162	1.32456
C	3.91571	1.97637	1.13942
H	4.62999	1.46608	1.80698
C	4.40273	2.81686	0.12852
H	5.48702	2.96151	-0.00160
C	3.49419	3.47707	-0.70937
H	3.87614	4.14633	-1.49787
C	2.10118	3.31237	-0.56393
C	2.05810	0.84889	2.45018
H	0.94932	0.82596	2.40925
C	2.46353	1.37637	3.84394
H	3.56741	1.39630	3.96793
H	2.04880	0.73031	4.64664
H	2.09181	2.40749	4.01448
C	2.55852	-0.59743	2.25795
H	2.23682	-1.00636	1.27393
H	2.15801	-1.26780	3.04740
H	3.66695	-0.65779	2.29679
C	1.15674	4.07373	-1.49725
H	0.12368	3.88098	-1.14050
C	1.38645	5.59941	-1.44638
H	1.31729	5.99165	-0.41052
H	0.62946	6.12957	-2.06228
H	2.38667	5.87850	-1.84078
C	1.25107	3.55551	-2.94811
H	2.26994	3.70550	-3.36495
H	0.53550	4.08997	-3.60870
H	1.02282	2.47088	-3.01395
C	-3.21236	-0.75577	0.35819
C	-2.88144	-1.97524	1.03279
C	-3.63017	-3.13721	0.75634
H	-3.37792	-4.07620	1.27648
C	-4.68951	-3.12380	-0.16057
H	-5.26161	-4.04278	-0.36481
C	-5.01590	-1.92673	-0.81232
H	-5.85204	-1.91541	-1.53078
C	-4.30239	-0.73466	-0.57258
C	-1.74746	-2.04363	2.05924
H	-1.29709	-1.02964	2.11405
C	-0.63785	-3.02532	1.62568
H	-1.02550	-4.06101	1.52030
H	0.18057	-3.05159	2.37637
H	-0.19971	-2.72921	0.64788
C	-2.27120	-2.39259	3.46932
H	-3.05248	-1.67868	3.80245
H	-1.44763	-2.36689	4.21434
H	-2.71620	-3.41016	3.49893
C	-4.70973	0.53758	-1.32234
H	-4.09928	1.36985	-0.91527
C	-6.19502	0.89919	-1.10468
H	-6.87229	0.13863	-1.54805
H	-6.43403	1.87204	-1.58411
H	-6.44938	0.98037	-0.02740
C	-4.39533	0.43138	-2.82994
H	-3.32012	0.22511	-3.00912
H	-4.65430	1.37503	-3.35597
H	-4.97549	-0.38815	-3.30565

C	3.73090	-3.64809	-0.54882
H	2.59288	-4.92323	0.86173
C	5.06078	-2.87105	-0.56362
H	5.11074	-2.10341	0.23767
H	5.91891	-3.56908	-0.40152
H	3.85020	-4.23064	-2.68706
C	3.54059	-4.34563	0.80985
H	4.37266	-5.06963	0.99280
H	3.54490	-3.62809	1.65778
H	5.24137	-2.35682	-1.53211
C	3.71760	-4.69133	-1.68428
H	2.77716	-5.28315	-1.70688
H	4.55508	-5.41980	-1.55030
H	-1.40107	-0.51615	-3.71128
O	0.88604	-1.27745	-1.37386
C	0.54131	-1.23471	-3.05397
C	-0.39503	-0.18780	-3.38994
H	0.18206	-2.27545	-3.17611
H	1.57081	-1.11252	-3.44656
H	-1.93600	2.49978	-2.22493
H	-0.00378	0.65598	-3.98784

K

SCF (BP86) Energy = -4008.091810
Enthalpy 0K = -4007.297587
Enthalpy 298K = -4007.239657
Free Energy 298K = -4007.406271
Lowest Frequency = 1.7409 cm^{-1}
Second Frequency = 3.6708 cm^{-1}
SCF (BP86-D3^{BJ}) Energy = -4008.311470
SCF ($C_6H_5CH_3$) Energy = -4008.129899
SCF (BS2) Energy = -4010.124712

Zn	-3.26805	-1.60978	0.39352
H	-0.46333	-5.42995	7.37268
Ca	0.20745	0.00521	0.39604
N	0.45057	2.09823	-0.70107
N	2.05713	-0.47538	-1.00716
C	1.18156	2.41175	-1.77847
C	2.09254	1.52137	-2.41662
H	2.58347	1.93673	-3.30892
C	2.53086	0.22133	-2.05376
C	1.06670	3.79457	-2.40998
H	0.31690	3.78396	-3.23036
H	2.03057	4.11132	-2.85406
H	0.73656	4.55718	-1.67787
C	3.62477	-0.39266	-2.91620
H	4.56407	-0.52078	-2.33881
H	3.84712	0.22993	-3.80306
H	3.33130	-1.40731	-3.25655
C	-0.43815	3.01536	-0.07529
C	-1.73951	3.28004	-0.61212
C	-2.61566	4.11090	0.11549
H	-3.61789	4.32033	-0.29307
C	-2.24854	4.66984	1.34710
H	-2.95179	5.31612	1.89591
C	-0.98063	4.39393	1.87861
H	-0.69581	4.83304	2.84762
C	-0.06383	3.57116	1.19406

C	-2.21846	2.65713	-1.92619
H	-1.35238	2.13604	-2.38370
C	-2.71568	3.71552	-2.93355
H	-3.63409	4.22602	-2.57298
H	-2.96586	3.24106	-3.90598
H	-1.95261	4.49905	-3.12066
C	-3.30436	1.58828	-1.67134
H	-2.92943	0.79174	-0.99328
H	-3.61637	1.11208	-2.62558
H	-4.20895	2.03787	-1.20809
C	1.33407	3.32299	1.76834
H	1.68899	2.35353	1.34647
C	2.33500	4.39329	1.27783
H	2.38772	4.42469	0.17152
H	3.35730	4.18898	1.66159
H	2.03159	5.40194	1.63138
C	1.36630	3.21213	3.30398
H	1.15020	4.18403	3.79593
H	2.37296	2.89501	3.64790
H	0.62613	2.47635	3.68249
C	2.66994	-1.67570	-0.55366
C	2.05601	-2.94339	-0.82276
C	2.57867	-4.09934	-0.20528
H	2.10862	-5.07538	-0.40989
C	3.68826	-4.03787	0.64873
H	4.07901	-4.95342	1.12060
C	4.30563	-2.80037	0.88462
H	5.18812	-2.75608	1.54421
C	3.82457	-1.61268	0.29872
C	0.89338	-3.08104	-1.81061
H	0.60381	-2.05271	-2.11726
C	-0.34899	-3.74961	-1.18671
H	-0.12130	-4.76733	-0.80349
H	-1.14974	-3.85722	-1.94943
H	-0.76180	-3.13560	-0.35790
C	1.34045	-3.83254	-3.08540
H	2.22148	-3.35257	-3.55958
H	0.52112	-3.85625	-3.83492
H	1.61787	-4.88419	-2.85805
C	4.53405	-0.28703	0.59686
H	4.09204	0.48428	-0.06635
C	6.04624	-0.34974	0.29374
H	6.57655	-1.04960	0.97410
H	6.51234	0.65011	0.42171
H	6.24292	-0.68583	-0.74538
C	4.29310	0.17847	2.04899
H	3.21095	0.30212	2.26359
H	4.78812	1.15418	2.24074
H	4.69566	-0.55327	2.78223
C	-5.08463	-2.38188	0.35434
H	-4.82592	-3.49048	2.25700
C	-5.47882	-2.70402	-1.09975
H	-4.79334	-3.44161	-1.57038
H	-6.50656	-3.14459	-1.13976
H	-6.10907	-0.41671	0.37193
C	-5.09645	-3.67384	1.19447
H	-6.11660	-4.13358	1.19489
H	-4.39697	-4.44200	0.79937
H	-5.49018	-1.79917	-1.74493
C	-6.07882	-1.36592	0.94914
H	-5.84044	-1.10779	2.00378
H	-7.11717	-1.78281	0.94413
H	-0.53144	-5.10233	6.68205
O	-1.63070	-0.92988	0.47333
C	-0.05130	-0.92316	3.27718
C	1.04393	-1.64686	2.96558
H	-0.99442	-1.05437	2.70909
H	-0.03313	-0.17976	4.09324
H	1.99664	-1.53535	3.50918
H	1.02761	-2.41400	2.16899

L

SCF (BP86) Energy = -4006.919888
Enthalpy 0K = -4006.135579
Enthalpy 298K = -4006.082164
Free Energy 298K = -4006.229430
Lowest Frequency = 8.2224 cm^{-1}
Second Frequency = 11.5117 cm^{-1}
SCF (BP86-D3^{BJ}) Energy = -4007.138440
SCF ($C_6H_5CH_3$) Energy = -4006.959092
SCF (BS2) Energy = -4008.947682

Zn	-3.16218	-1.76351	-0.55185
Ca	0.22339	0.02505	-0.42701
N	2.13725	-0.42957	0.89742
N	0.40519	2.07767	0.75121
C	2.64833	0.26702	1.92536
C	2.18141	1.54131	2.34269
H	2.71075	1.95968	3.21113
C	1.18005	2.39431	1.79762
C	3.81545	-0.31863	2.70734
H	3.56873	-1.33632	3.07492
H	4.09111	0.31401	3.57173
H	4.71002	-0.43367	2.06042
C	1.01031	3.73849	2.49631
H	0.63554	4.51895	1.80536
H	1.96312	4.07917	2.94599
H	0.26973	3.65413	3.32081
C	2.73492	-1.62960	0.41968
C	2.17627	-2.90191	0.77318
C	2.66684	-4.06172	0.13793
H	2.23731	-5.04130	0.40529
C	3.69252	-3.99938	-0.81521
H	4.05867	-4.91849	-1.29984
C	4.25973	-2.75637	-1.13380
H	5.08059	-2.71081	-1.86877
C	3.80827	-1.56463	-0.53171
C	1.10445	-3.03694	1.85926
H	0.84947	-2.00845	2.19361
C	1.65347	-3.79885	3.08694
H	1.89635	-4.85328	2.83443
H	0.90297	-3.81572	3.90555
H	2.57896	-3.33185	3.48340
C	-0.19267	-3.69277	1.34097
H	-0.66951	-3.07284	0.55144
H	-0.92559	-3.80042	2.16922
H	-0.00374	-4.70936	0.93393
C	4.47162	-0.23500	-0.90767
H	4.04944	0.54568	-0.24201

C	5.99887	-0.26177	-0.68424
H	6.25660	-0.55904	0.35329
H	6.43956	0.74059	-0.87015
H	6.50378	-0.97650	-1.36849
C	4.14522	0.18192	-2.35764
H	4.52140	-0.56442	-3.08998
H	4.61095	1.15969	-2.60453
H	3.05126	0.27983	-2.51725
C	-0.57870	2.96036	0.22702
C	-1.86242	3.09915	0.84700
C	-2.83289	3.90752	0.22068
H	-3.82196	4.02112	0.69404
C	-2.57474	4.56297	-0.99068
H	-3.34962	5.19007	-1.45949
C	-1.32464	4.40670	-1.60681
H	-1.12814	4.91905	-2.56152
C	-0.31682	3.61142	-1.02549
C	-2.22196	2.36401	2.14108
H	-1.29592	1.88437	2.52005
C	-3.23983	1.23276	1.87380
H	-4.19905	1.64036	1.48789
H	-3.46285	0.67798	2.81036
H	-2.84904	0.50730	1.12804
C	-2.73869	3.31897	3.23803
H	-2.02627	4.14657	3.43543
H	-2.89822	2.76948	4.18982
H	-3.71103	3.77785	2.95853
C	1.05582	3.48432	-1.69396
H	1.47116	2.49368	-1.39186
C	2.04384	4.54330	-1.15522
H	1.67922	5.56737	-1.38467
H	3.04628	4.42478	-1.61906
H	2.16770	4.46530	-0.05733
C	1.00633	3.52751	-3.23243
H	0.26804	2.80922	-3.64582
H	2.00190	3.28232	-3.65787
H	0.73637	4.53627	-3.61047
C	-4.95083	-2.59585	-0.62334
H	-5.62632	-1.38869	-2.35546
C	-5.43716	-2.89564	0.80742
H	-5.52341	-1.97614	1.42564
H	-6.45044	-3.36981	0.78680
H	-4.16730	-4.64151	-0.96354
C	-5.93157	-1.62847	-1.31380
H	-6.95405	-2.07932	-1.37105
H	-6.03266	-0.66687	-0.76589
H	-4.76344	-3.59761	1.34475
C	-4.86106	-3.90739	-1.42749
H	-4.52115	-3.74067	-2.47270
H	-5.86321	-4.40149	-1.48787
O	-1.54662	-1.03138	-0.52310
C	0.34990	-0.43817	-3.54093
C	0.57109	-1.61220	-2.91420
H	1.12084	0.05550	-4.15679
H	-0.63902	0.05332	-3.49052
H	-0.23139	-2.07180	-2.30633
H	1.54465	-2.12883	-2.96889

TS(L-M)

SCF (BP86) Energy = -4006.914150
Enthalpy 0K = -4006.131160
Enthalpy 298K = -4006.077516
Free Energy 298K = -4006.231113
Lowest Frequency = -10.7286 cm^{-1}
Second Frequency = 5.8754 cm^{-1}
SCF (BP86-D3^{BJ}) Energy = -4007.121740
SCF ($C_6H_5CH_3$) Energy = -4006.957566
SCF (BS2) Energy = -4008.943425

Zn	2.14659	-2.60609	0.28379
Ca	-0.39256	0.23286	0.48586
N	-2.61877	0.23615	-0.27708
N	-0.42605	2.35526	-0.53267
C	-3.30637	1.21334	-0.88940
C	-2.76145	2.47533	-1.25471
H	-3.47435	3.15397	-1.74403
C	-1.44957	3.00496	-1.10997
C	-4.77244	0.98280	-1.22594
H	-4.88652	0.11897	-1.91390
H	-5.23265	1.87108	-1.69736
H	-5.35023	0.72381	-0.31386
C	-1.21839	4.40714	-1.65417
H	-0.83965	5.08222	-0.85816
H	-2.14181	4.84287	-2.07901
H	-0.43689	4.39794	-2.44269
C	-3.22507	-1.01411	0.04127
C	-3.18150	-2.09645	-0.89446
C	-3.69157	-3.35105	-0.50409
H	-3.66081	-4.18916	-1.21946
C	-4.23043	-3.55976	0.77341
H	-4.62410	-4.54919	1.05538
C	-4.25688	-2.50083	1.69191
H	-4.67280	-2.66940	2.69873
C	-3.76183	-1.22575	1.35202
C	-2.55308	-1.92881	-2.27996
H	-2.34843	-0.84648	-2.41750
C	-3.49663	-2.36950	-3.41809
H	-3.70623	-3.45994	-3.38318
H	-3.03992	-2.15546	-4.40745
H	-4.47190	-1.84127	-3.37287
C	-1.19818	-2.66856	-2.36443
H	-0.48870	-2.32962	-1.57792
H	-0.72056	-2.49975	-3.35332
H	-1.33530	-3.76462	-2.24120
C	-3.75244	-0.10366	2.39364
H	-3.53799	0.84150	1.85158
C	-5.10389	0.07553	3.11285
H	-5.92843	0.23582	2.38833
H	-5.07275	0.95268	3.79323
H	-5.36791	-0.80902	3.73043
C	-2.61577	-0.31858	3.41935
H	-2.78078	-1.24092	4.01567
H	-2.53339	0.53536	4.12473
H	-1.62248	-0.45193	2.93169
C	0.86975	2.94109	-0.43722
C	1.83853	2.71974	-1.46750
C	3.14536	3.21656	-1.28753
H	3.89619	3.05010	-2.07733
C	3.51338	3.91263	-0.12715

H	4.54018	4.29381	-0.00974
C	2.56440	4.11537	0.88533
H	2.85800	4.65635	1.79970
C	1.24371	3.64102	0.75524
C	1.49534	1.92473	-2.72953
H	0.40025	1.74333	-2.71301
C	2.19248	0.54493	-2.72273
H	3.29806	0.65750	-2.74292
H	1.90032	-0.04489	-3.61791
H	1.93226	-0.04990	-1.81970
C	1.81569	2.70146	-4.02357
H	1.31480	3.69184	-4.04476
H	1.47771	2.13069	-4.91421
H	2.90639	2.87701	-4.14121
C	0.24441	3.82827	1.90007
H	-0.76304	3.59833	1.49394
C	0.20259	5.26876	2.44690
H	1.15893	5.55916	2.93130
H	-0.59661	5.37486	3.21059
H	0.00087	6.00164	1.63898
C	0.52391	2.82289	3.04062
H	0.55286	1.76897	2.67974
H	-0.24686	2.88376	3.83796
H	1.51485	3.00814	3.50666
C	3.38802	-4.13861	0.21260
H	5.15479	-2.90606	-0.29911
C	2.65040	-5.36200	-0.36444
H	2.28332	-5.18524	-1.39857
H	3.33318	-6.24748	-0.40923
H	3.04509	-4.71904	2.32440
C	4.58925	-3.78197	-0.68346
H	5.30825	-4.63793	-0.73555
H	4.28390	-3.55340	-1.72757
H	1.77593	-5.65968	0.25389
C	3.87877	-4.45136	1.63935
H	4.42573	-3.59677	2.09250
H	4.58323	-5.32088	1.63104
O	1.01091	-1.24567	0.36254
C	6.67427	-0.29675	1.58920
C	5.46709	0.10608	2.01839
H	7.34448	-0.90243	2.22256
H	7.04759	-0.03860	0.58367
H	4.79663	0.71158	1.38572
H	5.09589	-0.15242	3.02476

M

SCF (BP86) Energy = -4006.914187
Enthalpy 0K = -4006.131114
Enthalpy 298K = -4006.076571
Free Energy 298K = -4006.234916
Lowest Frequency = 2.5861 cm-1
Second Frequency = 7.5088 cm-1
SCF (BP86-D3BJ) Energy = -4007.120659
SCF (C6H5CH3) Energy = -4006.957544
SCF (Et2O) Energy = N/A
SCF (BS2) Energy = -4008.943619

Zn	1.98306	2.72860	-0.31424
Ca	-0.43579	-0.21621	-0.49008
N	-2.65410	-0.37001	0.27896
N	-0.31528	-2.32311	0.55434
C	-3.26813	-1.38352	0.90987
C	-2.63359	-2.59851	1.28968
H	-3.29454	-3.31907	1.79181
C	-1.28868	-3.03675	1.14306
C	-4.74512	-1.25172	1.25153
H	-4.91643	-0.38740	1.92691
H	-5.13929	-2.16273	1.73914
H	-5.34409	-1.04828	0.33903
C	-0.95857	-4.41494	1.69745
H	-0.54261	-5.07011	0.90331
H	-1.84684	-4.90794	2.13446
H	-0.17244	-4.34642	2.47832
C	-3.34805	0.82924	-0.05629
C	-3.37720	1.92743	0.86148
C	-3.97461	3.13678	0.45253
H	-4.00007	3.98681	1.15392
C	-4.53064	3.28623	-0.82589
H	-4.99301	4.24109	-1.12237
C	-4.48624	2.21285	-1.72673
H	-4.91603	2.33522	-2.73441
C	-3.90245	0.98119	-1.36805
C	-2.73534	1.82737	2.24733
H	-2.45505	0.76449	2.40201
C	-3.70554	2.21957	3.38083
H	-3.99079	3.29202	3.32886
H	-3.23337	2.05411	4.37225
H	-4.64169	1.62395	3.34687
C	-1.43546	2.66159	2.31568
H	-0.70369	2.35881	1.53481
H	-0.94740	2.54506	3.30704
H	-1.64930	3.74288	2.17255
C	-3.81852	-0.15459	-2.39122
H	-3.53461	-1.07295	-1.83524
C	-5.15761	-0.44100	-3.09883
H	-5.96495	-0.64697	-2.36653
H	-5.06802	-1.32503	-3.76497
H	-5.48684	0.41226	-3.72901
C	-2.70541	0.12365	-3.42741
H	-2.93805	1.02286	-4.03636
H	-2.56702	-0.73317	-4.12044
H	-1.72128	0.33342	-2.94817
C	1.01676	-2.82035	0.45595
C	1.97120	-2.53599	1.48419
C	3.30726	-2.94609	1.30119
H	4.04732	-2.73116	2.08940
C	3.71775	-3.61639	0.13967
H	4.76676	-3.93090	0.02077
C	2.78196	-3.88045	-0.87101
H	3.10809	-4.40137	-1.78607
C	1.43331	-3.49376	-0.73761
C	1.57929	-1.76363	2.74611
H	0.47382	-1.66293	2.73711
C	2.17278	-0.33629	2.72816
H	3.28377	-0.36727	2.73965
H	1.84468	0.23482	3.62301
H	1.86254	0.23306	1.82458
C	1.96525	-2.50838	4.04094
H	1.54060	-3.53363	4.06924

H	1.59055	-1.96079	4.93142
H	3.06672	-2.60059	4.15269
C	0.44570	-3.74709	-1.87974
H	-0.57310	-3.57653	-1.47240
C	0.49015	-5.19046	-2.41874
H	1.46235	-5.42559	-2.90156
H	-0.30117	-5.34846	-3.18168
H	0.33255	-5.92960	-1.60675
C	0.66240	-2.73270	-3.02594
H	0.63267	-1.67709	-2.66998
H	-0.10608	-2.84183	-3.82033
H	1.66080	-2.86229	-3.49487
C	3.22110	4.26484	-0.27370
H	5.00034	3.03943	0.21831
C	2.49178	5.49126	0.30729
H	2.14197	5.32088	1.34844
H	3.17411	6.37777	0.33499
H	2.84233	4.83101	-2.38328
C	4.43773	3.91631	0.60486
H	5.15541	4.77424	0.63971
H	4.15000	3.69403	1.65526
H	1.60689	5.78386	-0.29848
C	3.68762	4.56871	-1.71056
H	4.22811	3.71234	-2.16841
H	4.39120	5.43888	-1.71974
O	0.86480	1.35347	-0.37106
C	7.16145	0.29939	-1.32997
C	6.07717	-0.21952	-1.92836
H	7.88955	0.91841	-1.88119
H	7.37047	0.12897	-0.26021
H	5.34856	-0.83800	-1.37745
H	5.86986	-0.04802	-2.99844

N

SCF (BP86) Energy = -3928.389531
Enthalpy 0K = -3927.656127
Enthalpy 298K = -3927.607176
Free Energy 298K = -3927.745022
Lowest Frequency = 5.8068 cm^{-1}
Second Frequency = 9.1065 cm^{-1}
SCF (BP86-D3^{BJ}) Energy = -3928.589964
SCF ($C_6H_5CH_3$) Energy = -3928.432118
SCF (BS2) Energy = -3930.328570

Zn	-0.26999	3.52115	-0.68284
Ca	0.02184	-0.28081	-0.56030
N	-1.39362	-1.84587	0.48048
N	1.65857	-1.61226	0.48159
C	-1.07405	-2.93749	1.19404
C	0.25516	-3.33689	1.50475
H	0.32580	-4.26227	2.09371
C	1.50831	-2.73943	1.19556
C	-2.19134	-3.82921	1.71581
H	-2.86223	-3.26203	2.39453
H	-1.79834	-4.70692	2.26193
H	-2.83252	-4.18600	0.88247
C	2.74782	-3.44957	1.71999
H	3.43725	-3.70563	0.88815
H	2.49260	-4.37612	2.26720
H	3.32322	-2.78552	2.39855
C	-2.75121	-1.49699	0.22075
C	-3.46145	-0.63963	1.12043
C	-4.76412	-0.22579	0.77611
H	-5.31681	0.43468	1.46422
C	-5.37036	-0.62852	-0.42225
H	-6.39012	-0.29292	-0.66910
C	-4.66491	-1.45559	-1.30770
H	-5.13990	-1.76383	-2.25346
C	-3.36070	-1.89986	-1.01106
C	-2.82029	-0.13255	2.41440
H	-1.86403	-0.68163	2.54232
C	-3.68761	-0.41778	3.65796
H	-4.64584	0.14377	3.63170
H	-3.15439	-0.11257	4.58303
H	-3.93583	-1.49590	3.74911
C	-2.47796	1.37140	2.30911
H	-1.81074	1.58714	1.44588
H	-1.96896	1.72241	3.23231
H	-3.39889	1.98072	2.18212
C	-2.59318	-2.75596	-2.02206
H	-1.70642	-3.16597	-1.49418
C	-3.40636	-3.95265	-2.55346
H	-3.78021	-4.58679	-1.72364
H	-2.78079	-4.58957	-3.21395
H	-4.28578	-3.62756	-3.14887
C	-2.07618	-1.88819	-3.19240
H	-2.91708	-1.46094	-3.77878
H	-1.43727	-2.47445	-3.88639
H	-1.48085	-1.01356	-2.84203
C	2.94739	-1.06121	0.22195
C	3.51676	-0.10236	1.11926
C	4.74123	0.50491	0.77469
H	5.18534	1.24442	1.46104
C	5.40367	0.19570	-0.42168
H	6.36029	0.68273	-0.66894
C	4.83453	-0.73279	-1.30474
H	5.35256	-0.96786	-2.24891
C	3.61324	-1.37044	-1.00780
C	2.80434	0.30410	2.41147
H	1.94106	-0.38219	2.53848
C	2.24029	1.73948	2.30423
H	3.05931	2.48013	2.17716
H	1.68363	2.01101	3.22672
H	1.54882	1.85139	1.44039
C	3.70278	0.15302	3.65651
H	4.10947	-0.87571	3.74912
H	3.12861	0.37579	4.58060
H	4.56626	0.85159	3.63072
C	2.98729	-2.33757	-2.01621
H	2.17540	-2.87933	-1.48672
C	3.97592	-3.39459	-2.54644
H	4.79303	-2.93839	-3.14459
H	3.45582	-4.12274	-3.20411
H	4.44527	-3.96072	-1.71597
C	2.34084	-1.56325	-3.18775
H	1.61719	-0.79097	-2.83845
H	1.79991	-2.24311	-3.87966
H	3.10473	-1.01204	-3.77607
C	-0.42073	5.48577	-0.79053

H	0.87569	5.65202	-2.58074
C	-0.46949	6.07459	0.63228
H	0.44597	5.84162	1.21792
H	-0.55536	7.18962	0.59383
H	-2.62309	5.47249	-1.03971
C	0.80201	6.04468	-1.54340
H	0.74030	7.15920	-1.62198
H	1.75890	5.80986	-1.02895
H	-1.34115	5.70387	1.21373
C	-1.71093	5.85124	-1.54957
H	-1.71854	5.45226	-2.58710
H	-1.82021	6.96209	-1.62833
O	-0.13464	1.75548	-0.60790

O

SCF (BP86) Energy = -2148.983276
Enthalpy 0K = -2148.249408
Enthalpy 298K = -2148.202644
Free Energy 298K = -2148.335075
Lowest Frequency = 8.2185 cm^{-1}
Second Frequency = 12.2996 cm^{-1}
SCF (BP86-D3^{BJ}) Energy = -2149.180174
SCF ($C_6H_5CH_3$) Energy = -2149.019464
SCF (BS2) Energy = -2150.657071

Ca	0.00020	0.56481	-0.19566
N	-1.53633	-1.17197	0.23512
N	1.53928	-1.16932	0.23798
C	-1.29381	-2.46035	0.53025
C	0.00238	-3.02973	0.66265
H	0.00306	-4.10136	0.90688
C	1.29787	-2.45776	0.53439
C	-2.47115	-3.40137	0.74040
H	-3.11797	-3.04480	1.56925
H	-2.14028	-4.43153	0.96884
H	-3.11934	-3.42795	-0.16065
C	2.47612	-3.39624	0.75100
H	3.12715	-3.42499	-0.14793
H	2.14614	-4.42613	0.98196
H	3.11991	-3.03577	1.58053
C	-2.87297	-0.68383	0.12416
C	-3.52978	-0.12992	1.26866
C	-4.80455	0.44900	1.10688
H	-5.31608	0.88011	1.98277
C	-5.43483	0.49103	-0.14492
H	-6.43161	0.94809	-0.24921
C	-4.78471	-0.04847	-1.26379
H	-5.28068	-0.00834	-2.24725
C	-3.50940	-0.63898	-1.15695
C	-2.84798	-0.11317	2.63900
H	-1.97895	-0.80151	2.58011
C	-3.75922	-0.61796	3.77555
H	-4.62481	0.05683	3.94633
H	-3.19576	-0.67382	4.73077
H	-4.16152	-1.62910	3.55821
C	-2.29845	1.29542	2.95906
H	-1.62008	1.67407	2.16313
H	-1.73510	1.29918	3.91631
H	-3.12180	2.03596	3.04611
C	-2.80424	-1.17468	-2.40559
H	-1.94127	-1.78059	-2.05757
C	-3.69746	-2.09593	-3.25996
H	-4.10754	-2.93493	-2.66078
H	-3.11786	-2.52997	-4.10186
H	-4.55688	-1.54911	-3.70269
C	-2.24121	-0.01407	-3.25717
H	-3.05837	0.62530	-3.65350
H	-1.65579	-0.39357	-4.12139
H	-1.58097	0.66292	-2.66985
C	2.87617	-0.68103	0.12945
C	3.52759	-0.11948	1.27319
C	4.80417	0.45620	1.11406
H	5.31181	0.89287	1.98943
C	5.44099	0.48814	-0.13468
H	6.43906	0.94278	-0.23704
C	4.79551	-0.05760	-1.25322
H	5.29640	-0.02447	-2.23441
C	3.51872	-0.64526	-1.14887
C	2.83708	-0.09017	2.63892
H	1.97117	-0.78265	2.58214
C	2.28003	1.32013	2.93870
H	3.10054	2.06413	3.02334
H	1.70925	1.33301	3.89145
H	1.60657	1.68746	2.13300
C	3.74223	-0.57796	3.78755
H	4.15012	-1.58983	3.58461
H	3.17216	-0.62526	4.73930
H	4.60377	0.10234	3.95690
C	2.81735	-1.18609	-2.39744
H	1.95727	-1.79621	-2.04968
C	3.71540	-2.10268	-3.25162
H	4.57097	-1.55127	-3.69610
H	3.13756	-2.54121	-4.09237
H	4.13122	-2.93847	-2.65190
C	2.24824	-0.02869	-3.24947
H	1.58287	0.64347	-2.66232
H	1.66600	-0.41129	-4.11448
H	3.06220	0.61574	-3.64425
C	-0.00403	3.97210	-0.72635
H	1.91989	3.95615	-1.77919
C	-0.64150	4.68437	0.49127
H	-0.06589	4.45239	1.41226
H	-0.66604	5.78866	0.36803
H	-1.87068	3.92826	-1.87705
C	1.44977	4.46963	-0.91414
H	1.50018	5.56563	-1.09192
H	2.05307	4.23613	-0.01183
H	-1.68296	4.32863	0.63849
C	-0.82779	4.28976	-1.99807
H	-0.38537	3.77418	-2.87674
H	-0.86093	5.37882	-2.21743
O	-0.00095	2.58597	-0.50709

2

Enthalpy 0K = -4296.532739
Enthalpy 298K = -4296.440850
Free Energy 298K = -4296.662726
Lowest Frequency = 6.4098 cm^{-1}

Second Frequency = 15.7854 cm^{-1}
SCF (BP86-D3^{BJ}) Energy = -4298.480370
SCF ($C_6H_5CH_3$) Energy = -4298.054706
SCF (BS2) Energy = -4301.344692

Ca	-1.72015	0.05285	-0.36679
O	-0.28642	0.14442	1.38797
N	-3.88861	-1.05191	-0.69486
N	-2.93457	2.00426	-1.05282
C	-3.76180	1.82142	-2.10412
C	-4.26777	-4.65260	0.36994
H	-4.10645	-5.66868	-0.02609
C	-1.47444	4.02539	-1.16246
C	-4.44174	0.61482	-2.41857
H	-5.08410	0.70051	-3.30807
C	-4.94592	-3.20450	2.18175
H	-5.32066	-3.08290	3.20939
C	-4.70590	-4.49554	1.68934
H	-4.87570	-5.37589	2.32934
C	-5.11510	-0.67671	1.90868
H	-4.39608	0.04916	1.46491
C	-3.60729	-3.82143	-1.92079
H	-3.49920	-2.84185	-2.43124
C	-3.46718	3.99787	0.30234
C	-5.89371	-1.36836	-2.15518
H	-5.71699	-1.81783	-3.15540
H	-6.75788	-0.68251	-2.26209
H	-6.16820	-2.18096	-1.45766
C	-0.50809	3.36370	-2.14480
H	-0.95695	2.39192	-2.44459
C	-1.20290	5.33797	-0.72207
H	-0.31748	5.86368	-1.11540
C	-3.15402	5.31175	0.70437
H	-3.80404	5.82048	1.43385
C	-4.71025	3.30785	0.87107
H	-4.55814	2.21633	0.72715
C	-0.54071	0.42364	2.76726
C	-5.05665	-0.53532	3.44002
H	-5.20500	0.52524	3.73024
H	-4.08461	-0.86781	3.85580
H	-5.85783	-1.12032	3.94000
C	-2.03507	5.98772	0.19748
H	-1.81119	7.01615	0.52294
C	-4.67261	-4.63948	-2.68689
H	-4.39181	-4.74015	-3.75688
H	-5.67638	-4.17341	-2.63891
H	-4.76345	-5.66617	-2.27289
C	-4.03550	2.98828	-3.04891
H	-4.35738	3.89308	-2.49520
H	-4.80870	2.73736	-3.79886
H	-3.10713	3.27324	-3.58749
C	-6.51993	-0.27707	1.40047
H	-6.79463	0.73790	1.75793
H	-7.28566	-0.98942	1.77547
H	-6.57485	-0.27097	0.29489
C	0.83764	3.06913	-1.45479
H	1.53356	2.52933	-2.12722
H	0.68445	2.45262	-0.54295
H	1.34341	4.00195	-1.13304
C	-5.98872	3.69682	0.09424
H	-6.16621	4.79277	0.14725
H	-6.87931	3.18860	0.52179
H	-5.92619	3.41408	-0.97483
C	0.77933	0.80044	3.47173
H	0.62845	1.00987	4.55167
H	1.52201	-0.02500	3.39848
H	1.22031	1.70694	3.01228
C	-1.13268	-0.82479	3.45518
H	-2.04790	-1.16822	2.92928
H	-1.40070	-0.62021	4.51341
H	-0.40137	-1.65776	3.44378
C	-2.24609	-4.54068	-2.00920
H	-2.29072	-5.54919	-1.54585
H	-1.44620	-3.97601	-1.49337
H	-1.94046	-4.67542	-3.06812
C	-4.90873	3.56797	2.37772
H	-3.99609	3.33161	2.96161
H	-5.73948	2.94438	2.77040
H	-5.17846	4.62507	2.58727
C	-4.23629	-2.22552	0.04696
C	-2.62353	3.34393	-0.65629
C	-4.73130	-2.06277	1.38506
C	-4.65616	-0.59994	-1.69542
C	-4.03295	-3.54533	-0.47392
C	-0.28890	4.19144	-3.42846
H	0.22159	5.15375	-3.21313
H	-1.24705	4.42937	-3.93507
H	0.34971	3.63671	-4.14818
C	-1.54045	1.59565	2.86880
H	-1.15186	2.48997	2.34043
H	-1.74914	1.87695	3.92261
H	-2.51270	1.32091	2.40436
Ca	1.68443	-0.19632	0.32272
O	0.23935	-0.47215	-1.40295
N	3.69182	1.16799	0.80793
N	3.09958	-2.02777	0.92957
C	3.87482	-1.81505	2.01358
C	3.83007	4.87621	0.09056
H	3.55106	5.83366	0.56025
C	1.92328	-4.22915	0.83720
C	4.36721	-0.55369	2.44287
H	4.98150	-0.62317	3.35322
C	4.77339	3.66429	-1.77302
H	5.24124	3.66903	-2.76922
C	4.40583	4.88241	-1.18462
H	4.57585	5.83208	-1.71638
C	5.06596	1.13794	-1.74825
H	4.38071	0.32810	-1.40913
C	2.97444	3.77670	2.19274
H	2.94197	2.75239	2.61765
C	3.91758	-3.81462	-0.56434
C	5.55470	1.60936	2.41926
H	5.24974	1.97260	3.42380
H	6.48394	1.02225	2.56038
H	5.78205	2.49102	1.79259
C	0.87804	-3.80551	1.86991
H	1.18208	-2.80789	2.25484
C	1.82749	-5.51652	0.26817
H	1.01235	-6.18557	0.58955
C	3.78121	-5.11341	-1.09415

H	4.50558	-5.46611	-1.84613
C	5.07811	-2.93319	-1.03450
H	4.85669	-1.90132	-0.68865
C	0.37593	-0.94885	-2.74452
C	5.06469	1.15374	-3.28778
H	5.26961	0.13705	-3.68152
H	4.09210	1.49001	-3.70142
H	5.85304	1.82153	-3.69592
C	2.74666	-5.96684	-0.68722
H	2.65774	-6.97864	-1.11387
C	3.81250	4.65439	3.14933
H	3.39239	4.62002	4.17694
H	4.86964	4.32597	3.20069
H	3.81007	5.71835	2.83046
C	4.31017	-3.00620	2.86148
H	4.82010	-3.77280	2.24336
H	4.98992	-2.70250	3.67917
H	3.42813	-3.51137	3.30697
C	6.47445	0.77952	-1.22103
H	6.83647	-0.16923	-1.67050
H	7.20162	1.57847	-1.48048
H	6.48360	0.65601	-0.12043
C	-0.50928	-3.65600	1.21507
H	-1.27296	-3.32280	1.94591
H	-0.47989	-2.91823	0.38566
H	-0.85572	-4.62037	0.79049
C	6.41487	-3.36046	-0.38783
H	6.68066	-4.40182	-0.67113
H	7.24371	-2.69856	-0.71812
H	6.37097	-3.31375	0.71868
C	-1.01755	-0.99748	-3.41019
H	-0.96950	-1.36022	-4.45828
H	-1.48516	0.01144	-3.43257
H	-1.69503	-1.68382	-2.85811
C	1.28756	-0.00334	-3.55513
H	2.28738	0.07597	-3.07879
H	1.43909	-0.37013	-4.59230
H	0.85025	1.01378	-3.61158
C	1.52427	4.30289	2.13662
H	1.48471	5.33493	1.72880
H	0.87653	3.67394	1.49467
H	1.07514	4.32981	3.15184
C	5.21304	-2.89973	-2.57066
H	4.26700	-2.58946	-3.05983
H	6.00726	-2.18605	-2.87575
H	5.49512	-3.89043	-2.98634
C	3.96284	2.43170	0.18783
C	2.98005	-3.36097	0.42160
C	4.56893	2.43698	-1.11219
C	4.45390	0.73292	1.82155
C	3.59921	3.67636	0.79771
C	0.80371	-4.76768	3.07483
H	0.43665	-5.77171	2.77369
H	1.79390	-4.90804	3.55562
H	0.10225	-4.37902	3.84318
C	0.99730	-2.36074	-2.72992
H	0.36790	-3.06199	-2.14703
H	1.11435	-2.77406	-3.75410
H	2.00065	-2.34592	-2.25516

TS(A–P)

SCF (BP86) Energy = -4244.899277
Enthalpy 0K = -4243.918255
Enthalpy 298K = -4243.855827
Free Energy 298K = -4244.016148
Lowest Frequency = -1348.6365 cm^{-1}
Second Frequency = 14.5893 cm^{-1}
SCF (BP86-D3^{BJ}) Energy = -4245.187097
SCF ($C_6H_5CH_3$) Energy = -4244.939573
SCF (BS2) Energy = -4247.178281

Zn	-1.68520	2.44597	-0.26011
H	-0.86915	1.17564	-0.93299
Ca	0.24815	-0.17125	0.37067
N	-0.54431	-2.33063	-0.20664
N	2.34268	-1.11899	-0.20099
C	0.20367	-3.43896	-0.31576
C	1.62747	-3.46831	-0.27744
H	2.04997	-4.48062	-0.35585
C	2.60080	-2.43632	-0.31974
C	-0.45533	-4.80159	-0.50460
H	-1.47274	-4.71806	-0.93169
H	-0.54805	-5.31571	0.47650
H	0.15750	-5.45498	-1.15561
C	4.03204	-2.88881	-0.57467
H	4.39312	-2.49803	-1.54914
H	4.11664	-3.99127	-0.58988
H	4.72677	-2.48780	0.19040
C	-1.95777	-2.35761	-0.43238
C	-2.87870	-2.72796	0.59848
C	-4.26283	-2.64548	0.33732
H	-4.97597	-2.93140	1.12782
C	-4.75133	-2.21254	-0.90084
H	-5.83621	-2.15497	-1.08210
C	-3.84571	-1.85143	-1.90857
H	-4.23217	-1.51445	-2.88267
C	-2.45327	-1.91192	-1.70310
C	-2.42198	-3.22120	1.97378
H	-1.31121	-3.24048	1.96400
C	-2.91595	-4.65396	2.27261
H	-4.02254	-4.68950	2.36176
H	-2.49584	-5.02227	3.23274
H	-2.62534	-5.36716	1.47537
C	-2.86368	-2.25881	3.09679
H	-2.48665	-1.23159	2.92194
H	-2.48862	-2.60327	4.08437
H	-3.97058	-2.20047	3.16590
C	-1.49551	-1.56697	-2.84649
H	-0.54026	-1.23942	-2.37836
C	-1.17168	-2.81886	-3.69371
H	-0.73181	-3.63045	-3.08088
H	-0.44800	-2.57363	-4.50003
H	-2.09188	-3.21771	-4.17214
C	-1.99175	-0.41921	-3.74334
H	-2.89392	-0.70198	-4.32606
H	-1.20906	-0.14309	-4.48003
H	-2.23263	0.48463	-3.14794
C	3.37854	-0.16314	-0.45398
C	4.22062	0.31168	0.60286

C	5.14534	1.34065	0.33000
H	5.79154	1.71160	1.14260
C	5.26630	1.89910	-0.94925
H	5.99442	2.70357	-1.13904
C	4.45644	1.41689	-1.98585
H	4.55952	1.84684	-2.99575
C	3.51411	0.39093	-1.76886
C	4.16033	-0.26919	2.01854
H	3.42394	-1.10094	1.99711
C	3.66325	0.77144	3.04431
H	4.34597	1.64583	3.09613
H	3.60823	0.33083	4.06262
H	2.65577	1.15327	2.78078
C	5.51597	-0.85749	2.46743
H	5.89938	-1.61041	1.74835
H	5.42070	-1.35152	3.45760
H	6.29134	-0.06829	2.56457
C	2.67844	-0.11105	-2.95012
H	2.01458	-0.91417	-2.56658
C	3.56396	-0.73283	-4.05248
H	4.23398	0.02387	-4.51369
H	2.93782	-1.16128	-4.86350
H	4.20530	-1.54608	-3.65456
C	1.77865	0.99805	-3.53476
H	1.07165	1.39674	-2.77717
H	1.17621	0.60918	-4.38253
H	2.37753	1.85193	-3.91693
C	0.14635	3.74701	0.40732
C	1.56642	3.16997	0.44941
H	1.74490	2.43205	-0.36604
H	1.79104	2.67203	1.41830
H	2.34222	3.96094	0.31705
C	-0.03374	4.83500	1.47767
H	0.15543	4.45388	2.50403
H	-1.05918	5.26045	1.46455
H	0.67685	5.68229	1.31521
C	-0.06954	4.38990	-0.98638
H	0.70666	5.17426	-1.17690
H	-1.05254	4.90017	-1.07913
H	0.02416	3.65944	-1.82174
C	-3.57138	3.14833	-0.47878
H	-3.15283	5.25852	0.05332
C	-4.58866	2.06491	-0.07443
H	-4.54731	1.82934	1.01069
H	-5.63483	2.40384	-0.28762
H	-3.59059	2.55374	-2.61591
C	-3.83931	4.42856	0.32944
H	-4.88001	4.80257	0.15204
H	-3.74144	4.26777	1.42516
H	-4.43783	1.11221	-0.62677
C	-3.74622	3.45356	-1.98122
H	-3.04945	4.24075	-2.34415
H	-4.78304	3.82035	-2.19384
H	-2.80180	1.09484	2.34696
O	-0.12519	0.31143	2.55334
C	0.12004	-0.12606	3.90730
C	0.76209	-1.50387	3.90207
H	-0.84236	-0.14336	4.46486
H	0.77983	0.62545	4.40065
H	1.72900	-1.49964	3.35896
H	0.95775	-1.83502	4.94254
H	0.10006	-2.25463	3.42557
C	-0.67943	1.67657	2.38584
C	-2.11160	1.76798	2.89928
H	-0.43029	2.57020	1.12668
H	-0.02500	2.33252	3.00866
H	-2.23091	1.54866	3.98727
H	-2.48430	2.80341	2.74847

P

SCF (BP86) Energy = -4244.974465
Enthalpy 0K = -4243.989097
Enthalpy 298K = -4243.924904
Free Energy 298K = -4244.106208
Lowest Frequency = 1.6047 cm^{-1}
Second Frequency = 2.7582 cm^{-1}
SCF (BP86-D3^{BJ}) Energy = -4245.229590
SCF ($C_6H_5CH_3$) Energy = -4245.020698
SCF (BS2) Energy = -4247.259722

Zn	-1.66019	0.42484	1.35091
H	-0.06467	-0.06989	1.70269
Ca	0.91499	0.04271	-0.24463
N	3.00423	1.09953	-0.47351
N	2.14550	-1.89070	-0.74083
C	4.09189	0.57170	-1.05384
C	4.19470	-0.77514	-1.50897
H	5.15002	-1.00205	-2.00492
C	3.36865	-1.91281	-1.30485
C	5.34411	1.41627	-1.26243
H	5.35875	2.30758	-0.60670
H	5.39387	1.77228	-2.31404
H	6.25953	0.81954	-1.08077
C	3.96303	-3.25031	-1.72347
H	4.15986	-3.88469	-0.83397
H	4.91318	-3.12265	-2.27488
H	3.25398	-3.82278	-2.35548
C	2.98724	2.41495	0.08363
C	2.78978	3.57668	-0.73032
C	2.69231	4.83754	-0.10504
H	2.54723	5.73522	-0.72829
C	2.77239	4.97600	1.28587
H	2.69295	5.97098	1.75174
C	2.94926	3.83499	2.08066
H	3.00892	3.94501	3.17436
C	3.05625	2.55055	1.51111
C	2.66583	3.50415	-2.25438
H	2.77826	2.43786	-2.54535
C	3.77178	4.30852	-2.97204
H	3.67695	5.39634	-2.76836
H	3.70605	4.17183	-4.07240
H	4.78542	3.99815	-2.64848
C	1.27160	3.97387	-2.72435
H	0.46201	3.39393	-2.23698
H	1.16552	3.86375	-3.82477
H	1.10403	5.04468	-2.48217
C	3.30925	1.33605	2.40819
H	2.92907	0.44211	1.86364
C	4.82502	1.11371	2.61373

H	5.35611	0.98355	1.64952
H	5.01222	0.20859	3.22965
H	5.28128	1.98198	3.13585
C	2.57576	1.40258	3.75971
H	2.97299	2.20875	4.41228
H	2.70458	0.44902	4.31151
H	1.48744	1.56948	3.62674
C	1.50782	−3.11282	−0.35161
C	0.54115	−3.73970	−1.20105
C	−0.12899	−4.89383	−0.74459
H	−0.87156	−5.37887	−1.39942
C	0.13122	−5.43981	0.51834
H	−0.40255	−6.34218	0.85615
C	1.07602	−4.82237	1.34879
H	1.27765	−5.24833	2.34532
C	1.77325	−3.66655	0.94389
C	0.21487	−3.20158	−2.59538
H	0.86330	−2.31315	−2.75691
C	−1.25611	−2.74326	−2.69442
H	−1.95511	−3.59051	−2.53117
H	−1.47566	−2.32259	−3.69895
H	−1.49603	−1.96783	−1.93732
C	0.54274	−4.21988	−3.70865
H	1.60084	−4.55032	−3.66441
H	0.36682	−3.77773	−4.71236
H	−0.09205	−5.12784	−3.62939
C	2.77955	−3.03444	1.90910
H	3.22841	−2.15977	1.39373
C	3.92945	−4.00107	2.26644
H	3.56023	−4.88806	2.82425
H	4.68075	−3.49505	2.90903
H	4.45411	−4.37234	1.36179
C	2.08213	−2.51588	3.18535
H	1.28813	−1.77790	2.94556
H	2.81543	−2.02698	3.86144
H	1.60882	−3.34446	3.75429
C	−10.51227	0.46674	−0.48372
C	−10.91645	−0.75972	−1.32027
H	−11.03359	−1.66499	−0.68764
H	−10.15979	−0.98980	−2.10013
H	−11.88619	−0.58639	−1.83732
C	−10.30013	1.70396	−1.37344
H	−9.53212	1.51882	−2.15416
H	−9.97091	2.58253	−0.77921
H	−11.24250	1.98652	−1.89293
C	−11.53885	0.74755	0.62732
H	−12.53591	0.98776	0.19613
H	−11.23229	1.60856	1.25820
H	−11.66751	−0.13009	1.29558
C	−3.10461	0.31978	2.76412
H	−4.81956	0.51998	1.37559
C	−2.60983	1.00612	4.05018
H	−2.42050	2.09303	3.90398
H	−3.36595	0.92112	4.87388
H	−2.44264	−1.69765	3.39669
C	−4.41570	0.98035	2.30445
H	−5.21597	0.88596	3.08409
H	−4.29458	2.06910	2.10856
H	−1.66714	0.55471	4.42972
C	−3.36099	−1.17167	3.05456
H	−3.74276	−1.71828	2.16320
H	−4.12869	−1.30217	3.86144
H	−1.31407	3.19346	−0.20556
O	−0.74962	0.80945	−1.61533
C	−1.09057	0.95877	−3.00680
C	0.10087	0.59729	−3.87959
H	−1.40962	2.00744	−3.19826
H	−1.96362	0.30022	−3.22517
H	0.42222	−0.45168	−3.71632
H	−0.16759	0.70504	−4.95040
H	0.96506	1.26239	−3.67970
C	−1.83836	1.09417	−0.64126
C	−2.15806	2.59076	−0.60645
H	−9.53852	0.23395	0.00800
H	−2.72771	0.53845	−1.02558
H	−2.43687	3.02783	−1.59450
H	−3.02512	2.75948	0.06682

TS(A–C)

SCF (BP86) Energy = −4244.891108
Enthalpy 0K = −4243.909546
Enthalpy 298K = −4243.847387
Free Energy 298K = −4244.009293
Lowest Frequency = −1388.3160 cm^{-1}
Second Frequency = 9.6747 cm^{-1}
SCF (BP86-D3^{BJ}) Energy = −4245.174953
SCF ($C_6H_5CH_3$) Energy = −4244.932437
SCF (BS2) Energy = −4247.171597

Zn	3.16391	−0.96346	−0.11661
H	1.61457	−1.38146	−0.51780
Ca	0.00010	−0.09770	0.20897
N	−0.77527	1.89587	−0.84717
N	−1.72648	−1.20008	−0.97650
C	−1.27643	1.76293	−2.08577
C	−1.56388	0.52200	−2.73184
H	−1.78173	0.64200	−3.80399
C	−1.86754	−0.78165	−2.25088
C	−1.65365	2.98464	−2.91982
H	−1.29578	3.93489	−2.48459
H	−2.75875	3.04544	−3.01338
H	−1.25648	2.88508	−3.95060
C	−2.39557	−1.73878	−3.31584
H	−1.54061	−2.25716	−3.80100
H	−2.94642	−1.20079	−4.11110
H	−3.05160	−2.51974	−2.88665
C	−0.70283	3.19349	−0.23802
C	−1.81076	3.66908	0.53381
C	−1.72918	4.93609	1.14697
H	−2.58672	5.30609	1.73243
C	−0.58679	5.73543	1.02171
H	−0.54261	6.72597	1.50170
C	0.50302	5.25810	0.28241
H	1.40309	5.88554	0.18958
C	0.47932	3.99720	−0.35062
C	−3.09489	2.85392	0.69749
H	−2.90650	1.85416	0.25183
C	−4.27608	3.48870	−0.06868
H	−4.52725	4.49096	0.34007

H	-5.18601	2.85656	0.01063
H	-4.04380	3.61786	-1.14476
C	-3.46129	2.64531	2.18146
H	-2.61697	2.20286	2.74880
H	-4.33534	1.96822	2.28065
H	-3.72837	3.60159	2.67927
C	1.70610	3.55835	-1.15818
H	1.64126	2.45395	-1.28027
C	1.72540	4.16892	-2.57861
H	0.83575	3.88583	-3.17166
H	2.62417	3.82839	-3.13465
H	1.75860	5.27843	-2.52732
C	3.03488	3.88559	-0.44474
H	3.23943	4.97693	-0.43956
H	3.88376	3.40287	-0.97153
H	3.03964	3.53480	0.60662
C	-2.34670	-2.41435	-0.53403
C	-3.64422	-2.33473	0.07773
C	-4.22945	-3.50623	0.59726
H	-5.22406	-3.45084	1.06579
C	-3.57618	-4.74505	0.52672
H	-4.05073	-5.64885	0.94095
C	-2.31973	-4.82087	-0.08354
H	-1.81237	-5.79700	-0.15287
C	-1.68606	-3.68082	-0.62481
C	-4.41537	-1.01228	0.12271
H	-3.65345	-0.20247	0.17177
C	-5.34611	-0.87475	1.34281
H	-6.21141	-1.56875	1.28539
H	-5.76697	0.15135	1.39042
H	-4.82063	-1.07476	2.29886
C	-5.22808	-0.78746	-1.17365
H	-4.58268	-0.77071	-2.07239
H	-5.77182	0.18050	-1.13365
H	-5.97952	-1.59475	-1.30912
C	-0.33407	-3.86632	-1.31858
H	0.01804	-2.86487	-1.64599
C	-0.44947	-4.76628	-2.57009
H	-0.72496	-5.80652	-2.29380
H	0.52094	-4.81220	-3.10828
H	-1.21738	-4.39906	-3.27957
C	0.73264	-4.43502	-0.36252
H	0.86005	-3.79695	0.53455
H	1.71787	-4.49825	-0.87026
H	0.46863	-5.45672	-0.01553
C	3.47045	-1.78064	2.06732
C	2.30718	-2.36482	2.88208
H	1.56271	-2.87479	2.23303
H	1.76211	-1.59229	3.46320
H	2.66903	-3.12577	3.61653
C	4.47889	-1.08553	2.99631
H	4.01588	-0.26603	3.58825
H	5.32501	-0.64265	2.42876
H	4.91584	-1.80442	3.73216
C	4.19398	-2.95631	1.36485
H	4.53442	-3.71088	2.12064
H	5.10304	-2.63387	0.81457
H	3.53923	-3.50840	0.65432
C	4.68704	-0.41658	-1.33764
H	4.50042	-2.26794	-2.54429
C	5.80663	0.33983	-0.60398
H	6.28337	-0.27777	0.18817
H	6.62270	0.63933	-1.31113
H	3.67899	1.42533	-2.04644
C	5.27139	-1.68060	-1.99802
H	6.05889	-1.40893	-2.74708
H	5.75240	-2.36456	-1.26584
H	5.44149	1.27201	-0.12050
C	4.09993	0.47772	-2.44595
H	3.28924	-0.03147	-3.01223
H	4.88540	0.76423	-3.19217
H	2.76954	-0.51484	1.60606
O	0.04047	0.22268	2.54286
C	-0.79040	0.01475	3.69484
C	-1.93371	-0.91880	3.34014
H	-1.16779	0.99995	4.05854
H	-0.16126	-0.41795	4.50809
H	-1.56069	-1.90965	3.00961
H	-2.58236	-1.07913	4.22503
H	-2.56708	-0.49446	2.53466
C	1.23743	1.03310	2.80931
C	2.19788	0.90772	1.64249
H	1.70317	0.64417	3.74376
H	0.87793	2.07101	3.01883
H	1.86517	1.55453	0.79249
H	3.16532	1.36282	1.93983

TS(D-Q)

SCF (BP86) Energy = -4086.583035
Enthalpy 0K = -4085.729252
Enthalpy 298K = -4085.674375
Free Energy 298K = -4085.820661
Lowest Frequency = -169.4136 cm-1
Second Frequency = 8.9003 cm-1
SCF (BP86-D3BJ) Energy = -4086.829979
SCF (C6H5CH3) Energy = -4086.625265
SCF (Et2O) Energy = N/A
SCF (BS2) Energy = -4088.691578

Zn	-3.54897	-1.54872	-0.81064
H	-1.57031	-1.36186	0.67945
Ca	0.42904	-0.19304	-0.12354
N	0.21807	1.95869	0.92495
N	2.66370	0.11152	0.58264
C	1.14874	2.64513	1.61062
C	2.48100	2.20601	1.83694
H	3.08314	2.89212	2.44999
C	3.19728	1.08494	1.34020
C	0.82294	4.02700	2.16822
H	-0.25521	4.14811	2.38792
H	1.09361	4.81526	1.43278
H	1.40582	4.22826	3.08816
C	4.67816	1.02733	1.68734
H	4.92307	0.10210	2.24825
H	4.99240	1.89897	2.29104
H	5.29355	0.99475	0.76392
C	-1.03282	2.56258	0.60399
C	-1.13161	3.48049	-0.49318
C	-2.40107	3.98016	-0.84894

H	-2.48547	4.68289	-1.69396
C	-3.55736	3.60917	-0.14906
H	-4.53834	4.01614	-0.44119
C	-3.45492	2.71631	0.92730
H	-4.36526	2.43262	1.47825
C	-2.21328	2.17633	1.32004
C	0.09917	3.92022	-1.29437
H	0.99819	3.57345	-0.74505
C	0.20119	5.45464	-1.42589
H	-0.61823	5.87286	-2.04833
H	1.15845	5.74137	-1.91015
H	0.15491	5.95522	-0.43685
C	0.13990	3.25494	-2.68716
H	0.15470	2.14720	-2.61450
H	1.04662	3.56452	-3.24899
H	-0.74686	3.53494	-3.29513
C	-2.13150	1.23428	2.52427
H	-1.20653	0.62830	2.39444
C	-1.96698	2.01514	3.84797
H	-1.05963	2.65051	3.84157
H	-1.88519	1.31795	4.70884
H	-2.84226	2.67580	4.02677
C	-3.32080	0.26207	2.62088
H	-4.26925	0.78292	2.87078
H	-3.14373	-0.49074	3.41674
H	-3.47634	-0.28791	1.66793
C	3.47629	-0.97000	0.12037
C	3.99829	-0.94027	-1.21130
C	4.69048	-2.06819	-1.69865
H	5.09317	-2.05176	-2.72498
C	4.88042	-3.20679	-0.90436
H	5.42242	-4.07897	-1.30318
C	4.37524	-3.22575	0.40384
H	4.52586	-4.12294	1.02634
C	3.67432	-2.12738	0.94005
C	3.82463	0.28533	-2.11336
H	3.31011	1.06263	-1.50887
C	2.93440	-0.02192	-3.33773
H	3.38717	-0.80688	-3.98039
H	2.79745	0.88485	-3.96473
H	1.92550	-0.38306	-3.04429
C	5.18088	0.87487	-2.55622
H	5.82287	1.12042	-1.68524
H	5.03271	1.80617	-3.14323
H	5.74640	0.16527	-3.19711
C	3.11490	-2.21206	2.36356
H	2.73492	-1.20332	2.62876
C	4.19096	-2.58870	3.40344
H	4.57783	-3.61744	3.24261
H	3.76960	-2.55390	4.43037
H	5.05962	-1.89845	3.36933
C	1.91777	-3.18566	2.43720
H	1.12986	-2.91856	1.70145
H	1.46712	-3.18423	3.45270
H	2.23408	-4.22648	2.21057
C	-4.48429	-3.09032	0.06480
H	-6.46598	-2.09902	-0.08207
C	-3.63454	-3.76073	1.15604
H	-3.38613	-3.06428	1.98511
H	-4.18278	-4.62527	1.60833
H	-3.87188	-4.52305	-1.51682
C	-5.80650	-2.58202	0.67255
H	-6.39121	-3.42896	1.11296
H	-5.64183	-1.84797	1.49085
H	-2.67491	-4.15792	0.76088
C	-4.79373	-4.11165	-1.04985
H	-5.41774	-3.68100	-1.86407
H	-5.36162	-4.98506	-0.64028
O	-0.67788	-0.73618	-2.13552
C	-0.64708	-2.21957	-2.04754
C	0.20692	-2.61081	-0.87321
H	-0.34000	-2.57340	-3.06378
H	-1.70461	-2.56450	-1.89846
H	-0.95214	-1.84849	0.33379
H	-0.08711	-3.60287	-0.47562
H	1.28846	-2.67352	-1.14246
C	-1.81287	-0.20708	-2.87271
C	-3.04765	-0.02557	-1.99592
H	-2.01051	-0.89177	-3.72953
H	-1.45466	0.75323	-3.30432
H	-2.93672	0.88627	-1.36665
H	-3.92950	0.16412	-2.65055

Q

SCF (BP86) Energy = -4086.591664
Enthalpy 0K = -4085.739918
Enthalpy 298K = -4085.682698
Free Energy 298K = -4085.837451
Lowest Frequency = 7.3022 cm^{-1}
Second Frequency = 10.5558 cm^{-1}
SCF (BP86-D3^{BJ}) Energy = -4086.827852
SCF ($C_6H_5CH_3$) Energy = -4086.635285
SCF (BS2) Energy = -4088.704349

Zn	-4.22715	-1.66872	-0.87844
H	1.28443	0.23101	3.09231
Ca	0.61671	-0.30485	0.41572
N	0.56269	2.07392	0.57087
N	2.96077	0.08708	0.32436
C	1.59518	2.86571	0.90928
C	2.94980	2.44455	1.00675
H	3.64283	3.24265	1.30822
C	3.59400	1.22112	0.66394
C	1.33457	4.34101	1.18091
H	0.52458	4.47008	1.92789
H	0.98636	4.85398	0.26005
H	2.24172	4.85895	1.54411
C	5.11630	1.25200	0.66148
H	5.53662	0.50001	1.35962
H	5.50754	2.24844	0.93849
H	5.50650	0.98652	-0.34335
C	-0.72587	2.62829	0.30195
C	-1.04022	3.08409	-1.02073
C	-2.36076	3.48340	-1.30953
H	-2.60750	3.82903	-2.32711
C	-3.36579	3.45690	-0.33179
H	-4.39198	3.77170	-0.57972
C	-3.04631	3.04202	0.96798
H	-3.83028	3.04320	1.74326

C	-1.74073	2.63382	1.31187
C	0.02006	3.15293	-2.12412
H	0.99042	2.86650	-1.66844
C	0.17586	4.58053	-2.69153
H	-0.74396	4.91749	-3.21539
H	1.00868	4.62183	-3.42517
H	0.39155	5.31954	-1.89244
C	-0.26827	2.14812	-3.25905
H	-0.32619	1.10809	-2.87709
H	0.52895	2.18274	-4.03170
H	-1.23359	2.36806	-3.76287
C	-1.43882	2.23458	2.75841
H	-0.35076	2.02208	2.81494
C	-1.73539	3.38255	3.74791
H	-1.19620	4.31262	3.47267
H	-1.42629	3.10174	4.77701
H	-2.81895	3.62465	3.78206
C	-2.18746	0.95052	3.17195
H	-3.28847	1.09212	3.12769
H	-1.93003	0.65858	4.21222
H	-1.93376	0.09382	2.51280
C	3.69797	-1.07183	-0.07261
C	3.84972	-1.36063	-1.46754
C	4.48872	-2.55601	-1.85526
H	4.61115	-2.77745	-2.92838
C	4.97423	-3.46569	-0.90678
H	5.46968	-4.39533	-1.22899
C	4.82295	-3.17998	0.45691
H	5.20411	-3.89683	1.20264
C	4.19512	-1.99849	0.90133
C	3.35090	-0.39565	-2.54643
H	2.83209	0.43609	-2.02314
C	2.33257	-1.06580	-3.49264
H	2.79397	-1.89326	-4.07252
H	1.93038	-0.33326	-4.22399
H	1.47628	-1.49602	-2.93194
C	4.51779	0.22369	-3.34604
H	5.23799	0.74344	-2.68141
H	4.14275	0.96404	-4.08440
H	5.08170	-0.55249	-3.90639
C	4.05515	-1.75546	2.40732
H	3.62793	-0.73976	2.53969
C	5.41469	-1.78799	3.13888
H	5.87451	-2.79849	3.10549
H	5.28784	-1.51966	4.20903
H	6.14392	-1.07834	2.69555
C	3.07201	-2.75744	3.05026
H	2.07367	-2.71573	2.56839
H	2.93984	-2.54204	4.13207
H	3.44472	-3.80022	2.95928
C	-5.64489	-2.36004	0.33949
H	-7.29314	-1.81309	-1.04195
C	-5.25838	-2.09159	1.80557
H	-5.13835	-1.00751	2.02056
H	-6.05014	-2.47209	2.49903
H	-4.87459	-4.43957	0.34594
C	-6.96872	-1.64346	0.00825
H	-7.79530	-2.01652	0.66412
H	-6.90739	-0.54438	0.16548
H	-4.30993	-2.59484	2.09334
C	-5.80412	-3.87600	0.11274
H	-6.09166	-4.12220	-0.93314
H	-6.60671	-4.29200	0.77241
O	-0.87433	-1.45702	-0.94555
C	-1.23512	-2.55434	0.05952
C	-0.56094	-2.30863	1.36316
H	-0.94583	-3.49625	-0.46270
H	-2.35760	-2.52711	0.10841
H	0.86455	-0.41667	3.06789
H	-1.27793	-2.12933	2.19056
H	0.10471	-3.14811	1.65103
C	-1.55073	-1.55123	-2.21353
C	-2.95720	-0.94741	-2.21072
H	-1.57455	-2.62572	-2.51681
H	-0.88504	-1.03396	-2.94153
H	-2.89100	0.15607	-2.07592
H	-3.41135	-1.10801	-3.21632

TS(Q–R)

SCF (BP86) Energy = -4086.591344
Enthalpy 0K = -4085.741005
Enthalpy 298K = -4085.683741
Free Energy 298K = -4085.840138
Lowest Frequency = -82.8465 cm^{-1}
Second Frequency = 5.5793 cm^{-1}
SCF (BP86-D3^{BJ}) Energy = -4086.825415
SCF ($C_6H_5CH_3$) Energy = -4086.636203
SCF (BS2) Energy = -4088.704333

Zn	-4.23989	-1.75276	-0.86989
H	1.24111	0.24330	3.98972
Ca	0.61365	-0.25868	0.36304
N	0.60193	2.11606	0.48285
N	2.96320	0.09099	0.29354
C	1.65234	2.91837	0.72983
C	3.00535	2.49023	0.80828
H	3.71623	3.29744	1.03423
C	3.62157	1.23472	0.54497
C	1.41464	4.41133	0.90967
H	0.62643	4.59958	1.66697
H	1.04800	4.86736	-0.03382
H	2.33695	4.94023	1.21409
C	5.14370	1.23477	0.52229
H	5.55666	0.50196	1.24472
H	5.55820	2.23329	0.75437
H	5.51671	0.92210	-0.47551
C	-0.69313	2.66662	0.23715
C	-1.05139	3.07037	-1.09158
C	-2.37535	3.47984	-1.34762
H	-2.65581	3.78636	-2.36889
C	-3.34210	3.51187	-0.33220
H	-4.37136	3.83617	-0.55380
C	-2.98148	3.13943	0.96942
H	-3.73643	3.18205	1.77177
C	-1.67132	2.71979	1.28148
C	-0.03563	3.06060	-2.23829
H	0.95679	2.82748	-1.80015
C	0.07684	4.43287	-2.93597
H	-0.86417	4.71012	-3.45704

H	0.88407	4.41876	-3.69859
H	0.30701	5.24307	-2.21354
C	-0.35321	1.95353	-3.26569
H	-0.37984	0.95109	-2.79008
H	0.41053	1.92668	-4.07176
H	-1.34266	2.11537	-3.74409
C	-1.32597	2.36779	2.73067
H	-0.26170	2.05085	2.74342
C	-1.45443	3.59397	3.66175
H	-0.83696	4.44625	3.31034
H	-1.12738	3.34013	4.69235
H	-2.50549	3.94864	3.72309
C	-2.17204	1.19283	3.26267
H	-3.25385	1.44449	3.28129
H	-1.87408	0.93143	4.29991
H	-2.04691	0.28372	2.63928
C	3.67752	-1.10168	-0.04174
C	3.85265	-1.45081	-1.42043
C	4.46249	-2.67965	-1.74507
H	4.60061	-2.94874	-2.80539
C	4.90041	-3.56328	-0.74979
H	5.37413	-4.51957	-1.02295
C	4.73120	-3.21619	0.59728
H	5.07809	-3.91082	1.37993
C	4.12916	-1.99980	0.97954
C	3.40387	-0.51912	-2.54971
H	2.94483	0.37329	-2.07327
C	2.33189	-1.17923	-3.44267
H	2.73149	-2.07255	-3.96818
H	1.96921	-0.47148	-4.21787
H	1.45548	-1.51549	-2.84935
C	4.59442	-0.02481	-3.39953
H	5.36078	0.47915	-2.77554
H	4.25542	0.69926	-4.17066
H	5.09451	-0.86489	-3.92720
C	3.98064	-1.68421	2.47101
H	3.53152	-0.67210	2.55112
C	5.34285	-1.65102	3.19853
H	5.82584	-2.65135	3.20669
H	5.21292	-1.33814	4.25617
H	6.05379	-0.94483	2.72159
C	3.02281	-2.67405	3.16774
H	2.01984	-2.66932	2.69452
H	2.89556	-2.41080	4.23909
H	3.41356	-3.71318	3.12262
C	-5.67819	-2.29078	0.39955
H	-7.29802	-2.00334	-1.08967
C	-5.33890	-1.79129	1.81619
H	-5.24692	-0.68448	1.86503
H	-6.14205	-2.08054	2.53991
H	-4.86437	-4.32459	0.74440
C	-7.00692	-1.66679	-0.07040
H	-7.84343	-1.95585	0.61459
H	-6.97338	-0.55541	-0.08234
H	-4.38915	-2.22163	2.20145
C	-5.79845	-3.82701	0.40402
H	-6.05039	-4.23725	-0.59864
H	-6.61077	-4.15746	1.09919
O	-0.88553	-1.52073	-0.88518
C	-1.27441	-2.49298	0.23501
C	-0.60780	-2.11634	1.51051
H	-0.99638	-3.49164	-0.17563
H	-2.39637	-2.44037	0.26755
H	0.83139	-0.33214	3.68644
H	-1.32713	-1.82221	2.30187
H	0.03160	-2.93350	1.90200
C	-1.52997	-1.76681	-2.14983
C	-2.94379	-1.18867	-2.25240
H	-1.53376	-2.86899	-2.32957
H	-0.85231	-1.32526	-2.91566
H	-2.89478	-0.07621	-2.25523
H	-3.37071	-1.47926	-3.24055

R

SCF (BP86) Energy = -4085.421349
Enthalpy 0K = -4084.582410
Enthalpy 298K = -4084.527515
Free Energy 298K = -4084.677742
Lowest Frequency = 8.3579 cm^{-1}
Second Frequency = 13.0925 cm^{-1}
SCF (BP86-D3^{BJ}) Energy = -4085.651795
SCF ($C_6H_5CH_3$) Energy = -4085.466663
SCF (BS2) Energy = -4087.529419

Zn	-4.28450	-1.72243	-0.64901
Ca	0.63466	-0.19192	0.08175
N	0.72928	2.18553	0.14981
N	2.98268	0.12755	0.02746
C	1.79530	3.00313	0.17603
C	3.14590	2.56724	0.12197
H	3.88781	3.37794	0.14068
C	3.70181	1.26121	0.04250
C	1.57311	4.50588	0.26975
H	0.96288	4.75441	1.16300
H	1.00268	4.88232	-0.60466
H	2.52843	5.05991	0.32889
C	5.21957	1.17822	-0.03361
H	5.63450	0.63997	0.84360
H	5.68396	2.18085	-0.08028
H	5.53507	0.59748	-0.92538
C	-0.59927	2.70072	0.10548
C	-1.19005	3.04667	-1.15520
C	-2.55689	3.38689	-1.19554
H	-3.01913	3.64707	-2.16193
C	-3.34148	3.40801	-0.03252
H	-4.40871	3.67571	-0.08737
C	-2.75115	3.10271	1.20123
H	-3.36279	3.14366	2.11764
C	-1.38774	2.75426	1.29981
C	-0.37001	3.04470	-2.44980
H	0.69778	2.94851	-2.16450
C	-0.51872	4.35697	-3.24750
H	-1.54929	4.49046	-3.63967
H	0.16725	4.36186	-4.12077
H	-0.28274	5.24460	-2.62465
C	-0.70894	1.82823	-3.33672
H	-0.55166	0.87185	-2.79542
H	-0.07439	1.80887	-4.24820
H	-1.77087	1.84719	-3.66215

C	-0.77557	2.48589	2.67762
H	0.29314	2.23012	2.51317
C	-0.81475	3.74778	3.56806
H	-0.32329	4.61327	3.07742
H	-0.29581	3.56365	4.53258
H	-1.85854	4.04746	3.80260
C	-1.43813	1.29077	3.39366
H	-2.51676	1.47566	3.58470
H	-0.95397	1.10431	4.37567
H	-1.35546	0.35820	2.79730
C	3.62263	-1.14851	0.01944
C	3.74034	-1.87420	-1.20897
C	4.24796	-3.18960	-1.18096
H	4.34071	-3.75206	-2.12486
C	4.64228	-3.79329	0.02030
H	5.03456	-4.82267	0.02299
C	4.53687	-3.07362	1.21941
H	4.85100	-3.55000	2.16253
C	4.03634	-1.75673	1.24980
C	3.34190	-1.24866	-2.54874
H	3.00299	-0.21223	-2.33406
C	2.16610	-2.00159	-3.20788
H	2.44278	-3.04820	-3.45696
H	1.85582	-1.50527	-4.15210
H	1.27959	-2.05064	-2.53997
C	4.53931	-1.14582	-3.51771
H	5.38239	-0.58378	-3.06588
H	4.24571	-0.62476	-4.45374
H	4.92211	-2.14946	-3.80100
C	3.92976	-1.02236	2.59022
H	3.65698	0.03056	2.36975
C	5.26837	-1.00236	3.35839
H	5.57579	-2.02045	3.67922
H	5.18124	-0.38230	4.27556
H	6.09319	-0.58533	2.74351
C	2.80304	-1.60984	3.46809
H	1.82334	-1.58266	2.94668
H	2.70607	-1.04081	4.41734
H	3.00988	-2.66961	3.73062
C	-5.58554	-2.21394	0.77754
H	-7.34168	-2.05278	-0.56964
C	-5.13085	-1.61913	2.12332
H	-5.05883	-0.51013	2.09445
H	-5.85888	-1.87457	2.93401
H	-4.69244	-4.20519	1.16942
C	-6.96705	-1.64681	0.39564
H	-7.72988	-1.90810	1.17176
H	-6.96289	-0.53792	0.31265
H	-4.14114	-2.00895	2.44583
C	-5.66594	-3.74871	0.88699
H	-5.99748	-4.22635	-0.06118
H	-6.40311	-4.05053	1.67301
O	-0.95412	-1.49514	-1.01909
C	-1.22424	-2.45539	0.15292
C	-0.43232	-2.06215	1.34608
H	-0.99256	-3.45679	-0.27992
H	-2.33520	-2.39875	0.30514
H	-1.06394	-1.81177	2.22199
H	0.29958	-2.84178	1.64076
C	-1.72244	-1.76686	-2.20641
C	-3.14118	-1.19236	-2.17252
H	-1.74248	-2.87260	-2.36246
H	-1.12859	-1.34127	-3.04719
H	-3.09461	-0.08014	-2.20004
H	-3.66904	-1.50136	-3.10473

TS(R-S)

SCF (BP86) Energy = -4085.420393
Enthalpy 0K = -4084.582354
Enthalpy 298K = -4084.527831
Free Energy 298K = -4084.676575
Lowest Frequency = -276.4528 cm^{-1}
Second Frequency = 9.7110 cm^{-1}
SCF (BP86-D3^{BJ}) Energy = -4085.651874
SCF ($C_6H_5CH_3$) Energy = -4085.465069
SCF (BS2) Energy = -4087.529153

Zn	-4.18430	-1.74883	-0.68092
Ca	0.59820	-0.18934	0.03474
N	0.64405	2.18357	0.18465
N	2.94045	0.18031	0.02520
C	1.69169	3.02401	0.23222
C	3.05150	2.61990	0.16649
H	3.77536	3.44622	0.20203
C	3.63565	1.32825	0.06129
C	1.43716	4.51871	0.36564
H	0.81090	4.72933	1.25747
H	0.87033	4.90910	-0.50490
H	2.38034	5.08960	0.45262
C	5.15474	1.27955	-0.01698
H	5.58360	0.74291	0.85436
H	5.59649	2.29275	-0.05464
H	5.48133	0.71469	-0.91486
C	-0.69418	2.67401	0.14647
C	-1.27834	3.06458	-1.10425
C	-2.64830	3.39159	-1.14169
H	-3.10548	3.68626	-2.10043
C	-3.44327	3.35480	0.01393
H	-4.51253	3.61455	-0.03920
C	-2.86168	2.99890	1.23808
H	-3.48240	2.99158	2.14925
C	-1.49544	2.66037	1.33322
C	-0.45042	3.11611	-2.39279
H	0.61741	3.03190	-2.10396
C	-0.61881	4.44671	-3.15517
H	-1.64853	4.57083	-3.55263
H	0.07347	4.48818	-4.02242
H	-0.40496	5.32198	-2.50715
C	-0.76147	1.91729	-3.31374
H	-0.59587	0.95022	-2.79489
H	-0.11776	1.93237	-4.21888
H	-1.82026	1.92988	-3.64965
C	-0.89347	2.33037	2.70214
H	0.17181	2.06340	2.53144
C	-0.91368	3.55832	3.63938
H	-0.39845	4.42983	3.18556
H	-0.40851	3.32775	4.60128
H	-1.95347	3.87167	3.87382
C	-1.57996	1.12236	3.37231

H	-2.65173	1.32452	3.58270
H	-1.09233	0.87941	4.34004
H	-1.52959	0.21517	2.73479
C	3.60459	-1.08314	0.01241
C	3.73174	-1.80458	-1.21772
C	4.25531	-3.11387	-1.19187
H	4.35462	-3.67309	-2.13697
C	4.65790	-3.71504	0.00782
H	5.06174	-4.73994	0.00847
C	4.54847	-2.99732	1.20784
H	4.87279	-3.47029	2.14931
C	4.03248	-1.68647	1.24090
C	3.33709	-1.17820	-2.55841
H	2.96379	-0.15426	-2.34015
C	2.19821	-1.95725	-3.25055
H	2.50758	-2.99496	-3.49843
H	1.90245	-1.46311	-4.20051
H	1.29556	-2.02607	-2.60704
C	4.55188	-1.02926	-3.50003
H	5.36871	-0.44791	-3.02488
H	4.26282	-0.50719	-4.43689
H	4.96951	-2.01863	-3.78447
C	3.93187	-0.95219	2.58239
H	3.63846	0.09582	2.36564
C	5.28180	-0.91038	3.33015
H	5.60869	-1.92312	3.64855
H	5.20019	-0.28873	4.24678
H	6.09097	-0.48387	2.70132
C	2.83055	-1.55496	3.48152
H	1.84043	-1.53704	2.98120
H	2.74589	-0.98919	4.43385
H	3.05371	-2.61294	3.73749
C	-5.41876	-2.30603	0.78157
H	-7.24073	-1.87064	-0.40839
C	-4.83438	-1.90224	2.14772
H	-4.67913	-0.80490	2.23496
H	-5.52597	-2.19744	2.97672
H	-4.65757	-4.38623	0.88616
C	-6.77485	-1.60553	0.56615
H	-7.50411	-1.90187	1.36180
H	-6.69149	-0.49738	0.60604
H	-3.85901	-2.39427	2.35356
C	-5.60951	-3.83413	0.72784
H	-6.03749	-4.17586	-0.24025
H	-6.31489	-4.17308	1.52786
O	-0.86499	-1.42055	-1.15322
C	-1.04858	-2.57036	0.09972
C	-0.31414	-2.20415	1.28220
H	-0.71064	-3.45962	-0.47082
H	-2.16035	-2.55645	0.17960
H	-0.91157	-1.93367	2.17198
H	0.55942	-2.83849	1.52134
C	-1.65960	-1.72128	-2.29778
C	-3.09233	-1.17679	-2.22334
H	-1.67120	-2.83071	-2.44616
H	-1.12102	-1.30452	-3.18233
H	-3.06731	-0.06335	-2.23063
H	-3.63938	-1.47825	-3.14760

S

SCF (BP86) Energy = -4085.448218
Enthalpy 0K = -4084.610691
Enthalpy 298K = -4084.554847
Free Energy 298K = -4084.702829
Lowest Frequency = 13.5053 cm^{-1}
Second Frequency = 25.4519 cm^{-1}
SCF (BP86-D3^{BJ}) Energy = -4085.698017
SCF ($C_6H_5CH_3$) Energy = -4085.485317
SCF (BS2) Energy = -4087.556176

Zn	-2.48714	-2.99566	0.36953
Ca	0.13447	-0.33994	-0.00360
N	-0.54919	1.74192	-0.90210
N	2.26847	0.39336	-0.78464
C	0.12488	2.46267	-1.81479
C	1.45527	2.19886	-2.23909
H	1.81138	2.86940	-3.03481
C	2.46065	1.33476	-1.72570
C	-0.53750	3.68613	-2.43253
H	-0.76743	4.43844	-1.64908
H	-1.50865	3.42704	-2.89983
H	0.10820	4.16152	-3.19398
C	3.85547	1.58039	-2.29527
H	4.32913	2.45520	-1.80048
H	3.79539	1.81804	-3.37600
H	4.52957	0.71480	-2.15273
C	-1.84245	2.16762	-0.46490
C	-3.02728	1.73704	-1.14615
C	-4.28360	2.10158	-0.61848
H	-5.19854	1.77251	-1.13789
C	-4.39823	2.87267	0.54602
H	-5.39119	3.14417	0.93793
C	-3.23633	3.29378	1.20645
H	-3.32589	3.90206	2.12167
C	-1.95494	2.95726	0.72539
C	-2.97896	0.89663	-2.42619
H	-1.90949	0.73818	-2.67890
C	-3.64424	1.61739	-3.62008
H	-4.73420	1.75548	-3.45603
H	-3.52166	1.02467	-4.55109
H	-3.20726	2.62157	-3.79875
C	-3.61806	-0.49322	-2.22256
H	-3.09733	-1.06231	-1.42492
H	-3.55552	-1.09385	-3.15428
H	-4.69136	-0.41252	-1.94838
C	-0.71932	3.44509	1.48723
H	0.17032	3.06408	0.94243
C	-0.62343	4.98590	1.50888
H	-0.62799	5.41004	0.48384
H	0.31215	5.31634	2.00818
H	-1.47403	5.43994	2.06077
C	-0.66979	2.87653	2.92170
H	-1.53078	3.22588	3.53033
H	0.25779	3.19362	3.44310
H	-0.70226	1.76621	2.92570
C	3.38193	-0.24239	-0.16196
C	3.66186	-1.62233	-0.44220
C	4.70120	-2.25969	0.26675
H	4.92876	-3.31565	0.05772

C	5.45984	-1.57934	1.23058
H	6.26608	-2.10036	1.77084
C	5.18418	-0.23223	1.49714
H	5.78204	0.30079	2.25456
C	4.15826	0.45862	0.81883
C	2.88318	-2.38410	-1.52184
H	1.81953	-2.05488	-1.46493
C	2.89044	-3.91232	-1.33489
H	3.89641	-4.34883	-1.51219
H	2.19961	-4.38678	-2.06147
H	2.56608	-4.21697	-0.31810
C	3.37970	-2.03202	-2.94270
H	3.27729	-0.95237	-3.16235
H	2.79525	-2.58640	-3.70701
H	4.44922	-2.30753	-3.06417
C	3.89083	1.92418	1.18104
H	3.15477	2.32433	0.45461
C	5.16186	2.79503	1.08490
H	5.91324	2.51557	1.85381
H	4.91416	3.86588	1.24324
H	5.65139	2.69998	0.09406
C	3.25481	2.05307	2.58247
H	2.29020	1.50874	2.64606
H	3.05528	3.11746	2.82947
H	3.92273	1.64459	3.37085
C	-3.24332	-2.00611	1.95266
H	-5.30440	-2.72181	1.58020
C	-2.54423	-0.64641	2.12258
H	-2.66898	0.01132	1.23266
H	-2.95576	-0.06809	2.98848
H	-1.99893	-3.03537	3.48116
C	-4.74613	-1.76884	1.70813
H	-5.21653	-1.23227	2.57172
H	-4.93437	-1.14800	0.80573
H	-1.45430	-0.76266	2.33739
C	-3.06718	-2.84185	3.23407
H	-3.56836	-3.83148	3.16148
H	-3.50862	-2.32083	4.12233
O	-0.75553	-2.05950	-0.80520
C	0.92259	-2.48847	2.05973
C	1.40220	-1.39651	2.69052
H	1.53538	-3.04510	1.33178
H	-0.08778	-2.88418	2.25811
H	0.80583	-0.85859	3.44864
H	2.42583	-1.02990	2.50118
C	-0.98153	-3.10911	-1.75460
C	-1.88701	-4.17360	-1.12256
H	0.00405	-3.51817	-2.09221
H	-1.46643	-2.68461	-2.66840
H	-2.66531	-4.56841	-1.80881
H	-1.31333	-5.04214	-0.72977

TS(S–T)

SCF (BP86) Energy = -4085.445006
Enthalpy 0K = -4084.608402
Enthalpy 298K = -4084.552675
Free Energy 298K = -4084.704446
Lowest Frequency = -18.7443 cm^{-1}
Second Frequency = 9.4162 cm^{-1}
SCF (BP86-D3^{BJ}) Energy = -4085.689571
SCF ($C_6H_5CH_3$) Energy = -4085.483819
SCF (BS2) Energy = -4087.553640

Zn	1.65510	3.32529	0.11952
Ca	-0.12668	0.19052	-0.13386
N	1.02346	-1.78503	-0.72469
N	-2.00209	-1.13674	-0.67396
C	0.53566	-2.82015	-1.42962
C	-0.84282	-3.03818	-1.69732
H	-1.05064	-3.95942	-2.26076
C	-2.00611	-2.33847	-1.27775
C	1.48622	-3.85482	-2.02009
H	2.40661	-3.97217	-1.41641
H	1.80439	-3.52892	-3.03449
H	0.99608	-4.84114	-2.13131
C	-3.32516	-3.07014	-1.49610
H	-3.61950	-3.60483	-0.56658
H	-3.23645	-3.82767	-2.29820
H	-4.15581	-2.37925	-1.73826
C	2.39274	-1.74838	-0.31644
C	3.38853	-1.10766	-1.11944
C	4.70695	-1.01980	-0.62416
H	5.47952	-0.53071	-1.23986
C	5.05804	-1.54500	0.62606
H	6.09464	-1.46763	0.99038
C	4.07718	-2.16697	1.41211
H	4.35602	-2.57669	2.39596
C	2.74332	-2.27694	0.97172
C	3.07395	-0.53113	-2.50201
H	1.99277	-0.70250	-2.69200
C	3.86428	-1.25152	-3.61696
H	4.95661	-1.07486	-3.51779
H	3.55790	-0.87919	-4.61743
H	3.70292	-2.34858	-3.59521
C	3.32207	0.99064	-2.55815
H	2.70518	1.52780	-1.80872
H	3.05768	1.39528	-3.55783
H	4.38846	1.23753	-2.36969
C	1.70522	-2.98458	1.84902
H	0.70504	-2.66552	1.48140
C	1.77725	-4.51964	1.68249
H	1.62411	-4.82890	0.62993
H	1.00039	-5.02062	2.29863
H	2.76792	-4.90517	2.00628
C	1.80497	-2.60474	3.33977
H	2.74131	-2.98063	3.80400
H	0.96342	-3.05248	3.90923
H	1.77501	-1.50623	3.49088
C	-3.22413	-0.56312	-0.21130
C	-3.87565	0.44806	-0.99083
C	-5.06016	1.02658	-0.48861
H	-5.57728	1.79610	-1.08192
C	-5.59138	0.65095	0.75381
H	-6.51821	1.11753	1.12353
C	-4.92795	-0.31189	1.52717
H	-5.33927	-0.59156	2.51035
C	-3.74739	-0.93133	1.06977
C	-3.31329	0.89104	-2.34582
H	-2.21118	0.74306	-2.29780

C	-3.56602	2.37979	-2.65045
H	-4.63805	2.58731	-2.85557
H	-3.00071	2.68498	-3.55491
H	-3.24673	3.03350	-1.81343
C	-3.83366	0.01630	-3.50869
H	-3.55324	-1.04791	-3.38798
H	-3.41377	0.36507	-4.47597
H	-4.94124	0.07228	-3.57960
C	-3.01814	-1.93961	1.96250
H	-2.31458	-2.50643	1.31818
C	-3.96021	-2.96212	2.62812
H	-4.63846	-2.48683	3.36820
H	-3.37483	-3.73262	3.17239
H	-4.59372	-3.48066	1.87919
C	-2.16870	-1.20923	3.02618
H	-1.43737	-0.50955	2.56486
H	-1.59355	-1.92727	3.64784
H	-2.80477	-0.59994	3.70243
C	2.23738	2.61971	1.91441
H	4.31676	3.33803	1.72653
C	1.52978	1.28650	2.21774
H	1.86101	0.47464	1.52384
H	1.77518	0.90260	3.24083
H	0.75400	3.80635	3.04991
C	3.76051	2.39737	1.92989
H	4.10795	2.02715	2.92854
H	4.08810	1.64674	1.17802
H	0.41551	1.39214	2.20755
C	1.85277	3.64467	2.99759
H	2.32772	4.63430	2.82373
H	2.17690	3.30369	4.01461
O	0.23649	2.04133	-1.05246
C	-2.31697	2.94808	1.53812
C	-2.38195	3.43875	2.78808
H	-3.07842	2.24681	1.15460
H	-1.51322	3.23800	0.83787
H	-1.63636	4.15535	3.17304
H	-3.19264	3.15268	3.47964
C	0.24767	3.07511	-2.05339
C	1.13609	4.23485	-1.57760
H	-0.80138	3.40742	-2.24204
H	0.61837	2.64562	-3.01630
H	1.98148	4.45785	-2.26222
H	0.57623	5.18100	-1.41716

T

SCF (BP86) Energy = -4085.445436
Enthalpy 0K = -4084.608498
Enthalpy 298K = -4084.552038
Free Energy 298K = -4084.706476
Lowest Frequency = 8.5814 cm^{-1}
Second Frequency = 11.4101 cm^{-1}
SCF (BP86-D3^{BJ}) Energy = -4085.683035
SCF ($C_6H_5CH_3$) Energy = -4085.486284
SCF (BS2) Energy = -4087.554927

Zn	-0.01464	3.62883	-0.19029
Ca	-0.02141	0.03126	0.25159
N	-1.82718	-1.46234	0.48732
N	1.20432	-1.95635	0.54968
C	-1.79709	-2.70963	0.98743
C	-0.60940	-3.45276	1.22563
H	-0.78075	-4.46506	1.62044
C	0.75379	-3.15501	0.95441
C	-3.09866	-3.41192	1.35438
H	-3.93197	-3.11762	0.68688
H	-3.40203	-3.13199	2.38659
H	-2.98591	-4.51277	1.33050
C	1.71932	-4.32421	1.10872
H	1.73557	-4.93239	0.17847
H	1.39726	-4.99688	1.92780
H	2.75748	-3.99085	1.29952
C	-3.05632	-0.81551	0.15796
C	-3.81419	-0.11601	1.15025
C	-4.96638	0.59209	0.74982
H	-5.55431	1.13368	1.50873
C	-5.37949	0.62353	-0.58807
H	-6.28350	1.18244	-0.87753
C	-4.63107	-0.05871	-1.55862
H	-4.96065	-0.02962	-2.60851
C	-3.46945	-0.78040	-1.21700
C	-3.39913	-0.09584	2.62361
H	-2.55553	-0.80830	2.74001
C	-4.53358	-0.56050	3.56136
H	-5.38690	0.15056	3.55718
H	-4.16977	-0.62846	4.60834
H	-4.92952	-1.55550	3.27068
C	-2.88030	1.29856	3.03691
H	-2.01933	1.61365	2.41134
H	-2.54942	1.29720	4.09735
H	-3.67374	2.06930	2.93366
C	-2.69123	-1.54877	-2.29081
H	-1.62488	-1.56237	-1.96597
C	-3.14023	-3.02597	-2.37174
H	-2.99574	-3.55693	-1.41118
H	-2.56111	-3.57067	-3.14757
H	-4.21618	-3.09266	-2.64055
C	-2.75577	-0.90280	-3.68754
H	-3.76577	-0.99691	-4.14006
H	-2.04834	-1.40870	-4.37710
H	-2.49830	0.17558	-3.66421
C	2.56361	-1.75266	0.17241
C	3.43729	-1.03924	1.06000
C	4.75220	-0.75983	0.63509
H	5.43380	-0.22120	1.31069
C	5.21327	-1.15411	-0.62981
H	6.24554	-0.92564	-0.93921
C	4.35059	-1.83671	-1.49738
H	4.71454	-2.13766	-2.49344
C	3.02664	-2.14764	-1.12496
C	2.97130	-0.62605	2.46013
H	1.88202	-0.40336	2.38810
C	3.66279	0.64106	2.99448
H	4.73043	0.45792	3.24136
H	3.16976	0.97789	3.92946
H	3.62009	1.47691	2.26705
C	3.12011	-1.78264	3.47460
H	2.53857	-2.67613	3.17585
H	2.76438	-1.46939	4.47905

H	4.18507	-2.08480	3.56973
C	2.11860	-2.86978	-2.12543
H	1.17775	-3.12950	-1.59795
C	2.74012	-4.18372	-2.64451
H	3.64814	-3.99679	-3.25637
H	2.01833	-4.73057	-3.28728
H	3.03397	-4.85535	-1.81197
C	1.73768	-1.94751	-3.30430
H	1.22739	-1.02454	-2.95744
H	1.05262	-2.46518	-4.00880
H	2.63465	-1.62987	-3.87746
C	-0.19049	3.15777	-2.14120
H	-1.68272	4.72353	-2.58361
C	-0.07628	1.63335	-2.31906
H	-0.94535	1.10156	-1.85542
H	-0.09923	1.32563	-3.39529
H	1.95060	3.53428	-2.58834
C	-1.55624	3.62350	-2.67841
H	-1.67279	3.37993	-3.76591
H	-2.40942	3.14684	-2.14746
H	0.89035	1.23417	-1.92190
C	0.93913	3.83922	-2.93506
H	0.88820	4.94647	-2.85460
H	0.87722	3.59148	-4.02600
O	0.24635	1.87835	1.22233
C	3.83464	3.17781	-0.15390
C	4.50961	4.33693	-0.08560
H	4.30751	2.24592	-0.50760
H	2.77473	3.10670	0.14794
H	4.03115	5.26459	0.27105
H	5.56970	4.41015	-0.38259
C	0.49340	2.86408	2.23928
C	0.13190	4.25308	1.69382
H	1.57024	2.82821	2.53245
H	-0.08860	2.59579	3.15589
H	-0.85014	4.61737	2.06714
H	0.88750	5.03537	1.91596

U

SCF (BP86) Energy = -4006.921398
Enthalpy 0K = -4006.134657
Enthalpy 298K = -4006.083272
Free Energy 298K = -4006.223194
Lowest Frequency = 8.3086 cm^{-1}
Second Frequency = 14.2346 cm^{-1}
SCF (BP86-D3^{BJ}) Energy = -4007.147227
SCF ($C_6H_5CH_3$) Energy = -4006.961482
SCF (BS2) Energy = -4008.941613

Zn	-0.82982	-3.66509	-0.39469
Ca	0.06817	-0.28100	0.26780
N	1.83827	1.24469	0.50780
N	-1.18032	1.63061	0.83020
C	1.81849	2.42676	1.14732
C	0.63619	3.07783	1.59277
H	0.81100	4.03302	2.11015
C	-0.73608	2.77034	1.38139
C	3.12618	3.15021	1.44337
H	3.87139	2.99824	0.63773
H	3.58183	2.75354	2.37637
H	2.96533	4.23563	1.58751
C	-1.71756	3.86051	1.79630
H	-1.71916	4.68598	1.05263
H	-1.41612	4.30510	2.76603
H	-2.75447	3.48283	1.87915
C	3.04062	0.69001	-0.02319
C	3.94360	-0.05509	0.80055
C	5.06421	-0.66501	0.19968
H	5.76360	-1.23981	0.82843
C	5.30680	-0.56124	-1.17604
H	6.18957	-1.04563	-1.62270
C	4.41316	0.15852	-1.98222
H	4.60636	0.23416	-3.06350
C	3.27603	0.78740	-1.43629
C	3.70716	-0.23958	2.30136
H	2.86492	0.42396	2.58888
C	4.93114	0.16483	3.14960
H	5.79504	-0.51079	2.97349
H	4.68946	0.11033	4.23204
H	5.26737	1.19826	2.92504
C	3.27317	-1.68786	2.61566
H	2.35624	-1.96709	2.05609
H	3.06020	-1.81030	3.69901
H	4.06975	-2.41323	2.34436
C	2.33738	1.60130	-2.33270
H	1.32994	1.56876	-1.85665
C	2.74904	3.09058	-2.37377
H	2.74860	3.54617	-1.36428
H	2.05077	3.67619	-3.00893
H	3.77020	3.20177	-2.79724
C	2.20563	1.04444	-3.76255
H	3.13926	1.18056	-4.34857
H	1.40252	1.57939	-4.31083
H	1.96150	-0.03778	-3.76916
C	-2.53810	1.43855	0.44756
C	-3.35365	0.52272	1.19428
C	-4.65509	0.24032	0.73173
H	-5.28941	-0.45662	1.30048
C	-5.16353	0.83213	-0.43333
H	-6.18358	0.59610	-0.77554
C	-4.36340	1.72494	-1.15826
H	-4.76538	2.18643	-2.07523
C	-3.05349	2.04429	-0.74595
C	-2.85493	-0.08460	2.50934
H	-1.74692	-0.17015	2.43669
C	-3.40266	-1.49504	2.78920
H	-4.49418	-1.48547	2.99481
H	-2.91057	-1.92433	3.68601
H	-3.21736	-2.18451	1.94088
C	-3.14675	0.85627	3.70060
H	-2.68440	1.85333	3.56209
H	-2.75207	0.42771	4.64621
H	-4.24103	1.00446	3.82335
C	-2.21977	3.00576	-1.59912
H	-1.26552	3.19036	-1.06458
C	-2.91808	4.36908	-1.79255
H	-3.84891	4.27212	-2.39096
H	-2.25307	5.07692	-2.33113
H	-3.19464	4.82979	-0.82232

C	-1.86029	2.38338	-2.96605
H	-1.30388	1.43029	-2.85008
H	-1.22615	3.07482	-3.56061
H	-2.76983	2.16316	-3.56460
C	-0.94386	-3.01539	-2.29929
H	-0.13218	-4.88644	-3.14643
C	-0.61836	-1.51367	-2.38298
H	0.43043	-1.29628	-2.05717
H	-0.67576	-1.12231	-3.43048
H	-3.13716	-2.68839	-2.25657
C	0.06814	-3.79338	-3.16057
H	0.02638	-3.47180	-4.23308
H	1.11755	-3.64790	-2.82096
H	-1.35015	-0.89272	-1.80735
C	-2.36876	-3.24614	-2.83574
H	-2.65100	-4.32062	-2.80680
H	-2.46319	-2.91582	-3.90213
O	-0.08068	-2.19554	1.12514
C	-0.14490	-3.27519	2.07252
C	-0.82778	-4.48541	1.41912
H	-0.68091	-2.92339	2.98831
H	0.89008	-3.53853	2.40078
H	-0.26287	-5.43457	1.53060
H	-1.85794	-4.66033	1.79813

TS(U-L)

SCF (BP86) Energy = -4006.886950
Enthalpy 0K = -4006.103459
Enthalpy 298K = -4006.051317
Free Energy 298K = -4006.195833
Lowest Frequency = -338.8201 cm-1
Second Frequency = 9.9329 cm-1
SCF (BP86-D3BJ) Energy = -4007.104583
SCF (C6H5CH3) Energy = -4006.929050
SCF (Et2O) Energy = N/A
SCF (BS2) Energy = -4008.909514

Zn	-2.21643	2.77799	-0.39932
Ca	0.27459	0.04317	0.25703
N	2.49614	-0.72758	0.23868
N	-0.12846	-2.25254	-0.05524
C	2.95741	-1.98912	0.27024
C	2.14325	-3.14386	0.11617
H	2.69309	-4.09547	0.13517
C	0.73781	-3.27799	-0.05918
C	4.44265	-2.23056	0.49941
H	4.78392	-1.70937	1.41852
H	5.05171	-1.81708	-0.33110
H	4.67244	-3.30805	0.59603
C	0.20795	-4.69098	-0.25302
H	-0.53342	-4.95059	0.53153
H	1.01937	-5.44216	-0.23557
H	-0.33084	-4.77416	-1.22020
C	3.36632	0.39356	0.34246
C	4.09220	0.86810	-0.79854
C	4.85149	2.04817	-0.67523
H	5.41225	2.41962	-1.54824
C	4.90338	2.76885	0.52768
H	5.50492	3.68883	0.59799
C	4.17619	2.31311	1.63501
H	4.21088	2.88428	2.57745
C	3.40383	1.13545	1.56709
C	3.99668	0.16113	-2.15317
H	3.55000	-0.83884	-1.97693
C	5.36799	-0.05389	-2.82436
H	5.84593	0.90631	-3.11303
H	5.25610	-0.65313	-3.75232
H	6.07451	-0.58980	-2.15662
C	3.03582	0.92252	-3.09384
H	2.03119	1.04445	-2.63798
H	2.91065	0.38262	-4.05645
H	3.42058	1.93953	-3.32147
C	2.59937	0.68332	2.78952
H	2.16424	-0.30987	2.53732
C	3.47740	0.47699	4.04061
H	4.29730	-0.24304	3.84243
H	2.87433	0.08482	4.88668
H	3.94052	1.42889	4.37645
C	1.43569	1.65689	3.09171
H	1.81678	2.65241	3.40327
H	0.78799	1.27554	3.90934
H	0.79151	1.85883	2.20326
C	-1.53289	-2.46386	-0.18854
C	-2.15968	-2.34128	-1.46838
C	-3.56476	-2.43108	-1.54683
H	-4.05523	-2.33654	-2.52951
C	-4.35076	-2.63475	-0.40460
H	-5.44698	-2.70032	-0.48905
C	-3.73135	-2.75096	0.84881
H	-4.35358	-2.90796	1.74440
C	-2.33110	-2.67062	0.98355
C	-1.34229	-2.09161	-2.73792
H	-0.27115	-2.14055	-2.44835
C	-1.60702	-0.67916	-3.30653
H	-2.66465	-0.56925	-3.62957
H	-0.96651	-0.48731	-4.19392
H	-1.40409	0.11506	-2.55468
C	-1.57958	-3.17580	-3.81011
H	-1.37676	-4.19395	-3.41744
H	-0.91699	-3.01089	-4.68599
H	-2.62634	-3.16445	-4.18202
C	-1.68211	-2.75498	2.36860
H	-0.60851	-2.99198	2.21604
C	-2.27565	-3.86458	3.25849
H	-3.33005	-3.65765	3.53893
H	-1.69961	-3.95580	4.20329
H	-2.25171	-4.84932	2.74814
C	-1.74924	-1.39162	3.09250
H	-1.28942	-0.57235	2.49383
H	-1.22123	-1.42163	4.06911
H	-2.79930	-1.08160	3.27548
C	-3.96852	2.96436	0.57182
H	-4.01650	5.16474	0.34934
C	-4.04381	1.93834	1.71532
H	-3.23412	2.08271	2.46518
H	-5.01284	2.02275	2.27268
H	-5.09926	1.69534	-0.84800
C	-4.07391	4.38886	1.14366
H	-5.04864	4.53948	1.67558

H	-3.27022	4.61016	1.88051
H	-3.97833	0.89080	1.34687
C	-5.12195	2.72051	-0.41769
H	-5.10571	3.43880	-1.26651
H	-6.11599	2.83764	0.08630
O	-0.61165	1.72182	-0.61456
C	-0.20231	3.18519	-1.89221
C	-1.25765	4.11462	-1.91516
H	-0.08858	2.46998	-2.72218
H	0.73527	3.42697	-1.36744
H	-1.15435	5.07368	-1.37849
H	-1.97893	4.10220	-2.75074

TS(G-V)

SCF (BP86) Energy = -4008.052937
Enthalpy 0K = -4007.253706
Enthalpy 298K = -4007.201381
Free Energy 298K = -4007.342479
Lowest Frequency = -368.2149 cm^{-1}
Second Frequency = 15.2912 cm^{-1}
SCF (BP86-D3^{BJ}) Energy = -4008.285520
SCF ($C_6H_5CH_3$) Energy = -4008.093464
SCF (BS2) Energy = -4010.075511

Zn	-2.57410	-1.91751	-0.58262
H	-2.02465	-0.50942	-2.04841
Ca	0.42412	-0.14859	-0.38070
N	2.51824	0.44409	0.54375
N	-0.14020	1.86889	0.70337
C	2.87197	1.48538	1.31100
C	1.98533	2.52408	1.71014
H	2.44220	3.30538	2.33374
C	0.60637	2.71351	1.43418
C	4.30674	1.59015	1.80550
H	4.59631	0.67448	2.36184
H	4.45258	2.46639	2.46433
H	5.01487	1.66816	0.95470
C	-0.04528	3.95903	2.01525
H	-0.47987	4.58691	1.20920
H	0.67461	4.57174	2.58923
H	-0.89027	3.68951	2.68273
C	3.46420	-0.54290	0.13495
C	3.54434	-1.78545	0.84355
C	4.36754	-2.80903	0.33014
H	4.43251	-3.76798	0.87027
C	5.10751	-2.63216	-0.84662
H	5.74163	-3.44623	-1.23219
C	5.03776	-1.40778	-1.52674
H	5.62610	-1.26904	-2.44872
C	4.23103	-0.35031	-1.06102
C	2.78877	-2.01515	2.15681
H	2.21270	-1.08835	2.36701
C	3.76339	-2.22441	3.33738
H	4.35458	-3.15705	3.21516
H	3.20816	-2.30510	4.29601
H	4.48339	-1.38518	3.43093
C	1.78234	-3.18317	2.06575
H	1.00425	-3.01082	1.29177
H	1.26078	-3.32126	3.03709
H	2.29333	-4.14068	1.82818
C	4.18991	0.96614	-1.84464
H	3.57845	1.68152	-1.25600
C	5.59424	1.58533	-2.01343
H	6.10672	1.71901	-1.03809
H	5.52677	2.58075	-2.50132
H	6.24948	0.95091	-2.64753
C	3.50289	0.79859	-3.21790
H	4.05822	0.08455	-3.86295
H	3.45627	1.76967	-3.75538
H	2.46642	0.41382	-3.11895
C	-1.53137	2.07665	0.49117
C	-2.48416	1.48758	1.38690
C	-3.85470	1.55326	1.05952
H	-4.59457	1.10782	1.74508
C	-4.29822	2.19066	-0.10892
H	-5.37350	2.23314	-0.34376
C	-3.36316	2.78847	-0.96657
H	-3.71679	3.30029	-1.87644
C	-1.98200	2.75146	-0.68989
C	-2.04464	0.82064	2.69450
H	-0.94707	0.96273	2.77954
C	-2.31166	-0.69943	2.69739
H	-3.39667	-0.92251	2.61842
H	-1.94333	-1.16669	3.63523
H	-1.79690	-1.21469	1.85642
C	-2.69547	1.48451	3.92756
H	-2.50726	2.57768	3.95182
H	-2.29133	1.04893	4.86569
H	-3.79628	1.33632	3.94067
C	-0.99176	3.42063	-1.64730
H	-0.00047	3.41116	-1.14735
C	-1.35393	4.89449	-1.92659
H	-2.30878	4.98784	-2.48644
H	-0.56552	5.37944	-2.54001
H	-1.46024	5.47548	-0.98703
C	-0.84669	2.63277	-2.96788
H	-0.50630	1.58732	-2.80324
H	-0.10884	3.12101	-3.63920
H	-1.81352	2.57493	-3.51183
C	-4.48576	-2.45120	-0.76607
H	-4.69488	-1.54211	-2.77129
C	-5.20614	-2.39907	0.59106
H	-5.24831	-1.36847	1.00226
H	-6.26273	-2.74835	0.48068
H	-3.97733	-4.60657	-0.59825
C	-5.18513	-1.52488	-1.77462
H	-6.24661	-1.84345	-1.92292
H	-5.19942	-0.46926	-1.43081
H	-4.72813	-3.05094	1.35329
C	-4.47532	-3.90002	-1.29677
H	-3.97137	-3.98774	-2.28366
H	-5.52266	-4.26660	-1.43712
H	-1.18804	-0.65931	-2.24063
O	-0.71051	-2.14030	-0.24690
C	-0.21143	-2.67812	-1.55303
C	0.38796	-1.56666	-2.40660
H	-1.06836	-3.15663	-2.09342
H	0.47322	-3.51828	-1.28985
H	1.50186	-1.52751	-2.29032

H	0.20006	-1.70965	-3.48939

V

SCF (BP86) Energy = -4008.069117
Enthalpy 0K = -4007.274128
Enthalpy 298K = -4007.217486
Free Energy 298K = -4007.378077
Lowest Frequency = 2.5108 cm^{-1}
Second Frequency = 4.1701 cm^{-1}
SCF ($BP86-D3^{BJ}$) Energy = -4008.287750
SCF ($C_6H_5CH_3$) Energy = -4008.110739
SCF (Et2O) Energy = N/A
SCF (BS2) Energy = -4010.097145

Zn	-3.46465	-0.47381	-0.73204
H	-3.36347	5.50476	-5.69901
Ca	0.43505	0.04936	-0.64073
N	1.72088	-1.38556	0.76302
N	1.30897	1.67017	0.87401
C	2.44984	-1.07803	1.84800
C	2.59509	0.23087	2.38361
H	3.22432	0.28305	3.28329
C	2.10459	1.48964	1.94058
C	3.20045	-2.18665	2.57275
H	2.51082	-3.00142	2.87561
H	3.72271	-1.81102	3.47231
H	3.95005	-2.65578	1.90151
C	2.54505	2.70288	2.74791
H	3.14372	3.39485	2.11912
H	3.15189	2.41517	3.62656
H	1.66927	3.28849	3.09594
C	1.66961	-2.72142	0.26532
C	0.61097	-3.59885	0.66737
C	0.50508	-4.86637	0.05931
H	-0.30789	-5.54522	0.36650
C	1.41248	-5.28421	-0.92380
H	1.31066	-6.27769	-1.38873
C	2.45365	-4.42702	-1.30629
H	3.17024	-4.75882	-2.07561
C	2.60788	-3.15022	-0.72930
C	-0.39838	-3.19727	1.74672
H	-0.11018	-2.18531	2.10209
C	-0.35737	-4.14672	2.96370
H	-0.67193	-5.17625	2.68962
H	-1.04053	-3.79158	3.76436
H	0.66266	-4.21542	3.39451
C	-1.83300	-3.10230	1.18402
H	-1.88536	-2.38026	0.33959
H	-2.54513	-2.77361	1.97143
H	-2.18747	-4.08309	0.80130
C	3.76460	-2.25427	-1.18193
H	3.75454	-1.35390	-0.53242
C	5.13706	-2.93748	-1.00211
H	5.29308	-3.28054	0.04183
H	5.95905	-2.23494	-1.25544
H	5.24564	-3.82370	-1.66315
C	3.57551	-1.77529	-2.63777
H	3.57244	-2.63101	-3.34665
H	4.39852	-1.09282	-2.93919
H	2.61564	-1.23215	-2.77165
C	0.90322	2.97755	0.47250
C	-0.34747	3.51210	0.92219
C	-0.78796	4.74812	0.40664
H	-1.74963	5.16378	0.75096
C	-0.02889	5.46216	-0.53054
H	-0.39212	6.42524	-0.92305
C	1.19936	4.94058	-0.96018
H	1.79826	5.50549	-1.69341
C	1.68878	3.71072	-0.47564
C	-1.20834	2.78028	1.95575
H	-0.66471	1.85270	2.23434
C	-2.57520	2.36228	1.37209
H	-3.17609	3.24490	1.06557
H	-3.16937	1.79860	2.12338
H	-2.44723	1.72129	0.47224
C	-1.40183	3.61025	3.24340
H	-0.42997	3.90471	3.69010
H	-1.96374	3.03019	4.00610
H	-1.97270	4.54259	3.04621
C	3.03714	3.18637	-0.97816
H	3.27264	2.27359	-0.39167
C	4.18175	4.19454	-0.74145
H	4.04589	5.12038	-1.34016
H	5.15763	3.75301	-1.03492
H	4.25197	4.49553	0.32455
C	2.96593	2.77416	-2.46468
H	2.18117	2.00814	-2.64285
H	3.93622	2.35421	-2.80550
H	2.73042	3.64497	-3.11354
C	-5.37555	-0.75548	-0.30965
H	-6.06574	0.50338	-2.00129
C	-5.57478	-0.82020	1.21529
H	-5.26513	0.11800	1.72336
H	-6.65461	-0.97873	1.45641
H	-5.26884	-2.95584	-0.56097
C	-6.17788	0.42114	-0.89868
H	-7.26826	0.28483	-0.69403
H	-5.88574	1.39736	-0.45600
H	-5.01383	-1.65851	1.68093
C	-5.82364	-2.07937	-0.95884
H	-5.70063	-2.07498	-2.06334
H	-6.90757	-2.26106	-0.75512
H	-2.81836	5.12680	-5.31301
O	-1.71745	-0.21171	-1.23825
C	-1.46442	-0.11684	-2.79677
C	-0.02894	0.07385	-3.10160
H	-2.11438	0.73074	-3.11033
H	-1.89258	-1.06900	-3.18379
H	0.42702	-0.76864	-3.65911
H	0.19949	1.03606	-3.60186

W

SCF (BP86) Energy = -4006.896653
Enthalpy 0K = -4006.111752
Enthalpy 298K = -4006.059530
Free Energy 298K = -4006.203107
Lowest Frequency = 5.1295 cm^{-1}
Second Frequency = 9.8974 cm^{-1}

SCF (BP86-$D3^{BJ}$) Energy = -4007.115064
SCF ($C_6H_5CH_3$) Energy = -4006.938858
SCF (BS2) Energy = -4008.919412

Zn	-3.51622	-0.00085	-0.80532
Ca	0.41510	0.00010	-0.65448
N	1.48042	-1.54173	0.81762
N	1.47955	1.54257	0.81758
C	2.21403	-1.29545	1.91503
C	2.51384	0.00072	2.41660
H	3.11824	0.00090	3.33464
C	2.21331	1.29672	1.91500
C	2.79775	-2.46733	2.69186
H	2.00295	-3.18080	2.99233
H	3.33690	-2.13380	3.59804
H	3.50172	-3.04521	2.05696
C	2.79635	2.46894	2.69181
H	3.49988	3.04729	2.05686
H	3.33581	2.13573	3.59793
H	2.00113	3.18189	2.99239
C	1.27975	-2.87449	0.35019
C	0.11162	-3.60664	0.73941
C	-0.13002	-4.86813	0.15809
H	-1.02768	-5.43546	0.45554
C	0.74701	-5.41862	-0.78644
H	0.53851	-6.40479	-1.23086
C	1.89440	-4.70347	-1.15692
H	2.58636	-5.13944	-1.89621
C	2.18595	-3.43947	-0.60550
C	-0.87217	-3.05777	1.77671
H	-0.47434	-2.07826	2.11694
C	-0.97992	-3.97175	3.01658
H	-1.40808	-4.96343	2.75720
H	-1.63781	-3.51590	3.78683
H	0.01186	-4.15007	3.48069
C	-2.26851	-2.80717	1.16720
H	-2.21049	-2.10637	0.30556
H	-2.95788	-2.37561	1.92472
H	-2.72731	-3.74851	0.79648
C	3.45329	-2.70055	-1.04508
H	3.53427	-1.78808	-0.41780
C	4.72886	-3.53718	-0.80926
H	4.81550	-3.86767	0.24674
H	5.63543	-2.94474	-1.05539
H	4.74762	-4.44775	-1.44538
C	3.36018	-2.24229	-2.51682
H	3.27429	-3.11057	-3.20491
H	4.26556	-1.66959	-2.81054
H	2.47482	-1.59420	-2.69135
C	1.27808	2.87521	0.35019
C	0.10956	3.60668	0.73953
C	-0.13288	4.86803	0.15825
H	-1.03083	5.43484	0.45580
C	0.74374	5.41904	-0.78636
H	0.53462	6.40510	-1.23075
C	1.89149	4.70456	-1.15699
H	2.58311	5.14094	-1.89636
C	2.18383	3.44073	-0.60562
C	-0.87382	3.05720	1.77689
H	-0.47528	2.07800	2.11720
C	-2.26998	2.80552	1.16740
H	-2.72946	3.74649	0.79659
H	-2.95903	2.37352	1.92495
H	-2.21144	2.10468	0.30582
C	-0.98223	3.97120	3.01669
H	0.00942	4.15027	3.48078
H	-1.63979	3.51493	3.78697
H	-1.41112	4.96254	2.75723
C	3.45154	2.70253	-1.04536
H	3.53313	1.79011	-0.41808
C	4.72665	3.53991	-0.80970
H	4.74479	4.45049	-1.44582
H	5.63354	2.94800	-1.05597
H	4.81324	3.87044	0.24628
C	3.35850	2.24423	-2.51709
H	2.47349	1.59563	-2.69151
H	4.26416	1.67204	-2.81093
H	3.27202	3.11246	-3.20517
C	-5.45165	-0.00139	-0.40100
H	-5.94471	1.30140	-2.12779
C	-5.67056	-0.00198	1.12265
H	-5.23671	0.89526	1.61354
H	-6.76391	-0.00232	1.35477
H	-5.65241	-2.20034	-0.60240
C	-6.07615	1.26178	-1.02496
H	-7.17648	1.28447	-0.82959
H	-5.65344	2.19760	-0.60086
H	-5.23629	-0.89936	1.61290
C	-6.07556	-1.26442	-1.02584
H	-5.94411	-1.30321	-2.12870
H	-7.17589	-1.28777	-0.83049
O	-1.74300	-0.00052	-1.28627
C	-1.45306	-0.00070	-2.84339
C	-0.00074	-0.00041	-3.12361
H	-1.98893	0.90665	-3.20220
H	-1.98853	-0.90840	-3.20190
H	0.35684	-0.91090	-3.64455
H	0.35640	0.91002	-3.64497

TS(W-L)

SCF (BP86) Energy = -4006.896400
Enthalpy 0K = -4006.112036
Enthalpy 298K = -4006.060270
Free Energy 298K = -4006.202256
Lowest Frequency = -232.6134 cm^{-1}
Second Frequency = 7.7321 cm^{-1}
SCF (BP86-$D3^{BJ}$) Energy = -4007.114886
SCF ($C_6H_5CH_3$) Energy = -4006.937479
SCF (BS2) Energy = -4008.920021

Zn	3.52171	0.00004	-0.77280
Ca	-0.37413	-0.00000	-0.64250
N	-1.44281	1.54328	0.82494
N	-1.44277	-1.54331	0.82494
C	-2.14761	1.29610	1.94093
C	-2.42711	-0.00003	2.45416
H	-3.00476	-0.00004	3.38929
C	-2.14758	-1.29615	1.94093
C	-2.72396	2.46629	2.72585

H	-1.93338	3.19946	2.98677
H	-3.22181	2.13346	3.65562
H	-3.46398	3.02139	2.11183
C	-2.72390	-2.46636	2.72585
H	-3.46390	-3.02147	2.11183
H	-3.22176	-2.13354	3.65562
H	-1.93330	-3.19950	2.98677
C	-1.27672	2.87480	0.33995
C	-0.10997	3.62987	0.68753
C	0.09290	4.88939	0.08719
H	0.98938	5.47411	0.35263
C	-0.82071	5.41659	-0.83557
H	-0.64178	6.40165	-1.29509
C	-1.96712	4.67975	-1.16414
H	-2.68862	5.09774	-1.88540
C	-2.22083	3.41664	-0.59244
C	0.91137	3.11191	1.70436
H	0.54999	2.12149	2.05396
C	1.01491	4.03189	2.94049
H	1.40693	5.03552	2.67008
H	1.70200	3.59853	3.69811
H	0.02785	4.18107	3.42453
C	2.30247	2.90330	1.06827
H	2.25033	2.19272	0.21481
H	3.02009	2.50077	1.81553
H	2.72337	3.85646	0.68312
C	-3.49017	2.65486	-0.98512
H	-3.53576	1.74510	-0.35055
C	-4.77046	3.47302	-0.71257
H	-4.82730	3.80898	0.34366
H	-5.67535	2.86510	-0.92491
H	-4.82422	4.37915	-1.35297
C	-3.43953	2.18924	-2.45647
H	-3.38940	3.05397	-3.15243
H	-4.34473	1.60056	-2.71707
H	-2.55087	1.55363	-2.65607
C	-1.27665	-2.87483	0.33995
C	-0.10987	-3.62987	0.68753
C	0.09303	-4.88938	0.08720
H	0.98952	-5.47408	0.35264
C	-0.82056	-5.41660	-0.83558
H	-0.64160	-6.40166	-1.29509
C	-1.96699	-4.67979	-1.16415
H	-2.68848	-5.09780	-1.88542
C	-2.22074	-3.41669	-0.59245
C	0.91144	-3.11188	1.70438
H	0.55003	-2.12148	2.05398
C	2.30253	-2.90322	1.06829
H	2.72348	-3.85636	0.68313
H	3.02014	-2.50067	1.81555
H	2.25038	-2.19263	0.21484
C	1.01501	-4.03187	2.94049
H	0.02795	-4.18109	3.42453
H	1.70209	-3.59850	3.69812
H	1.40707	-5.03549	2.67008
C	-3.49010	-2.65495	-0.98513
H	-3.53570	-1.74517	-0.35057
C	-4.77036	-3.47313	-0.71255
H	-4.82411	-4.37927	-1.35295
H	-5.67526	-2.86524	-0.92490
H	-4.82719	-3.80908	0.34368
C	-3.43948	-2.18934	-2.45648
H	-2.55084	-1.55371	-2.65611
H	-4.34470	-1.60069	-2.71708
H	-3.38934	-3.05408	-3.15244
C	5.46050	0.00006	-0.38897
H	5.94057	-1.30205	-2.11994
C	5.69493	0.00012	1.13264
H	5.26515	-0.89702	1.62750
H	6.79044	0.00014	1.35502
H	5.66146	2.19883	-0.59140
C	6.08095	-1.26261	-1.01812
H	7.18303	-1.28590	-0.83190
H	5.66151	-2.19872	-0.59125
H	5.26513	0.89728	1.62744
C	6.08092	1.26270	-1.01821
H	5.94055	1.30207	-2.12003
H	7.18300	1.28603	-0.83199
O	1.75632	0.00002	-1.23535
C	1.40546	0.00001	-2.89312
C	-0.02130	0.00000	-3.14880
H	1.95276	-0.91483	-3.19777
H	1.95275	0.91486	-3.19778
H	-0.43366	0.92235	-3.59928
H	-0.43365	-0.92235	-3.59927

TS(G-X)

SCF (BP86) Energy = -4008.113098
Enthalpy 0K = -4007.310374
Enthalpy 298K = -4007.257681
Free Energy 298K = -4007.401530
Lowest Frequency = -46.5488 cm^{-1}
Second Frequency = 14.5665 cm^{-1}
SCF (BP86-D3^{BJ}) Energy = -4008.346859
SCF ($C_6H_5CH_3$) Energy = -4008.157014
SCF (BS2) Energy = -4010.135394

Zn	0.86357	-3.36311	0.36486
H	0.42950	-3.63686	1.86492
Ca	-0.11033	-0.09623	-0.01424
N	-1.86795	1.38295	0.34189
N	1.12145	1.85651	0.49106
C	-1.85769	2.65935	0.76985
C	-0.68231	3.40567	1.04725
H	-0.86241	4.42816	1.40753
C	0.68790	3.05577	0.91380
C	-3.18185	3.37773	0.97728
H	-3.76529	3.40874	0.03330
H	-3.03482	4.41341	1.33567
H	-3.81611	2.83464	1.70845
C	1.70388	4.12721	1.27791
H	2.41593	3.74990	2.04108
H	1.21650	5.04040	1.66704
H	2.31943	4.40608	0.39727
C	-3.11003	0.72335	0.07447
C	-3.65690	0.75280	-1.24739
C	-4.82745	0.01476	-1.51531
H	-5.25622	0.03110	-2.53019
C	-5.45782	-0.74031	-0.51560

H	-6.37220	-1.31015	-0.74485
C	-4.91411	-0.76758	0.77609
H	-5.40985	-1.36556	1.55807
C	-3.74616	-0.04611	1.09821
C	-2.96550	1.53365	-2.36839
H	-2.26050	2.24308	-1.88673
C	-3.94050	2.36451	-3.22557
H	-4.63435	1.72355	-3.80974
H	-3.38223	2.98842	-3.95487
H	-4.55729	3.04195	-2.59965
C	-2.12701	0.58832	-3.25778
H	-1.39753	-0.00726	-2.66503
H	-1.55729	1.15299	-4.02561
H	-2.77308	-0.14777	-3.78141
C	-3.17323	-0.12213	2.51588
H	-2.34480	0.61577	2.57237
C	-4.20553	0.26878	3.59395
H	-4.63494	1.27517	3.40790
H	-3.73378	0.28076	4.59912
H	-5.04978	-0.45179	3.63622
C	-2.57573	-1.51770	2.80467
H	-3.36694	-2.29783	2.80509
H	-2.08390	-1.54217	3.80009
H	-1.82772	-1.82577	2.04263
C	2.51463	1.59428	0.32345
C	3.16264	1.92050	-0.91231
C	4.50097	1.52341	-1.10102
H	5.00569	1.76381	-2.05075
C	5.20494	0.82417	-0.10946
H	6.24904	0.51894	-0.28139
C	4.56967	0.51696	1.10092
H	5.12739	-0.02651	1.88106
C	3.23182	0.89210	1.34421
C	2.42030	2.65536	-2.03255
H	1.47693	3.04968	-1.60125
C	2.02916	1.68841	-3.17156
H	2.92693	1.24022	-3.64746
H	1.45024	2.21348	-3.96068
H	1.40798	0.84390	-2.80400
C	3.21269	3.85701	-2.58695
H	3.50888	4.55975	-1.78074
H	2.60092	4.42089	-3.32228
H	4.13995	3.53879	-3.10881
C	2.58499	0.55441	2.69049
H	1.55014	0.95993	2.66521
C	3.31140	1.24355	3.86635
H	4.35162	0.86991	3.97603
H	2.78401	1.04657	4.82351
H	3.36497	2.34292	3.72694
C	2.49329	-0.96749	2.92676
H	1.94468	-1.50492	2.12108
H	1.96793	-1.19484	3.87790
H	3.49942	-1.43440	2.98060
C	2.26075	-3.34510	-1.10152
H	3.61912	-4.77481	-0.10383
C	2.34811	-1.92449	-1.68541
H	2.68127	-1.17073	-0.93180
H	3.08981	-1.85301	-2.52266
H	0.86999	-4.06415	-2.66942
C	3.63344	-3.75186	-0.53938
H	4.42176	-3.75087	-1.33688
H	3.98398	-3.06043	0.25798
H	1.36945	-1.60112	-2.11308
C	1.85407	-4.32205	-2.21897
H	1.78871	-5.37191	-1.85703
H	2.60058	-4.32003	-3.05614
H	-1.08857	-4.85692	-0.56688
O	-0.70793	-2.10522	-0.45308
C	-1.73225	-2.82758	-1.11062
C	-1.99114	-4.20761	-0.49975
H	-1.47554	-2.96128	-2.19345
H	-2.68436	-2.24127	-1.08856
H	-2.27242	-4.12915	0.56992
H	-2.80907	-4.73012	-1.04076

X

SCF (BP86) Energy = -4008.120872
Enthalpy 0K = -4007.316324
Enthalpy 298K = -4007.263911
Free Energy 298K = -4007.403769
Lowest Frequency = 10.2214 cm_{-1}
Second Frequency = 16.8494 cm_{-1}
SCF (BP86-D3$_{BJ}$) Energy = -4008.355808
SCF ($C_6H_5CH_3$) Energy = -4008.161186
SCF (BS2) Energy = -4010.142424

Zn	-1.16651	3.35745	-0.01831
H	-1.36391	4.65125	0.86997
Ca	0.11829	0.29451	-0.11047
N	1.88491	-1.27009	-0.08195
N	-1.08533	-1.61707	0.62254
C	1.86631	-2.56958	0.25793
C	0.72768	-3.25196	0.75972
H	0.91621	-4.30049	1.03909
C	-0.61638	-2.83275	0.94514
C	3.11592	-3.43219	0.10684
H	3.93977	-2.89547	-0.39969
H	2.87625	-4.34632	-0.47436
H	3.48438	-3.77282	1.09709
C	-1.53335	-3.88244	1.56609
H	-2.54831	-3.49099	1.76541
H	-1.09803	-4.24151	2.52165
H	-1.62653	-4.77020	0.90648
C	3.08234	-0.58212	-0.42536
C	3.23172	-0.08884	-1.76476
C	4.34167	0.72031	-2.07891
H	4.46365	1.09912	-3.10528
C	5.30213	1.04515	-1.11069
H	6.16182	1.68147	-1.37348
C	5.16111	0.54951	0.19225
H	5.92202	0.80268	0.94864
C	4.06964	-0.26188	0.56488
C	2.24829	-0.50837	-2.86176
H	1.26027	-0.69005	-2.37277
C	2.65782	-1.86675	-3.47559
H	3.64972	-1.78385	-3.96841
H	1.92155	-2.19768	-4.23852
H	2.72927	-2.65749	-2.70296
C	2.04611	0.54733	-3.96343

H	1.80842	1.54784	-3.54595
H	1.21671	0.25014	-4.63786
H	2.95127	0.65771	-4.59682
C	3.97920	-0.76172	2.01105
H	3.10999	-1.44670	2.07922
C	5.23602	-1.55750	2.42701
H	5.45357	-2.38636	1.72331
H	5.10405	-1.99321	3.43997
H	6.13648	-0.90821	2.46448
C	3.71901	0.39014	3.00472
H	4.54646	1.13125	2.99420
H	3.63043	0.00116	4.04134
H	2.78339	0.93308	2.76495
C	-2.47483	-1.30907	0.61793
C	-3.35231	-1.85088	-0.38160
C	-4.68635	-1.39666	-0.42699
H	-5.36539	-1.80622	-1.19277
C	-5.16973	-0.43564	0.47059
H	-6.21444	-0.09273	0.40886
C	-4.31252	0.08396	1.45079
H	-4.69900	0.82919	2.16248
C	-2.97042	-0.33514	1.54923
C	-2.89964	-2.90323	-1.40183
H	-1.84923	-3.17303	-1.17004
C	-2.91681	-2.36159	-2.84812
H	-3.94005	-2.06020	-3.15799
H	-2.56724	-3.13899	-3.56031
H	-2.26084	-1.47617	-2.96620
C	-3.74553	-4.19297	-1.30897
H	-3.76466	-4.60278	-0.27916
H	-3.33867	-4.97663	-1.98267
H	-4.79821	-4.01162	-1.61370
C	-2.09125	0.16742	2.69901
H	-1.03103	0.13839	2.35308
C	-2.17078	-0.80072	3.90205
H	-3.20544	-0.83451	4.30491
H	-1.49533	-0.47230	4.72023
H	-1.88624	-1.83296	3.61673
C	-2.39235	1.60945	3.13982
H	-2.33440	2.32823	2.29532
H	-1.65696	1.93828	3.90178
H	-3.39908	1.70279	3.59858
C	-1.90708	2.60102	-1.77569
H	-3.82181	3.60880	-1.34892
C	-1.46129	1.22512	-2.28710
H	-1.84347	0.38348	-1.65683
H	-1.86667	1.00263	-3.30822
H	-0.32760	3.68241	-2.90622
C	-3.44352	2.62174	-1.69206
H	-3.91146	2.42408	-2.69174
H	-3.83871	1.85458	-0.99149
H	-0.35208	1.14883	-2.42075
C	-1.43472	3.66460	-2.78957
H	-1.74972	4.68919	-2.49444
H	-1.86374	3.47632	-3.80847
H	0.09360	3.84781	2.86731
O	0.47720	2.27823	0.65405
C	1.42944	3.09591	1.32443
C	1.07507	3.33672	2.79132
H	1.51554	4.08388	0.80926
H	2.43915	2.62357	1.24822
H	1.01500	2.37918	3.35197
H	1.84056	3.97443	3.28348

TS(X-V)

SCF (BP86) Energy = -4008.046185
Enthalpy 0K = -4007.247260
Enthalpy 298K = -4007.195112
Free Energy 298K = -4007.336752
Lowest Frequency = -621.0685 cm^{-1}
Second Frequency = 11.6447 cm^{-1}
SCF (BP86-D3^{BJ}) Energy = -4008.271434
SCF ($C_6H_5CH_3$) Energy = -4008.086750
SCF (BS2) Energy = -4010.068684

Zn	-3.11124	-1.85912	-1.01988
H	-2.99069	-1.25735	-2.87273
Ca	0.38812	0.10347	-0.44081
N	2.41406	-0.23999	0.68495
N	0.51037	2.11128	0.75402
C	3.06131	0.54525	1.56052
C	2.59674	1.81880	1.98985
H	3.24742	2.32371	2.71759
C	1.43494	2.54942	1.62475
C	4.38216	0.06899	2.14458
H	4.25752	-0.90822	2.65564
H	4.80001	0.79559	2.86595
H	5.12884	-0.09988	1.34060
C	1.25039	3.91211	2.27374
H	0.27876	3.96514	2.80772
H	1.22170	4.71370	1.50630
H	2.06111	4.14082	2.99026
C	2.94063	-1.50524	0.28346
C	2.52618	-2.69960	0.95712
C	2.95305	-3.94548	0.45334
H	2.63721	-4.86881	0.96622
C	3.76856	-4.03617	-0.68299
H	4.08823	-5.02056	-1.06017
C	4.17492	-2.86347	-1.33533
H	4.81774	-2.93731	-2.22774
C	3.77967	-1.59123	-0.87485
C	1.63884	-2.65699	2.20468
H	1.44591	-1.58672	2.43147
C	2.34615	-3.26688	3.43461
H	2.54179	-4.35161	3.29604
H	1.71694	-3.15663	4.34314
H	3.32124	-2.77661	3.63557
C	0.27317	-3.33775	1.96340
H	-0.27446	-2.88188	1.11096
H	-0.36805	-3.25585	2.86702
H	0.39626	-4.42001	1.74343
C	4.23289	-0.33801	-1.62934
H	3.93847	0.53897	-1.01548
C	5.76391	-0.27879	-1.81187
H	6.29634	-0.36531	-0.84200
H	6.06390	0.68232	-2.28032
H	6.13433	-1.09404	-2.46910
C	3.51189	-0.21102	-2.98953
H	3.75624	-1.06586	-3.65585

H	3.81149	0.72171	-3.51318
H	2.40461	-0.19301	-2.87731
C	-0.62782	2.89505	0.40177
C	-1.87342	2.68431	1.07787
C	-3.02223	3.35929	0.61663
H	-3.98350	3.20179	1.13308
C	-2.96766	4.22800	-0.48203
H	-3.87746	4.74336	-0.82839
C	-1.74469	4.43748	-1.13518
H	-1.70450	5.12428	-1.99649
C	-0.56464	3.79033	-0.71587
C	-1.97904	1.75236	2.28819
H	-0.95952	1.35900	2.48664
C	-2.89922	0.54469	2.00806
H	-3.94526	0.86708	1.81932
H	-2.91533	-0.14988	2.87422
H	-2.56778	-0.03567	1.11989
C	-2.43873	2.50196	3.55738
H	-1.77582	3.36124	3.78785
H	-2.43507	1.82418	4.43710
H	-3.47064	2.89797	3.44655
C	0.74651	4.04680	-1.46482
H	1.55387	3.54050	-0.89530
C	1.09917	5.54786	-1.53176
H	0.36466	6.11777	-2.13962
H	2.09624	5.69398	-1.99827
H	1.12491	6.00944	-0.52285
C	0.72293	3.42608	-2.87900
H	0.53323	2.33104	-2.84958
H	1.69188	3.58596	-3.39793
H	-0.07400	3.87962	-3.50626
C	-4.87752	-2.53464	-0.35809
H	-6.02463	-1.03744	-1.51637
C	-4.76127	-3.00419	1.10183
H	-4.47376	-2.18046	1.78964
H	-5.74194	-3.40280	1.46563
H	-4.56419	-4.56091	-1.19991
C	-5.91949	-1.40880	-0.47401
H	-6.92835	-1.77321	-0.15519
H	-5.67399	-0.53658	0.16968
H	-4.01659	-3.81963	1.22660
C	-5.28223	-3.71430	-1.26117
H	-5.36059	-3.41908	-2.32974
H	-6.28185	-4.11555	-0.95809
H	-2.22347	-0.81263	-3.06669
O	-1.22541	-1.47587	-0.71451
C	-0.65785	-1.75775	-2.02486
C	-0.58169	-0.47359	-2.80783
H	-1.33074	-2.54164	-2.51092
H	0.32690	-2.28672	-1.91794
H	-1.15831	0.41478	-2.49277
H	-0.06912	-0.41909	-3.78150

Et_2O

SCF (BP86) Energy = -233.480709
Enthalpy 0K = -233.348848
Enthalpy 298K = -233.340964
Free Energy 298K = -233.378424
Lowest Frequency = 99.7667 cm^{-1}
Second Frequency = 103.5995 cm^{-1}
SCF (BP86-D3^{BJ}) Energy = -233.493785
SCF ($C_6H_5CH_3$) Energy = -233.485315
SCF (BS2) Energy = -233.749444

H	-0.89721	2.37881	1.06603
O	-0.00000	0.00000	0.25966
C	0.00000	-1.18421	-0.51719
C	0.00000	-2.38850	0.41377
H	0.89644	-1.21018	-1.19116
H	-0.89644	-1.21018	-1.19116
H	-0.89721	-2.37881	1.06603
H	0.00000	-3.33304	-0.16786
H	0.89721	-2.37881	1.06603
C	-0.00000	1.18421	-0.51719
C	-0.00000	2.38850	0.41377
H	-0.89644	1.21018	-1.19116
H	0.89644	1.21018	-1.19116
H	0.89721	2.37881	1.06603
H	-0.00000	3.33304	-0.16786

$C_6H_5CH_3$ (C_7H_8)

SCF (BP86) Energy = -271.362925
Enthalpy 0K = -271.238967
Enthalpy 298K = -271.231609
Free Energy 298K = -271.270575
Lowest Frequency = 14.1639 cm^{-1}
Second Frequency = 204.6014 cm^{-1}
SCF (BP86-D3^{BJ}) Energy = -271.384694
SCF ($C_6H_5CH_3$) Energy = -271.371061
SCF (BS2) Energy = -271.656061

C	-1.91810	-0.00347	0.00668
C	-1.21269	1.21174	0.00175
C	-1.20497	-1.21583	0.00176
H	-1.76006	2.16823	0.00168
H	-1.74754	-2.17510	0.00175
C	0.19336	1.21267	-0.00716
C	0.19949	-1.20924	-0.00722
H	0.73772	2.17194	-0.01398
H	0.74832	-2.16616	-0.01409
C	0.92404	0.00454	-0.00990
H	-3.01978	-0.00687	0.01140
C	2.43722	0.00226	0.00716
H	2.85155	0.99284	-0.26820
H	2.82715	-0.25046	1.01788
H	2.85258	-0.75051	-0.69489

C_2H_4

SCF (BP86) Energy = -78.524435
Enthalpy 0K = -78.475166
Enthalpy 298K = -78.471139
Free Energy 298K = -78.496085
Lowest Frequency = 795.2523 cm^{-1}
Second Frequency = 920.4947 cm^{-1}
SCF (BP86-D3^{BJ}) Energy = -78.527754
SCF ($C_6H_5CH_3$) Energy = -78.525489
SCF (BS2) Energy = -78.615512

C	0.00000	0.00000	0.67102
C	0.00000	-0.00000	-0.67102
H	0.00000	0.93807	1.25174
H	-0.00000	-0.93807	1.25174
H	0.00000	0.93807	-1.25174
H	-0.00000	-0.93807	-1.25174

H_2

SCF (BP86) Energy = -1.172397
Enthalpy 0K = -1.162653
Enthalpy 298K = -1.159349
Free Energy 298K = -1.174202
Lowest Frequency = 4276.9563 cm^{-1}
SCF (BP86-$D3^{BJ}$) Energy = -1.172508
SCF ($C_6H_5CH_3$) Energy = -1.171941
SCF (BS2) Energy = -1.177713

H	0.00000	0.00000	0.38368
H	0.00000	0.00000	-0.38368
